# Supplementary material for: Multidirectional desymmetrization of pluripotent building block en route to diastereoselective synthesis of complex nature-inspired scaffolds
Source: Nat Commun. 2018 Nov 26;9:4989. doi: 10.1038/s41467-018-07521-2 (PMC6255838; doi:10.1038/s41467-018-07521-2)
Supplement: Supplementary file 1 — Supplementary Information [file 41467_2018_7521_MOESM1_ESM.pdf]

Multidirectional Desymmetrization of Pluripotent Building Block *en Route* to Diastereoselective Synthesis of Complex Nature-Inspired Scaffolds

Vunnam et al.

Supplementary Tables

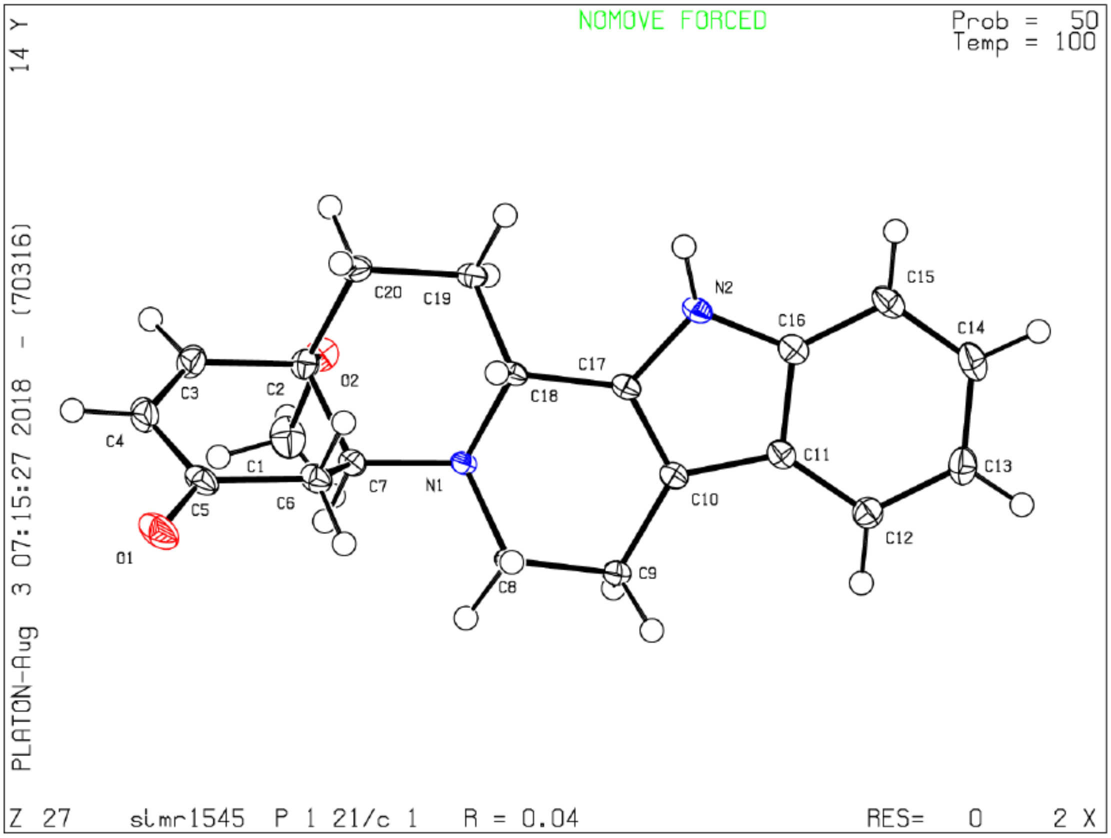

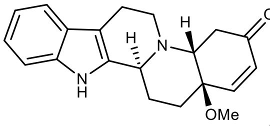

≡
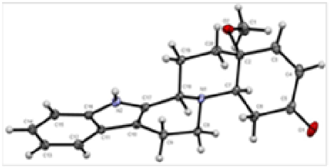

5a

Identification code	SIMR-1545	
Empirical formula	$C_{20}H_{22}N_2O_2$	
Formula weight	322.39	
Temperature	100(2) K	
Wavelength	0.71073Å	
Crystal system	Monoclinic	
Space group	P_12_1/c_1	
Unit cell dimensions	a = 10.612(5) Å	α = 90°
	b = 16.433(7) Å	β = 114.413(7)

	c = 10.452(5) Å γ = 90°
Volume	1659.8(13) Å^3
Z	4
Density (calculated)	1.290 g cm$^{–3}$
Absorption coefficient	0.084 mm$^{–1}$
F(000)	688
Crystal size	0.2 × 0.05 × 0.05 mm^3
Reflections collected	39285
Independent reflections	4184 [R(int) = 0.0355]
Absorption correction	multi-scan
Max. and min. transmission	0.7137and 0.7457
Refinement method	Least Squares minimization
Data / restraints / parameters	4184 / 0 / 218
Goodness-of-fit on F2	0.916
Final R indices [I>2sigma(I)]	R_1 = 0.0385, wR_2 = 0.0965
R indices (all data)	R_1 = 0.0473, wR_2 = 0.1041
Largest diff. peak and hole	0.38 and –0.23 e.Å^{-3}

Supplementary Table 1 X-ray crystal structure and crystallographic data for compound **5a**

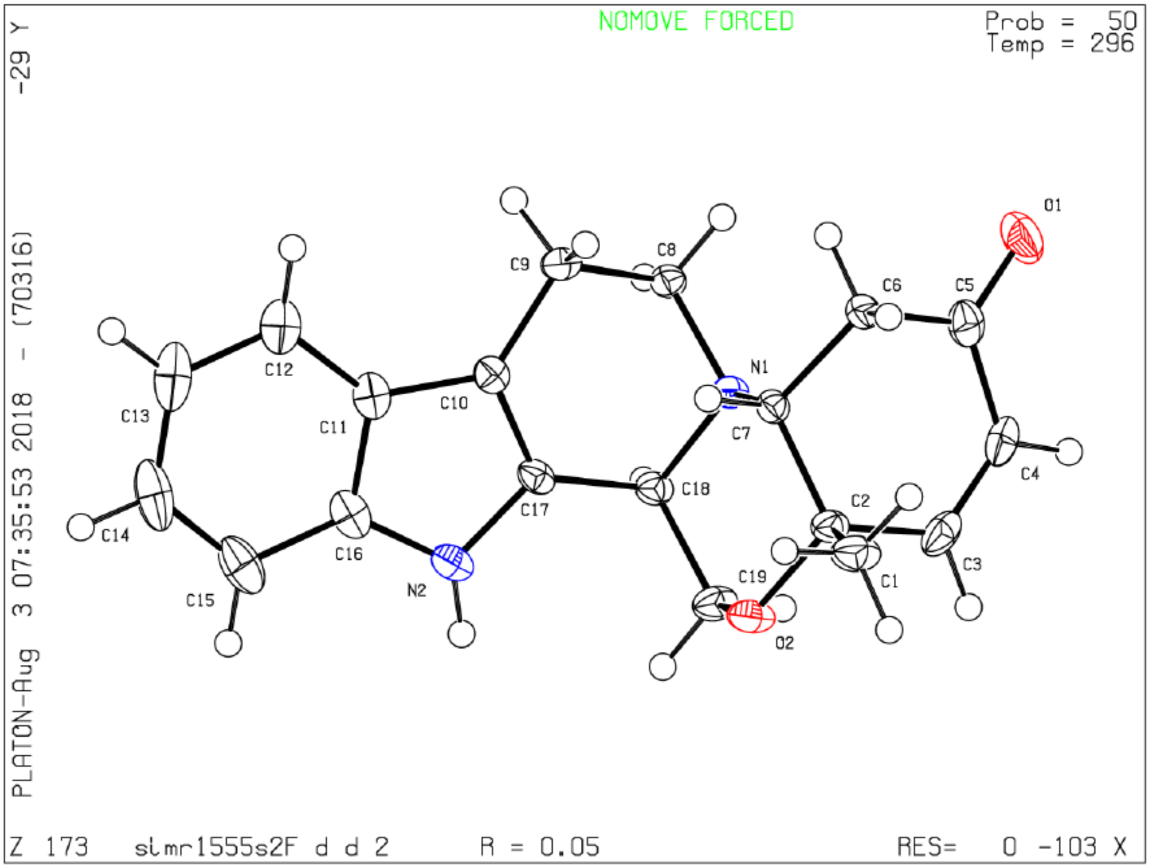

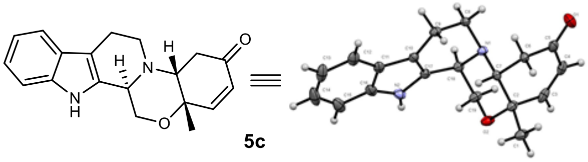

5c

Identification code	SIMR-1555s2	
Empirical formula	$C_{19}H_{20}N_2O_2$	
Formula weight	308.37	
Temperature	296(2) K	
Wavelength	0.71073 Å	
Crystal system	Orthorhombic	
Space group	Fdd_2	
Unit cell dimensions	a = 28.514(6) Å	α = 90°
	b = 31.144(7) Å	β = 90°
	c = 7.0650(15) Å	γ = 90°

Volume	6274(2) $Å^3$
Z	16
Density (calculated)	1.306 g cm^{-3}
Absorption coefficient	0.085 mm^{-1}
F(000)	2624
Crystal size	0.4 × 0.1 × 0.1 mm^3
Reflections collected	31030
Independent reflections	3901 [R(int) = 0.1374]
Absorption correction	multi-scan
Max. and min. transmission	0.6670 and 0.7457
Refinement method	Least Squares minimization
Data / restraints / parameters	3901 / 0 /209
Goodness-of-fit on F2	1.012
Final R indices [I>2sigma(I)]	R_1 = 0.0532, wR_2 = 0.0915
R indices (all data)	R_1 = 0.1074, wR_2 = 0.1094
Largest diff. peak and hole	0.21 and –0.23 e.$Å^{-3}$

Supplementary Table 2 X-ray crystal structure and crystallographic data for compound **5c**

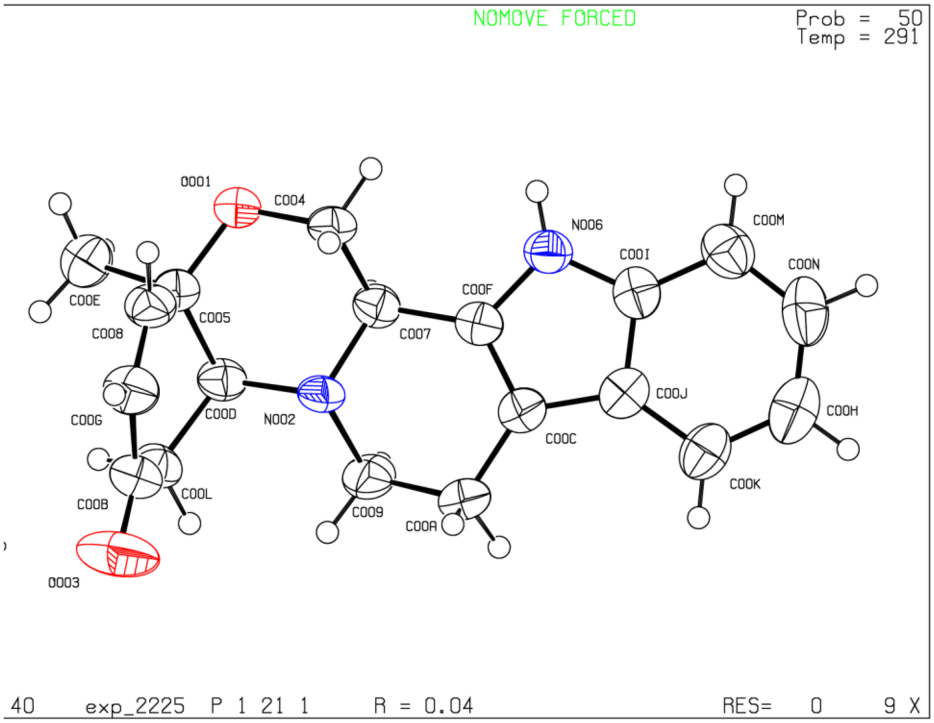

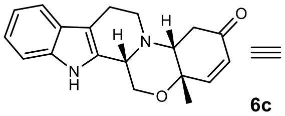

6c

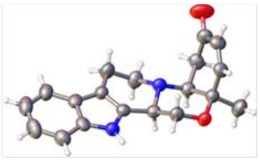

Identification code	exp_2225	
	SIMR-1555	
Empirical formula	$C_{19}H_{20}N_2O_2$	
Formula weight	308.38	
Temperature	291(2) K	
Wavelength	0.71073 Å	
Crystal system	Monoclinic	
Space group	$P2_1$	
Unit cell dimensions	a = 9.9579(11) Å	α = 90°
	b = 9.2394(6) Å	β = 119.203(16)°
	c = 10.0669(12) Å	γ = 90°
Volume	808.48(19) $Å^3$	

Z	2
Density (calculated)	1.2667 g cm^{-3}
Absorption coefficient	0.083 mm^{-1}
F(000)	328.1
Crystal size	0.8 × 0.2 × 0.1 mm^3
Theta range for data collection	6.4 to 58.64°.
Index ranges	$-11 \le h \le 13$, $-12 \le k \le 7$, $-7 \le l \le 12$
Reflections collected	2626
Independent reflections	2051 [R(int) = 0.0170]
Absorption correction	multi-scan
Max. and min. transmission	0.210 and 1.000
Refinement method	Least Squares minimization
Data / restraints / parameters	4630 / 0 /275
Goodness-of-fit on F2	1.028
Final R indices [I>2sigma(I)]	R_1 = 0.0420, wR_2 = 0.0981
R indices (all data)	R_1 = 0.0574, wR_2 = 0.1083
Largest diff. peak and hole	0.12 and –0.13 e.$Å^{-3}$

Supplementary Table 3 X-ray crystal structure and crystallographic data for compound **6c**

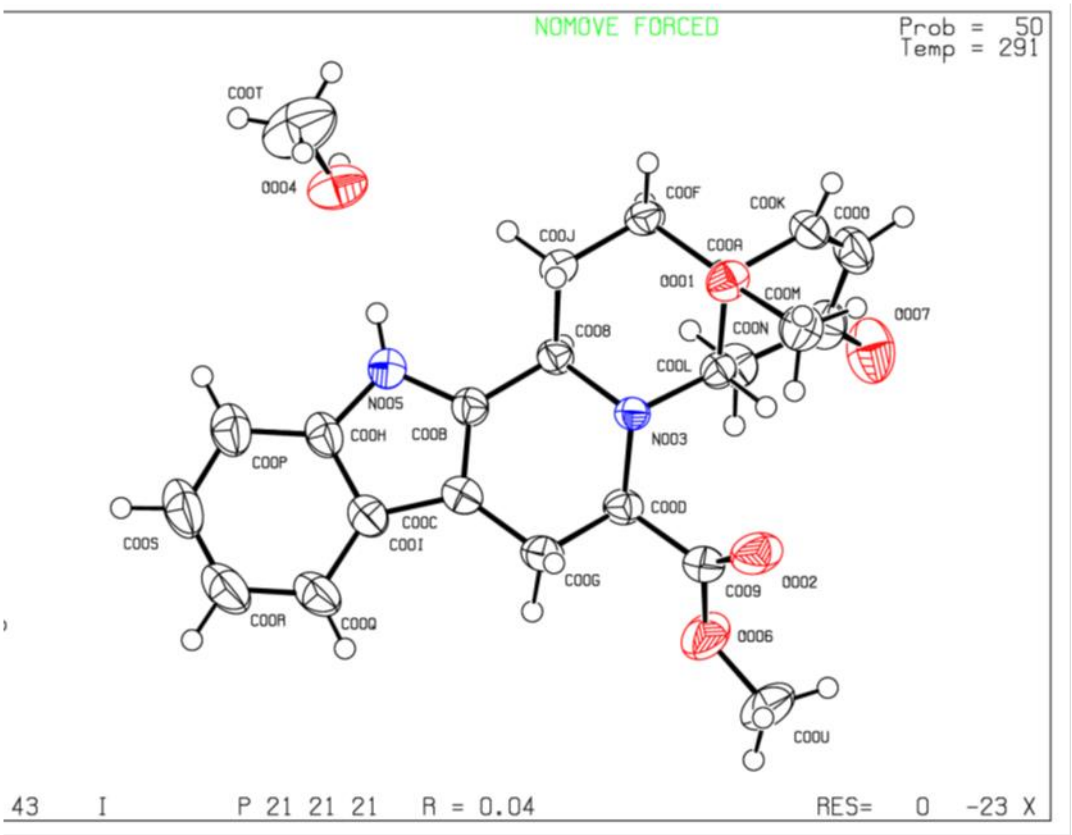

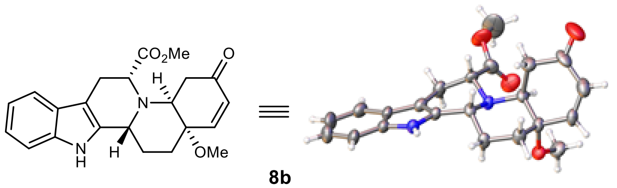

8b

Identification code	exp_2229	
	SIMR-1549	
Empirical formula	$C_{22}H_{24}N_2O_4 \cdot CH_4O$	
Formula weight	412.49	
Temperature	291(2) K	
Wavelength	0.71073 Å	
Crystal system	orthorhombic	
Space group	$P2_12_12_1$	
Unit cell dimensions	a = 7.0712(7) Å	α = 90°
	b = 8.8316(9) Å	β = 90°
	c = 34.544(3) Å	γ = 90°

Volume	2157.3(4) Å^3
Z	4
Density (calculated)	1.2699 g cm^{-3}
Absorption coefficient	0.090 mm^{-1}
F(000)	880.5
Crystal size	0.8 × 0.4 × 0.2 mm^3
Theta range for data collection	2.297 to 28.349°.
Index ranges	$8 \leq h \leq 9$, $-10 \leq k \leq 12$, $-43 \leq l \leq 44$
Reflections collected	8073
Independent reflections	4630 [R(int) = 0.0166]
Completeness to theta = 29.25°	99.9 %
Absorption correction	multi-scan
Max. and min. transmission	0.724 and 1.000
Refinement method	Least Squares minimization
Data / restraints / parameters	4630 / 0 /275
Goodness-of-fit on F2	1.069
Final R indices [I>2sigma(I)]	R_1 = 0.0428, wR_2 = 0.0966
R indices (all data)	R_1 = 0.0530, wR_2 = 0.1026
Largest diff. peak and hole	0.38 and –0.23 e.Å^{-3}

Supplementary Table 4 X-ray crystal structure and crystallographic data for compound **8b**

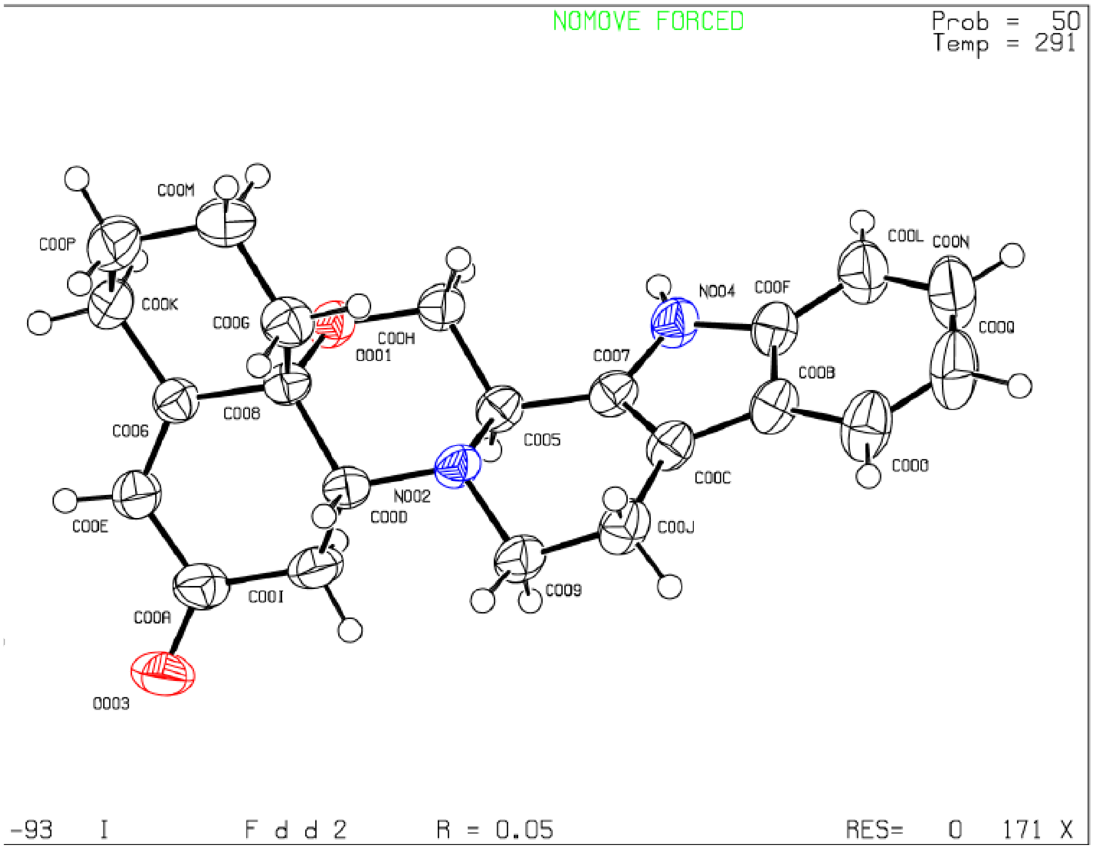

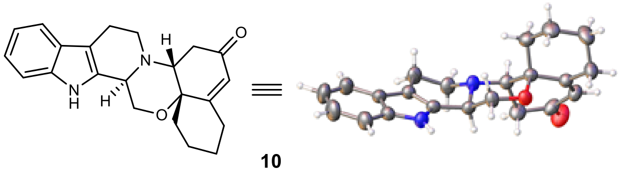

10

Identification code	exp_2239	
	SIMR-1805	
Empirical formula	$C_{22}H_{24}N_2O_2$·solvent	
Formula weight	348.43	
Temperature	291(2) K	
Wavelength	0.71073 Å	
Crystal system	monoclinic	
Space group	$P2_1/c$	
Unit cell dimensions	a = 13.4439(9) Å	α = 90°
	b = 9.8893(6) Å	β = 108.931(7) °

	c = 17.2687(11) Å γ = 90°
Volume	2171.7(3)$Å^3$
Z	4
Density (calculated)	1.066 g cm^{-3}
Absorption coefficient	0.069 mm^{-1}
F(000)	744.0
Crystal size	0.6 × 0.2 × 0.04 mm^3
Theta range for data collection	6.408 to 58.694°.
Index ranges	-17 ≤ h ≤ 16, -13 ≤ k ≤ 11, -23 ≤ l ≤ 22
Reflections collected	10757
Independent reflections	5072 [R(int) = 0.0233]
Absorption correction	multi-scan
Max. and min. transmission	0.800 and 1.000
Refinement method	Least Squares minimization
Data / restraints / parameters	5072 / 0 /236
Goodness-of-fit on F2	1.005
Final R indices [I>2sigma(I)]	R_1 = 0.0486, wR_2 = 0.1359
R indices (all data)	R_1 = 0.0795, wR_2 = 0.1518
Largest diff. peak and hole	0.18 and –0.17 e.$Å^{-3}$

Supplementary Table 5 X-ray crystal structure and crystallographic data for compound **10**

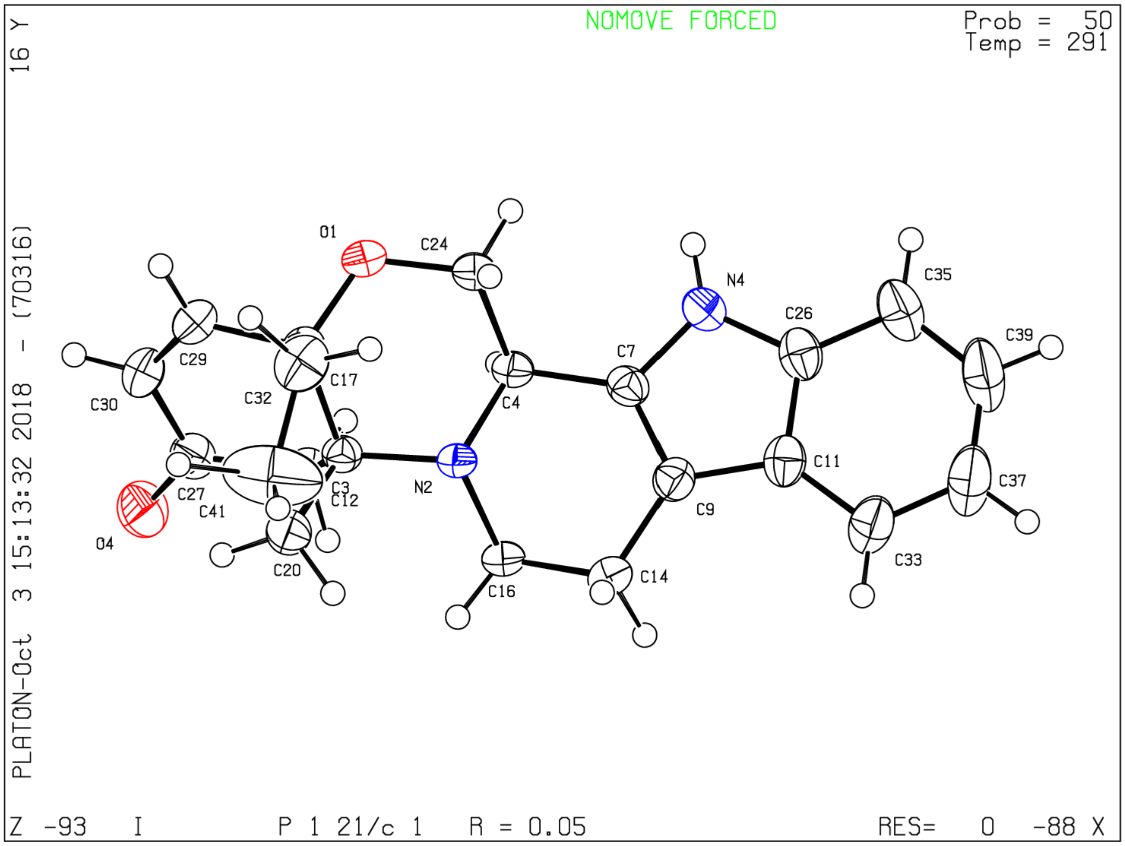

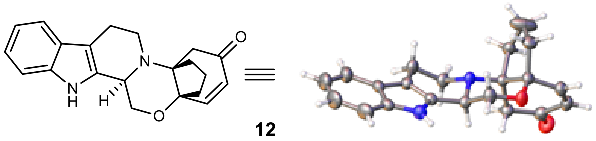

12

Identification code	exp_2222	
	SIMR-1811	
Empirical formula	$C_{21}H_{22}N_2O_2$	
Formula weight	334.40	
Temperature	291(2) K	
Wavelength	0.71073 Å	
Crystal system	monoclinic	
Space group	$P2_1/c$	
Unit cell dimensions	a = 7.0056(3) Å	α = 90.002(6)°
	b = 13.8119(9) Å	β = 90.041(5)°

	c = 18.7722(17)Å γ = 103.969(5)°
Volume	1762.7(2)Å^3
Z	4
Density (calculated)	1.260 g cm^{-3}
Absorption coefficient	0.082 mm^{-1}
F(000)	712.3
Crystal size	0.8 × 0.4 × 0.2 mm^3
Theta range for data collection	6.02 to 58.84°.
Index ranges	$-9 \le h \le 9$, $-15 \le k \le 24$, $-17 \le l \le 12$
Reflections collected	8619
Independent reflections	4115 [R(int) = 0.0175]
Absorption correction	multi-scan
Max. and min. transmission	0.902 and 1.000
Refinement method	Least Squares minimization
Data / restraints / parameters	4115/ 0 /234
Goodness-of-fit on F2	1.035
Final R indices [I>2sigma(I)]	R_1 = 0.0459, wR_2 = 0.1208
R indices (all data)	R_1 = 0.0596, wR_2 = 0.1306
Largest diff. peak and hole	0.34 and –0.22 e.Å^{-3}

Supplementary Table 6 X-ray crystal structure and crystallographic data for compound **12**

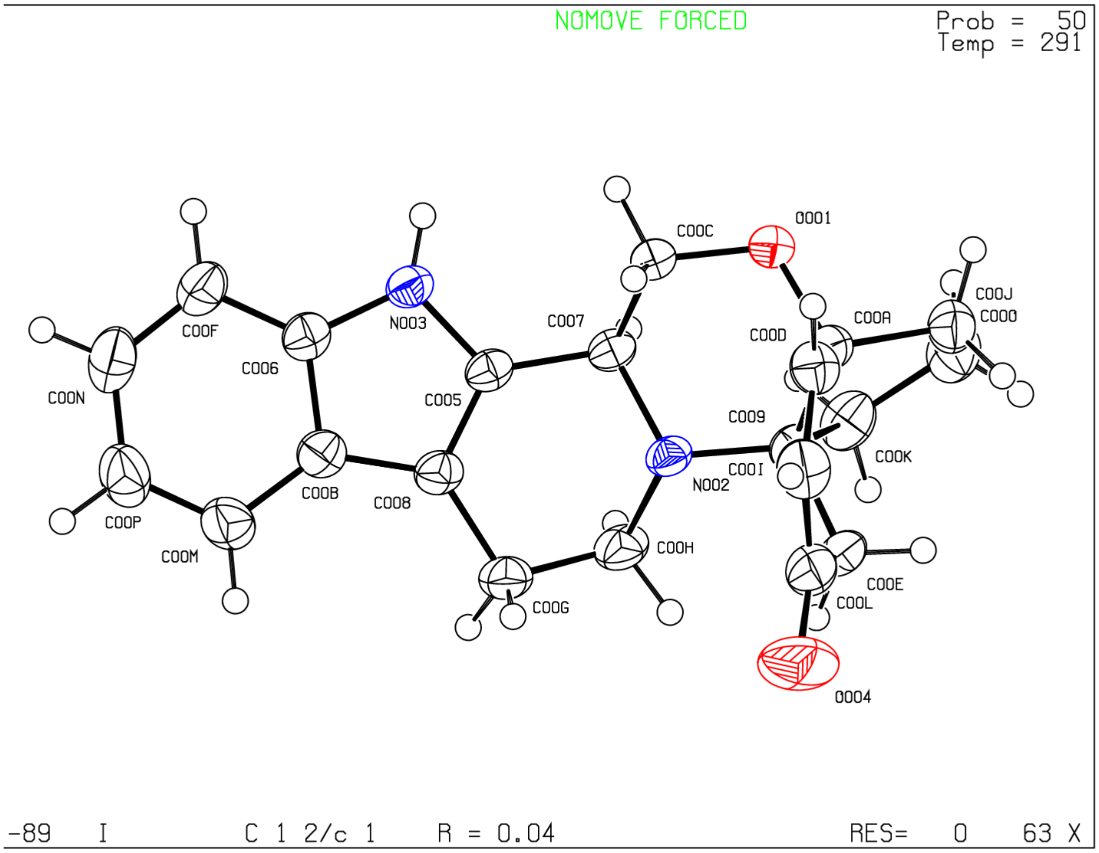

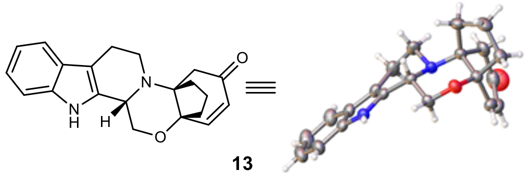

13

Identification code	exp_2227	
	SIMR-1813	
Empirical formula	$C_{21}H_{22}N_2O_2$	
Formula weight	334.40	
Temperature	291(2) K	
Wavelength	0.71073 Å	
Crystal system	monoclinic	
Space group	C2/c	
Unit cell dimensions	a = 18.3021(11) Å	α = 90 °
	b = 9.0743(5) Å	β = 90.400(6)°

	c = 20.5335(13) Å γ = 90°
Volume	3410.1(4)Å^3
Z	8
Density (calculated)	1.303 g cm^{-3}
Absorption coefficient	0.084 mm^{-1}
F(000)	1424.0
Crystal size	0.8 × 0.4 × 0.1 mm^3
Theta range for data collection	6.382 to 58.49°.
Index ranges	-23 ≤ h ≤ 22, -12 ≤ k ≤ 10, -27 ≤ l ≤ 16
Reflections collected	6251
Independent reflections	3633 [R(int) = 0.0130]
Absorption correction	multi-scan
Max. and min. transmission	0.836 and 1.000
Refinement method	Least Squares minimization
Data / restraints / parameters	3633/ 0 /226
Goodness-of-fit on F2	1.051
Final R indices [I>2sigma(I)]	R_1 = 0.0446, wR_2 = 0.1075
R indices (all data)	R_1 = 0.0583, wR_2 = 0.1160
Largest diff. peak and hole	0.22 and –0.21 e.Å^{-3}

Supplementary Table 7 X-ray crystal structure and crystallographic data for compound **13**

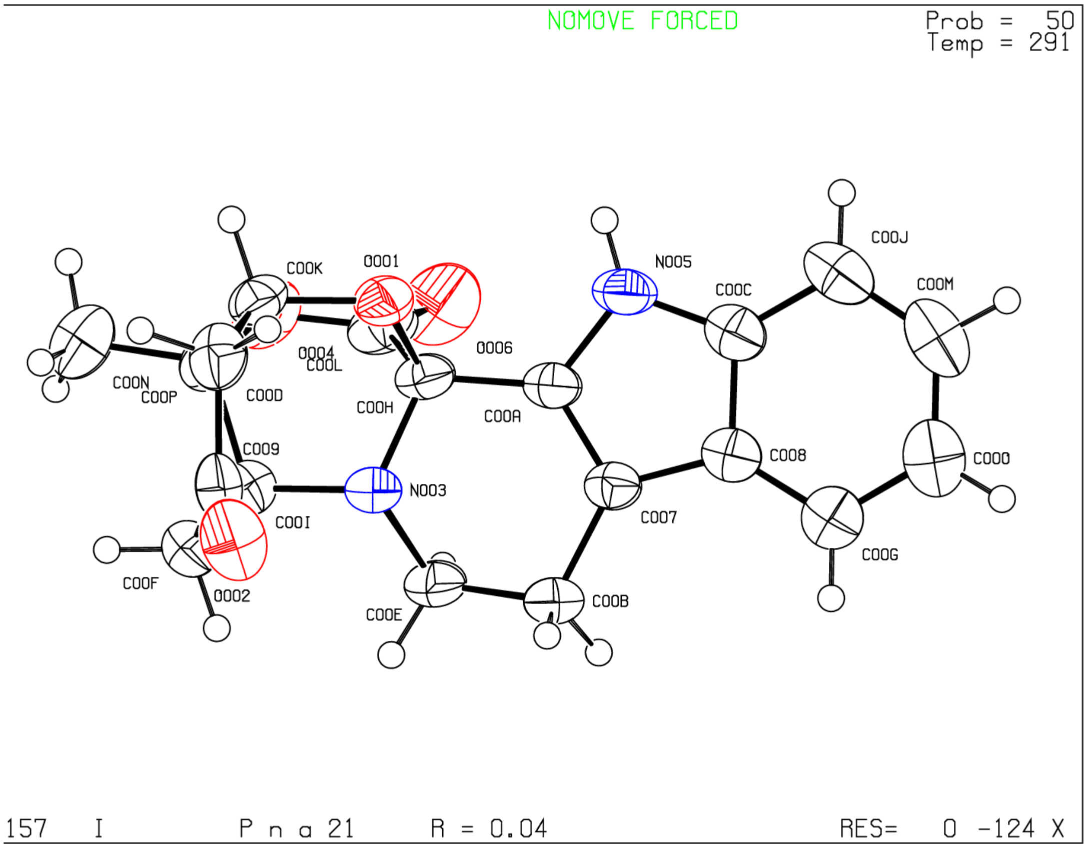

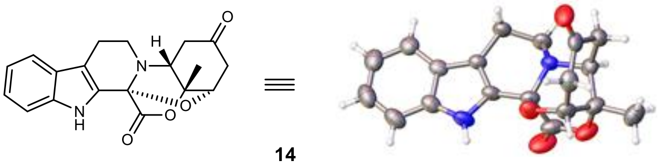

14

Identification code	exp_2268	
	SIMR-1870	
Empirical formula	$C_{19}H_{18}N_2O_4$·	
Formula weight	338.35	
Temperature	291(2) K	
Wavelength	0.71073 Å	
Crystal system	orthorhombic	
Space group	$Pna2_1$	
Unit cell dimensions	a = 12.0313(8) Å	α = 90 °
	b = 17.8969(12) Å	β = 90°
	c = 7.7473(5) Å	γ = 90°

Volume	1668.17(19)Å^3
Z	4
Density (calculated)	1.347 g cm^{-3}
Absorption coefficient	0.096 mm^{-1}
F(000)	712.0
Crystal size	0.4 × 0.2 × 0.1 mm^3
Theta range for data collection	6.656 to 58.596°.
Index ranges	$-13 \le h \le 15$, $-20 \le k \le 23$, $-6 \le l \le 10$
Reflections collected	4992
Independent reflections	2952 [R(int) = 0.0172]
Absorption correction	multi-scan
Max. and min. transmission	0.928 and 1.000
Refinement method	Least Squares minimization
Data / restraints / parameters	2952/ 1 /227
Goodness-of-fit on F2	1.081
Final R indices [I>2sigma(I)]	R_1 = 0.0416, wR_2 = 0.0829
R indices (all data)	R_1 = 0.0524, wR_2 = 0.0878
Largest diff. peak and hole	0.14 and –0.14 e.Å^{-3}

Supplementary Table 8 X-ray crystal structure and crystallographic data for compound **14**

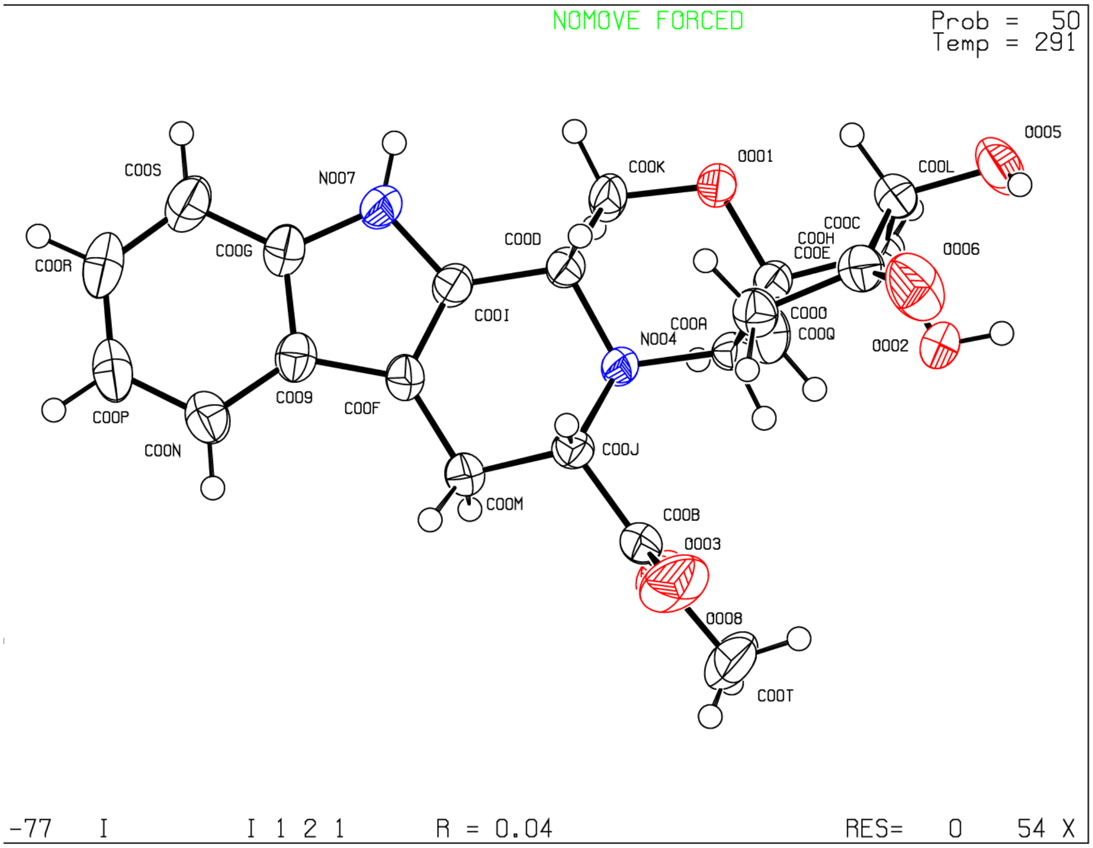

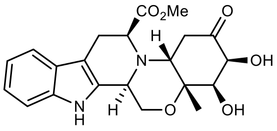

≡

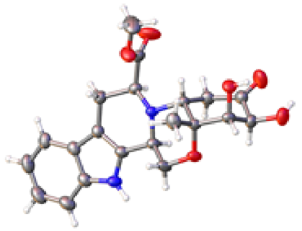

16

Identification code	exp_2305	
	SIMR-1984	
Empirical formula	$C_{21}H_{24}N_2O_6$	
Formula weight	400.42	
Temperature	291(2) K	
Wavelength	0.71073 Å	
Crystal system	monoclinic	
Space group	I2	
Unit cell dimensions	a = 9.7616(6) Å	α = 90 °

	b = 8.9376(4) Å β = 97.650(5)°
	c = 23.1897(12) Å γ = 90°
Volume	2005.18(19)$Å^3$
Z	4
Density (calculated)	1.326 g cm^{-3}
Absorption coefficient	0.098 mm^{-1}
F(000)	848.0
Crystal size	0.8 × 0.6 × 0.2 mm^3
Theta range for data collection	6.206 to 58.174°.
Index ranges	$-13 \leq h \leq 9$, $-7 \leq k \leq 11$, $-31 \leq l \leq 25$
Reflections collected	4932
Independent reflections	3397 [R(int) = 0.0152]
Absorption correction	multi-scan
Max. and min. transmission	0.959 and 1.000
Refinement method	Least Squares minimization
Data / restraints / parameters	3397/ 1 /267
Goodness-of-fit on F2	1.042
Final R indices [I>2sigma(I)]	R_1 = 0.0350, wR_2 = 0.0836
R indices (all data)	R_1 = 0.0429, wR_2 = 0.0889
Largest diff. peak and hole	0.18 and –0.16 e.$Å^{-3}$

Supplementary Table 9 X-ray crystal structure and crystallographic data for compound **16**

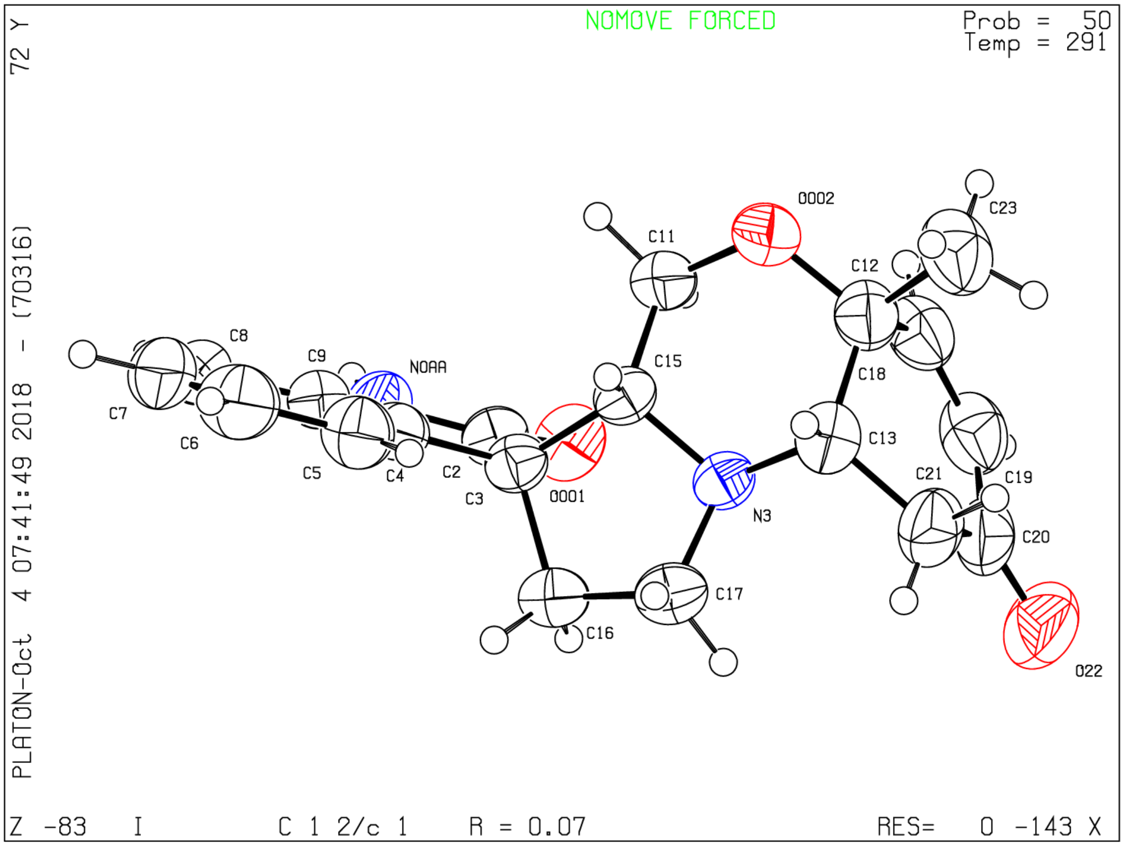

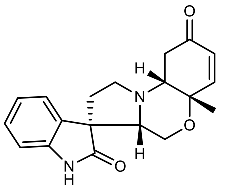

≡ **19**

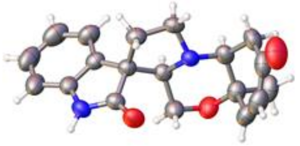

Identification code	exp_2276	
	SIMR-1890	
Empirical formula	$C_{19}H_{20}N_2O_3$	
Formula weight	324.37	
Temperature	291(2) K	
Wavelength	0.71073 Å	
Crystal system	Monoclinic	
Space group	C2/c	
Unit cell dimensions	a = 21.571(4) Å	α = 90 °
	b = 8.7569(9) Å	β = 125.59(3)°

	c = 21.547(4) Å γ = 90°
Volume	3310.0(13)$Å^3$
Z	8
Density (calculated)	1.302 g cm^{-3}
Absorption coefficient	0.089 mm^{-1}
F(000)	1376.0
Crystal size	0.4 × 0.08 × 0.04 mm^3
Theta range for data collection	7.358 to 58.59°.
Index ranges	-28 ≤ h ≤ 18, -11 ≤ k ≤ 11, -28 ≤ l ≤ 29
Reflections collected	8640
Independent reflections	3863 [R(int) = 0.0366]
Absorption correction	multi-scan
Max. and min. transmission	0.953 and 1.000
Refinement method	Least Squares minimization
Data / restraints / parameters	3863/ 0 /280
Goodness-of-fit on F2	1.022
Final R indices [I>2sigma(I)]	R_1 = 0.0713, wR_2 = 01754
R indices (all data)	R_1 = 0.1355, wR_2 = 0.2141
Largest diff. peak and hole	1.02 and –0.15 e.$Å^{-3}$

Supplementary Table 10 X-ray crystal structure and crystallographic data for compound **19**

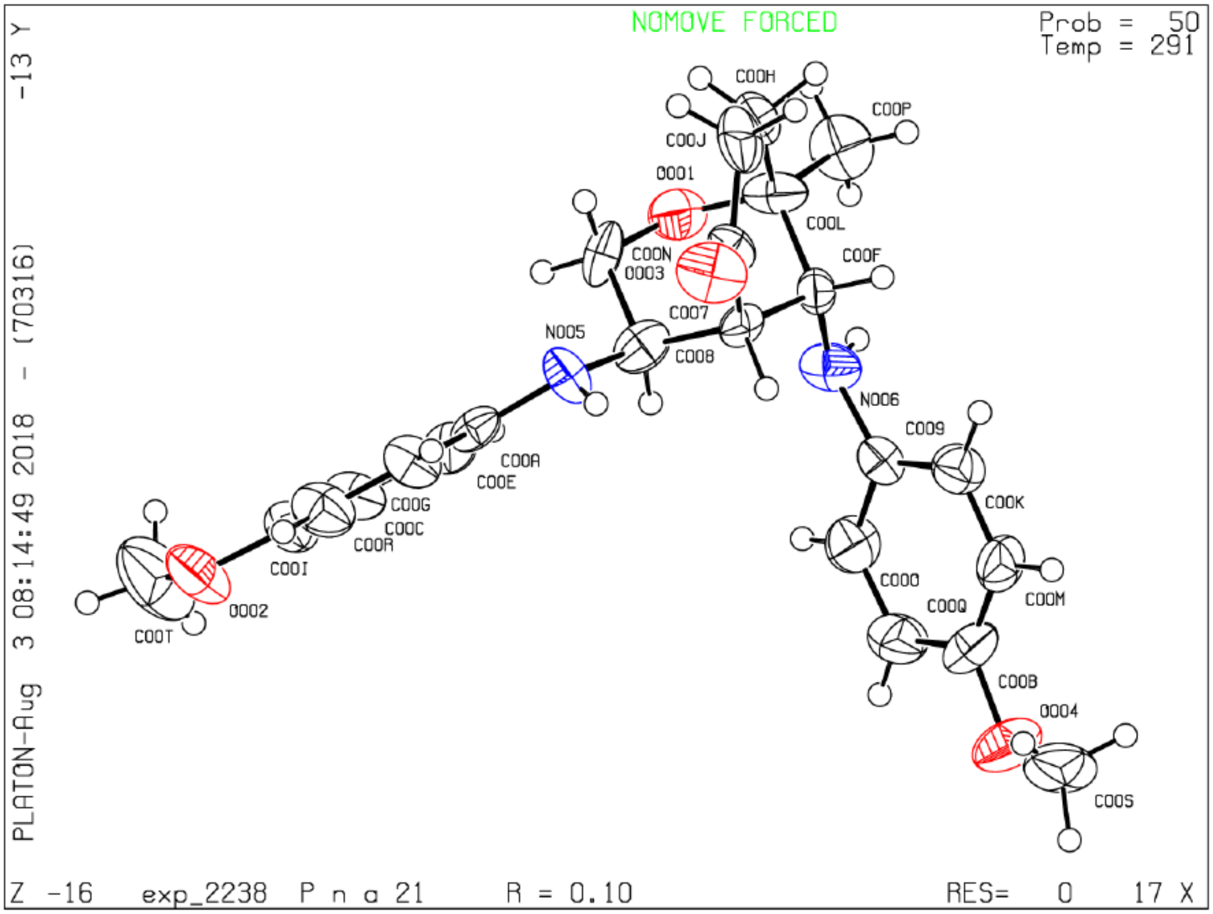

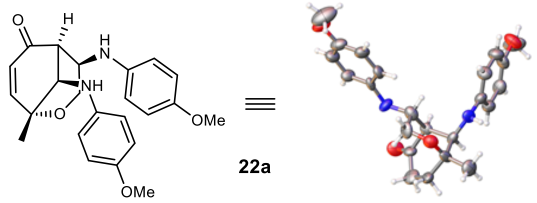

22a

Identification code	exp_2238
	SIMR-1769
Empirical formula	$C_{23}H_{28}N_2O_4$
Formula weight	396.49
Temperature	291(2) K
Wavelength	0.71073 Å
Crystal system	Orthorhombic
Space group	$Pna2_1$

Unit cell dimensions	a = 15.718(5) Å	α = 90°
	b = 19.298(6) Å	β = 90°
	c = 6.748(3) Å	γ = 90°
Volume	2047.0(12)$Å^3$	
Z	4	
Density (calculated)	1.2865 g $cm^{–3}$	
Absorption coefficient	0.088 $mm^{–1}$	
F(000)	848.4	
Crystal size	0.4 × 0.08 × 0.02 mm^3	
Theta range for data collection	6.4 to 58.3°.	
Index ranges	-19 ≤ h ≤ 21, -24 ≤ k ≤ 24, -9 ≤ l ≤ 7	
Reflections collected	7059	
Independent reflections	4142 [R(int) = 0.1449]	
Absorption correction	multi-scan	
Max. and min. transmission	0.551 and 1.000	
Refinement method	Least Squares minimization	
Data / restraints / parameters	4142/ 1 /265	
Goodness-of-fit on F2	1.002	
Final R indices [I>2sigma(I)]	R_1 = 0.0976, wR_2 = 0.1168	
R indices (all data)	R_1 = 0.4177, wR_2 = 0.2082	
Largest diff. peak and hole	1.37 and –1.45 e.$Å^{-3}$	

Supplementary Table 11 X-ray crystal structure and crystallographic data for compound **22a**

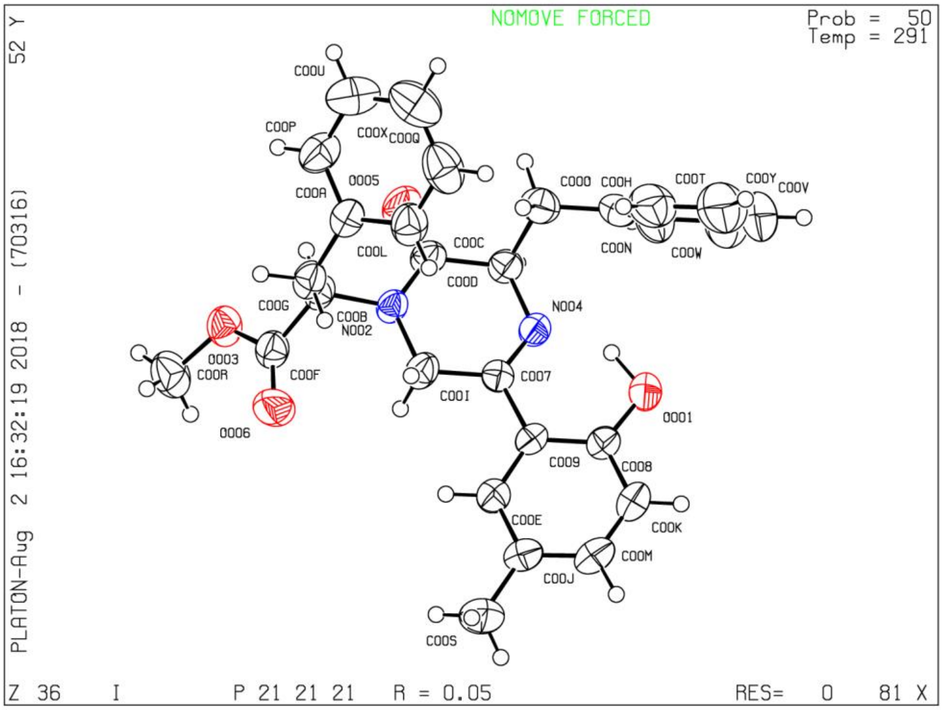

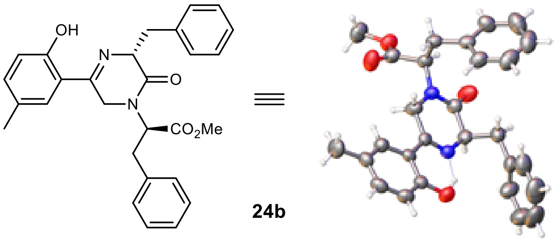

24b

Identification code	exp_2236	
	SIMR-1774	
Empirical formula	$C_{28}H_{28}N_2O_4$	
Formula weight	456.55	
Temperature	291(2) K	
Wavelength	0.71073 Å	
Crystal system	Orthorhombic	
Space group	$P2_12_12_1$	
Unit cell dimensions	a = 5.9593(8) Å	α = 90°
	b = 17.479(2) Å	β = 90°

	c = 23.174(3) Å γ = 90°
Volume	2413.8(5)Å^3
Z	4
Density (calculated)	1.2562 g cm^{-3}
Absorption coefficient	0.084 mm^{-1}
F(000)	968.5
Crystal size	0.6 × 0.04 × 0.02 mm^3
Theta range for data collection	7.04 to 58.18°.
Index ranges	-8 ≤ h ≤ 7, -18 ≤ k ≤ 23, -30 ≤ l ≤ 19
Reflections collected	7968
Independent reflections	5177 [R(int) = 0.0237]
Absorption correction	multi-scan
Max. and min. transmission	0.959 and 1.000
Refinement method	Least Squares minimization
Data / restraints / parameters	5177/ 0 /348
Goodness-of-fit on F2	1.035
Final R indices [I>2sigma(I)]	R_1 = 0.0492, wR_2 = 0.0879
R indices (all data)	R_1 = 0.0924, wR_2 = 0.1037
Largest diff. peak and hole	0.21 and –0.28 e.Å^{-3}

Supplementary Table 12 X-ray crystal structure and crystallographic data for compound **24b**

Compound	ATP content (Normalized luminescence units)	MMP (Normalized fluorescence units)	ROS content (Normalized fluorescence units)	Cytotoxicity (Percentage*)	Redox potential (Normalized fluorescence units)	Cell proliferation (Normalized colorimetric absorbance)
**5a**	0.84	0.59	1.20	-0.14	1.50	0.56
**5c**	0.67	0.27	0.98	0.14	3.14	0.21
**8a**	1.47	0.70	0.54	-2.98	1.31	0.48
**8b**	1.22	0.68	1.14	-2.44	1.46	0.75
**8d**	0.83	0.57	0.87	0.90	2.10	0.45
**26c**	0.62	0.13	0.89	-0.28	3.06	0.46
**26d**	0.66	0.48	0.99	-0.17	2.13	0.71

Supplementary Table 13 Biological assays results for the tested compounds. Hepatoma cells (Hepa1.6 cell line) were incubated for 24 h (72 h in case of proliferation assay) with the indicated compounds at a final concentration of 10 µM. **ATP content** was measured using a luminescence based detection method and results were normalized to the DMSO control and oligomycin treated (ATP depleted) controls. **Mitochondrial membrane potential (MMP)** was determined using the TMRE fluorophore and results were normalized to the DMSO control and FCCP treated (MMP uncoupled) controls. **ROS content** was measured using the MitoSOX fluorescent probe and results were normalized to the DMSO control and FCCP treated (MMP uncoupled) controls. **Cytotoxicity** was determined by measuring the release of the enzyme LDH into the culture medium and was detected by a colorimetric assay. Results were expressed as percentage* compared to the maximum LDH content in the cells (*background signal of spontaneous LDH release in healthy cells was subtracted). Cellular **Redox potential** was determined using the Alamar blue (resazurin) fluorophore and were normalized to the DMSO control and oligomycin treated controls. **Cell proliferation** was assessed after a 72 h incubation period by assessing BrdU incorporation and colorimetric detection. Results were normalized to the DMSO control and FCCP treated (non-proliferating) controls. Data were normalized as: $(x-\mu_{FCCP/oligomycin})/(\mu_{DMSO}-\mu_{FCCP/oligomycin})$.

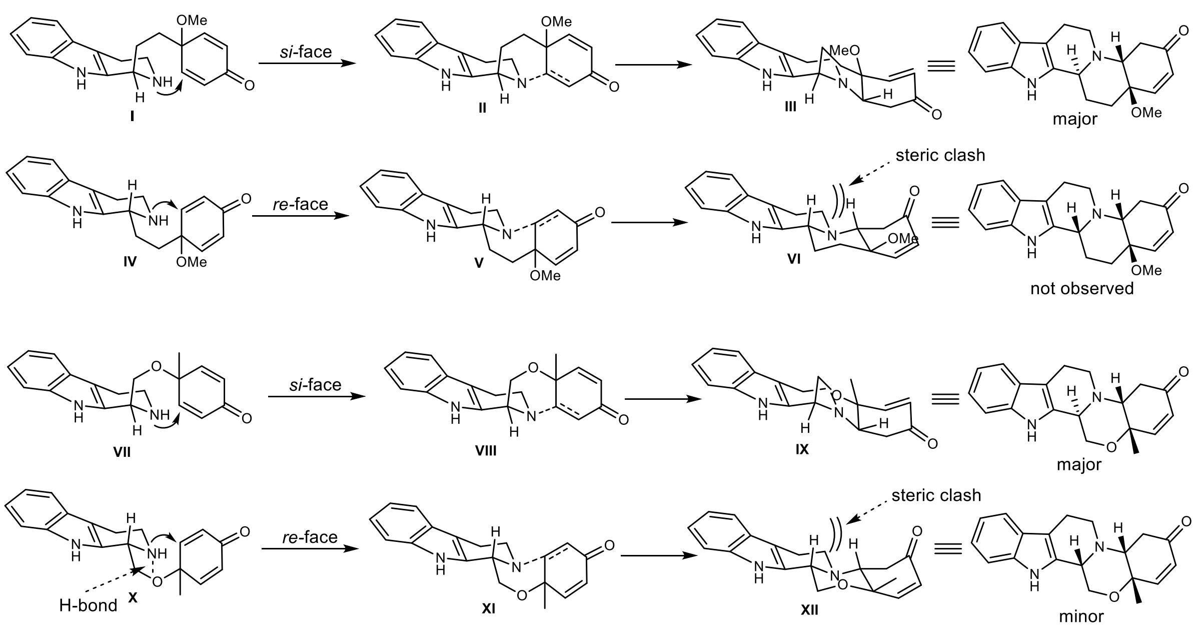

Supplementary Figure 1 Proposed stereochemical analysis of the intramolecular aza-Michael addition

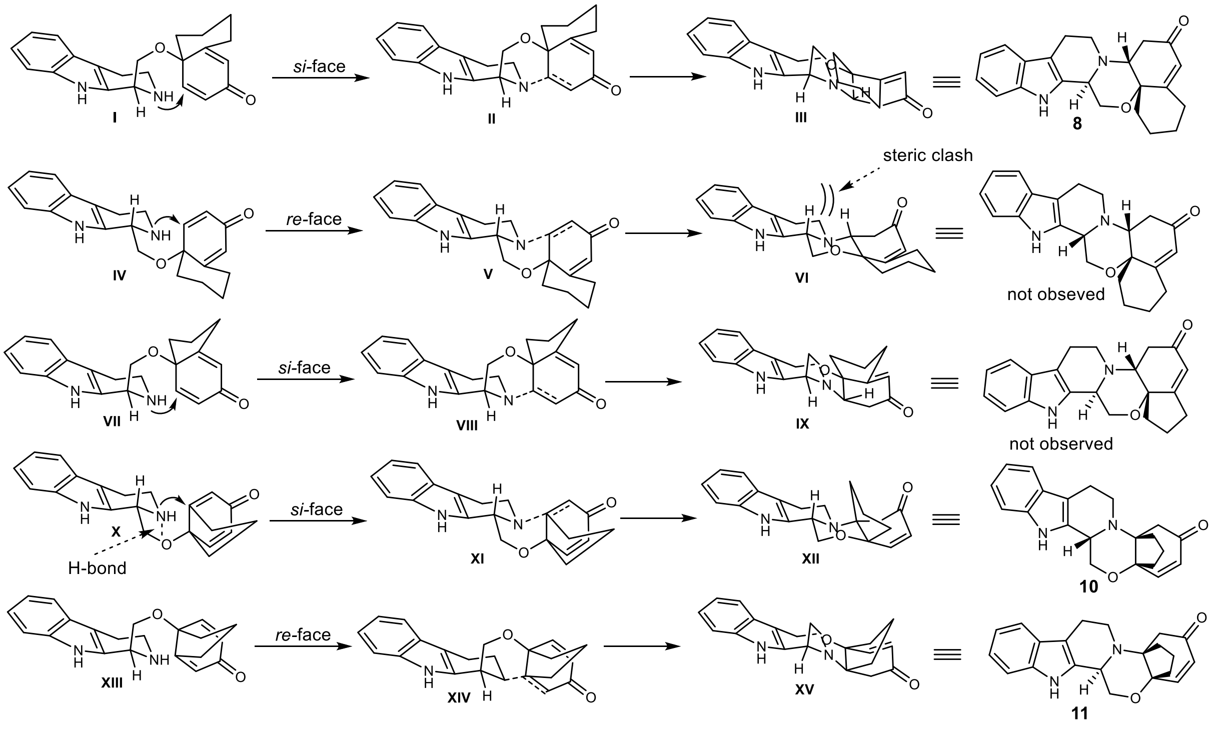

Supplementary Figure 2 Proposed stereochemical analysis of the intramolecular aza-Michael addition

Supplementary Discussion

To grow the pilot library with increased chirality, the carbonyl group of compound **5a** was reduced employing $CeCl_3.7H_2O$ and $NaBH_4$ at -20 °C to deliver the alcohol **25** as a single diastereoisomer in 86% yield (Supplementary Figure 3a). The stereochemistry of **25** was confirmed from its 1D- and 2D-NMR analysis (Supplementary Figures 169-177).

To further increase the diversity of the pilot library, representative examples of saturated cyclohexanone subunit containing products were synthesized. This was achieved by subjecting the α,β-unsaturated functionality contained in the THCs to conventional hydrogenation reaction using Pd/C. Thus the five saturated anologues **26a-26e** were synthesized in high yields (Supplementary Figure 3b).

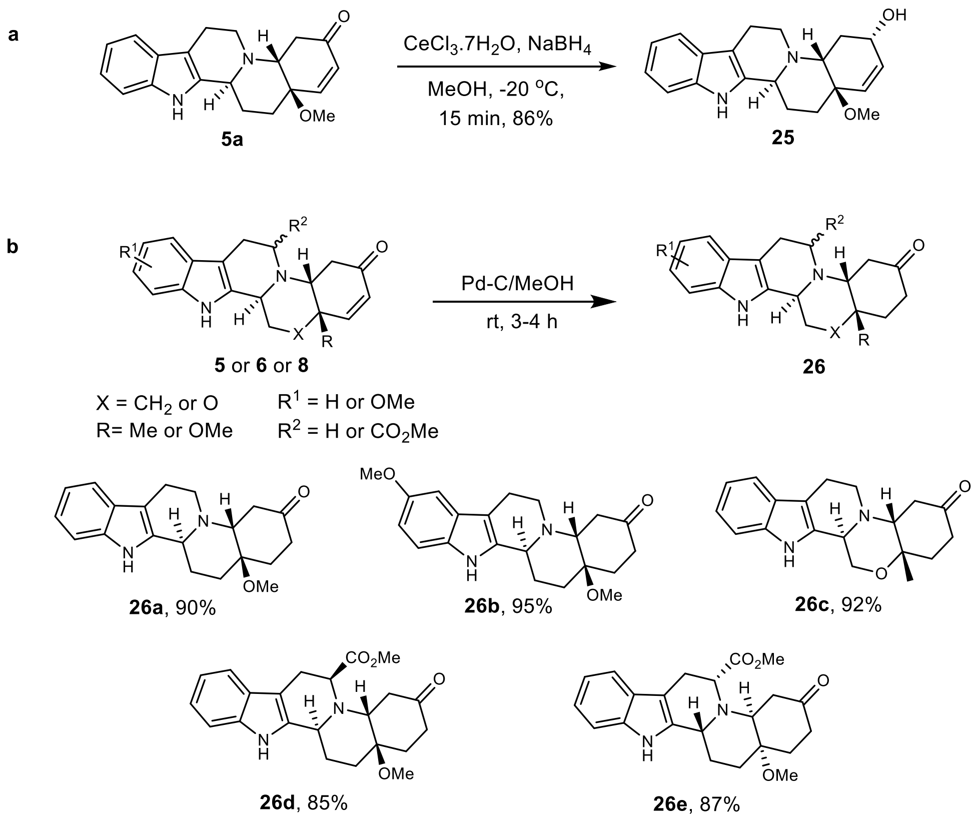

Supplementary Figure 3 Post-pairing transformations. **a** Reduction of the carbonyl group. **b** Reduction of α,β-unsaturated double bond

Supplementary Methods

Chemicals and cell culture

The tested compounds were dissolved in DMSO at a concentration of 100 mM and further diluted in PBS supplemented with 10% (v/v) DMSO. When necessary dissolution was enhanced by incubation at 50 °C and sonication at maximum output for 10 min. Hepa1.6 cell line (#CRL-1830, ATCC) was cultured in DMEM culture medium containing 1g L^{-1} glucose, 10% fetal bovine serum, 2mM glutamine, 100 U mL^{-1} Penicilin and 100 µg mL^{-1} Streptomycin and 30 µM oleic acid. Cells were seeded at a density of 20000 cells per well in 96 well plates and incubated for several hours to allow the cells to attach at 37 °C with 5% CO_2. Cells were then supplemented with the test substances at indicated concentrations (0.01-50 µM) and incubated for indicated time periods (24-48 h).

For all assays the tested substances were further diluted in 10% DMSO/PBS and added to the cells at the indicated concentrations with a final DMSO concentration of 0.01%. Negative vehicle controls were treated with 0.01% DMSO. Positive control was treated with 10 µM oligomycin or 10 µM FCCP (Carbonyl cyanide-4-(trifluoromethoxy) phenylhydrazone).

Cytotoxicity

Cytotoxicity was assessed using the Pierce LDH Cytotoxicity Assay Kit (#88953, Thermo Fisher). After cells were culture with the test substances for 24 h 20µL of the cell culture medium were transferred to a new 96 well plate and the cytotoxicity assay was performed as per the manufacturer's instructions. Absorbance was measure at 490 nm and background at 680 nm. The signal intensity of untreated cells was considered as the background signal of spontaneous LDH release and subtracted from all measurements. Cytotoxicity was expressed as the percentage of maximum signal intensity observed in freshly lysed cells.

Mitochondrial membrane potential

Mitochondrial membrane potential (ΔΨm) was assessed using the tetramethylrhodamine (TMRE) fluorophore. The cells were incubated with the test substances for 24 h and then loaded with TMRE at a final concentration of 200 nM for 45 min at 37 °C. Positive control wells were treated with 10µM FCCP 30 min before loading the cells with TMRE. The medium was removed and the cells were washed with PBS and supplemented with fresh culture medium. Fluorescence intensity of TRME was immediately measured (ex/em: 545/580 nm) using a plate

reader spectrophotometer. The results were normalized to the fluorescence intensity measured in mock treated negative controls and the positive controls treated with 10 µM FCCP.

ATP content

Cells were treated for 24 h with the test substances at the indicated concentrations. The cellular ATP content was assessed using the CellTiter-Glo Assay (#G7571, Promega) as per the manufacturer’s instructions. Luminescence was detected in a plate reader spectrophotometer. The results were normalized to the luminescence intensity measured in mock treated negative controls and the positive controls treated with 10 µM oligomycin.

Cellular Redox potential

Cells were treated for 24 h with the test substances or controls (0.1 % DMSO, 10 µM FCCP and 10 µM oligomycin). AlamarBlue reagent (resazurin, #BUF012A, Bio-Rad) was added to the cells in an amount equal to 10 % of the culture medium per well during the last 4 h of incubations. Fluorescence was measured at ex/em: 550/590 nm. Results were normalized to the fluorescence intensity measured in blank wells and wells containing fully reduced AlamarBlue reagent. Results were expressed relative to the mock treated negative controls.

BrdU incorporation/cell proliferation assay proliferation assay

Cells were incubated for a total of 48 h with the tested substances. During the last 12 h the cells were supplemented with 200 µM BrdU (#ab126556, Abcam). After the incubation period cells were washed with PBS. Following that the cells fixed and permeabilized using the reagents of the kit and labeled for 1 h with anti-BrdU antibodies conjugated with peroxidase. After washing the fixed cells 5 times with PBS the wells were incubated with peroxidase substrate TMB. The reaction was stopped after 30 min. The signal intensity was measure at absorbance 450 nm. Results were normalized to controls samples treated with vehicle (0.01% DMSO, negative control) or 10 µM oligomycin.

Lymphocyte and splenocyte preparation

Spleen and lymph nodes were collected from C57BL/6J mice and stored in RPMI medium on ice until further processing. Further procedures were performed using a laminar flow hood. Single cell suspensions were created by shearing the organs between two microscopy slides. Cell suspensions were passed through a 70 µm cell strainer to exclude remaining tissue chunks. Cells were washed in PBS and centrifuged at 300 g for 5min at 4 °C. During the preparation of splenic single cell suspension, erythrocytes were removed by incubating the cells for 5 min at rt in RBC

lysis buffer followed by extensive washing in PBS. Cells were resuspended in RPMI medium and cell count was determined. Lymphocytes were kept on ice until further use.

T cell enrichment from splenocytes

T cell was enriched from the splenocytes by negative selection using magnetic bead separation (Pan T cell isolation kit II, mouse, #130-095-130, Miltenyi) achieving more than 90% purity. The cells were kept in RPMI medium until further use.

Activation induced immune cell proliferation in T cells and lymphocytes

A total 17×10^6 cells were suspended in 15 mL Krebs Ringer buffer (with HEPES, 1g L^{-1} glucose, 1mM pyruvate), supplemented with 30 µL of Cytopainter blue stock solution (500x) (#ab176726, Abcam) and incubated for 30 min at 37 °C to let the CFSE analog accumulate in the cells. The remaining Cytopainter reagent that was not incorporated into the cells was quenched by addition of 10 mL FCS and incubated for 5 min at rt. Another 25 mL of PBS were added and cells were centrifuged at 300 g for 5 min at rt. The cells were then suspended in 12.75 mL of complete RPMI culture medium. 2×10^5 cells in a volume of 150 µL were added to the wells of 96 well plates. The cytopainter-loaded cells were supplemented with the test substances, or DMSO as a negative control and oligomycin as a positive control.

Cytopainter loaded T cells were stimulated using two different approaches. Either T cells stimulated by plate bound anti-CD3 antibodies (coated at 1µg/mL) and soluble anti-CD28 antibodies (at a final concentration of 0.2 µg mL^{-1}), or in separate replicates by addition of the cytokines IL-2 (50 ng mL^{-1}) and IL-7 (10 ng mL^{-1}) to stimulate T cells proliferation and survival.

Cytopainter loaded lymphocytes were stimulated with PMA (15 ng mL^{-1}) and Ionomycin (0.5 µg mL^{-1}). All cells were incubated for 60 h at 37 °C, 5% CO_2. Lymphocytes were then washed with 2% FBS in PBS, supplemented with anti-CD16/CD32 antibodies (to block unspecific Fc-receptor binding) and incubated for 10 min at 4 °C. Subsequently cells were incubated for 20 min at 4 °C with anti-CD3 antibodies conjugated to FITC (clone:145-2C11, #11-0031-82, eBioscience) and anti-B220 antibodies conjugated to PerCP-Cy5.5 (clone:RA3-6B2, #45-0452-82, eBioscience) to label T cells and B cells, respectively. Cells were subsequently washed with 2% FBS in PBS analyzed.

Cell proliferation was then analyzed by assessing the fluorescence intensity of cytopainter in the cells by flow cytometry. Reduced intensity of cytopainter dye indicates cells that have undergone

cell division. Data was analyzed using FlowJo v10.4.2. Proliferation parameters were derived using the FlowJo Proliferation Tool. The Expansion index determines the fold-expansion of the overall culture. The Division index expresses the average number of cell divisions that a cell in the original population has undergone and also includes the cells that have never divided.

Statistical analysis was performed using Graph Pad Prism v6.07. Comparison of the test substances was performed by ANOVA with Dunnett's post hoc test unless otherwise stated. Non-linear regression was fitted to the data points using Graph Pad.

X-Ray diffraction studies

Data collection, reduction, and cell refinement were performed using the software package CrysAlisPro[1]. Analytical absorption corrections were applied using spherical harmonics implemented in SCALE3 (ABSPACK) scaling algorithm. Using Olex2[2] program, the structures were solved with SHELXL-97[3] and/or ShelXT[4] structure solution programs, using direct method or intrinsic phasing and refined with Olex2 refine[5] refinement package using Gauss-Newton or Least Squares minimization. Anisotropic least-squares refinement of non-H atoms was applied. All crystallographic plots were obtained using the Olex2 program.

Experimental procedures and characterization data

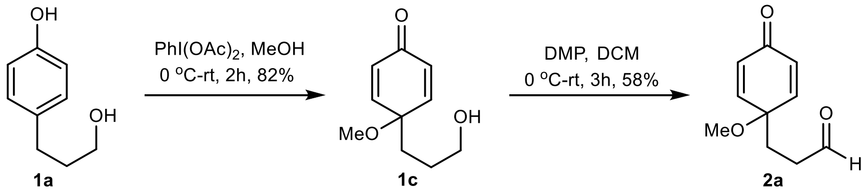

Supplementary Figure 4 Reaction scheme for the preparation of compound **2a**

Reaction procedure for the preparation of 4-(3-hydroxypropyl)-4-methoxycyclohexa-2,5-dien-1-one (1c): Phenol (**1a,** 1.0 mmol)) was dissolved in MeOH (5.0 mL) and a solution of $PhI(OAc)_2$ (1.2 mmol) in methanol (5.0 mL) was added dropwise at 0 °C over a period of 1h and stirring was continued at rt for another 1h. After completion, methanol was removed; the crude was dissolved in EtOAc (50 mL) and washed with water (2 x 50 mL). The organic layer was separated, dried over Na_2SO_4 and concentrated under vacuum. The crude product was purified on flash chromatography using 60 % EtOAc in hexane to obtain the compound **(1c)** as colorless liquid: 149 mg, 82% yield; R_f = 0.45 (silica gel, hexane/EtOAc 1:4); ^{1}H NMR (500 MHz,

DMSO-d6): δ 6.91 – 6.85 (m, 2H), 6.36 – 6.30 (m, 2H), 4.44 (t, J = 5.2 Hz, 1H), 3.35 – 3.30 (m, 2H), 3.12 (s, 3H), 1.75 – 1.67 (m, 2H), 1.37 – 1.27 (m, 2H); ^{13}C NMR (125 MHz, DMSO-d_6) δ 184.9, 151.8, 130.8, 75.3, 60.5, 52.4, 35.2, 26.7; HRMS (m/z): $[M+H]^+$ calcd. for $C_{10}H_{15}O_3$, 183.1021, found 183.1013.

Reaction procedure for the preparation of 3-(1-methoxy-4-oxocyclohexa-2,5-dien-1-yl)propanal (2a): Compound **1c** (0.5 mmol) was dissolved in DCM (5.0 mL) and added DMP (0.75 mmol) at 0 °C. The reaction mixture was slowly warmed to room temperature and stirring continued for 3h. After completion, the reaction mixture was passed through the pad of celite and collected the superintendent. This organic layer was concentrated and purified on filter column using 100% DCM to obtain the compound **2a** as colorless liquid; 53 mg, 58% yield; R_f = 0.55 (silica gel, hexane/EtOAc 1:1); ^{1}H NMR (500 MHz, $CDCl_3$): δ 9.75 (t, J = 1.2 Hz, 1H), 6.78 – 6.72 (m, 2H), 6.43 – 6.38 (m, 2H), 3.23 (s, 3H), 2.51 (td, J = 7.5, 1.2 Hz, 2H), 2.10 (t, J = 7.5 Hz, 2H); ^{13}C NMR (125 MHz, $CDCl_3$) δ 200.4, 185.0, 150.2, 131.9, 74.8, 53.3, 38.3, 31.5; HRMS (m/z): $[M+Na]^+$ calcd. for $C_{10}H_{12}O_3Na$, 203.0684, found 203.0678.

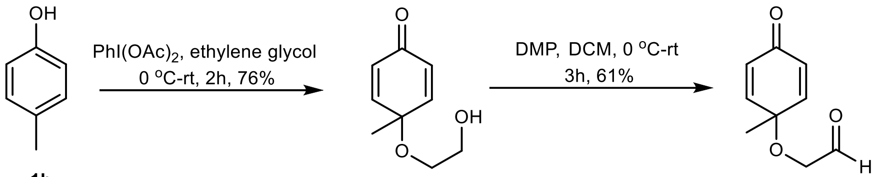

Supplementary Figure 5 Reaction scheme for the preparation of compound **2b**

Reaction procedure for the preparation of 4-(2-hydroxyethoxy)-4-methylcyclohexa-2,5-dien-1-one (1d): Phenol (**1b,** 1.0 mmol)) was dissolved in DCM (0.2 mL) and added ethylene glycol (30 mmol) at 0 °C. Next, a solution of $PhI(OAc)_2$ (1.2 mmol) in DCM (4.0 mL) was added dropwise at 0 °C over a period of 1h and stirring was continued at rt for another 1h. After completion, the crude was diluted with DCM (50 mL) and washed with water (2 x 50 mL). The organic layer was separated, dried over Na_2SO_4 and concentrated under vacuum. The crude product was purified on flash chromatography using 50 % EtOAc in hexane to obtain the compound (**1d)** as colorless liquid: 127 mg, 76% yield; R_f = 0.5 (silica gel, hexane/EtOAc 1:4); ^{1}H NMR (500 MHz, DMSO-d_6): δ 7.01 – 6.90 (m, 2H), 6.31 – 6.21 (m, 2H), 4.60 (t, J = 5.4 Hz, 1H), 3.45 (q, J = 5.1 Hz, 2H), 3.26 (t, J = 5.3 Hz, 2H), 1.37 (s, 3H); ^{13}C NMR (125 MHz,

DMSO-d_6) δ 185.1, 153.3, 129.7, 72.5, 67.4, 61.0, 26.3; HRMS (m/z): $[M+H]^+$ calcd. for $C_9H_{13}O_3$, 169.0864, found 169.0852.

Reaction procedure for the preparation of 2-((1-methyl-4-oxocyclohexa-2,5-dien-1-yl)oxy)acetaldehyde (2b): Compound **1d** (0.5 mmol) was dissolved in DCM (5.0 mL) and added DMP (0.75 mmol) at 0 °C. The reaction mixture was slowly warmed to room temperature and stirring continued for 3h. After completion, the reaction mixture was passed through the pad of celite and collected the superintendent. This organic layer was concentrated and purified on filter column using 100% DCM to obtain the compound **2b** as colorless liquid; 51 mg, 61% yield; R_f = 0.55 (silica gel, hexane/EtOAc 1:1); ^{1}H NMR (500 MHz, $CDCl_3$): δ 9.66 (s, 1H), 6.82 – 6.76 (m, 2H), 6.40 – 6.28 (m, 2H), 3.99 (d, J = 0.6 Hz, 2H), 1.57 (s, 3H); ^{13}C NMR (125 MHz, $CDCl_3$) δ 199.3, 184.8, 150.0, 131.0, 73.3, 71.3, 26.1; HRMS (m/z): $[M+H]^+$ calcd. for $C_9H_{11}O_3$, 167.0708, found 167.0697.

General reaction procedure for the preparation of compounds 5a-f: Aldehyde (**2**, 0.5 mmol) was dissolved in DCM (2 mL) and a solution of amine (**3**, 0.5 mmol) in DCM (2.0 mL) was added dropwise at -78 °C. Then, a solution of TFA (1.0 mmol) in DCM (1 mL) was added dropwise at -78 °C and slowly warmed to room temperature and stirring was continued for 2-4 h. After completion, the reaction mixture was diluted with DCM (30 mL) and washed with saturated sodium bicarbonate solution (2 x 20 mL). The organic layer was separated, dried over Na_2SO_4 and concentrated under vacuum. The crude was purified on flash chromatography, using EtOAc/hexane as an eluent to produce the title compounds **5a-f**.

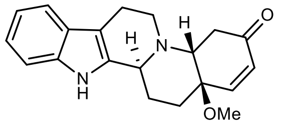

(2aR,6aR,14bS)-2a-Methoxy-2,2a,6,6a,8,9,14,14b-octahydroindolo[2',3':3,4]pyrido[1,2-a]quinolin-5(1H)-one (5a): Off-white solid, 107 mg, 67% yield; mp: 204–207 °C; R_f = 0.45 (silica gel, hexane/EtOAc 1:1); ^{1}H NMR (500 MHz, $CDCl_3$): δ 7.79 (s, 1H), 7.48 (t, J = 7.6 Hz, 1H), 7.31 (t, J = 8.4 Hz, 1H), 7.19 – 7.09 (m, 2H), 6.86 (d, J = 10.3 Hz, 1H), 6.12 (d, J = 10.3 Hz, 1H), 3.96 (d, J = 10.0 Hz, 1H), 3.83 – 3.74 (m, 1H), 3.32 (s, 3H), 3.11 – 3.05 (m, 1H), 3.03 – 2.90 (m, 2H), 2.85 – 2.73 (m, 3H), 2.09 – 1.97 (m, 2H), 1.95 – 1.83 (m, 2H); ^{13}C NMR (125 MHz, $CDCl_3$): δ 199.4, 155.5, 136.2, 134.5, 130.6, 127.3, 121.5, 119.5, 118.2, 110.7, 108.6,

74.7, 59.1, 50.7, 50.4, 49.3, 35.3, 30.3, 24.9, 22.1; HRMS (m/z): $[M+H]^+$ calcd. for $C_{20}H_{23}N_2O_2$, 323.1759; found 323.1764.

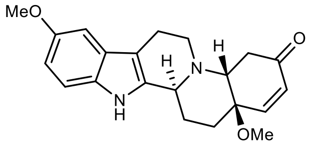

(2aR,6aR,14bS)-2a,11-Dimethoxy-2,2a,6,6a,8,9,14,14b-octahydroindolo[2',3':3,4]pyrido[1,2-a]quinolin-5(1H)-one (5b): Off-white solid, 121 mg, 69% yield; mp: 216–218 °C; R_f = 0.4 (silica gel, hexane/EtOAc 1:1); ^{1}H NMR (500 MHz, $CDCl_3$): δ 7.67 (s, 1H), 7.21 (d, *J* = 8.7 Hz, 1H), 6.94 (d, *J* = 2.0 Hz, 1H), 6.88 – 6.79 (m, 2H), 6.11 (d, *J* = 10.3 Hz, 1H), 3.92 (d, *J* = 10.7 Hz, 1H), 3.87 (s, 3H), 3.77 (t, *J* = 8.6 Hz, 1H), 3.33 (s, 3H), 3.11 – 3.04 (m, 1H), 3.02 – 2.86 (m, 2H), 2.80 (d, *J* = 8.6 Hz, 2H), 2.72 (d, *J* = 14.3 Hz, 1H), 2.10 – 1.96 (m, 2H), 1.93 – 1.80 (m, 2H); ^{13}C NMR (125 MHz, $CDCl_3$): δ 199.3, 155.5, 154.1, 135.4, 131.2, 130.6, 127.7, 111.4, 111.2, 108.5, 100.5, 74.7, 59.1, 55.9, 50.7, 50.5, 49.3, 35.3, 30.4, 24.9, 22.2; HRMS (m/z): $[M+H]^+$ calcd. for $C_{21}H_{25}N_2O_3$, 353.1865, found 353.1871.

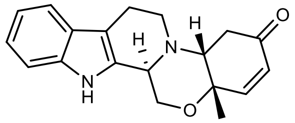

(2aS,6aR,14bR)-2a-Methyl-6,6a,8,9,14,14b-hexahydro-1H-benzo[5',6'][1,4]oxazino[4',3':1,2]pyrido[3,4-b]indol-5(2aH)-one (5c): White solid, 80 mg, 52% yield; mp: 217–219 °C; R_f = 0.45 (silica gel, hexane/EtOAc 1:1); ^{1}H NMR (500 MHz, $CDCl_3$): δ 7.95 (s, 1H), 7.52 (d, *J* = 7.7 Hz, 1H), 7.36 (d, *J* = 8.0 Hz, 1H), 7.23 – 7.17 (m, 1H), 7.17 – 7.12 (m, 1H), 6.70 (d, *J* = 10.6 Hz, 1H), 6.13 (d, *J* = 10.6 Hz, 1H), 4.27 – 4.14 (m, 2H), 4.10 – 4.01 (m, 1H), 3.32 (d, *J* = 4.4 Hz, 2H), 3.09 – 2.93 (m, 3H), 2.72 (d, *J* = 14.9 Hz, 1H), 2.64 (dd, *J* = 16.0, 3.5 Hz, 1H), 1.51 (s, 3H); ^{13}C NMR (125 MHz, $CDCl_3$): δ ^{13}C NMR (125 MHz, $CDCl_3$) δ 198.9, 151.4, 136.1, 130.8, 129.9, 127.3, 121.9, 119.7, 118.1, 111.0, 109.7, 72.2, 63.5, 59.0, 52.1, 48.0, 46.1, 23.6, 19.3; HRMS (m/z): $[M+H]^+$ calcd. for $C_{19}H_{21}N_2O_2$, 309.1603, found 309.1607.

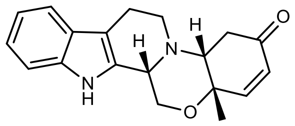

(2aS,6aR,14bS)-2a-Methyl-6,6a,8,9,14,14b-hexahydro-1H-benzo[5',6'][1,4]oxazino[4',3':1,2]pyrido[3,4-b]indol-5(2aH)-one (6c): White solid, 32 mg, 21% yield; mp: 226–228 °C; R_f = 0.5 (silica gel, hexane/EtOAc 1:1); ^{1}H NMR (500 MHz, $CDCl_3$): δ 7.66 (s, 1H), 7.48 (d, *J* = 7.8 Hz, 1H), 7.31 (d, *J* = 8.0 Hz, 1H), 7.19 – 7.13 (m, 1H), 7.11 (dd, *J* = 11.0, 3.9 Hz, 1H), 6.64 (dd, *J* = 10.1, 2.1 Hz, 1H), 6.06 (d, *J* = 10.1 Hz, 1H), 4.09 (d, *J* = 7.9 Hz, 1H), 3.81 – 3.71 (m, 2H), 3.54 (dd, *J* = 11.3, 4.5 Hz, 1H), 3.11 – 3.02 (m, 2H), 2.89 – 2.79 (m, 1H), 2.79 – 2.67 (m, 2H), 2.34 (td, *J* = 11.3, 3.9 Hz, 1H), 1.58 (s, 3H); ^{13}C NMR (125 MHz, $CDCl_3$): δ 196.7, 153.2, 136.2, 130.4, 130.2, 127.0, 121.9, 119.7, 118.3, 110.8, 110.1, 74.7, 66.3, 66.1, 58.4, 46.1, 40.1, 25.6, 22.0; HRMS (m/z): $[M+H]^+$ calcd. for $C_{19}H_{21}N_2O_2$, 309.1603, found 309.1611.

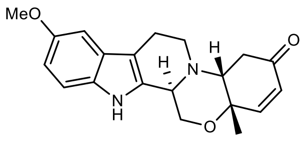

(2aS,6aR,14bR)-11-methoxy-2a-methyl-6,6a,8,9,14,14b-hexahydro-1H-benzo[5',6'][1,4]oxazino[4',3':1,2]pyrido[3,4-b]indol-5(2aH)-one (5d): Off-white solid, 89 mg, 53% yield; mp: 230–232 °C; R_f = 0.4 (silica gel, hexane/EtOAc 1:1); ^{1}H NMR (500 MHz, $CDCl_3$): δ 7.73 (s, 1H), 7.24 (d, *J* = 8.7 Hz, 1H), 6.97 (d, *J* = 1.7 Hz, 1H), 6.85 (dd, *J* = 8.7, 2.1 Hz, 1H), 6.70 (d, *J* = 10.2 Hz, 1H), 6.12 (d, *J* = 10.2 Hz, 1H), 4.22 – 4.09 (m, 2H), 4.00 (dd, *J* = 11.4, 6.0 Hz, 1H), 3.88 (s, 3H), 3.29 (d, *J* = 4.8 Hz, 2H), 3.09 – 2.90 (m, 3H), 2.70 – 2.59 (m, 2H), 1.52 (s, 3H); ^{13}C NMR (125 MHz, CD_3OD): δ 199.9, 153.6, 152.6, 131.9, 131.7, 129.3, 127.3, 111.6, 110.8, 107.9, 99.9, 72.0, 63.1, 58.8, 55.5, 53.3, 52.4, 35.6, 23.1, 18.9; HRMS (m/z): $[M+H]^+$ calcd. for $C_{20}H_{23}N_2O_3$, 339.1708, found 339.1719.

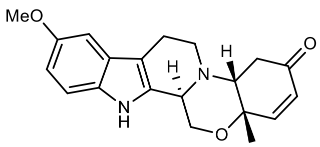

(2aS,6aR,14bS)-11-Methoxy-2a-methyl-6,6a,8,9,14,14b-hexahydro-1H-benzo[5',6'][1,4]oxazino[4',3':1,2]pyrido[3,4-b]indol-5(2aH)-one (6d): Off-white solid, 34 mg, 20% yield; mp: 243–246 °C; R_f = 0.4 (silica gel, hexane/EtOAc 1:1); ^{1}H NMR (500 MHz, $CDCl_3$): δ 7.54 (s, 1H), 7.20 (d, *J* = 8.7 Hz, 1H), 6.92 (d, *J* = 1.9 Hz, 1H), 6.81 (dd, *J* = 8.7, 2.4 Hz, 1H), 6.64 (d, *J* = 9.8 Hz, 1H), 6.05 (d, *J* = 10.3 Hz, 1H), 4.07 (d, *J* = 6.9 Hz, 1H), 3.85 (s, 3H), 3.75 (d, *J* = 5.9 Hz, 2H), 3.53 (dd, *J* = 11.2, 5.2 Hz, 1H), 3.05 (d, *J* = 13.5 Hz, 2H), 2.86 – 2.74 (m, 3H), 2.38 – 2.30 (m, 1H), 1.57 (s, 3H); ^{13}C NMR (125 MHz, $CDCl_3$): δ 196.7, 154.2, 153.2, 131.3, 130.4, 130.3, 127.5, 111.8, 111.5, 110.0, 100.4, 74.7, 66.3, 66.0, 58.5, 55.9, 46.2, 40.1, 25.6, 22.0; HRMS (m/z): $[M+H]^+$ calcd. for $C_{20}H_{23}N_2O_3$, 339.1708, found 339.1715.

General reaction procedure for the preparation of compounds 8a-e: Aldehyde (**2**, 0.5 mmol) was dissolved in DCM (2 mL) and a solution of amine (**5**, 0.5 mmol) in DCM (2.0 mL) was added dropwise at -78 °C. Then, a solution of TFA (1.0 mmol) in DCM (1 mL) was added dropwise at -78 °C and slowly warmed to room temperature and stirring was continued for 6 h. After completion, the reaction mixture was diluted with DCM (30 mL) and washed with saturated sodium bicarbonate solution (2 x 20 mL). The organic layer was separated, dried over Na_2SO_4 and concentrated under vacuum. The crude was purified on flash chromatography, using EtOAc/hexane as an eluent to deliver the title compounds **8a-e**.

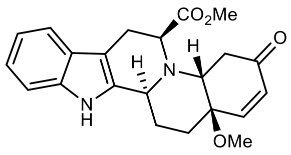

Methyl (2aR,6aR,8S,14bS)-2a-methoxy-5-oxo-1,2,2a,5,6,6a,8,9,14,14b-decahydroindolo[2',3':3,4]pyrido[1,2-a]quinoline-8-carboxylate (8a): Pinkish solid, 120 mg, 63 % yield; mp: 159–161 °C; R_f = 0.4 (silica gel, hexane/EtOAc 1:2); $[\alpha]_D$ = -275.0 (0.0047 M in acetone); ^{1}H NMR (500 MHz, $CDCl_3$): δ 7.86 (s, 1H), 7.44 (d, *J* = 7.7 Hz, 1H), 7.33 (d, *J* = 8.0 Hz, 1H), 7.21 – 7.08 (m, 2H), 6.85 (d, *J* = 10.3 Hz, 1H), 6.10 (d, *J* = 10.3 Hz, 1H), 4.01 (d, *J* = 11.3 Hz, 1H), 3.93 (dd, *J* = 10.6, 7.2 Hz, 1H), 3.83 (s, 3H), 3.75 (dd, *J* = 10.6, 3.9 Hz, 1H), 3.37 (s, 3H), 3.19 – 3.12 (m, 1H), 3.05 – 2.99 (m, 1H), 2.85 – 2.75 (m, 2H), 2.17 – 1.98 (m, 3H), 1.92 – 1.81 (m, 1H); ^{13}C NMR (125 MHz, $CDCl_3$): δ 198.6, 173.1, 156.6, 136.3, 133.2, 130.9, 126.9, 122.0, 119.9, 118.2, 110.9, 106.4, 74.8, 60.9, 54.8, 52.4, 51.0, 50.4, 34.0, 30.7, 27.1, 25.9; HRMS (m/z): $[M+H]^+$ calcd. for $C_{22}H_{25}N_2O_4$, 381.1814, found 381.1815.

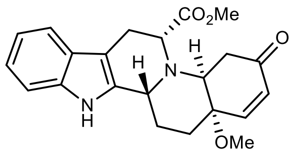

Methyl (2aS,6aS,8R,14bR)-2a-methoxy-5-oxo-1,2,2a,5,6,6a,8,9,14,14b-decahydroindolo[2',3':3,4]pyrido[1,2-a]quinoline-8-carboxylate (8b): Off-white solid, 104 mg, 55% yield; mp: 158–160 °C; R_f = 0.4 (silica gel, hexane/EtOAc 1:2); $[\alpha]_D$ = +280.7 (0.0034 M in acetone); ^{1}H NMR (500 MHz, $CDCl_3$): δ 8.09 (s, 1H), 7.43 (d, *J* = 7.8 Hz, 1H), 7.36 (d, *J* = 8.0 Hz, 1H), 7.16 (t, *J* = 7.3 Hz, 1H), 7.10 (t, *J* = 7.4 Hz, 1H), 6.83 (d, *J* = 10.3 Hz, 1H), 6.08 (d, *J* = 10.3 Hz, 1H), 3.92 (dd, *J* = 10.8, 6.2 Hz, 2H), 3.83 (s, 3H), 3.72 (dd, *J* = 10.6, 3.8 Hz, 1H), 3.35 (s, 3H), 3.19 – 3.11 (m, 1H), 3.02 (dd, *J* = 14.8, 2.6 Hz, 1H), 2.79 – 2.68 (m, 2H), 2.11 – 1.93 (m, 3H), 1.85 – 1.71 (m, 2H); ^{13}C NMR (125 MHz, $CDCl_3$): δ 198.6, 173.0, 156.6, 136.3, 133.3, 130.8, 126.9, 121.9, 119.8, 118.2, 111.0, 106.2, 74.8, 60.9, 54.8, 52.4, 51.0, 50.4, 34.0, 30.6, 27.1, 25.9; HRMS (m/z): $[M+H]^+$ calcd. for $C_{22}H_{25}N_2O_4$, 381.1814, found 381.1823.

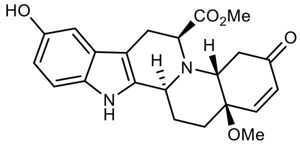

Methyl (2aR,6aR,8S,14bS)-11-hydroxy-2a-methoxy-5-oxo-1,2,2a,5,6,6a,8,9,14,14b-decahydroindolo[2',3':3,4]pyrido[1,2-a]quinoline-8-carboxylate (8c): Brownish solid, 79 mg, 40% yield; mp: 165–167 °C; R_f = 0.35 (silica gel, hexane/EtOAc 1:2); $[\alpha]_D$ = -214.2 (0.0017 M in acetone); ^{1}H NMR (500 MHz, CD_3OD): δ 7.13 (d, *J* = 8.6 Hz, 1H), 6.90 (dd, *J* = 10.4, 3.6 Hz, 1H), 6.76 (d, *J* = 2.3 Hz, 1H), 6.64 (dd, *J* = 8.6, 2.3 Hz, 1H), 6.05 (d, *J* = 10.3 Hz, 1H), 3.95 – 3.86 (m, 1H), 3.87 – 3.79 (m, 4H), 3.69 (dd, *J* = 10.5, 3.9 Hz, 1H), 3.31 (s, 3H), 3.04 – 2.95 (m, 1H), 2.95 – 2.82 (m, 2H), 2.72 – 2.63 (m, 1H), 2.13 (d, *J* = 6.7 Hz, 1H), 1.99 – 1.84 (m, 3H); ^{13}C NMR (125 MHz, CD_3OD): δ 199.5, 173.2, 156.5, 150.0, 134.4, 131.6, 129.8, 127.1, 110.9, 110.4, 103.7, 101.7, 74.9, 61.1, 55.2, 51.3, 50.4, 49.7, 33.5, 29.8, 26.4, 25.0; HRMS (m/z): $[M+H]^+$ calcd. for $C_{22}H_{25}N_2O_5$, 397.1763, found 397.1774.

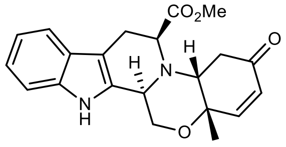

Methyl (2aS,6aR,8S,14bR)-2a-methyl-5-oxo-2a,5,6,6a,8,9,14,14b-octahydro-1H-benzo[5',6'][1,4]oxazino[4',3':1,2]pyrido[3,4-b]indole-8-carboxylate (8d): Off-white solid, 78 mg, 43% yield; mp: 150–153 °C; R_f = 0.45 (silica gel, hexane/EtOAc 1:1); $[\alpha]_D$ = -252.4 (0.0054 M in acetone); ^{1}H NMR (500 MHz, $CDCl_3$): δ 7.89 (s, 1H), 7.51 (d, *J* = 7.7 Hz, 1H), 7.36 (d, *J* = 8.0 Hz, 1H), 7.25 – 7.19 (m, 1H), 7.16 (dd, *J* = 11.0, 3.9 Hz, 1H), 6.73 (d, *J* = 10.2 Hz, 1H), 6.08 (d, *J* = 10.2 Hz, 1H), 4.36 – 4.29 (m, 1H), 4.17 (dd, *J* = 11.2, 2.8 Hz, 1H), 3.99 (dd, *J* = 11.2, 9.1 Hz, 1H), 3.86 – 3.76 (m, 4H), 3.46 – 3.38 (m, 1H), 3.22 – 3.12 (m, 1H), 3.11 – 2.94 (m, 2H), 2.58 (dd, *J* = 15.9, 3.7 Hz, 1H), 1.60 (d, *J* = 10.6 Hz, 3H); ^{13}C NMR (125 MHz, $CDCl_3$): δ 198.6, 172.8, 151.0, 136.4, 129.6, 126.8, 122.7, 120.1, 118.3, 111.2, 108.1, 76.9, 71.6, 64.0, 60.7, 56.9, 52.5, 52.0, 33.9, 25.0, 22.9; HRMS (m/z): $[M+H]^+$ calcd. for $C_{21}H_{23}N_2O_4$, 367.1657, found 367.1660.

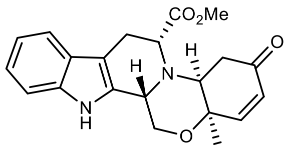

Methyl (2aR,6aS,8R,14bS)-2a-methyl-5-oxo-2a,5,6,6a,8,9,14,14b-octahydro-1H-benzo[5',6'][1,4]oxazino[4',3':1,2]pyrido[3,4-b]indole-8-carboxylate (8e): Off-white solid, 95 mg, 52% yield; mp: 150–152 °C; R_f = 0.45 (silica gel, hexane/EtOAc 1:1); $[\alpha]_D$ = +250.0 (0.0027 M in acetone); ^{1}H NMR (500 MHz, Acetone-d_6): δ 10.06 (s, 1H), 7.46 (d, *J* = 7.8 Hz, 1H), 7.32 (d, *J* = 8.0 Hz, 1H), 7.11 – 7.05 (m, 1H), 7.05 – 6.99 (m, 1H), 6.72 (d, *J* = 10.2 Hz, 1H), 5.93 (d, *J* = 10.2 Hz, 1H), 4.36 (d, *J* = 9.2 Hz, 1H), 4.27 (dd, *J* = 11.2, 3.5 Hz, 1H), 3.89 (t, *J* = 9.7 Hz, 1H), 3.83 – 3.74 (m, 4H), 3.35 (dd, *J* = 11.4, 3.8 Hz, 1H), 3.13 – 2.93 (m, 2H), 2.81 (t, *J* = 10.1 Hz, 1H), 2.53 (dd, *J* = 15.5, 3.8 Hz, 1H), 1.59 (s, 3H); ^{13}C NMR (125 MHz, Acetone-d_6): δ 198.7, 173.3, 151.4, 137.7, 137.6, 131.3, 129.6, 127.7, 122.2, 119.9, 118.6, 111.9, 107.3, 71.7, 64.4, 61.5, 57.8, 52.4, 33.7, 30.3, 22.7; HRMS (m/z): $[M+H]^+$ calcd. for $C_{21}H_{23}N_2O_4$, 367.1657, found 367.1649.

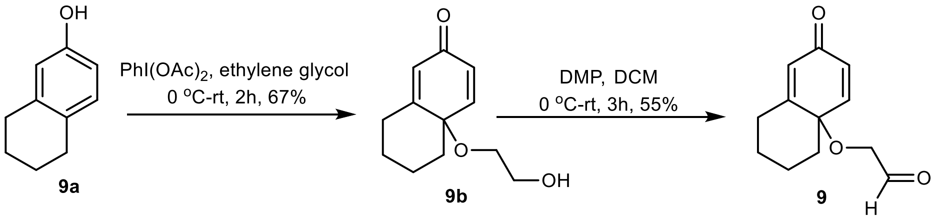

Supplementary Figure 6 Reaction scheme for the preparation of compound **9**

Reaction procedure for the preparation of 4a-(2-hydroxyethoxy)-5,6,7,8-tetrahydronaphthalen-2(4aH)-one (9b): Phenol (**9a**, 1.0 mmol)) was dissolved in DCM (0.2 mL) and added ethylene glycol (30 mmol) at 0 °C. Next, a solution of $PhI(OAc)_2$ (1.2 mmol) in DCM (4.0 mL) was added dropwise at 0 °C over a period of 1h and stirring was continued at rt for another 1h. After completion, the crude was diluted with DCM (50 mL) and washed with water (2 x 50 mL). The organic layer was separated, dried over Na_2SO_4 and concentrated under vacuum. The crude product was purified on flash chromatography using 55 % EtOAc in hexane to obtain the compound **9b** as colorless liquid: 139 mg, 67% yield; R_f = 0.5 (silica gel, hexane/EtOAc 1:4); ^{1}H NMR (500 MHz, DMSO-d_6) δ 6.84 (d, J = 10.0 Hz, 1H), 6.24 (dd, J = 10.0, 2.0 Hz, 1H), 6.17 (t, J = 1.7 Hz, 1H), 4.62 (t, J = 5.7 Hz, 1H), 3.49 – 3.44 (m, 2H), 3.17 – 3.12 (m, 1H), 3.05 – 3.00 (m, 1H), 2.48 – 2.40 (m, 1H), 2.36 – 2.28 (m, 1H), 2.15 – 2.09 (m, 1H), 1.98 – 1.84 (m, 2H), 1.56 – 1.50 (m, 1H), 1.33 – 1.22 (m, 2H); ^{13}C NMR (125 MHz, DMSO-d_6) δ 185.3, 163.7, 152.1, 129.8, 125.5, 72.9, 65.7, 60.3, 38.6, 31.6, 27.6, 19.9; HRMS (m/z): $[M+H]^+$ calcd. for $C_{12}H_{16}O_3$, 209.1177, found 209.1167.

Reaction procedure for the preparation of 2-((7-oxo-1,3,4,7-tetrahydronaphthalen-4a(2H)-yl)oxy)acetaldehyde (9): Compound **9b** (0.5 mmol) was dissolved in DCM (5.0 mL) and added DMP (0.75 mmol) at 0 °C. The reaction mixture was slowly warmed to room temperature and stirring continued for 3h. After completion, the reaction mixture was passed through the pad of celite and collected the superintendent. This organic layer was concentrated and purified on filter column using 100% DCM to obtain the compound **9** as colorless liquid; 57 mg, 55% yield; R_f = 0.55 (silica gel, hexane/EtOAc 1:1); ^{1}H NMR (500 MHz, $CDCl_3$) δ 9.68 (s, 1H), 6.71 (d, J = 10.0 Hz, 1H), 6.34 (dd, J = 10.0, 1.9 Hz, 1H), 6.25 (d, J = 1.4 Hz, 1H), 3.87 – 3.76 (m, 2H), 2.45 – 2.27 (m, 3H), 2.10 – 1.99 (m, 2H), 1.73 – 1.66 (m, 1H), 1.49 – 1.37 (m, 2H); ^{13}C NMR (125 MHz, $CDCl_3$) δ 199.2, 185.8, 161.4, 149.6, 131.3, 127.2, 74.3, 70.1, 39.1, 32.6, 28.0, 20.4; HRMS (m/z): $[M+H]^+$ calcd. for $C_{12}H_{15}O_3$, 207.1021, found 207.1012.

Reaction procedure for the preparation of (2aS,9aR,17bR)-3,4,5,6,9,9a,11,12,17,17b-Decahydro-1H,8H-naphtho[1'',8a'':5',6'][1,4]oxazino[4',3':1,2]pyrido[3,4-b]indol-8-one

(10): Aldehyde (**9**, 0.5 mmol) was dissolved in DCM (2 mL) and a solution of amine (**3a**, 0.5 mmol) in DCM (2.0 mL) was added dropwise at -78 °C. Then, a solution of TFA (1.0 mmol) in DCM (1 mL) was added dropwise at -78 °C and slowly warmed to room temperature and stirring was continued for 12 h. After completion, the reaction mixture was diluted with DCM (30 mL) and washed with saturated sodium bicarbonate solution (2 x 20 mL). The organic layer was separated, dried over Na_2SO_4 and concentrated under vacuum. The crude was purified on flash chromatography, using 42 %EtOAc in hexane as an eluent to produce the title compound **10**.

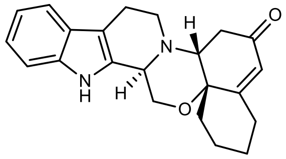

White solid, 88 mg, 55% yield; mp: 232–235 °C; R_f = 0.45 (silica gel, hexane/EtOAc 1:1); ^{1}H NMR (500 MHz, $CDCl_3$): δ 7.83 (s, 1H), 7.52 (d, *J* = 7.8 Hz, 1H), 7.34 (d, *J* = 7.8 Hz, 1H), 7.22 – 7.09 (m, 2H), 5.91 (s, 1H), 4.16 (d, *J* = 8.4 Hz, 1H), 4.05 (dd, *J* = 10.6, 2.8 Hz, 1H), 3.84 (t, *J*= 10.6 Hz, 1H), 3.21 – 3.12 (m, 2H), 3.12 – 3.02 (m, 1H), 3.11 – 2.92 (m, 3H), 2.80 – 2.70 (m, 1H), 2.68 – 2.51 (m, 2H), 2.36 (d, *J* = 13.9 Hz, 1H), 1.94 (d, *J* = 12.2 Hz, 1H), 1.73 – 1.57 (m, 2H), 1.53 – 1.39 (m, 1H), 1.21 (t, *J* = 12.9 Hz, 1H); ^{13}C NMR (125 MHz, $CDCl_3$): δ 199.6, 162.5, 136.3, 130.7, 127.1, 125.2, 121.9, 119.7, 118.2, 110.9, 109.8, 72.5, 63.2, 60.6, 50.6, 48.9, 32.6, 32.2, 32.1, 26.9, 21.4, 20.2; HRMS (m/z): $[M+H]^+$ calcd. for $C_{22}H_{25}N_2O_2$, 349.1916, found 349.1910.

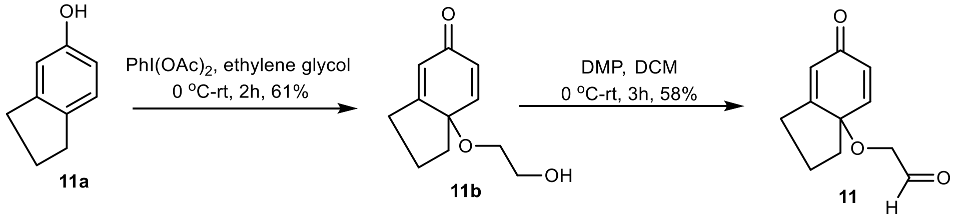

Supplementary Figure 7 Reaction scheme for the preparation of compound **11**

Reaction procedure for the preparation of 7a-(2-hydroxyethoxy)-1,2,3,7a-tetrahydro-5H-inden-5-one (11b): Phenol (**11a**, 1.0 mmol)) was dissolved in DCM (0.2 mL) and added ethylene glycol (30 mmol) at 0 °C. Next, a solution of $PhI(OAc)_2$ (1.2 mmol) in DCM (4.0 mL)

was added dropwise at 0 °C over a period of 1h and stirring was continued at rt for another 1h. After completion, the crude was diluted with DCM (50 mL) and washed with water (2 x 50 mL). The organic layer was separated, dried over Na_2SO_4 and concentrated under vacuum. The crude product was purified on flash chromatography using 57 % EtOAc in hexane to obtain the compound **11b** as colorless liquid: 119 mg, 61% yield; R_f = 0.5 (silica gel, hexane/EtOAc 1:4); ^{1}H NMR (500 MHz, DMSO-d_6) δ 6.94 (d, *J* = 10.0 Hz, 1H), 6.23 (dd, *J* = 10.0, 1.8 Hz, 1H), 6.16 (dd, *J* = 3.3, 2.0 Hz, 1H), 4.56 (t, *J* = 5.6 Hz, 1H), 3.46 – 3.38 (m, 2H), 3.19 – 3.13 (m, 1H), 3.08 – 3.02 (m, 1H), 2.73 – 2.62 (m, 1H), 2.49 – 2.39 (m, 1H), 2.16 – 1.99 (m, 2H), 1.91 – 1.80 (m, 1H), 1.60 – 1.52 (m, 1H); ^{13}C NMR (125 MHz, DMSO-d_6) δ 185.5, 167.2, 146.1, 130.7, 124.2, 77.7, 65.3, 60.2, 34.8, 28.0, 21.3; HRMS (m/z): $[M+H]^+$ calcd. for $C_{11}H_{15}O_3$, 195.1021, found 195.1015.

Reaction procedure for the preparation of 2-((6-oxo-1,2,3,6-tetrahydro-3aH-inden-3a-yl)oxy)acetaldehyde (11): Compound **11b** (0.5 mmol) was dissolved in DCM (5.0 mL) and added DMP (0.75 mmol) at 0 °C. The reaction mixture was slowly warmed to room temperature and stirring continued for 3h. After completion, the reaction mixture was passed through the pad of celite and collected the superintendent. This organic layer was concentrated and purified on filter column using 100% DCM to obtain the compound **11** as colorless liquid; 56 mg, 58% yield; R_f = 0.55 (silica gel, hexane/EtOAc 1:1); ^{1}H NMR (500 MHz, $CDCl_3$) δ 9.62 (s, 1H), 6.83 (d, *J* = 9.9 Hz, 1H), 6.32 (dd, *J* = 9.9, 1.7 Hz, 1H), 6.22 (s, 1H), 3.85 (q, *J* = 17.9, 0.6 Hz, 2H), 2.80 – 2.66 (m, 1H), 2.57 – 2.45 (m, 1H), 2.38 – 2.22 (m, 2H), 2.07 – 1.92 (m, 1H), 1.82 – 1.68 (m, 1H); ^{13}C NMR (125 MHz, $CDCl_3$) δ 199.3, 185.9, 165.4, 143.9, 132.3, 125.8, 79.4, 69.8, 35.5, 28.8, 21.7; HRMS (m/z): $[M+H]^+$ calcd. for $C_{11}H_{13}O_3$, 193.0864, found 193.0854.

Reaction procedure for the preparation of compounds 12 and 13: Aldehyde (**11**, 0.5 mmol) was dissolved in DCM (2 mL) and a solution of amine (**3a**, 0.5 mmol) in DCM (2.0 mL) was added dropwise at -78 °C. Then, a solution of TFA (1.0 mmol) in DCM (1 mL) was added dropwise at -78 °C and slowly warmed to room temperature and stirring was continued for 12 h. After completion, the reaction mixture was diluted with DCM (30 mL) and washed with saturated sodium bicarbonate solution (2 x 20 mL). The organic layer was separated, dried over Na_2SO_4 and concentrated under vacuum. The crude was purified on flash chromatography, using 45 % EtOAc in hexane as an eluent to deliver the title compounds **12** and **13**.

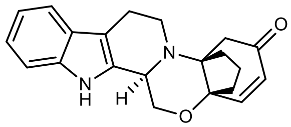

(2aS,6aR,14bR)-8,9,14,14b-Tetrahydro-1H-2a,6a-propanobenzo[5',6'][1,4]oxazino[4',3':1,2]pyrido[3,4-b]indol-5(6H)-one (12): Off-white solid, 50 mg, 30% yield; mp: 126–228 °C; R_f = 0.5 (silica gel, hexane/EtOAc 1:1); ^{1}H NMR (500 MHz, $CDCl_3$): δ 7.84 (s, 1H), 7.52 (d, *J* = 7.5 Hz, 1H), 7.34 (d, *J* = 7.5 Hz, 1H), 7.16 (dt, *J* = 26.8, 7.2 Hz, 2H), 6.62 (t, *J* = 12.2 Hz, 1H), 6.08 (d, *J* = 10.1 Hz, 1H), 4.17 – 4.06 (m, 2H), 3.93 (t, *J* = 10.6 Hz, 1H), 3.32 (d, *J* = 7.3 Hz, 1H), 3.03 (d, *J* = 15.6 Hz, 1H), 2.91 – 2.80 (m, 2H), 2.61 – 2.45 (d, *J* = 14.3 Hz, 1H), 2.62 – 2.43 (m, 2H), 2.22 (t, *J* = 9.7 Hz, 1H), 1.98 – 1.87 (m, 1H), 1.64 – 1.44 (m, 3H); ^{13}C NMR (125 MHz, $CDCl_3$) δ: 200.1, 150.4, 136.3, 130.9, 128.9, 127.0, 121.9, 119.7, 118.2, 110.9, 110.4, 80.3, 63.5, 62.5, 51.8, 43.1, 35.8, 33.8, 30.4, 22.6, 19.0; HRMS (m/z): $[M+H]^+$ calcd. for $C_{21}H_{23}N_2O_2$, 335.1759, found 335.1754.

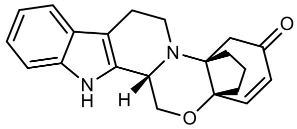

(2aS,6aR,14bS)-8,9,14,14b-Tetrahydro-1H-2a,6a-propanobenzo[5',6'][1,4]oxazino[4',3':1,2]pyrido[3,4-b]indol-5(6H)-one (13): Off-white solid, 50 mg, 30% yield; mp: 200–202 °C; R_f = 0.55 (silica gel, hexane/EtOAc 1:1); ^{1}H NMR (500 MHz, $CDCl_3$): δ 7.72 (s, 1H), 7.53 – 7.41 (m, 1H), 7.36 – 7.25 (m, 1H), 7.19 – 7.06 (m, 2H), 6.67 (d, *J* = 10.2 Hz, 1H), 6.08 (d, *J* = 10.2 Hz, 1H), 4.15 (d, *J* = 9.7 Hz, 1H), 4.06 (dd, *J* = 10.8, 3.0 Hz, 1H), 3.69 (t, *J* = 10.8 Hz, 1H), 3.47 (dd, *J* = 11.3, 4.8 Hz, 1H), 2.94 (d, *J* = 16.0 Hz, 1H), 2.85 – 2.74 (m, 1H), 2.67 (d, *J* = 14.9 Hz, 1H), 2.60 – 2.47 (m, 2H), 2.41 (td, *J* = 11.5, 3.4 Hz, 1H), 2.24 – 1.99 (m, 3H), 1.97 – 1.85 (m, 1H), 1.48 – 1.35 (m, 1H); ^{13}C NMR (125 MHz, $CDCl_3$): δ 197.8, 150.5, 136.2, 130.9, 130.8, 127.1, 121.7, 119.7, 118.2, 110.8, 110.2, 83.2, 71.5, 65.7, 51.9, 45.8, 43.5, 36.6, 25.7, 22.6, 20.5; HRMS (m/z): $[M+H]^+$ calcd. for $C_{21}H_{23}N_2O_2$, 335.1759, found 335.1755.

Reaction procedure for the preparation of (2aR,3S,6aR,14bS)-2a-methyl-3,4,6,6a,9,14-hexahydro-1H,8H-3,14b-epoxybenzo[5',6'][1,4]oxazino[4',3':1,2]pyrido[3,4-b]indole-1,5(2aH)-dione (14): Compound **5c/6c** (0.1 mmol) was dissolved in degassed acetone (0.3 mL) and a

solution of NMO (0.2 mmol) in degassed water (0.2 mL) was added at rt. Then, OsO_4 (10 mol%, 2.5 wt% in tert-Butanol) solution was added dropwise and stirring was continued for 14 h. After completion, the reaction mixture was quenched with saturated solution of $Na_2S_2O_3.5H_2O$ and stirring was continued for another 30 min. Then, the reaction mixture was passed through a celite pad and the filtrate was concentrated. Then, the crude was dissolved with DCM (30 mL) and washed with water (2 x 20 mL). The organic layer was separated, dried over Na_2SO_4 and concentrated under vacuum. The crude was purified on flash chromatography, using 55% EtOAc in hexane as an eluent to produce the title compound **14**.

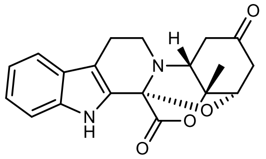

Off-white solid, 25 mg, 76% yield; mp: 163–166 °C; R_f = 0.45 (silica gel, hexane/EtOAc 1:4); ^{1}H NMR (500 MHz, CD_3OD): δ 7.50 (d, J = 7.9 Hz, 1H), 7.38 (d, J = 8.2 Hz, 1H), 7.21 – 7.15 (m, 1H), 7.10 – 7.02 (m, 1H), 4.55 – 4.51 (m, 1H), 3.33 – 3.31 (m, 1H), 3.25 – 3.18 (m, 1H), 3.01 – 2.61 (m, 8H), 1.79 (s, 3H); ^{13}C NMR (125 MHz, CD_3OD): δ 205.7, 167.5, 137.2, 126.1, 125.6, 122.2, 118.8, 118.4, 111.5, 111.4, 80.8, 79.0, 76.3, 62.2, 47.2, 44.2, 43.1, 21.2, 18.9; HRMS (m/z): $[M+H]^+$ calcd. for $C_{19}H_{19}N_2O_4$, 339.1344, found 339.1346.

Reaction procedure for the preparation of (2aR,6aS,9aR,17bS)-3,4,5,6,9,9a,12,17-Octahydro-1H,11H-6a,17b-epoxynaphtho[1'',8a'':5',6'][1,4]oxazino[4',3':1,2]pyrido[3,4-b]indole-1,8(7H)-dione (15): Compound **10** (0.1 mmol) was dissolved in degassed acetone (0.3 mL) and a solution of NMO (0.2 mmol) in degassed water (0.2 mL) was added at rt. Then, OSO_4 (10 mol%, 2.5 wt% in tert-Butanol) solution was added dropwise and stirring was continued for 17 h. After completion, the reaction mixture was quenched with saturated solution of $Na_2S_2O_3.5H_2O$ and stirring was continued for 30 min. Then, the reaction mixture was passed through a celite pad and the filtrate was concentrated. Then, the crude was dissolved with DCM (30 mL) and washed with water (2 x 20 mL). The organic layer was separated, dried over Na_2SO_4 and concentrated under vacuum. The crude was purified on flash chromatography, using 52% EtOAc in hexane as an eluent to produce the title compound **15**.

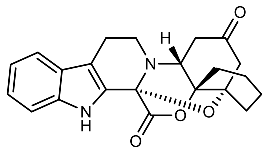

Off-white solid, 24 mg, 65% yield; mp: 185–187 °C; R_f = 0.55 (silica gel, hexane/EtOAc 1:3); ^{1}H NMR (500 MHz, Acetone-d_6): δ 7.49 (d, *J* = 7.9 Hz, 1H), 7.41 (d, *J* = 8.2 Hz, 1H), 7.19 – 7.11 (m, 1H), 7.07 – 6.99 (m, 1H), 3.31 c 3.21 (m, 2H), 3.07 (d, *J* = 16.9 Hz, 1H), 3.01 – 2.70 (m, 6H), 2.36 (dd, *J* = 17.0, 2.2 Hz, 1H), 2.33 – 2.24 (m, 1H), 1.89 – 1.72 (m, 4H), 1.71 – 1.64 (m, 1H), 1.59 – 1.47 (m, 1H); ^{13}C NMR (125 MHz, Acetone-d_6): δ 205.1, 167.4, 138.1, 138.0, 127.8, 127.6, 126.8, 123.1, 119.8, 119.4, 112.5, 112.3, 80.7, 79.1, 63.2, 48.4, 48.1, 44.3, 34.5, 22.5, 22.3, 20.7; HRMS (m/z): $[M+H]^+$ calcd. for $C_{22}H_{23}N_2O_4$, 379.1657, found 379.1666.

Reaction procedure for the preparation of methyl (2aR,3R,4S,6aR,8S,14bR)-3,4-dihydroxy-2a-methyl-5-oxo-2a,3,4,5,6,6a,8,9,14,14b-decahydro-1H-benzo[5',6'][1,4]oxazino[4',3':1,2]pyrido[3,4-b]indole-8-carboxylate (16): Compound **8d** (0.1 mmol) was dissolved in degassed acetone (0.3 mL) and a solution of NMO (0.2 mmol) in degassed water (0.2 mL) was added at rt. Then, OSO_4 (10 mol%, 2.5 wt% in tert-Butanol) solution was added dropwise and stirring was continued for 16 h. After completion, the reaction mixture was quenched with saturated solution of $Na_2S_2O_3.5H_2O$ and stirring was continued for another 30 min. Then, the reaction mixture was passed through a celite pad and the filtrate was concentrated. Then, the crude was dissolved with DCM (30 mL) and washed with water (2 x 20 mL). The organic layer was separated, dried over Na_2SO_4 and concentrated under vacuum. The crude was purified on flash chromatography, using EtOAc in hexane as an eluent to produce the title compound **16**.

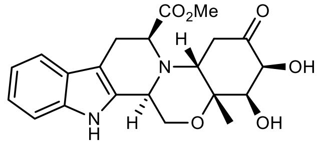

White solid, 20 mg, 52% yield; mp: 228–230 °C; R_f = 0.55 (silica gel, hexane/EtOAc 1:4); $[\alpha]_D$ = -100.0 (0.001 M in acetone); ^{1}H NMR (500 MHz, $CDCl_3$): δ 7.73 (s, 1H), 7.48 (d, *J* = 7.5 Hz, 1H), 7.36 (d, *J* = 7.9 Hz, 1H), 7.25 – 7.18 (m, 1H), 7.18 – 7.12 (m, 1H), 4.76 – 4.66 (m, 1H), 4.38 (d, *J* = 8.5 Hz, 1H), 4.18 – 4.11 (m, 1H), 4.09 – 3.98 (m, 2H), 3.84 (s, 3H), 3.72 – 3.62 (m,

2H), 3.48 – 3.39 (m, 1H), 3.25 – 3.12 (m, 2H), 3.07 – 2.96 (m, 1H), 2.69 (dd, *J* = 13.7, 5.8 Hz, 1H), 2.51 (s, 1H), 1.70 (s, 3H); ^{13}C NMR (125 MHz, $CDCl_3$): δ 208.7, 172.2, 136.3, 129.1, 126.5, 122.3, 120.1, 118.2, 111.0, 107.5, 77.5, 76.5, 74.3, 64.2, 60.6, 57.5, 52.4, 49.6, 32.0, 26.8, 18.5; HRMS (m/z): $[M+H]^+$ calcd. for $C_{21}H_{25}N_2O_6$, 401.1712, found 401.1711.

Reaction procedure for the preparation of methyl (2aS,3S,4R,6aS,8R,14bS)-3,4-dihydroxy-2a-methyl-5-oxo-2a,3,4,5,6,6a,8,9,14,14b-decahydro-1H-benzo[5',6'][1,4]oxazino[4',3':1,2]pyrido[3,4-b]indole-8-carboxylate (17): Compound **8e** (0.1 mmol) was dissolved in degassed acetone (0.3 mL) and a solution of NMO (0.2 mmol) in degassed water (0.2 mL) was added at rt. Then, OSO_4 (10 mol%, 2.5 wt% in tert-Butanol) solution was added dropwise and stirring was continued for 16 h. After completion, the reaction mixture was quenched with saturated solution of $Na_2S_2O_3.5H_2O$ and stirring was continued for another 30 min. Then, the reaction mixture was passed through a celite pad and the filtrate was concentrated. Then, the crude was dissolved with DCM (30 mL) and washed with water (2 x 20 mL). The organic layer was separated, dried over Na_2SO_4 and concentrated under vacuum. The crude was purified on flash chromatography, using EtOAc in hexane as an eluent to deliver the title compound **17**.

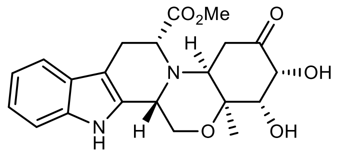

White solid, 17.5 mg, 44% yield; mp: 228–230 °C; R_f = 0.55 (silica gel, hexane/EtOAc 1:4); $[\alpha]_D$ = +105.5 (0.0022 M in acetone); ^{1}H NMR (500 MHz, $CDCl_3$) δ: 7.71 (s, 1H), 7.48 (d, *J* = 7.8 Hz, 1H), 7.36 (d, *J* = 8.1 Hz, 1H), 7.22 (t, *J* = 7.5 Hz, 1H), 7.15 (t, *J* = 7.4 Hz, 1H), 4.71 – 4.66 (m, 1H), 4.38 (d, *J* = 10.6 Hz, 1H), 4.14 (dd, *J* = 11.0, 4.0 Hz, 1H), 4.06 (t, *J* = 11.0 Hz, 1H), 4.03 – 3.98 (m, 1H), 3.84 (s, 3H), 3.69 – 3.62 (m, 2H), 3.43 (dd, *J* = 10.9, 5.8 Hz, 1H), 3.23 – 3.12 (m, 2H), 3.05 – 2.98 (m, 1H), 2.73 – 2.65 (m, 1H), 2.49 (d, *J* = 1.5 Hz, 1H), 1.71 (s, 3H); ^{13}C NMR (125 MHz, $CDCl_3$): δ 209.0, 172.4, 136.5, 129.3, 126.6, 122.4, 120.1, 118.3, 111.1, 107.5, 77.70, 76.6, 74.5, 64.3, 60.7, 57.6, 52.5, 49.7, 32.2, 26.9, 18.6. HRMS (m/z): $[M+H]^+$ calcd. for $C_{21}H_{25}N_2O_6$, 401.1712, found 401.1710.

General reaction procedure for the preparation of compounds 18-20: Compound (**5a/6c/8a**, 0.1 mmol) was dissolved in THF:AcOH:water (0.4:0.4:0.4 mL) and *N*-bromosuccinamide (0.1 mmol) was added slowly at -10 °C. The reaction mixture was slowly warmed to room temperature and stirring continued for 2 h. After completion, the reaction mixture was quenched slowly with saturated sodiumbicarbonate solution at 0 ^{o}C. Then, the reaction mixture was diluted with DCM (30 mL) and washed with water (2 x 20 mL). The organic layer was separated, dried over Na_2SO_4 and concentrated under vacuum. The crude was purified on flash chromatography, using EtOAc in hexane as an eluent to obtain the title compounds **18-20**.

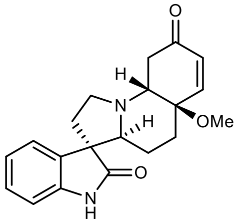

(3S,3a'S,5a'R,9a'R)-5a'-Methoxy-1',2',3a',4',5',5a',9',9a'-octahydro-8'H-spiro[indoline-3,3'-pyrrolo[1,2-a]quinoline]-2,8'-dione (18): Off-white solid, 17 mg, 51% yield; mp: 213–215 °C; R_f = 0.4 (silica gel, hexane/EtOAc 1:4); ^{1}H NMR (500 MHz, Acetone-d_6): δ 9.35 (s, 1H), 7.39 (d, *J* = 7.3 Hz, 1H), 7.18 (td, *J* = 7.7, 1.1 Hz, 1H), 7.00 (t, *J* = 7.5 Hz, 1H), 6.91 (d, *J* = 7.7 Hz, 1H), 6.82 (d, *J* = 10.3 Hz, 1H), 5.97 (d, *J* = 10.3 Hz, 1H), 4.05 – 3.98 (m, 1H), 3.31 (s, 3H), 3.20 – 3.11 (m, 1H), 3.09 – 3.01 (m, 2H), 2.72 (d, *J* = 8.8 Hz, 2H), 2.32 – 2.23 (m, 1H), 1.98 – 1.88 (m, 1H), 1.77 – 1.63 (m, 2H), 1.40 – 1.27 (m, 2H), 1.03 – 0.95 (m, 1H); ^{13}C NMR (125 MHz, Acetone-d_6): δ 198.8, 180.4, 156.7, 142.3, 134.5, 131.2, 128.3, 125.9, 122.4, 109.9, 75.4, 62.4, 57.1, 55.1, 50.7, 46.9, 35.4, 33.4, 30.4, 21.3; HRMS (m/z): $[M+H]^+$ calcd. for $C_{20}H_{23}N_2O_3$, 339.1708, found 339.1715.

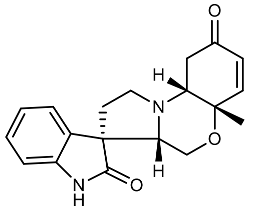

(3S,3aS,5aS,9aR)-5a-Methyl-1,2,3a,4,9,9a-hexahydrospiro[benzo[b]pyrrolo[1,2-d][1,4]oxazine-3,3'-indoline]-2',8(5aH)-dione (**19**): Off-white solid, 17 mg, 52% yield; mp: 215–217 °C; R_f = 0.45 (silica gel, hexane/EtOAc 1:4); ^{1}H NMR (500 MHz, CD_3OD): δ 7.31 (d, *J* = 7.4 Hz, 1H),

7.22 (t, *J* = 7.7 Hz, 1H), 7.07 (t, *J* = 7.5 Hz, 1H), 6.87 (d, *J* = 7.7 Hz, 1H), 6.67 (d, *J* = 10.8 Hz, 1H), 6.03 (d, *J* = 10.8 Hz, 1H), 3.70 (t, *J* = 10.5 Hz, 1H), 3.49 (t, *J* = 8.3 Hz, 1H), 3.28 (dd, *J* = 10.5, 2.5 Hz, 1H), 2.88 – 2.80 (m, 2H), 2.77 (t, *J* = 9.1 Hz, 1H), 2.69 (dd, *J* = 10.1, 2.2 Hz, 1H), 2.49 (dd, *J* = 17.4, 8.5 Hz, 1H), 2.28 (dt, *J* = 13.0, 9.0 Hz, 1H), 2.07 – 2.01 (m, 1H), 1.43 (s, 3H) ^{13}C NMR (125 MHz, CD_3OD): δ 199.2, 181.9, 155.6, 142.6, 134.4, 130.9, 129.4, 124.1, 123.8, 110.6, 74.4, 72.6, 69.7, 64.8, 55.7, 52.7, 41.3, 35.4, 24.9; HRMS (m/z): $[M+H]^+$ calcd. for $C_{19}H_{21}N_2O_3$, 325.1552, found 325.1555.

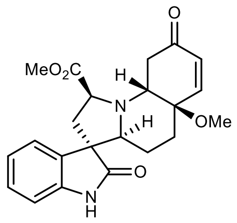

Methyl (1'S,3S,3a'S,5a'R,9a'R)-5a'-methoxy-2,8'-dioxo-1',2',3a',4',5a',8',9',9a'-octahydro-5'H-spiro[indoline-3,3'-pyrrolo[1,2-a]quinoline]-1'-carboxylate (20): Off-white solid, 19 mg, 48% yield; mp: 246–248 °C; R_f = 0.4 (silica gel, hexane/EtOAc 1:4); $[\alpha]_D$ = -12.5 (0.002 M in acetone); ^{1}H NMR (500 MHz, Acetone-d_6): δ 8.78 (s, 1H), 7.34 (d, *J* = 7.3 Hz, 1H), 7.11 (td, *J* = 7.7, 0.9 Hz, 1H), 6.95 (t, *J* = 7.5 Hz, 1H), 6.80 (d, *J* = 7.7 Hz, 1H), 6.52 (dd, *J* = 10.2, 2.1 Hz, 1H), 6.08 (d, *J* = 10.2 Hz, 1H), 4.07 (dd, *J* = 9.0, 1.5 Hz, 1H), 3.74 (s, 3H), 3.44 (dd, *J* = 10.1, 3.6 Hz, 1H), 3.27 (s, 3H), 3.20 (d, *J* = 2.3 Hz, 1H), 3.03 (dd, *J* = 16.4, 3.0 Hz, 1H), 2.56 – 2.49 (m, 1H), 2.12 (d, *J* = 9.3Hz, 1H), 1.46 – 1.25 (m, 3H); ^{13}C NMR (125 MHz, Acetone-d_6): δ 197.9, 179.8, 173.8, 148.0, 141.4, 132.5, 132.1, 128.0, 123.8, 122.3, 109.1, 71.5, 69.0, 62.0, 60.5, 55.6, 51.3, 50.5, 38.4, 37.9, 32.2, 21.5.HRMS (m/z): $[M+H]^+$ calcd. for $C_{22}H_{25}N_2O_5$, 397.1763, found 397.1774.

General reaction procedure for the preparation of 22a-d: Aldehyde (**2b**, 0.5 mmol) was dissolved in DCM (0.5 mL) and molecular sieves were added at -78 °C. Then, a solution of amine (**21**, 0.5 mmol) in DCM (0.5 mL) was added dropwise and stirring was continued at -78 °C. After 45 min, a second equivalent of solution of amine (**21**, 0.5 mmol) in DCM (0.5 mL) was added dropwise and stirring was continued at -78 °C for 2 h. Then, the reaction mixture was slowly warmed to rt and stirring was continued at room temperature for 10h. After completion, DCM was removed under vacuum and the crude was purified on flash chromatography, using EtOAc in hexane as eluent to produce the title compounds **22a-d**.

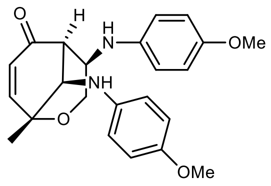

(1R,4R,5S,9R)-4,9-Bis((4-methoxyphenyl)amino)-1-methyl-2-oxabicyclo[3.3.1]non-7-en-6-one (22a): White solid, 124 mg, 63% yield; mp: 158–160 °C; R_f = 0.45 (silica gel, hexane/EtOAc 1:1); ^{1}H NMR (500 MHz, $CDCl_3$): δ 6.85 – 6.79 (m, 2H), 6.76 – 6.69 (m, 2H), 6.68 – 6.62 (m, 3H), 6.55 – 6.49 (m, 3H), 4.06 (dd, *J* = 12.0, 5.8 Hz, 1H), 3.98 – 3.91 (m, 1H), 3.84 (d, *J* = 3.2 Hz, 1H), 3.77 (s, 3H), 3.73 (s, 3H), 3.31 (t, *J* = 12.0 Hz, 1H), 3.21 – 3.15 (m, 1H), 1.50 (s, 3H); ^{13}C NMR (125 MHz, $CDCl_3$): δ 200.3, 152.7, 147.6, 140.0, 134.3, 115.3, 115.1, 115.0, 114.8, 71.1, 64.0, 56.4, 55.8, 55.7, 49.3, 46.4, 23.7; HRMS (m/z): $[M+H]^+$ calcd. for $C_{23}H_{27}N_2O_4$, 395.1970, found 395.1969.

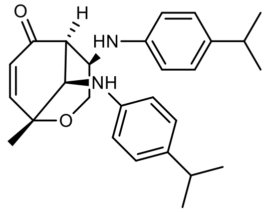

(1R,4R,5S,9R)-4,9-Bis((4-isopropylphenyl)amino)-1-methyl-2-oxabicyclo[3.3.1]non-7-en-6-one (22b): White solid, 127 mg, 67% yield; mp: 124–126 °C; R_f = 0.5 (silica gel, hexane/EtOAc 1:1); ^{1}H NMR (500 MHz, $CDCl_3$): δ 7.08 (t, *J* = 5.5 Hz, 2H), 7.00 (t, *J* = 8.8 Hz, 2H), 6.67 (d, *J* = 9.9 Hz, 1H), 6.65 – 6.59 (m, 2H), 6.56 – 6.46 (m, 3H), 4.09 (dd, *J* = 12.0, 5.9 Hz, 1H), 4.05 – 3.97 (m, 1H), 3.91 (d, *J* = 3.2 Hz, 1H), 3.32 (t, *J* = 11.9 Hz, 1H), 3.20 (t, *J* = 3.9 Hz, 1H), 2.87 – 2.75 (m, 2H), 1.52 (s, 3H), 1.24 (d, *J* = 6.9 Hz, 6H), 1.19 (d, *J* = 6.9 Hz, 6H); ^{13}C NMR (125 MHz, $CDCl_3$) δ: 200.4, 147.7, 143.7, 139.0, 138.7, 134.3, 127.6, 127.2, 113.3, 113.2, 71.0, 64.0, 55.5, 49.3, 45.5, 33.2, 33.1, 24.2, 24.2, 24.1, 23.7; HRMS (m/z): $[M+H]^+$ calcd. for $C_{27}H_{35}N_2O_2$, 419.2698, found 419.2697.

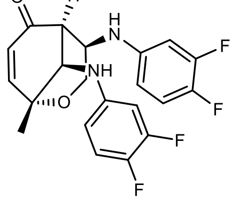

(1R,4R,5S,9R)-4,9-Bis((3,4-difluorophenyl)amino)-1-methyl-2-oxabicyclo[3.3.1]non-7-en-6-one (22c): White solid, 117 mg, 58% yield; mp: 160–163 °C; R_f = 0.55 (silica gel, hexane/EtOAc 1:1); ^{1}H NMR (500 MHz, Acetone-d$_6$): δ 7.14 – 7.06 (m, 1H), 7.04 – 6.95 (m, 1H), 6.94 – 6.86 (m, 1H), 6.75 – 6.70 (m, 2H), 6.51 – 6.44 (m, 1H), 6.42 (d, *J*= 10.0 Hz, 1H), 6.39 – 6.33 (m, 1H), 5.69 (d, *J* = 10.0 Hz, 1H), 4.53 (d, *J* = 9.3 Hz, 1H), 4.22 – 4.12 (m, 2H), 3.87 (dd, *J* = 11.8, 6.0 Hz, 1H), 3.38 (t, *J* = 11.8 Hz, 1H), 3.12 – 3.06 (m, 1H), 1.46 (s, 3H); ^{13}C NMR (125 MHz, Acetone-d$_6$): δ 198.2, 151.64 (dd), 149.72 (dd) 146.7, 144.74 (d), 144.40 (d),143.34 (dd), 141.48 (dd), 133.94, 117.47 (dd), 108.86 (dd), 108.52 (dd), 101.78 (d), 101.18 (d), 70.9, 62.5, 54.9, 48.5, 45.2, 23.0; HRMS (m/z): [M+H]$^+$ calcd. for $C_{21}H_{19}F_4N_2O_2$, 407.1382, found 407.1379.

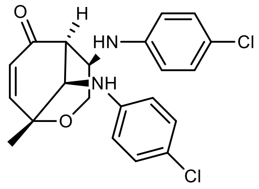

(1R,4R,5S,9R)-4,9-Bis((4-chlorophenyl)amino)-1-methyl-2-oxabicyclo[3.3.1]non-7-en-6-one (22d): Off-white solid, 116 mg, 58% yield; mp: 200–202 °C; R_f = 0.55 (silica gel, hexane/EtOAc 1:1); ^{1}H NMR (500 MHz, CDCl$_3$): δ 7.20 – 7.16 (m, 2H), 7.11 – 7.06 (m, 2H), 6.68 (d, *J*= 9.9 Hz, 1H), 6.63 – 6.59 (m, 2H), 6.54 (dd, *J* = 9.9, 1.0 Hz, 1H), 6.46 (d, *J*= 8.8 Hz, 2H), 4.05 (dd, *J* = 12.1, 5.9 Hz, 1H), 3.96 (dt, *J* = 11.1, 5.4 Hz, 1H), 3.87 (d, *J* = 3.3 Hz, 1H), 3.30 (t, *J* = 12.0 Hz, 1H), 3.16 (t, *J* = 3.9 Hz, 1H), 1.50 (s, 3H); ^{13}C NMR (125 MHz, CDCl$_3$): δ 199.7, 147.5, 144.4, 134.4, 129.6, 129.3, 123.2, 114.5, 114.4, 71.0, 63.5, 55.5, 49.0, 45.4, 23.7. HRMS (m/z): [M+H]$^+$ calcd. for $C_{21}H_{21}Cl_2N_2O_2$, 403.0980, found 403.0981.

General reaction procedure for the preparation of 24a-f: Aldehyde (**2b**, 0.5 mmol) was dissolved in DCM (0.5 mL) and molecular sieves were added at -78 °C. Then, a solution of amine (**23**, 0.5 mmol) in DCM (0.5 mL) was added dropwise and stirring was continued at -78 °C. After 45 min, a second equivalent of solution of amine (**23**, 0.5 mmol) in DCM (0.5 mL) was added dropwise and stirring was continued at -78 °C for 2h. Then, the reaction mixture was slowly warmed to rt and stirring was continued for 10h. After completion, DCM was removed under vacuum and the crude was purified on flash chromatography, using EtOAc in hexane as eluent to deliver the title compounds **24a-d**.

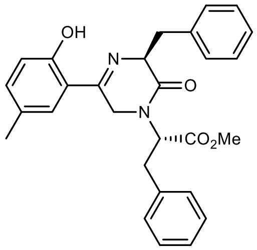

Methyl (S)-2-((S)-3-benzyl-5-(2-hydroxy-5-methylphenyl)-2-oxo-3,6-dihydropyrazin-1(2H)-yl)-3-phenylpropanoate (24a): Yellowish solid, 148 mg, 68% yield; mp: 120–123 °C; R_f = 0.6 (silica gel, hexane/EtOAc 1:1); $[\alpha]_D$ = -39.5 (0.0052 M in acetone); ^{1}H NMR (500 MHz, Acetone-d_6): δ 13.39 (s, 1H), 7.34 – 7.15 (m, 12H), 6.79 (d, *J* = 8.4 Hz, 1H), 5.36 (dd, *J* = 10.3, 5.7 Hz, 1H), 4.69 – 4.63 (m, 1H), 4.51 – 4.45 (m, 1H), 4.39 – 4.33 (m, 1H), 3.76 (s, 3H), 3.47 – 3.40 (m, 1H), 3.31 – 3.23 (m, 2H), 3.02 – 2.95 (m, 1H), 2.26 (s, 3H); ^{13}C NMR (125 MHz, CD_3OD): δ 170.2, 168.2, 166.0, 158.4, 136.7, 136.4, 133.5, 129.4, 128.6, 128.3, 127.9, 127.4, 126.7, 126.6, 126.4, 117.1, 115.8, 61.9, 58.9, 51.6, 47.3, 38.7, 33.7, 19.1; HRMS (m/z): $[M+H]^+$ calcd. for $C_{28}H_{29}N_2O_4$, 457.2127, found 457.2129.

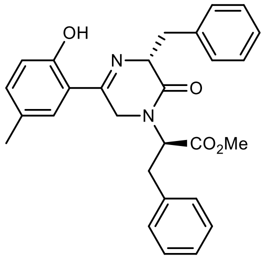

Methyl (R)-2-((R)-3-benzyl-5-(2-hydroxy-5-methylphenyl)-2-oxo-3,6-dihydropyrazin-1(2H)-yl)-3-phenylpropanoate (24b): Yellowish solid, 148 mg, 65% yield; mp: 121–123 °C; R_f = 0.6 (silica gel, hexane/EtOAc 1:1); $[\alpha]_D$ = -35.7 (0.0015 M in acetone); ^{1}H NMR (500 MHz, $CDCl_3$): δ 7.31 – 7.25 (m, 3H), 7.24 – 7.18 (m, 6H), 7.16 (dd, *J* = 8.4, 1.7 Hz, 1H), 7.13 – 7.09 (m, 2H), 6.90 (d, *J* = 8.4 Hz, 1H), 6.82 – 6.80 (d, *J* = 1.0 Hz, 1H), 5.43 (dd, *J* = 8.9, 6.8 Hz, 1H), 4.73 – 4.68 (m, 1H), 4.26 (dd, *J* = 17.2, 2.8 Hz, 1H), 3.76 – 3.70 (m, 4H), 3.35 – 3.24 (m, 2H), 3.07 – 2.96 (m, 2H), 2.29 (s, 3H); ^{13}C NMR (125 MHz, Acetone-d_6): δ 170.2, 166.7, 166.4, 137.5, 137.2, 133.6, 129.5, 128.9, 128.5, 128.1, 127.2, 126.9, 126.7, 126.4, 117.5, 116.1, 61.7, 57.6, 51.7, 45.6, 38.8, 33.9, 19.6; HRMS (m/z): $[M+H]^+$ calcd. for $C_{28}H_{29}N_2O_4$, 457.2127, found 457.2128.

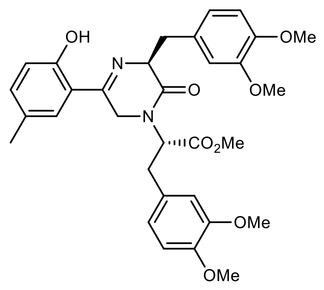

Methyl (S)-2-((S)-3-(3,4-dimethoxybenzyl)-5-(2-hydroxy-5-methylphenyl)-2-oxo-3,6-dihydropyrazin-1(2H)-yl)-3-(3,4-dimethoxyphenyl)propanoate (24c): Yellowish solid, 158 mg, 58% yield; mp: 155–158 °C; R_f = 0.45 (silica gel, hexane/EtOAc 1:1); $[\alpha]_D$ = -22.2 (0.0048 M in acetone); ^{1}H NMR (500 MHz, Acetone-d_6): δ 7.20 (s, 1H), 7.16 (dd, *J* = 8.4, 1.6 Hz, 1H), 6.84 (d, *J* = 1.6 Hz, 1H), 6.80 – 6.71 (m, 5H), 6.62 (dd, *J* = 8.1, 1.9 Hz, 1H), 5.28 (dd, *J* = 10.5, 5.5 Hz, 1H), 4.64 – 4.56 (m, 1H), 4.38 (dd, *J* = 17.6, 2.8 Hz, 1H), 4.21 (dd, *J* = 17.6, 1.9 Hz, 1H), 3.75 (s, 6H), 3.70 (s, 3H), 3.67 (s, 3H), 3.64 (s, 3H), 3.30 (dd, *J* = 14.6, 5.5 Hz, 1H), 3.18 (dt, *J* = 14.6, 8.2 Hz, 2H), 2.98 – 2.90 (s, 1H), 2.23 (s, 3H); ^{13}C NMR (125 MHz, Acetone-d_6): δ 170.3, 166.9, 166.6, 159.3, 149.5, 149.0, 148.4, 148.3, 133.6, 129.7, 129.3, 127.2, 126.9, 121.5, 121.0, 117.5, 116.2, 113.7, 112.6, 112.0, 111.8, 62.0, 57.6, 55.1 (2C), 55.0, 54.8, 51.7, 45.6, 38.3, 33.5, 19.6; HRMS (m/z): $[M+H]^+$ calcd. for $C_{32}H_{37}N_2O_8$, 577.2549, found 577.2550.

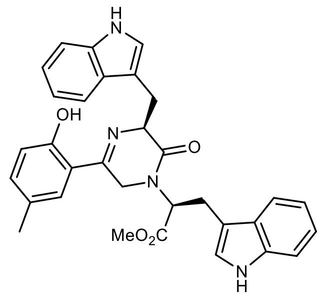

Methyl (S)-2-((S)-3-((1H-indol-3-yl)methyl)-5-(2-hydroxy-5-methylphenyl)-2-oxo-3,6-dihydropyrazin-1(2H)-yl)-3-(1H-indol-3-yl)propanoate (24d): Yellowish solid, 28 mg, 48% yield; mp: 138–140 °C; R_f = 0.4 (silica gel, hexane/EtOAc 1:3); $[\alpha]_D$ = +45.5 (0.0020 M in acetone); ^{1}H NMR (500 MHz, Acetone-d_6): δ 9.98 (bs, 2H), 7.61 – 7.53 (m, 2H), 7.40 – 7.34 (m, 2H), 7.14 – 7.02 (m, 5H), 6.99 – 6.94 (m, 1H), 6.93 – 6.88 (m, 2H), 6.77 – 6.73 (m, 1H), 5.43 (dd, *J* = 8.7, 6.6 Hz, 1H), 4.77 – 4.71 (m, 1H), 4.48 – 4.41(m, 1H), 3.96 – 3.89 (m, 1H), 3.64 (s, 3H), 3.41 – 3.30 (m, 2H), 3.24 – 3.17 (m, 1H), 3.07 – 2.99 (m, 1H), 2.14 (s, 3H); ^{13}C

NMR (125 MHz, Acetone-d_6): δ 170.8, 166.9, 165.9, 159.4, 136.6, 136.5, 133.4, 127.7, 127.3, 126.9, 126.7, 124.0, 123.2, 121.4, 121.3, 118.9, 118.8, 118.6, 118.3, 117.4, 116.2, 111.4, 111.2, 110.0, 109.8, 61.2, 56.1, 51.5, 45.0, 29.2, 23.8, 19.6; HRMS (m/z): $[M+H]^+$ calcd. for $C_{32}H_{31}N_4O_4$, 535.2345, found 535.2345.

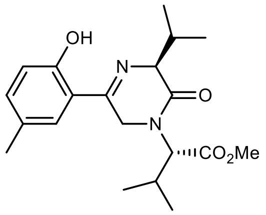

Methyl (S)-2-((S)-5-(2-hydroxy-5-methylphenyl)-3-isopropyl-2-oxo-3,6-dihydropyrazin-1(2H)-yl)-3-methylbutanoate (24e): Colorless oil, 99 mg, 55% yield; R_f = 0.55 (silica gel, hexane/EtOAc 2:1); $[\alpha]_D$ = -25.0 (0.0038 M in acetone); ^{1}H NMR (500 MHz, Acetone-d_6): δ 13.9 (s, 1H), 7.47 (s, 1H), 7.20 (d, *J* = 7.5 Hz, 1H), 6.83 (d, *J* = 8.4 Hz, 1H), 4.99 – 4.72 (m, 3H), 4.25 (d, *J* = 2.1 Hz, 1H), 3.69 (s, 3H), 2.62 – 2.39 (m, 2H), 2.28 (s, 3H), 1.15 (d, *J* = 6.8 Hz, 3H), 1.05 (d, *J* = 6.8 Hz, 3H), 0.89 (dd, *J* = 11.2, 6.8 Hz, 6H); ^{13}C NMR (125 MHz, Acetone-d_6): δ 171.5, 167.5, 167.5, 160.5, 134.6, 128.2, 127.9, 118.5, 117.2, 66.2, 62.1, 52.2, 45.5, 32.2, 27.8, 20.5, 20.2, 20.1 19.5, 17.7; HRMS (m/z): $[M+H]^+$ calcd. for $C_{20}H_{29}N_2O_4$, 361.2127, found 361.2135.

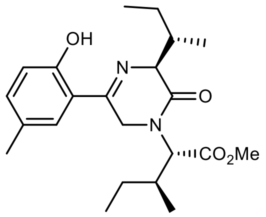

Methyl (2S,3S)-2-((S)-3-((S)-sec-butyl)-5-(2-hydroxy-5-methylphenyl)-2-oxo-3,6-dihydropyrazin-1(2H)-yl)-3-methylpentanoate (24f): Colorless oil, 100 mg, 52% yield; R_f = 0.55 (silica gel, hexane/EtOAc 2:1); $[\alpha]_D$ = +8.6 (0.0059 M in acetone); ^{1}H NMR (500 MHz, Acetone-d_6): δ 13.9 (s, 1H), 7.47 (s, 1H), 7.21 – 7.18 (m, 1H), 6.82 (d, *J* = 8.4 Hz, 1H), 4.99 (d, *J* = 10.7 Hz, 1H), 4.93 (dd, *J* = 18.0, 3.1 Hz, 1H), 4.74 (dd, *J* = 18.0, 2.0 Hz, 1H), 4.32 – 4.25 (m, 1H), 3.69 (s, 3H), 2.32 – 2.24 (s, 5H), 1.53 – 1.42 (m, 2H), 1.22 – 1.08 (m, 5H), 1.01 (d, *J* = 6.6 Hz, 3H), 0.93 – 0.84 (m, 6H); ^{13}C NMR (125 MHz, Acetone-d_6): δ 171.8, 167.6, 167.4, 160.4,

134.6, 128.1, 127.9, 118.4, 117.2, 66.0, 60.1, 52.2, 45.1, 38.0, 33.4, 25.9, 25.8, 20.5, 16.6, 16.1, 12.0, 10.4; HRMS (m/z): $[M+H]^+$ calcd. for $C_{22}H_{33}N_2O_4$, 389.2440, found 389.2445.

Reaction procedure for the preparation of (2aR,5S,6aR,14bS)-2a-methoxy-1,2,2a,5,6,6a,8,9,14,14b-decahydroindolo[2',3':3,4]pyrido[1,2-a]quinolin-5-ol (25): Compound **5a** (0.1 mmol) was dissolved in methanol (2.0 mL) and $CeCl_3.7\ H_2O$ (0.1 mmol) was added at -20 °C. Then, $NaBH_4$ (0.11 mmol) was added slowly and stirring was continued at -20 °C for 115 min. After completion, the reaction mixture was quenched with ice at 0 °C. Then, the reaction mixture was diluted with EtOAc (30 mL) and washed with water (2 x 20 mL). The organic layer was separated, dried over Na_2SO_4 and concentrated under vacuum. The crude was purified on flash chromatography, using 60 % EtOAc in hexane as an eluent to produce the title compound **25**.

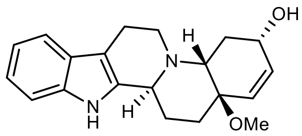

Half white solid, 26 mg. 86% yield; mp: 240–242 °C; R_f = 0.5 (silica gel, hexane/EtOAc 0:1); ^1H NMR (500 MHz, CD_3OD): δ 7.30 (d, *J* = 7.7 Hz, 1H), 7.19 (d, *J* = 8.0 Hz, 1H), 6.95 (t, *J* = 7.3 Hz, 1H), 6.88 (t, *J* = 7.3 Hz, 1H), 5.72 (d, *J* = 10.3 Hz, 1H), 5.46 (dd, *J* = 10.3, 1.5 Hz, 1H), 4.34 – 4.23 (m, 1H), 3.89 (d, *J* = 10.8 Hz, 1H), 3.32 – 3.20 (m, 1H), 3.13 (s, 3H), 3.08 – 2.94 (m, 2H), 2.92 – 2.80 (m, 1H), 2.66 (d, *J* = 14.8 Hz, 1H), 2.27 (d, *J* = 8.7 Hz, 1H), 1.98 – 1.92 (m, 1H), 1.90 – 1.71 (m, 4H); ^{13}C NMR (125 MHz, CD_3OD): δ 136.7, 134.9, 133.8, 132.5, 126.9, 120.6, 118.4, 117.4, 110.6, 106.7, 74.9, 67.6, 58.3, 51.5, 49.4, 49.1, 31.8, 29.3, 25.2, 21.4. HRMS (m/z): $[M+H]^+$ calcd. for $C_{20}H_{25}N_2O_2$, 325.1916, found 325.1922.

General reaction procedure for the preparation of 26a-e: Compound (**5/6/8**, 0.2 mmol) was dissolved in methanol (1.0 mL) and 10 wt% Pd/C (5 mol%) was added and stirring was continued under hydrogen atmosphere for 3-4 h at rt. After completion, the reaction mixture was filtered through a celite pad and the filtrate was concentrated. The crude was purified on flash chromatography, using EtOAc in hexane as an eluent to produce the title compounds **26a-e**.

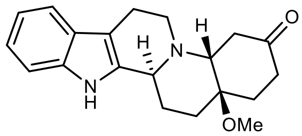

(2aR,6aR,14bS)-2a-Methoxy-2,2a,3,4,6,6a,8,9,14,14b-decahydroindolo[2',3':3,4]pyrido[1,2-a]quinolin-5(1H)-one (26a): Off-white solid, 58 mg, 90% yield; mp: 183–185 °C; R_f = 0.45 (silica gel, hexane/EtOAc 1:1); ^{1}H NMR (500 MHz, $CDCl_3$): δ 7.86 (s, 1H), 7.48 (d, J = 7.6 Hz, 1H), 7.35 (d, J = 7.6 Hz, 1H), 7.20 – 7.09 (m, 2H), 4.29 – 4.21 (m, 1H), 3.48 – 3.36 (m, 1H), 3.28 – 3.21 (m, 4H), 3.05 – 2.96 (m, 1H), 2.87 (t, J = 13.0 Hz, 2H), 2.62 (d, J = 13.0 Hz, 2H), 2.53 – 2.37 (m, 2H), 2.36 – 2.27 (m, 1H), 2.15 – 2.05 (m, 2H), 2.03 – 1.88 (m, 2H), 1.70 – 1.59 (s, 1H); ^{13}C NMR (125 MHz, $CDCl_3$): δ 211.1, 135.9, 133.2, 127.8, 121.6, 119.7, 118.2, 110.9, 109.0, 73.6, 61.0, 53.5, 48.6, 48.2, 38.3, 37.2, 28.5, 27.3, 24.6, 18.8; HRMS (m/z): $[M+H]^+$ calcd. for $C_{20}H_{25}N_2O_2$, 325.1916, found 325.1908.

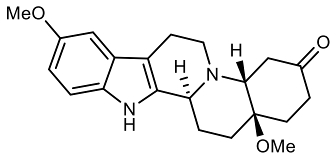

(2aR,6aR,14bS)-2a,11-dimethoxy-2,2a,3,4,6,6a,8,9,14,14b-decahydroindolo[2',3':3,4]pyrido[1,2-a]quinolin-5(1H)-one (26b): Off-white solid, 67 mg, 95% yield; mp: 180–183 °C; R_f = 0.4 (silica gel, hexane/EtOAc 1:1); ^{1}H NMR (500 MHz, Acetone): δ 9.73 (s, 1H), 7.17 (t, J = 10.1 Hz, 1H), 6.91 (d, J = 2.4 Hz, 1H), 6.68 (dd, J = 8.7, 2.4 Hz, 1H), 4.21 – 4.12 (m, 1H), 3.78 (s, 3H), 3.30 (dd, J= 12.8, 4.6 Hz, 1H), 3.24 – 3.17 (m, 4H), 2.95 – 2.88 (m, 1H), 2.85 – 2.69 (m, 3H), 2.64 (dd, J = 13.8, 7.0 Hz, 1H), 2.55 – 2.49 (m, 1H), 2.38 – 2.30 (m, 1H), 2.25 (dt, J = 16.0, 5.3 Hz, 2H), 2.18 – 2.12 (m, 1H), 2.11 – 2.07 (m, 1H), 1.91 (dd, J = 12.3, 6.3 Hz, 1H), 1.59 – 1.50 (m, 1H); ^{13}C NMR (125 MHz, Acetone): δ 209.6, 154.8, 136.1, 132.3, 128.9, 112.3, 111.3, 108.3, 100.7, 74.3, 61.8, 55.8, 54.0, 48.9, 48.4, 38.6, 37.7, 29.2, 28.2, 25.1, 20.1; HRMS (m/z): $[M+H]^+$ calcd. for $C_{21}H_{27}N_2O_3$, 355.2021, found 355.2029.

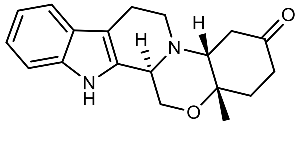

(2aS,6aR,14bR)-2a-Methyl-3,4,6,6a,8,9,14,14b-octahydro-1H-benzo[5',6'][1,4]oxazino[4',3':1,2]pyrido[3,4-b]indol-5(2aH)-one (26c): Off-white solid, 57 mg, 92% yield; mp: 236–238 °C; R_f = 0.5 (silica gel, hexane/EtOAc 1:1); ^{1}H NMR (500 MHz, $CDCl_3$): δ 7.74 (s, 1H), 7.49 (d, *J* = 7.7 Hz, 1H), 7.32 (d, *J* = 7.9 Hz, 1H), 7.16 (t, *J* = 7.4 Hz, 1H), 7.11 (t, *J* = 7.3 Hz, 1H), 4.17 – 4.05 (m, 2H), 3.89 (t, *J* = 10.6 Hz, 1H), 3.13 – 3.02 (m, 2H), 3.01 – 2.91 (m, 1H), 2.90 – 2.81 (m, 2H), 2.81 – 2.67 (m, 2H), 2.44 (d, *J* = 9.2 Hz, 1H), 2.19 (d, *J* = 11.9 Hz, 2H), 1.80 – 1.71 (m, 1H), 1.49 (s, 3H); ^{13}C NMR (125 MHz, $CDCl_3$): δ 211.7, 136.4, 131.0, 127.1, 121.9, 119.8, 118.3, 111.0, 109.7, 72.2, 64.6, 64.5, 49.8, 49.3, 36.7, 36.6, 35.8, 22.7, 22.1. HRMS (m/z): $[M+H]^+$ calcd. for $C_{19}H_{23}N_2O_2$, 311.1759, found 311.1755.

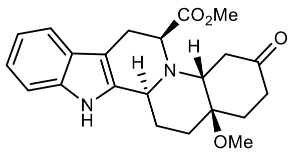

Methyl (2aR,6aR,8S,14bS)-2a-methoxy-5-oxo-1,2,2a,3,4,5,6,6a,8,9,14,14b-dodecahydroindolo[2',3':3,4]pyrido[1,2-a]quinoline-8-carboxylate (26d): Off-white solid, 64 mg, 85% yield; mp: 114–116 °C; R_f = 0.4 (silica gel, hexane/EtOAc 1:2); $[\alpha]_D$ = -92.8 (0.0018 M in acetone); ^{1}H NMR (500 MHz, Acetone) δ: 10.03 (s, 1H), 7.42 (d, *J* = 7.7 Hz, 1H), 7.32 (t, *J* = 10.0 Hz, 1H), 7.07 (t, *J* = 6.0 Hz, 1H), 7.00 (t, *J* = 6.0 Hz, 1H), 4.16 – 4.08 (m, 1H), 3.78 (s, 3H), 3.69 (dd, *J* = 10.8, 3.8 Hz, 1H), 3.47 (dd, *J* = 10.8, 5.0 Hz, 1H), 3.25 (s, 3H), 3.08 – 2.98 (m, 1H), 2.96 – 2.78 (m, 2H), 2.58 – 2.43 (m, 2H), 2.37 – 2.28 (m, 1H), 2.27 – 2.16 (m, 2H), 2.17 – 2.09 (m, 1H), 2.04 – 1.95 (m, 1H), 1.90 – 1.81 (m, 1H), 1.68 – 1.59 (m, 1H); ^{13}C NMR (125 MHz, Acetone): δ 208.6, 173.6, 137.6, 137.4, 135.4, 127.8, 121.7, 119.7, 118.4, 111.8, 106.2, 73.9, 62.7, 58.7, 52.2, 48.2, 38.1, 37.1, 30.6, 28.5, 26.6, 26.5; HRMS (m/z): $[M+H]^+$ calcd. for $C_{22}H_{27}N_2O_4$, 383.1970, found 383.1981.

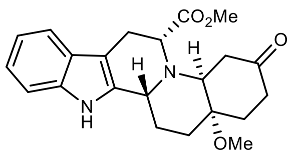

Methyl (2aS,6aS,8R,14bR)-2a-methoxy-5-oxo-1,2,2a,3,4,5,6,6a,8,9,14,14b-dodecahydroindolo[2',3':3,4]pyrido[1,2-a]quinoline-8-carboxylate (26e): Off-white solid, 66 mg, 87% yield; mp: 113–16 °C; R_f = 0.4 (silica gel, hexane/EtOAc 1:2); $[\alpha]_D$ = +95.2 (0.0005 M

in acetone); ^{1}H NMR (500 MHz, Acetone): δ 9.99 (s, 1H), 7.43 (d, *J* = 7.7 Hz, 1H), 7.32 (d, *J* = 8.0 Hz, 1H), 7.06 (t, *J* = 7.5 Hz, 1H), 7.00 (t, *J* = 7.1 Hz, 1H), 4.17 – 4.07 (m, 1H), 3.79 (s, 3H), 3.69 (dd, *J* = 10.6, 3.2 Hz, 1H), 3.47 (dd, *J* = 10.6, 4.8 Hz, 1H), 3.25 (s, 3H), 3.07 – 2.99 (m, 1H), 2.94 – 2.88 (m, 1H), 2.85 – 2.80 (m, 1H), 2.59 – 2.43 (m, 2H), 2.38 – 2.27 (m, 1H), 2.27 – 2.18 (m, 2H), 2.17 – 2.09 (m, 1H), 2.05 – 1.96 (m, 1H), 1.90 – 1.80 (m, 1H), 1.68 – 1.60 (m, 1H); ^{13}C NMR (125 MHz, Acetone): δ 208.6, 173.6, 137.6, 135.4, 127.8, 121.7, 119.7, 118.4, 111.8, 106.2, 73.9, 62.7, 58.7, 52.3, 52.2, 48.2, 38.1, 37.1, 30.6, 28.5, 26.6, 26.5; HRMS (m/z): $[M+H]^+$ calcd. for $C_{22}H_{27}N_2O_4$, 383.1970, found 383.1964.

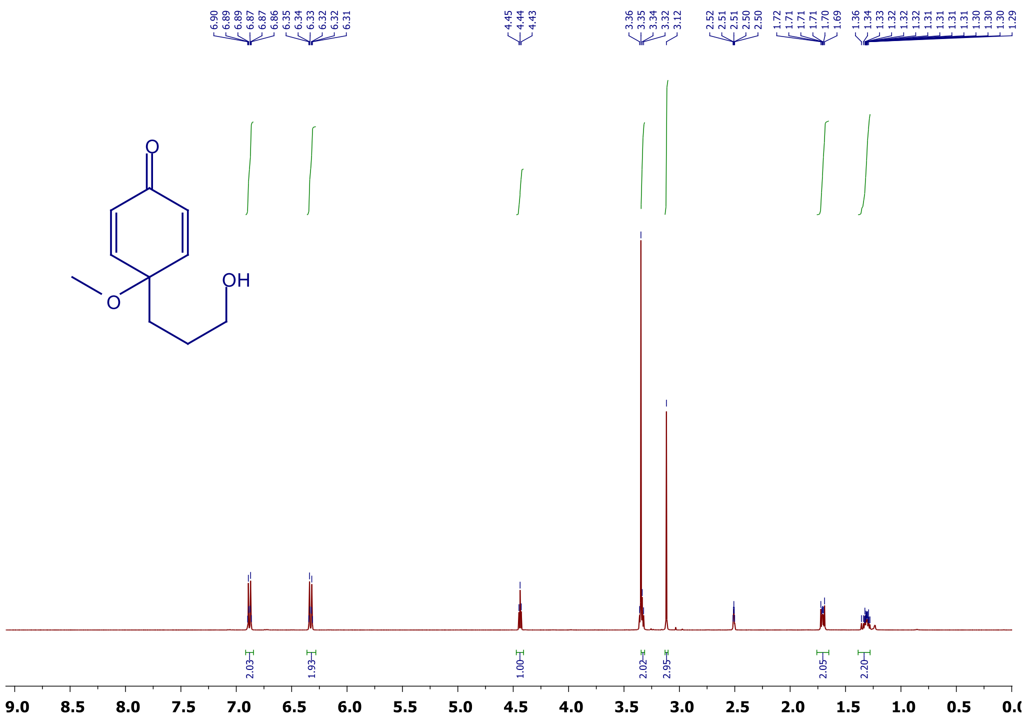

Supplementary Figure 8 ^{1}H NMR (500 MHz, DMSO-d_6) spectrum of 4-(3-hydroxypropyl)-4-methoxycyclohexa-2,5-dien-1-one (**1c**)

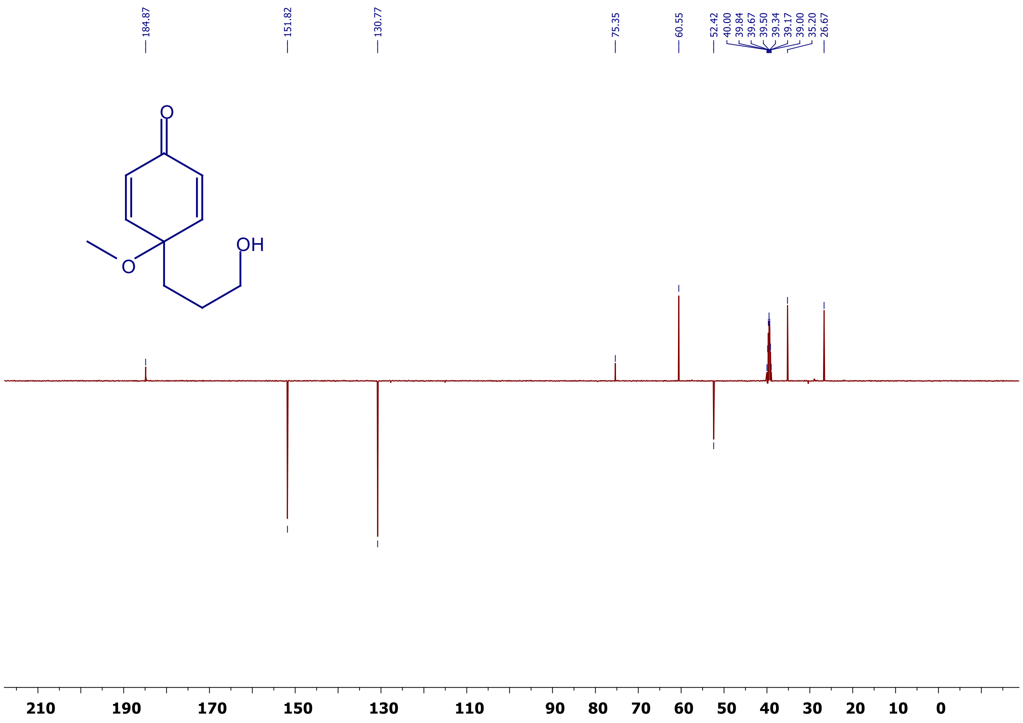

Supplementary Figure 9 APT NMR (125 MHz, DMSO-d_6) spectrum of 4-(3-hydroxypropyl)-4-methoxycyclohexa-2,5-dien-1-one (**1c**)

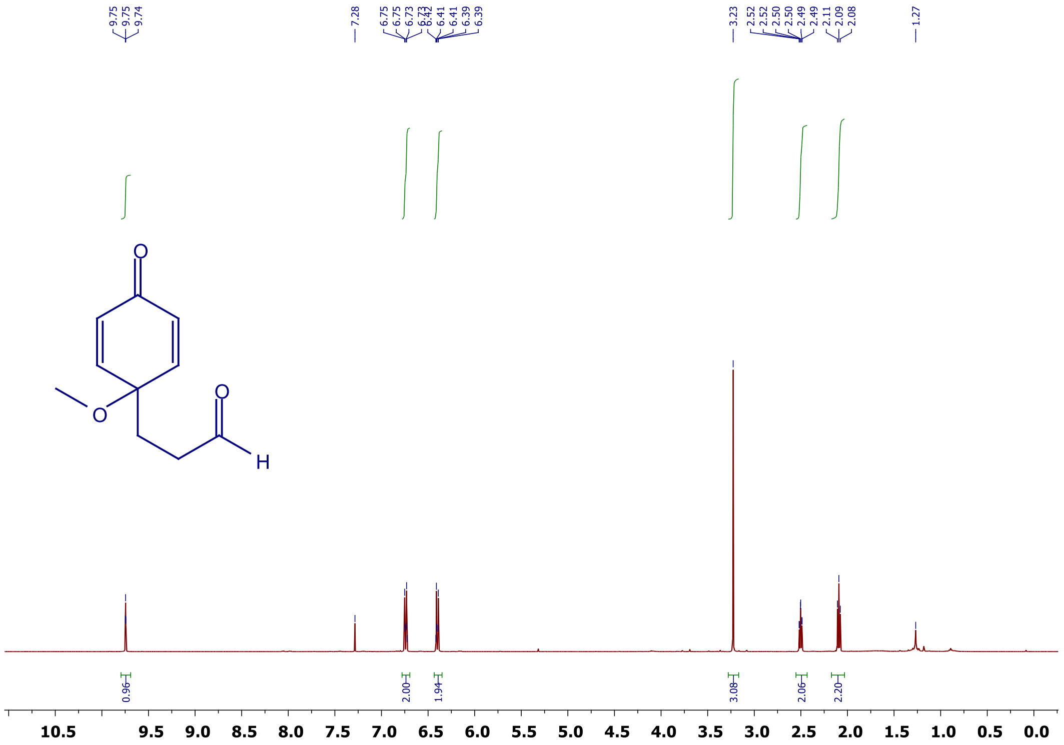

Supplementary Figure 10 ^{1}H NMR (500 MHz, $CDCl_3$) spectrum of 3-(1-methoxy-4-oxocyclohexa-2,5-dien-1-yl)propanal (**2a**)

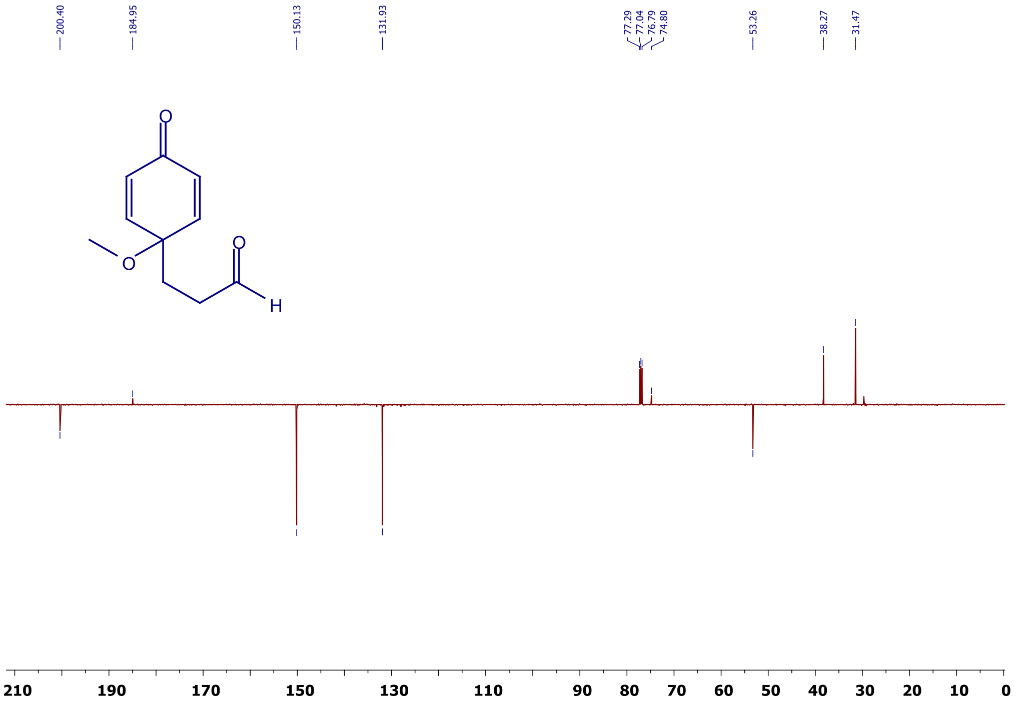

Supplementary Figure 11 APT NMR (125 MHz, $CDCl_3$) spectrum of 3-(1-methoxy-4-oxocyclohexa-2,5-dien-1-yl)propanal (**2a**)

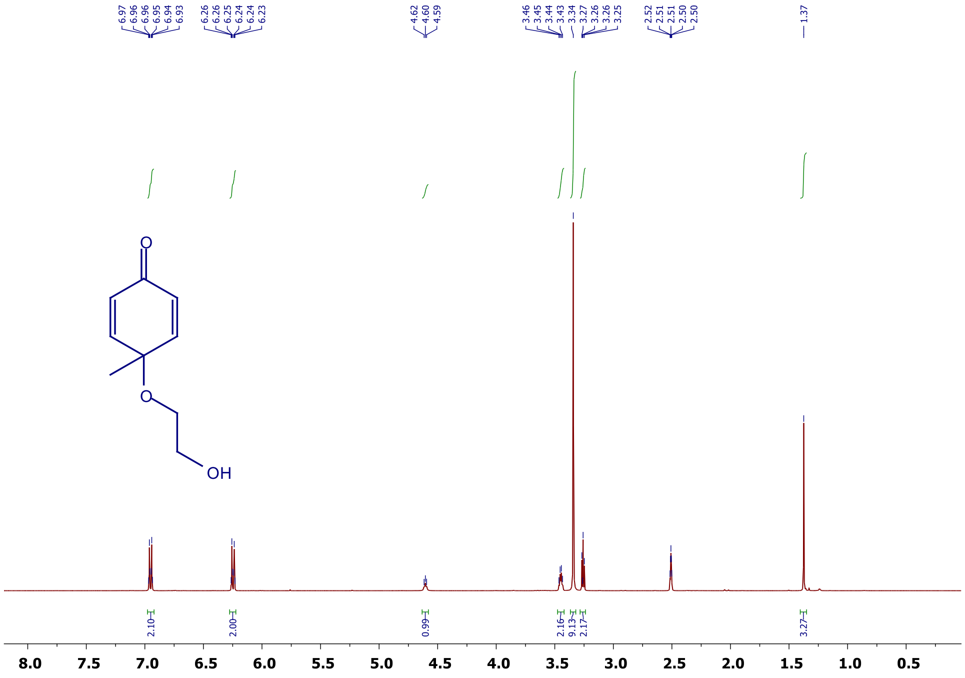

Supplementary Figure 12 ^{1}H NMR (500 MHz, DMSO-d_6) spectrum of 4-(2-hydroxyethoxy)-4-methylcyclohexa-2,5-dien-1-one (**1d**)

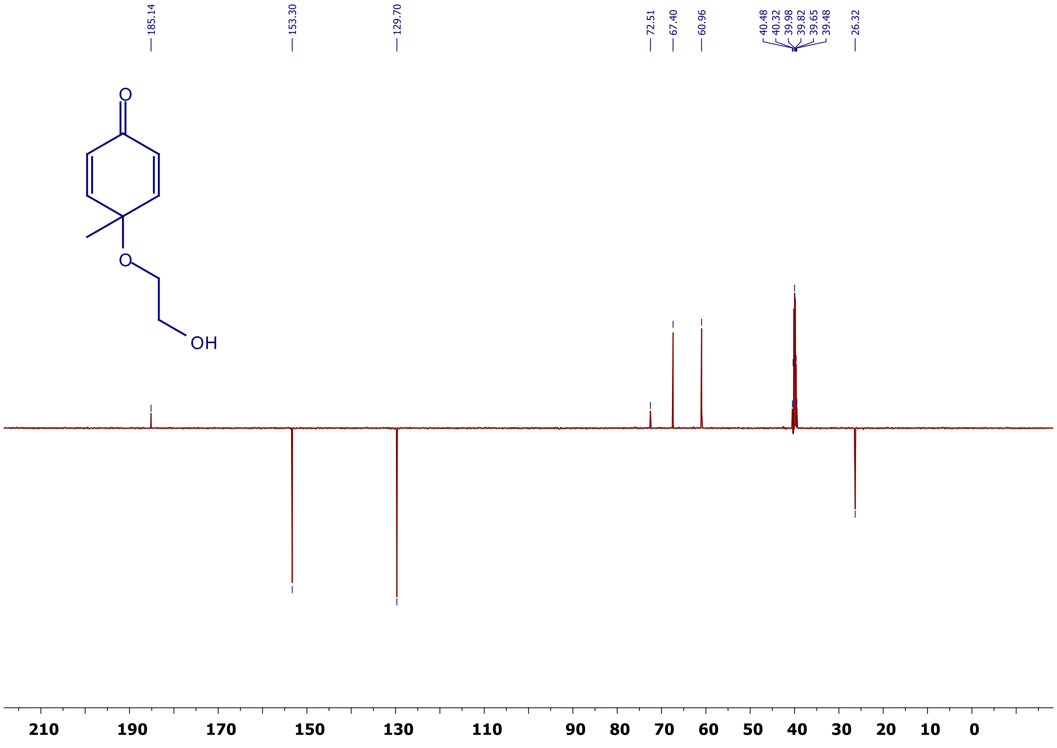

Supplementary Figure 13 APT NMR (125 MHz, DMSO-d_6) spectrum of 4-(2-hydroxyethoxy)-4-methylcyclohexa-2,5-dien-1-one (**1d**)

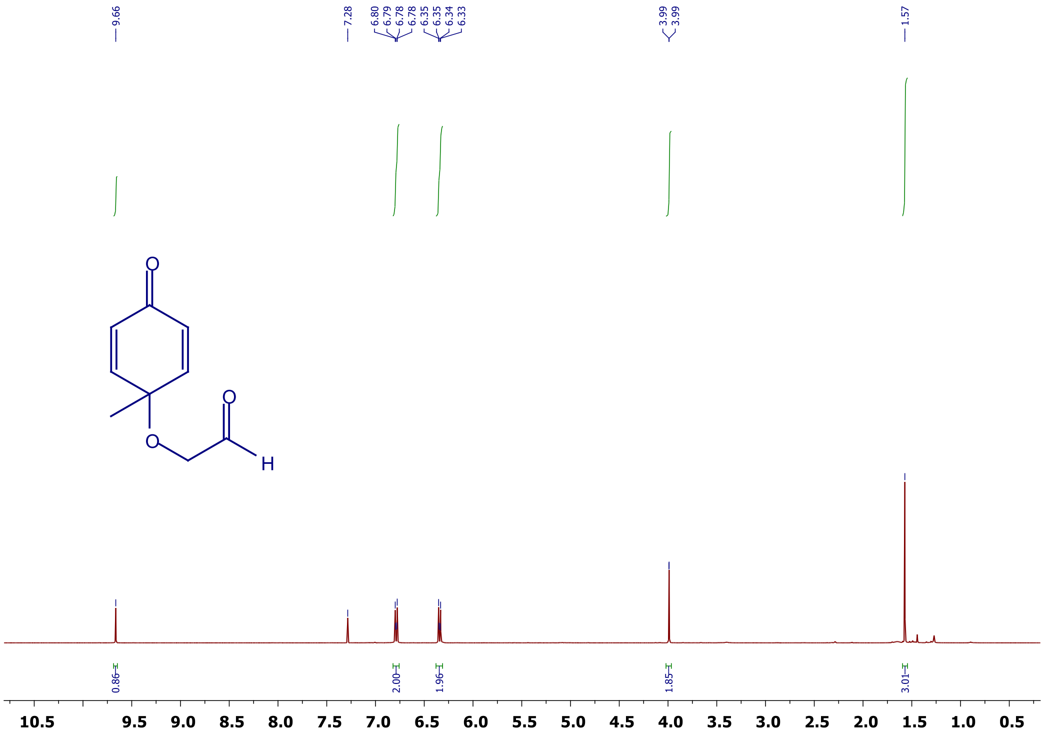

Supplementary Figure 14 ^{1}H NMR (500 MHz, $CDCl_3$) spectrum of 2-((1-methyl-4-oxocyclohexa-2,5-dien-1-yl)oxy)acetaldehyde (**2b**)

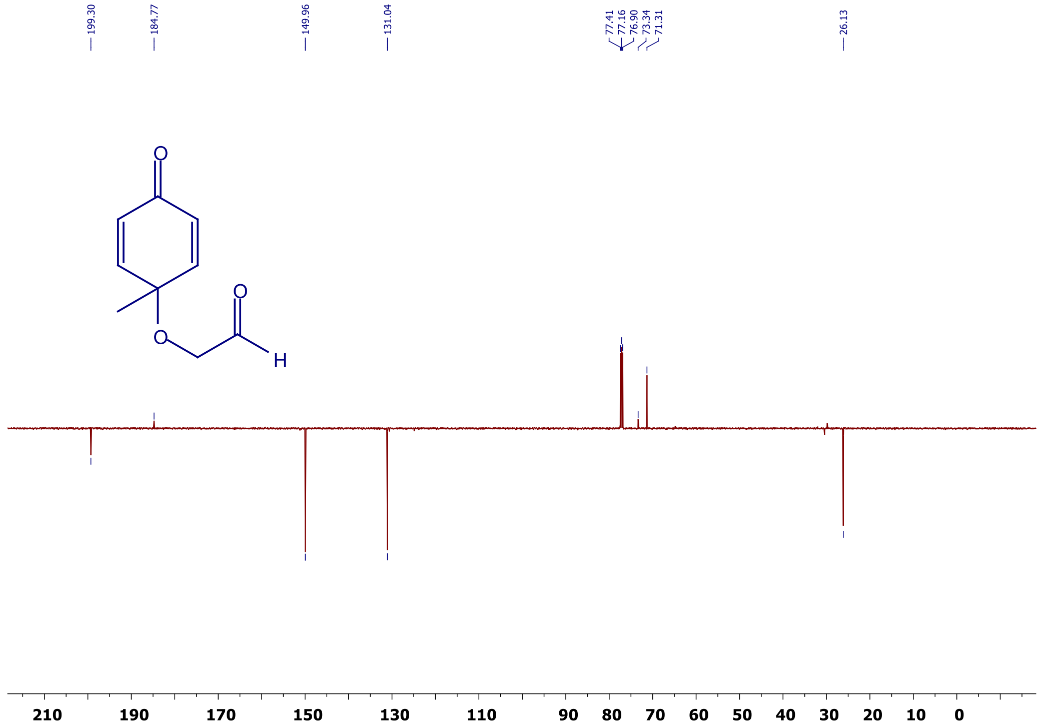

Supplementary Figure 15 APT NMR (125 MHz, $CDCl_3$) spectrum of 2-((1-methyl-4-oxocyclohexa-2,5-dien-1-yl)oxy)acetaldehyde (**2b**)

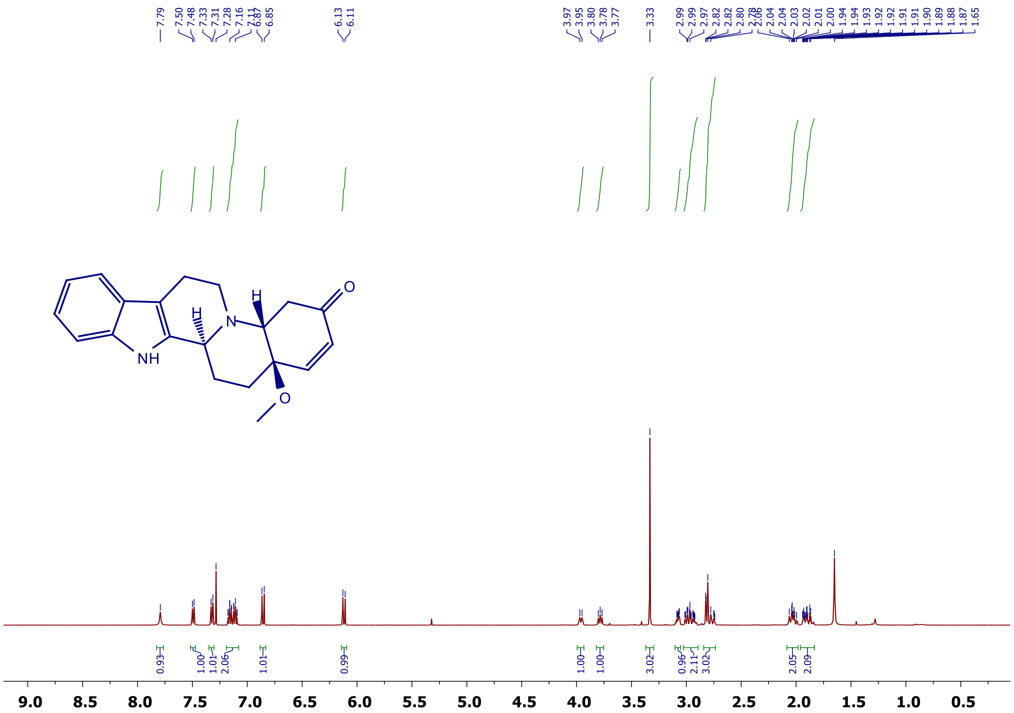

Supplementary Figure 16 ^{1}H NMR (500 MHz, $CDCl_3$) spectrum of (2aR,6aR,14bS)-2a-methoxy-2,2a,6,6a,8,9,14,14b-octahydroindolo[2',3':3,4]pyrido[1,2-a]quinolin-5(1H)-one (**5a**)

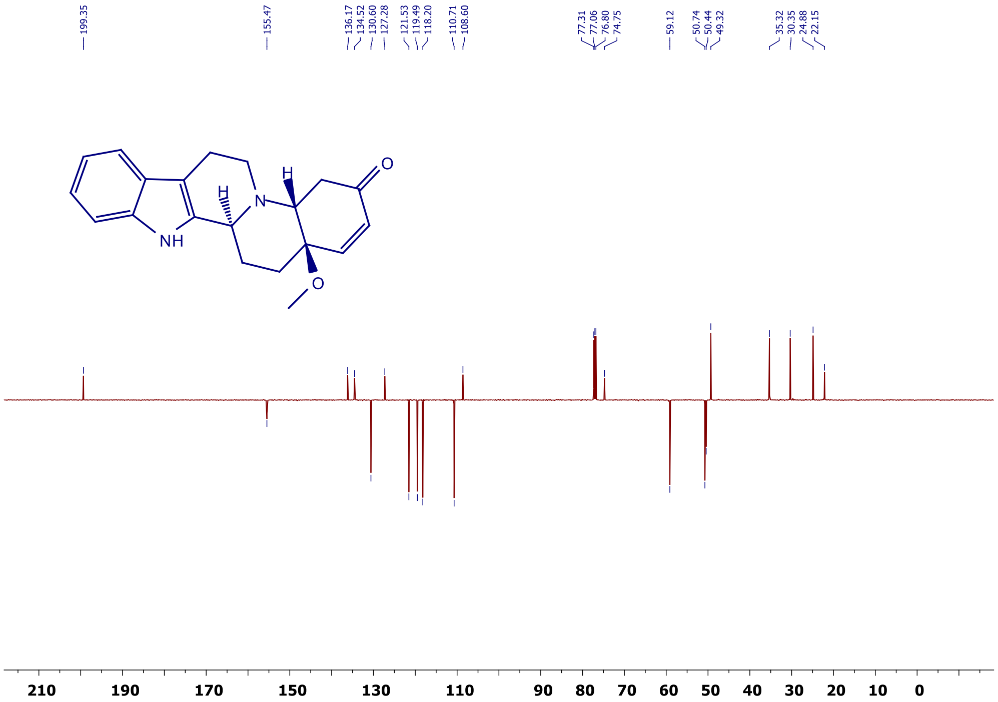

Supplementary Figure 17 APT NMR (125 MHz, $CDCl_3$) spectrum of (2aR,6aR,14bS)-2a-methoxy-2,2a,6,6a,8,9,14,14b-octahydroindolo[2',3':3,4]pyrido[1,2-a]quinolin-5(1H)-one (**5a**)

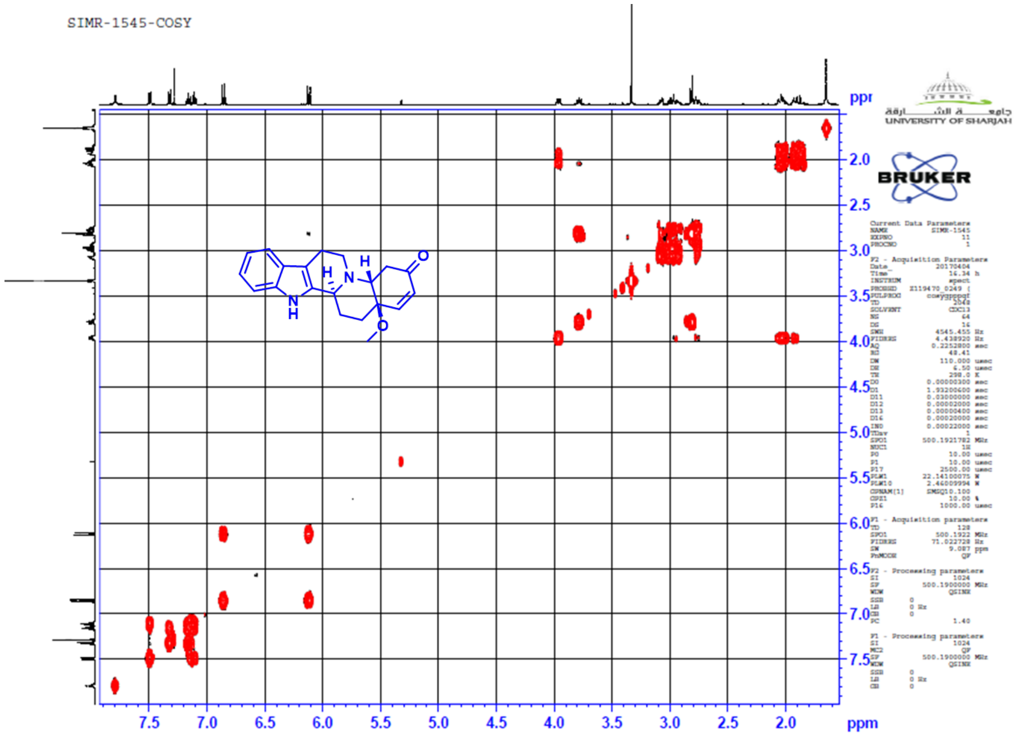

Supplementary Figure 18 COSY (500 MHz, $CDCl_3$) spectrum of (2aR,6aR,14bS)-2a-methoxy-2,2a,6,6a,8,9,14,14b-octahydroindolo[2',3':3,4]pyrido[1,2-a]quinolin-5(1H)-one (**5a**)

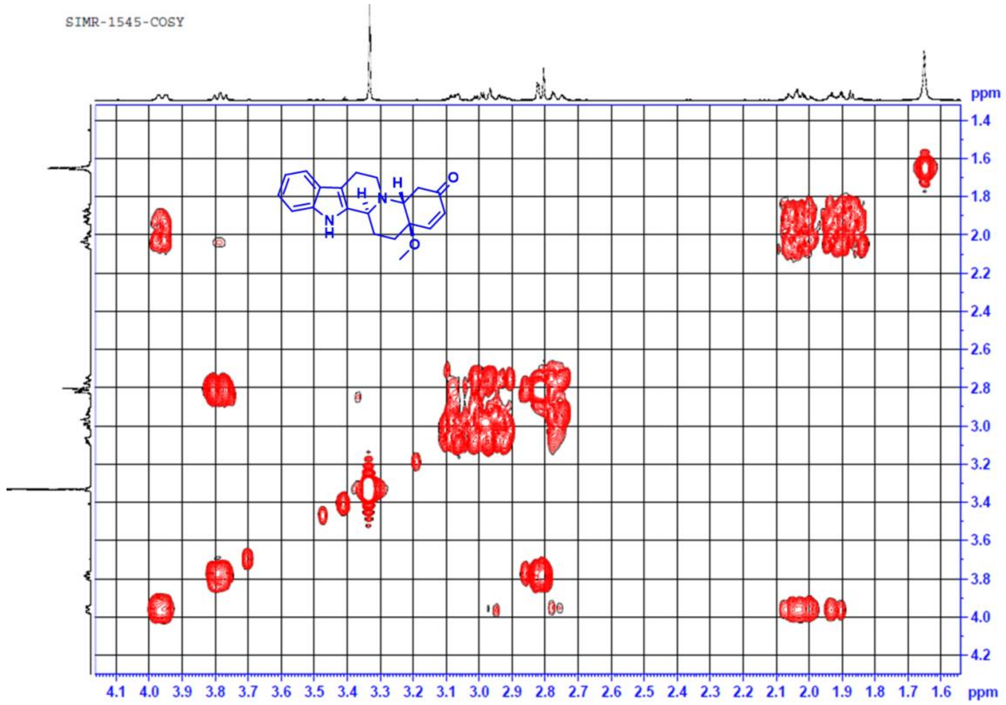

Supplementary Figure 19 COSY (Expansion) spectrum of (2aR,6aR,14bS)-2a-methoxy-2,2a,6,6a,8,9,14,14b-octahydroindolo[2',3':3,4]pyrido[1,2-a]quinolin-5(1H)-one (**5a**)

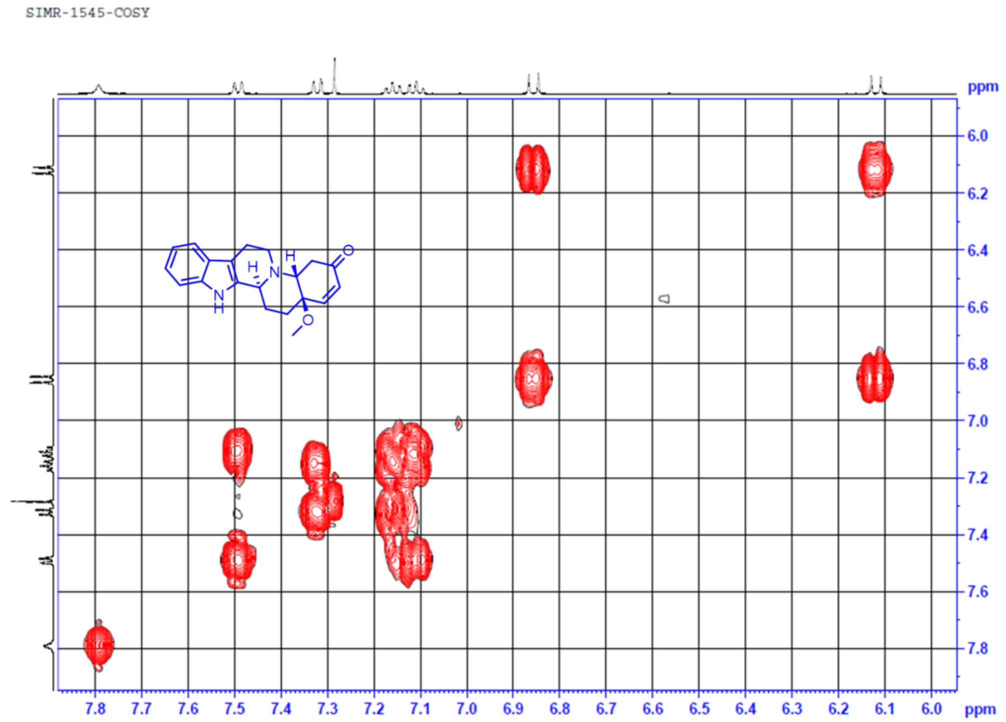

Supplementary Figure 20 COSY (Expansion) spectrum of (2aR,6aR,14bS)-2a-methoxy-2,2a,6,6a,8,9,14,14b-octahydroindolo[2',3':3,4]pyrido[1,2-a]quinolin-5(1H)-one (**5a**)

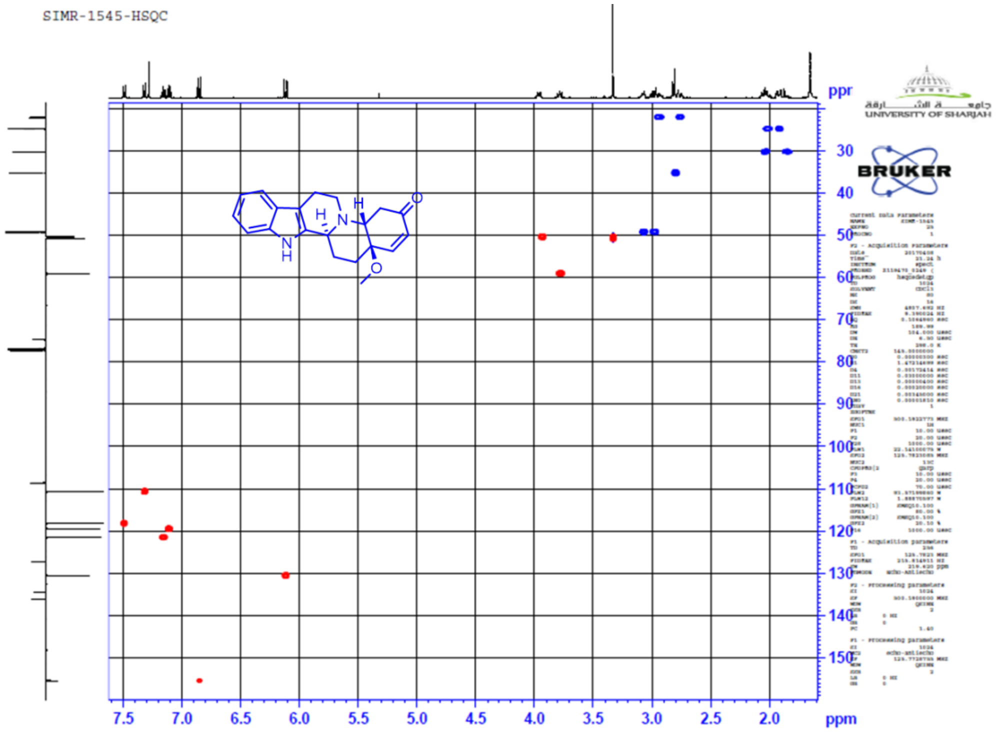

Supplementary Figure 21 HSQC (500 MHz, $CDCl_3$) spectrum of (2aR,6aR,14bS)-2a-methoxy-2,2a,6,6a,8,9,14,14b-octahydroindolo[2',3':3,4]pyrido[1,2-a]quinolin-5(1H)-one (**5a**)

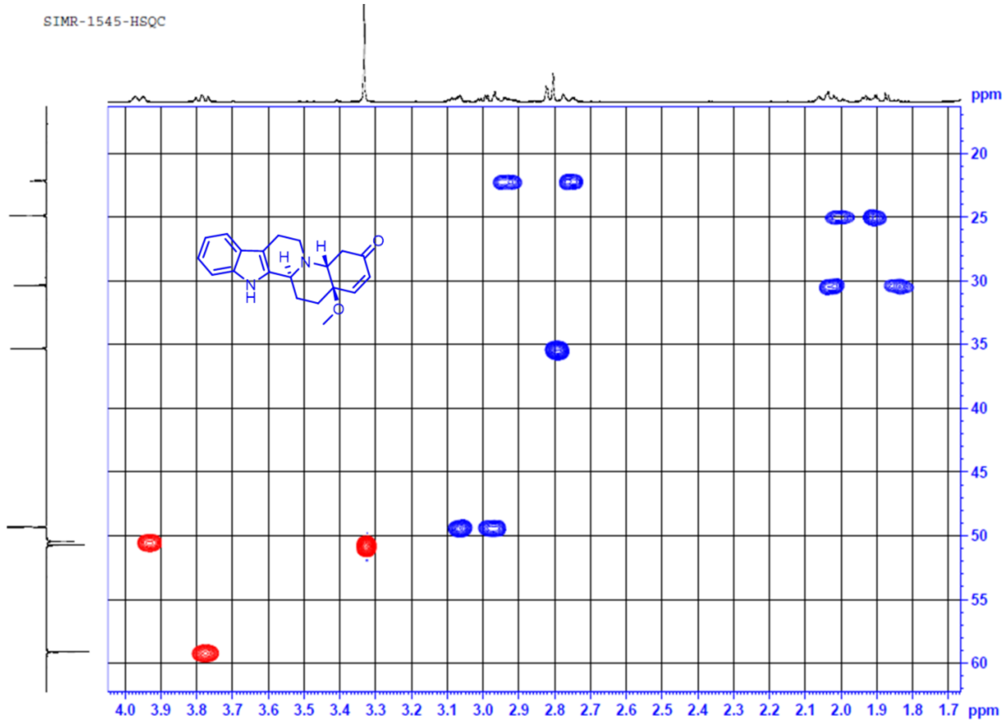

Supplementary Figure 22 HSQC (Expansion) spectrum of (2aR,6aR,14bS)-2a-methoxy-2,2a,6,6a,8,9,14,14b-octahydroindolo[2',3':3,4]pyrido[1,2-a]quinolin-5(1H)-one (**5a**)

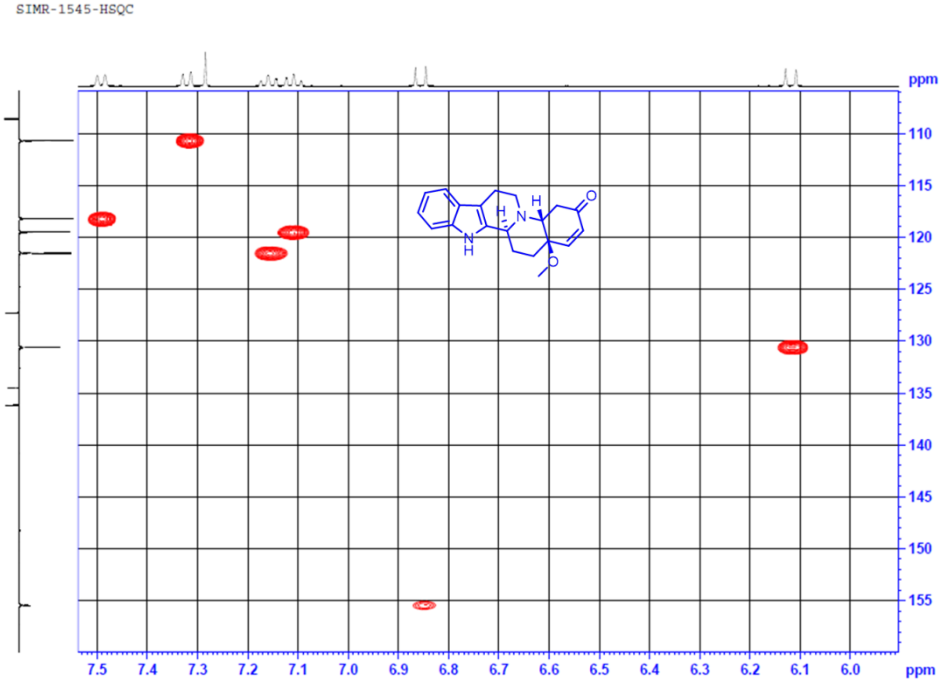

Supplementary Figure 23 HSQC (Expansion) spectrum of (2aR,6aR,14bS)-2a-methoxy-2,2a,6,6a,8,9,14,14b-octahydroindolo[2',3':3,4]pyrido[1,2-a]quinolin-5(1H)-one (**5a**)

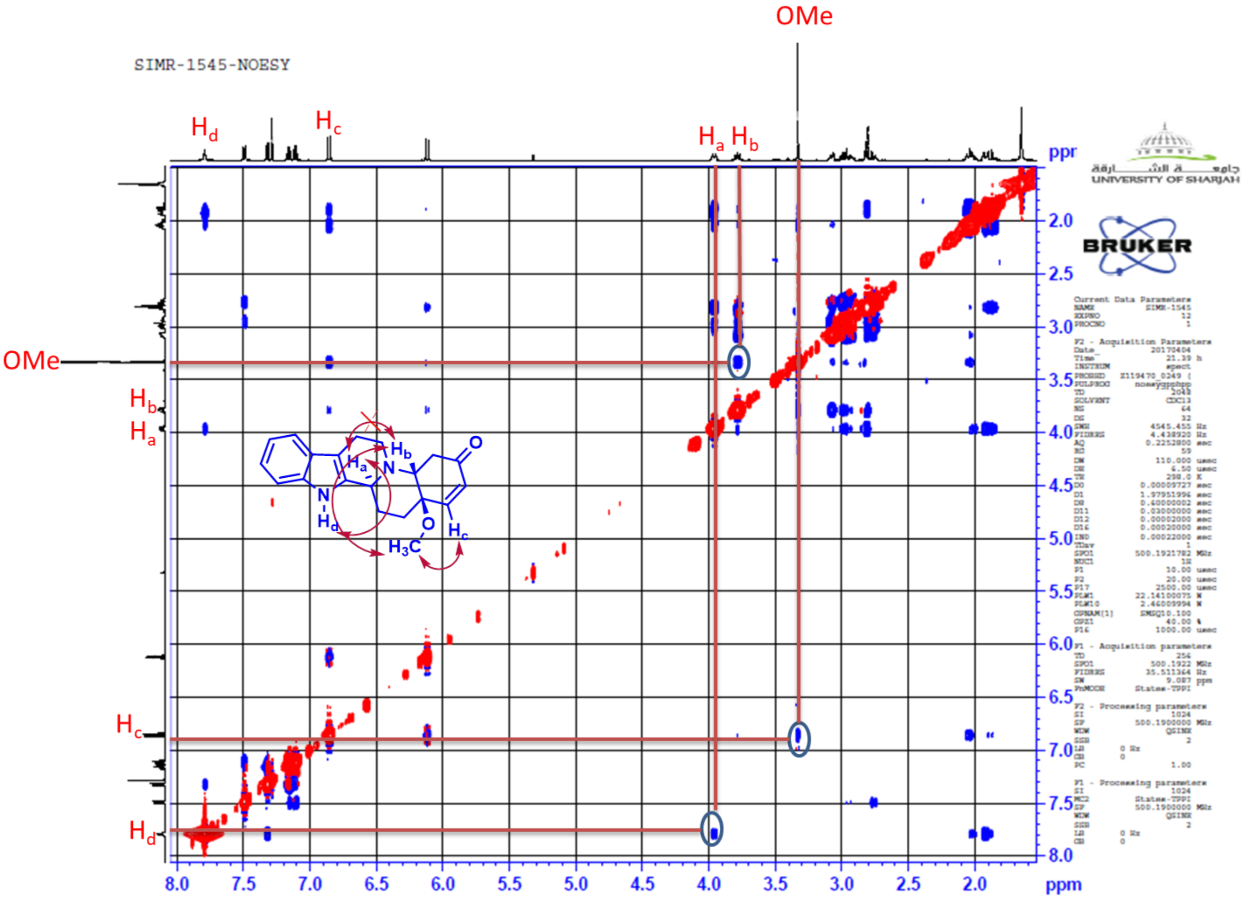

Supplementary Figure 24 NOESY (500 MHz, $CDCl_3$) spectrum of (2aR,6aR,14bS)-2a-methoxy-2,2a,6,6a,8,9,14,14b-octahydroindolo[2',3':3,4]pyrido[1,2-a]quinolin-5(1H)-one (**5a**)

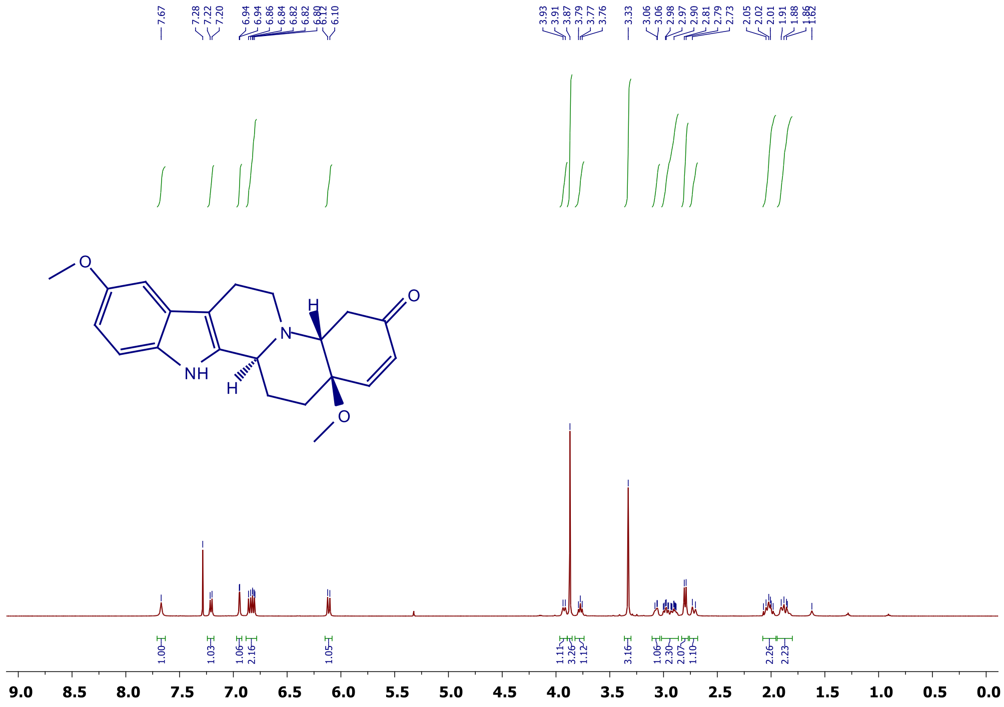

Supplementary Figure 25 ^{1}H NMR (500 MHz, $CDCl_3$) spectrum of (2aR,6aR,14bS)-2a,11-dimethoxy-2,2a,6,6a,8,9,14,14b-octahydroindolo[2',3':3,4]pyrido[1,2-a]quinolin-5(1H)-one (**5b**)

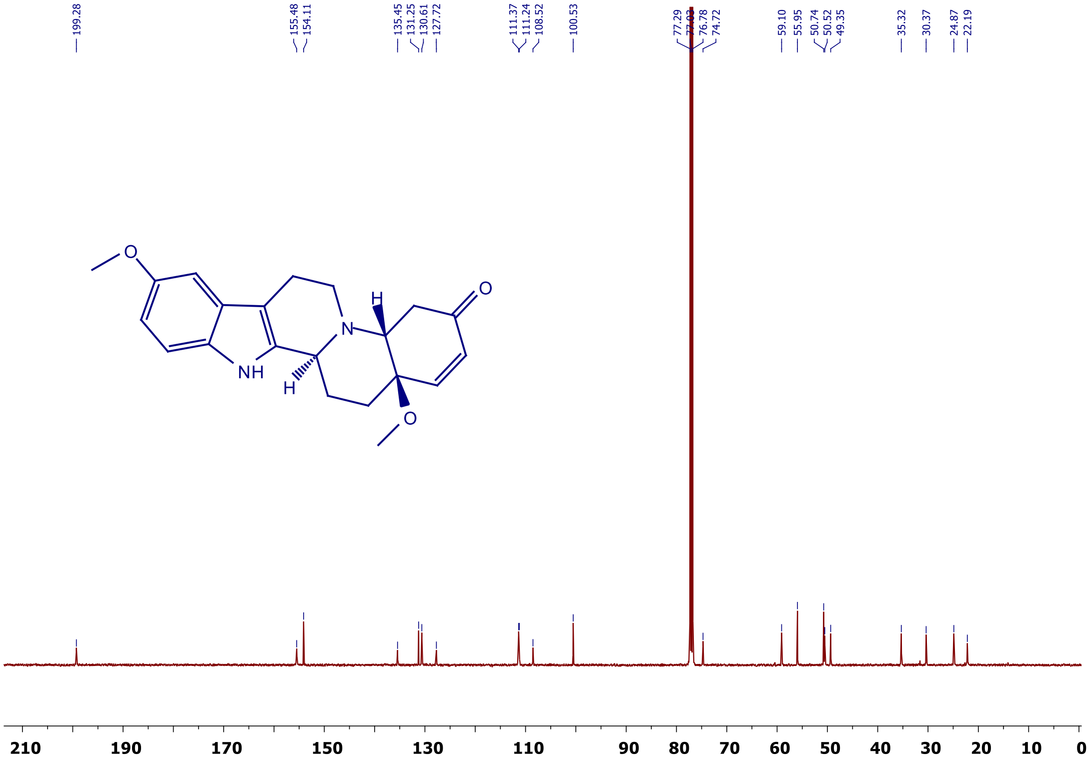

Supplementary Figure 26 ^{13}C NMR (125 MHz, $CDCl_3$) spectrum of (2aR,6aR,14bS)-2a,11-dimethoxy-2,2a,6,6a,8,9,14,14b-octahydroindolo[2',3':3,4]pyrido[1,2-a]quinolin-5(1H)-one (**5b**)

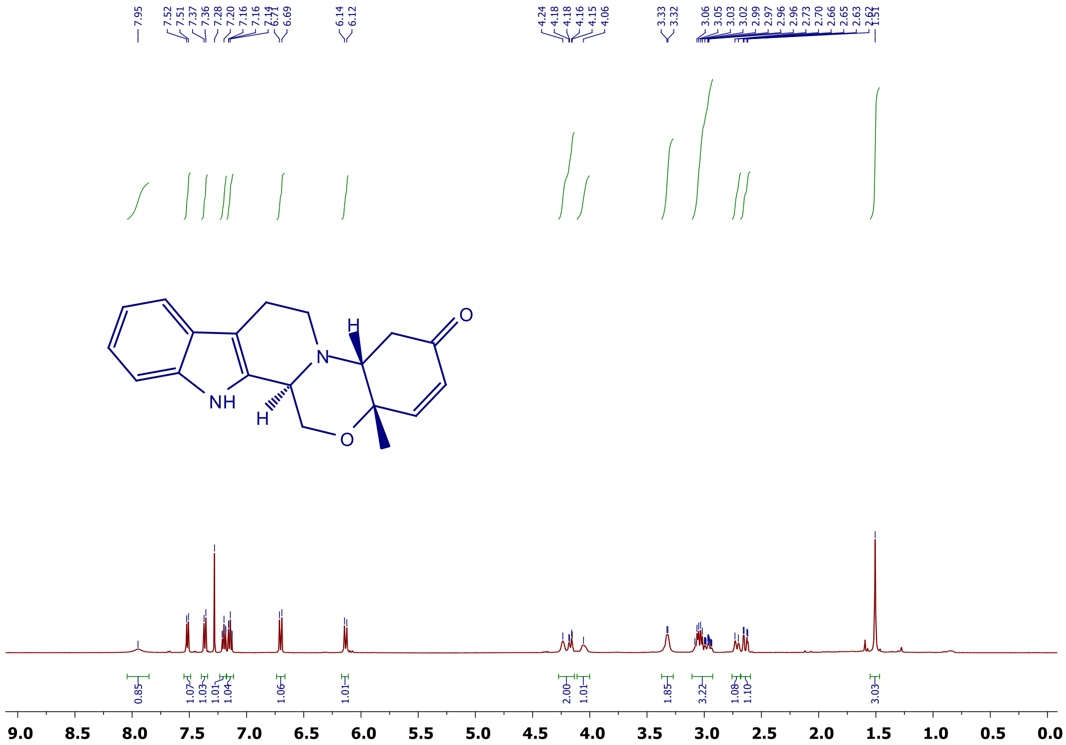

Supplementary Figure 27 ^{1}H NMR (500 MHz, $CDCl_3$) spectrum of (2aS,6aR,14bR)-2a-methyl-6,6a,8,9,14,14b-hexahydro-1H-benzo[5',6'][1,4]oxazino[4',3':1,2]pyrido[3,4-b]indol-5(2aH)-one (**5c**)

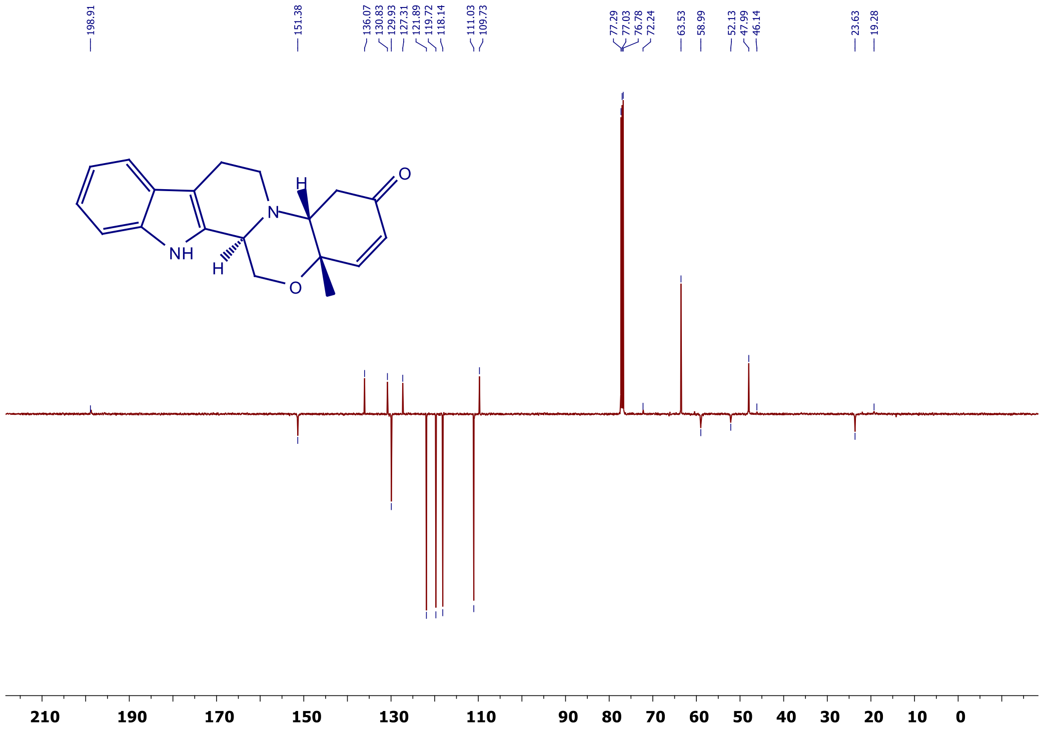

Supplementary Figure 28 APT NMR (125 MHz, $CDCl_3$) spectrum of (2aS,6aR,14bR)-2a-methyl-6,6a,8,9,14,14b-hexahydro-1H-benzo[5',6'][1,4]oxazino[4',3':1,2]pyrido[3,4-b]indol-5(2aH)-one (**5c**)

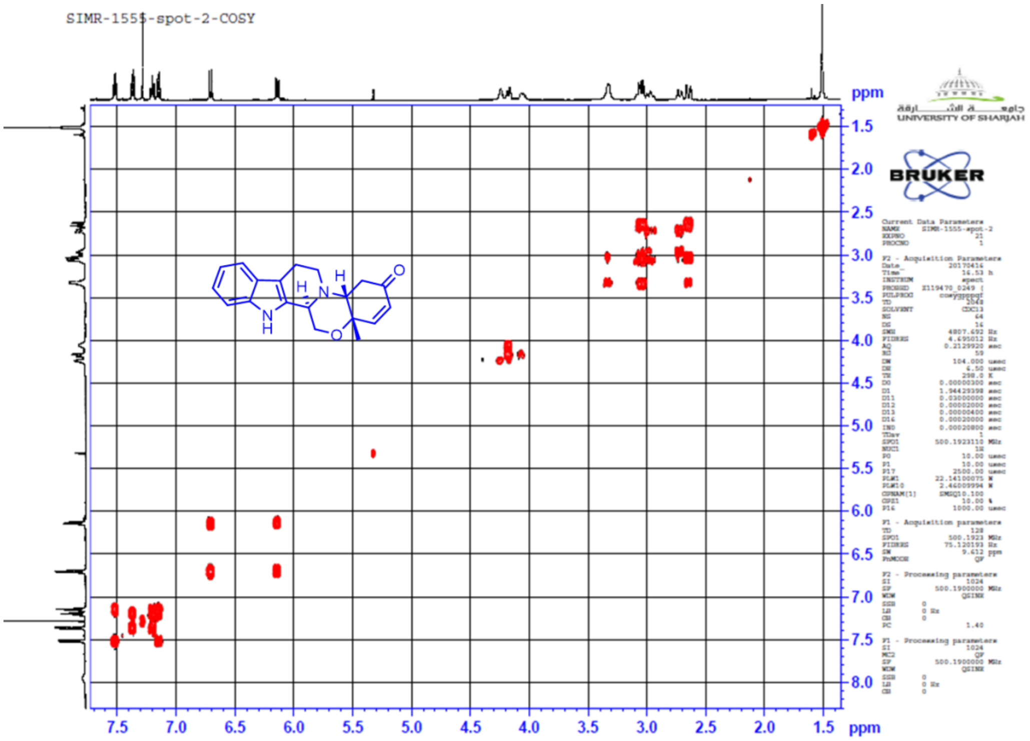

Supplementary Figure 29 COSY (500 MHz, $CDCl_3$) spectrum of (2aS,6aR,14bR)-2a-methyl-6,6a,8,9,14,14b-hexahydro-1H-benzo[5',6'][1,4]oxazino[4',3':1,2]pyrido[3,4-b]indol-5(2aH)-one (**5c**)

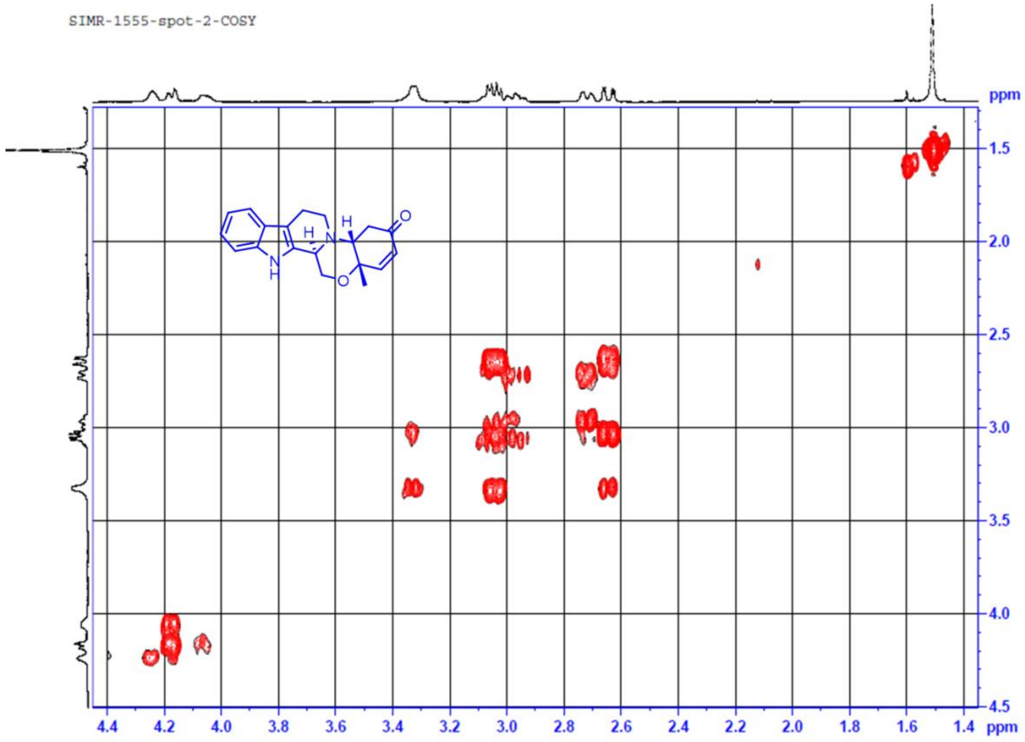

Supplementary Figure 30 COSY (Expansion) spectrum of (2aS,6aR,14bR)-2a-methyl-6,6a,8,9,14,14b-hexahydro-1H-benzo[5',6'][1,4]oxazino[4',3':1,2]pyrido[3,4-b]indol-5(2aH)-one (**5c**)

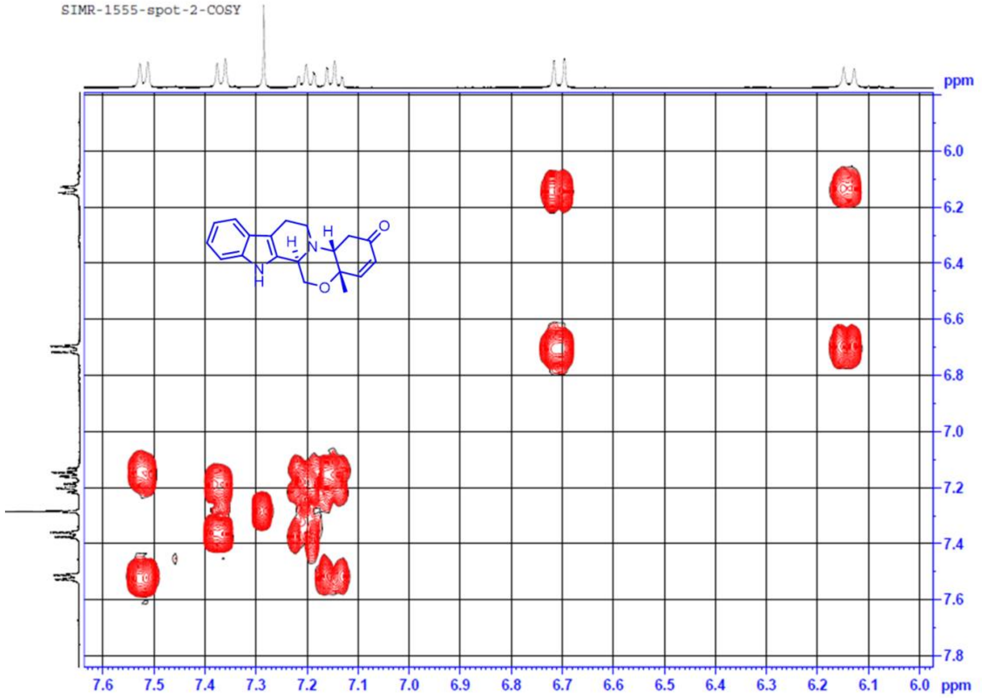

Supplementary Figure 31 COSY (Expansion) spectrum of (2aS,6aR,14bR)-2a-methyl-6,6a,8,9,14,14b-hexahydro-1H-benzo[5',6'][1,4]oxazino[4',3':1,2]pyrido[3,4-b]indol-5(2aH)-one (**5c**)

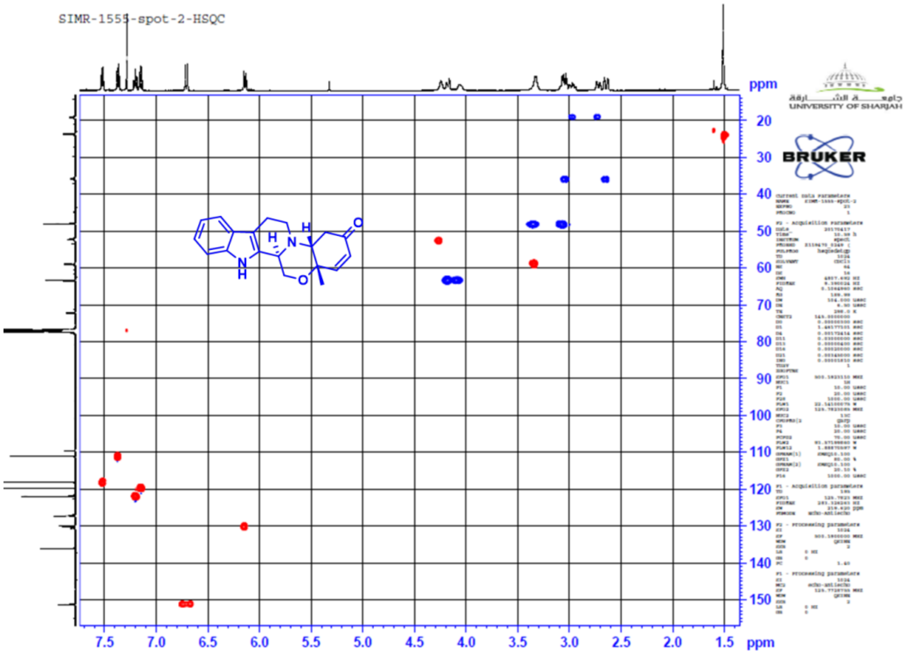

Supplementary Figure 32 HSQC (500 MHz, $CDCl_3$) spectrum of (2aS,6aR,14bR)-2a-methyl-6,6a,8,9,14,14b-hexahydro-1H-benzo[5',6'][1,4]oxazino[4',3':1,2]pyrido[3,4-b]indol-5(2aH)-one (**5c**)

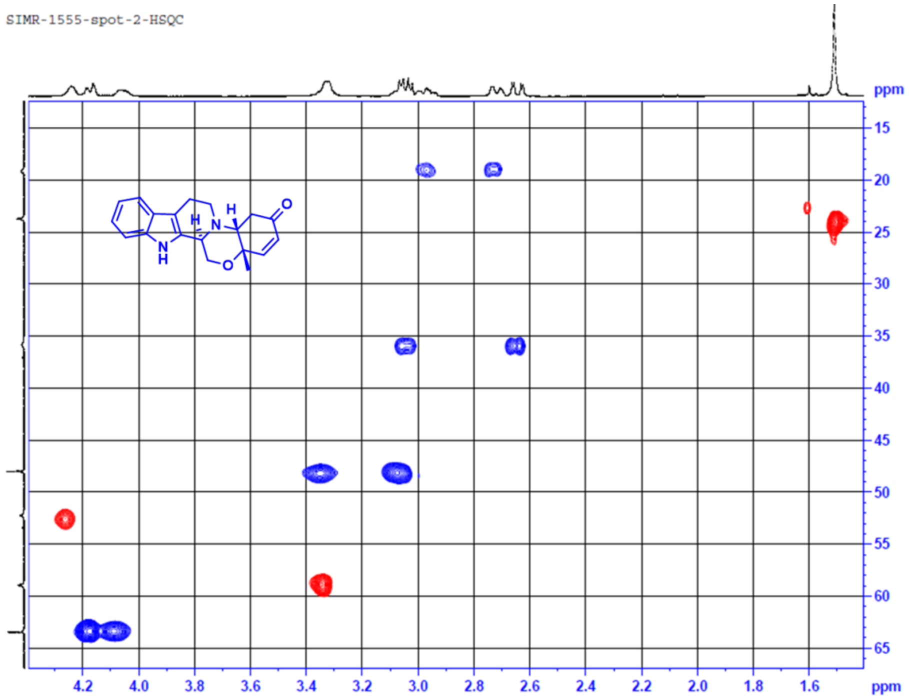

Supplementary Figure 33 HSQC (Expansion) spectrum of (2aS,6aR,14bR)-2a-methyl-6,6a,8,9,14,14b-hexahydro-1H-benzo[5',6'][1,4]oxazino[4',3':1,2]pyrido[3,4-b]indol-5(2aH)-one (**5c**)

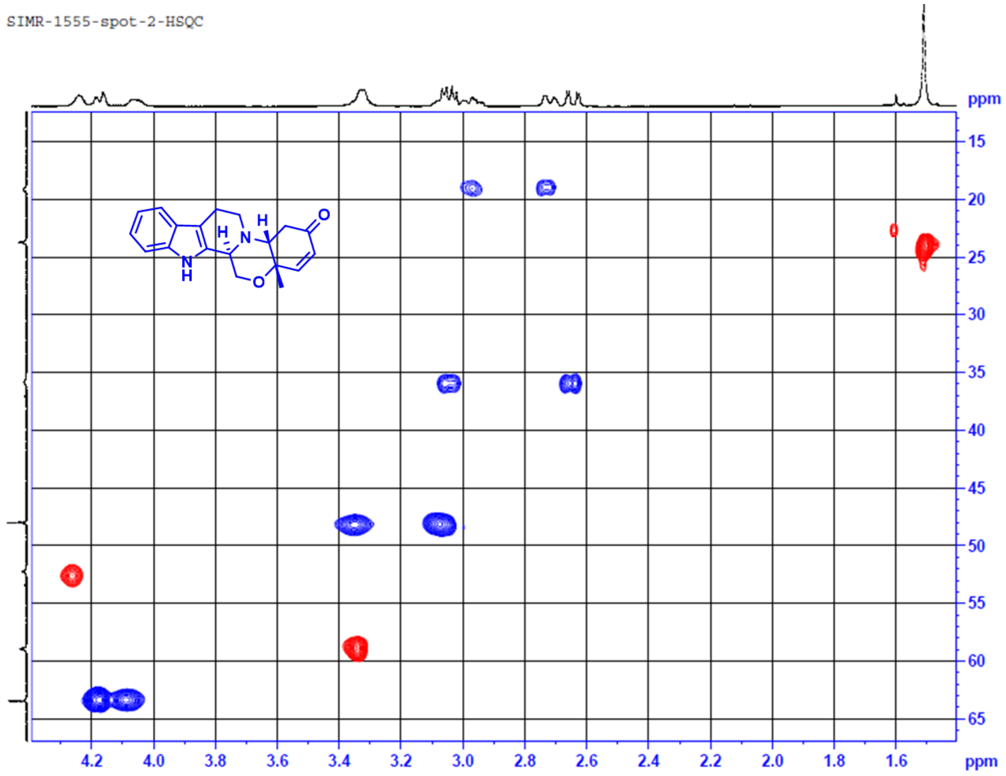

Supplementary Figure 34 HSQC (Expansion) spectrum of (2aS,6aR,14bR)-2a-methyl-6,6a,8,9,14,14b-hexahydro-1H-benzo[5',6'][1,4]oxazino[4',3':1,2]pyrido[3,4-b]indol-5(2aH)-one (**5c**)

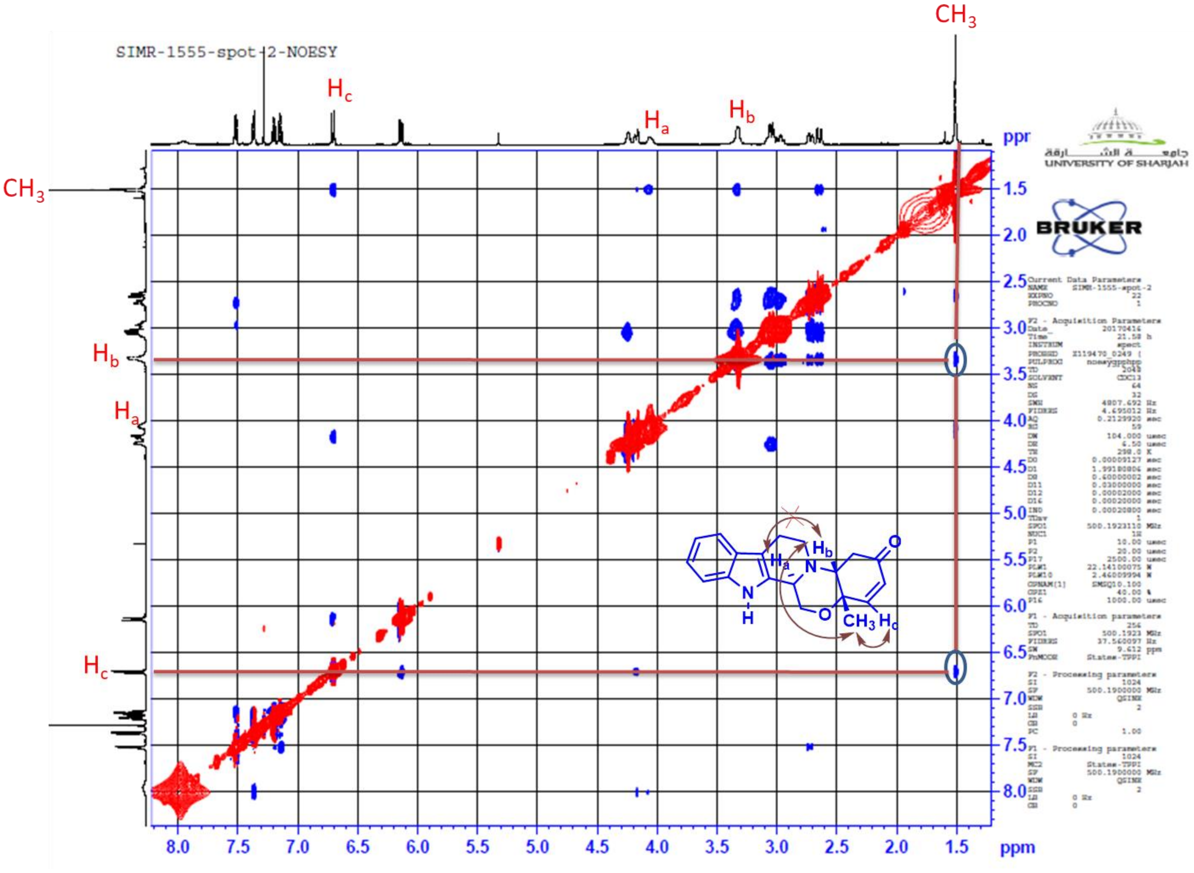

Supplementary Figure 35 NOESY (500 MHz, $CDCl_3$) spectrum of (2aS,6aR,14bR)-2a-methyl-6,6a,8,9,14,14b-hexahydro-1H-benzo[5',6'][1,4]oxazino[4',3':1,2]pyrido[3,4-b]indol-5(2aH)-one (**5c**)

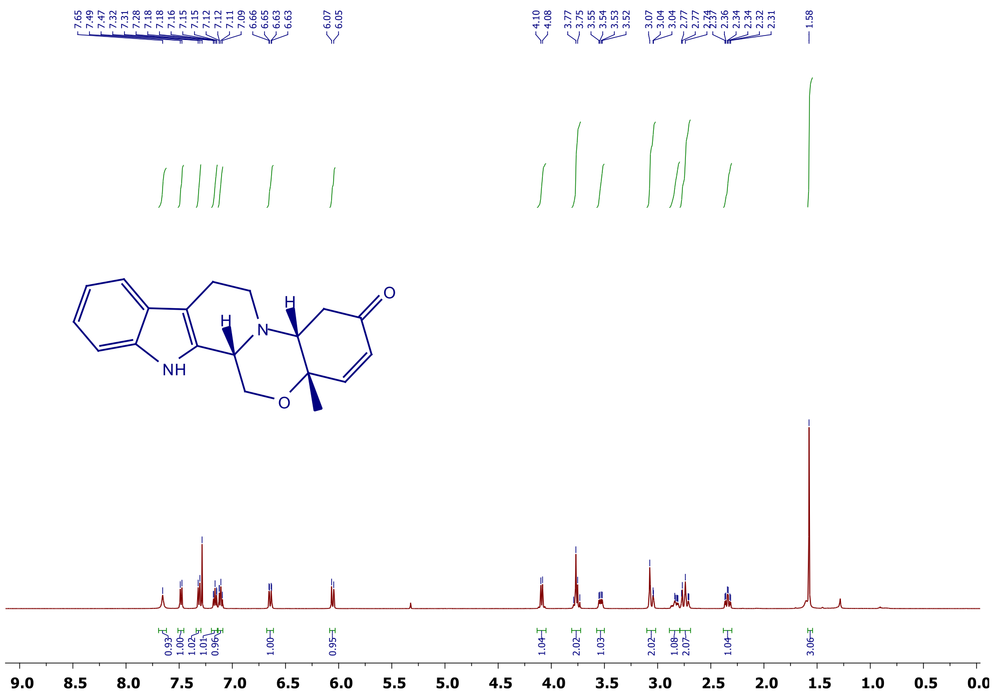

Supplementary Figure 36 ^{1}H NMR (500 MHz, $CDCl_3$) spectrum of (2aS,6aR,14bS)-2a-methyl-6,6a,8,9,14,14b-hexahydro-1H-benzo[5',6'][1,4]oxazino[4',3':1,2]pyrido[3,4-b]indol-5(2aH)-one (**6c**)

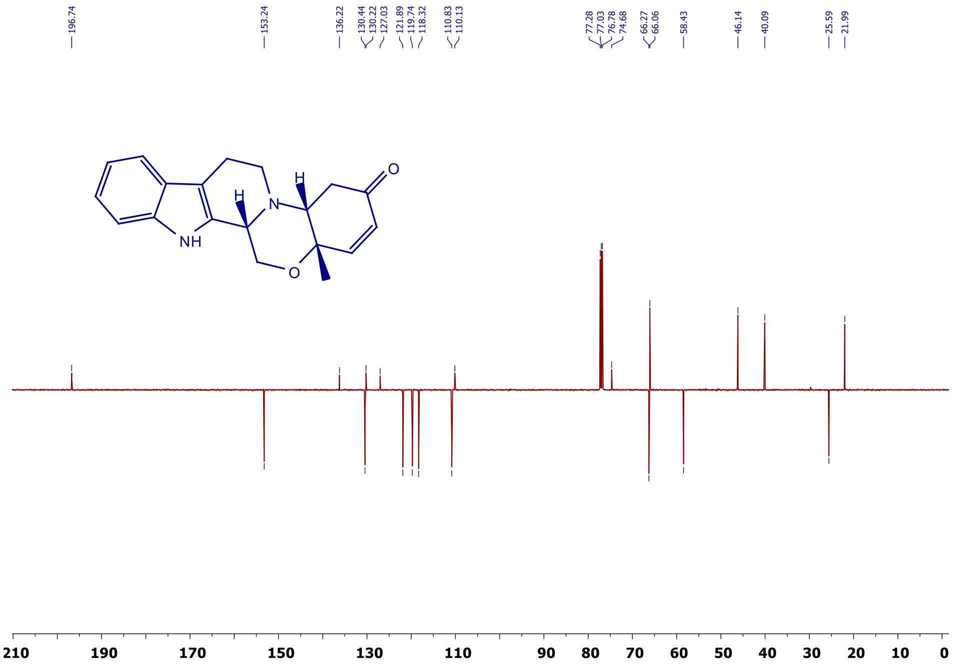

Supplementary Figure 37 APT NMR (125 MHz, $CDCl_3$) spectrum of (2aS,6aR,14bS)-2a-methyl-6,6a,8,9,14,14b-hexahydro-1H-benzo[5',6'][1,4]oxazino[4',3':1,2]pyrido[3,4-b]indol-5(2aH)-one (**6c**)

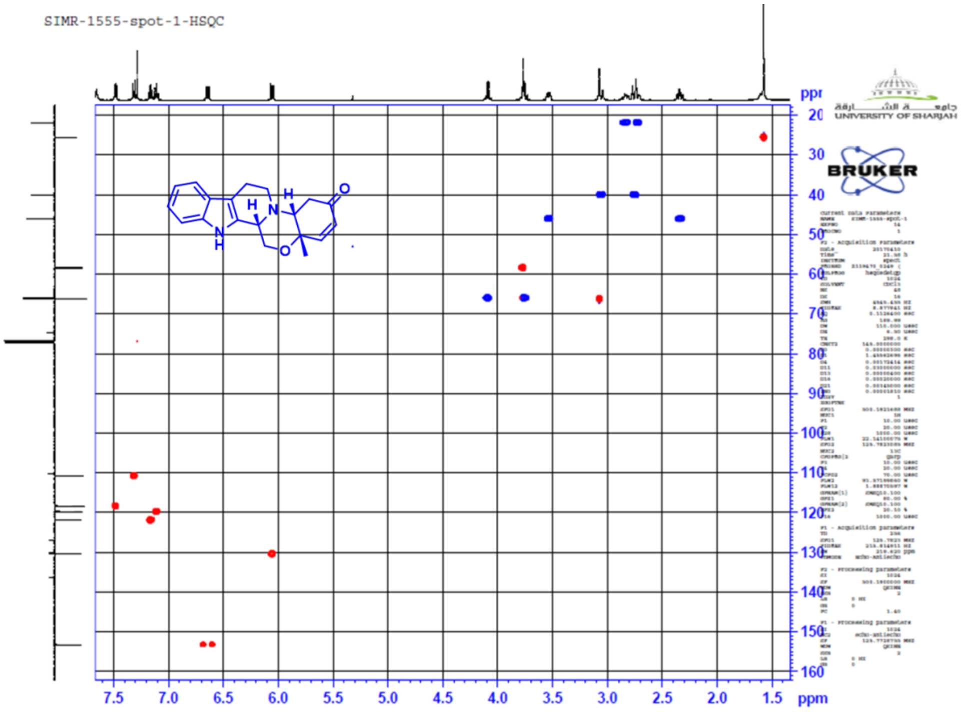

Supplementary Figure 38 HSQC (500 MHz, $CDCl_3$) spectrum of (2aS,6aR,14bS)-2a-methyl-6,6a,8,9,14,14b-hexahydro-1H-benzo[5',6'][1,4]oxazino[4',3':1,2]pyrido[3,4-b]indol-5(2aH)-one (**6c**)

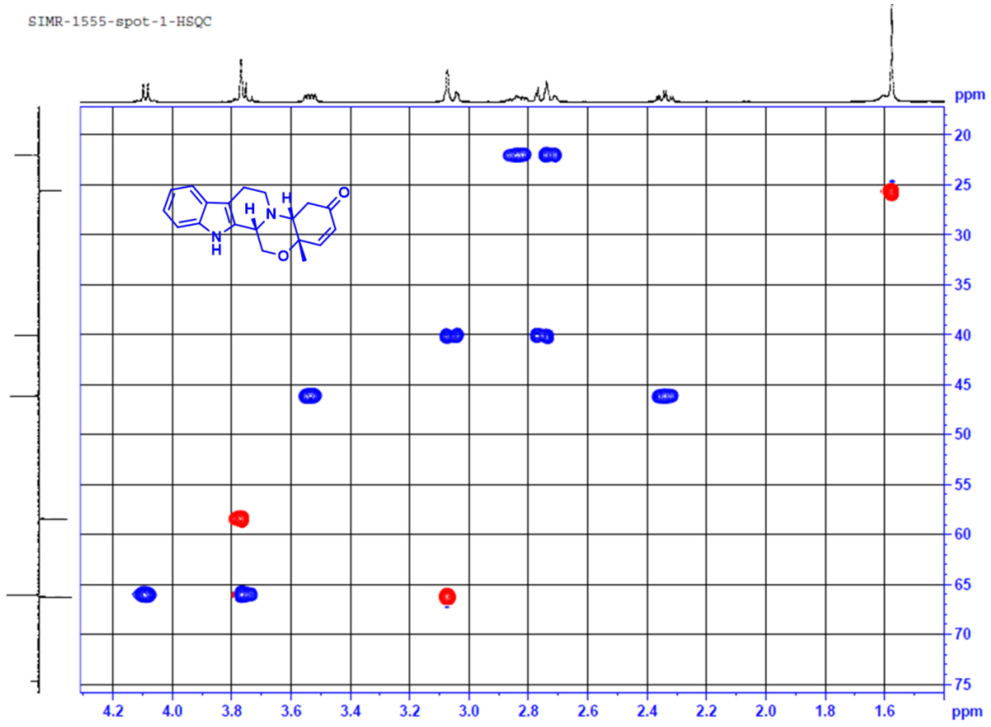

Supplementary Figure 39 HSQC (Expansion) spectrum of (2aS,6aR,14bS)-2a-methyl-6,6a,8,9,14,14b-hexahydro-1H-benzo[5',6'][1,4]oxazino[4',3':1,2]pyrido[3,4-b]indol-5(2aH)-one (**6c**)

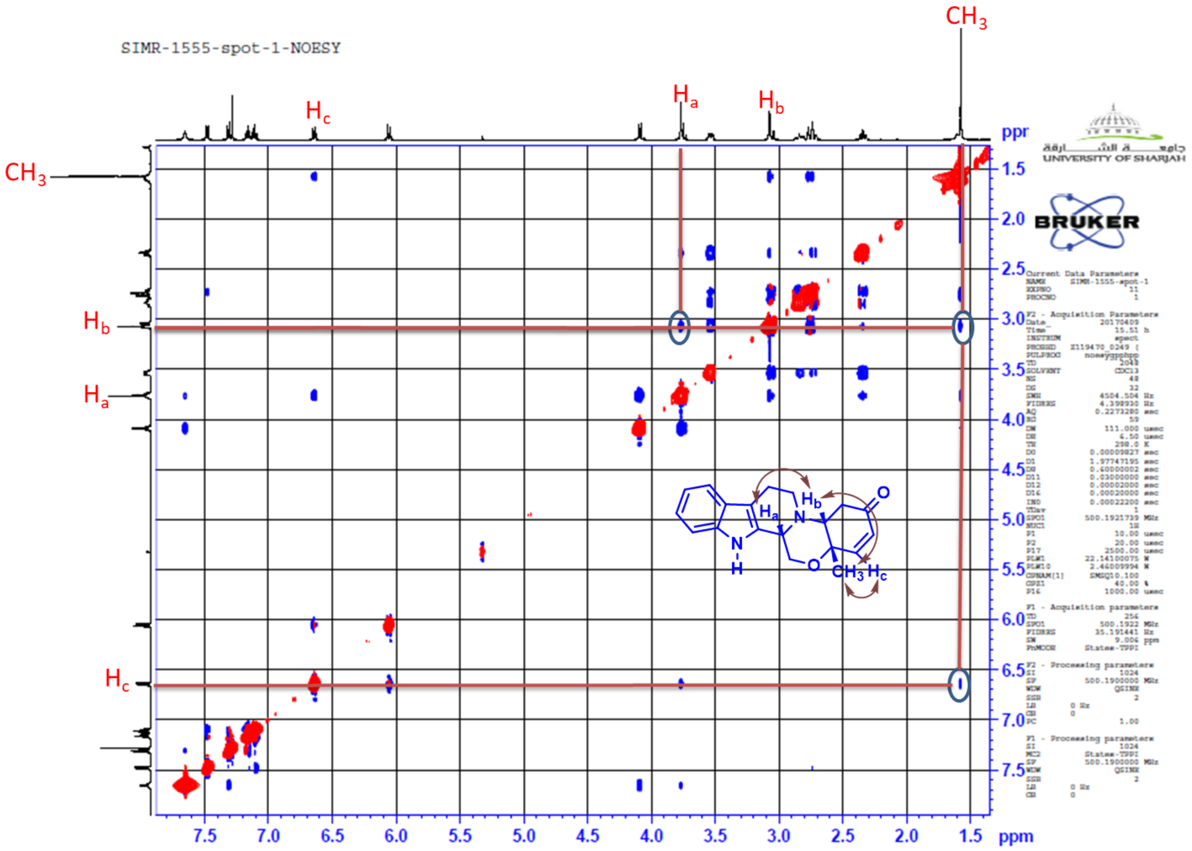

Supplementary Figure 40 NOESY (500 MHz, $CDCl_3$) spectrum of (2aS,6aR,14bS)-2a-methyl-6,6a,8,9,14,14b-hexahydro-1H-benzo[5',6'][1,4]oxazino[4',3':1,2]pyrido[3,4-b]indol-5(2aH)-one (**6c**)

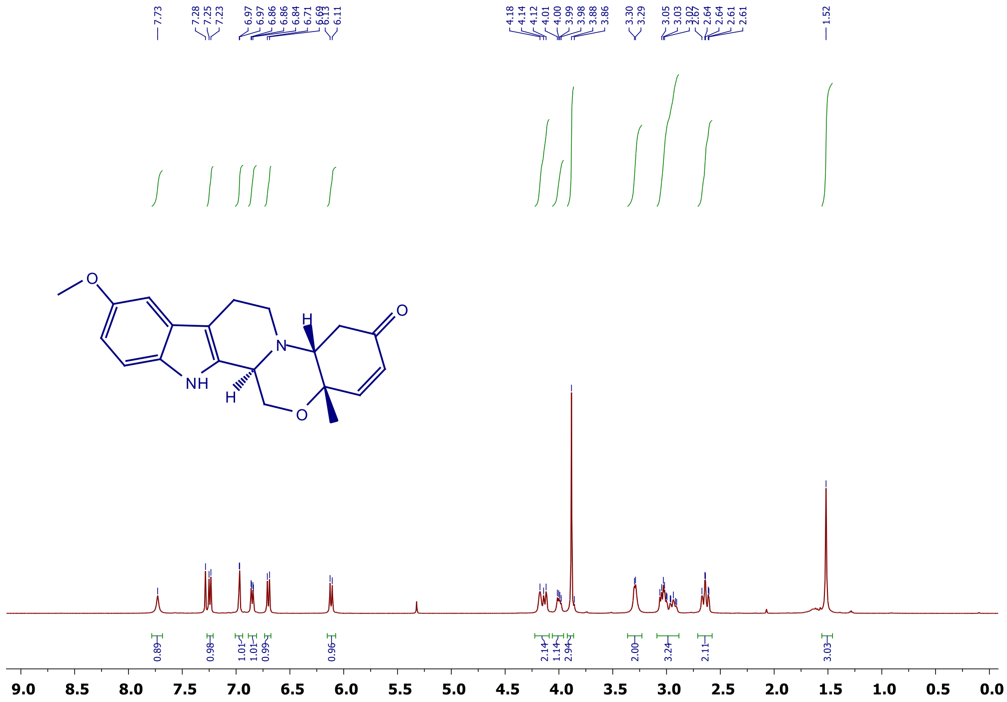

Supplementary Figure 41 ^{1}H NMR (500 MHz, $CDCl_3$) spectrum of (2aS,6aR,14bR)-11-methoxy-2a-methyl-6,6a,8,9,14,14b-hexahydro-1H-benzo[5',6'][1,4]oxazino[4',3':1,2]pyrido[3,4-b]indol-5(2aH)-one (**5d**)

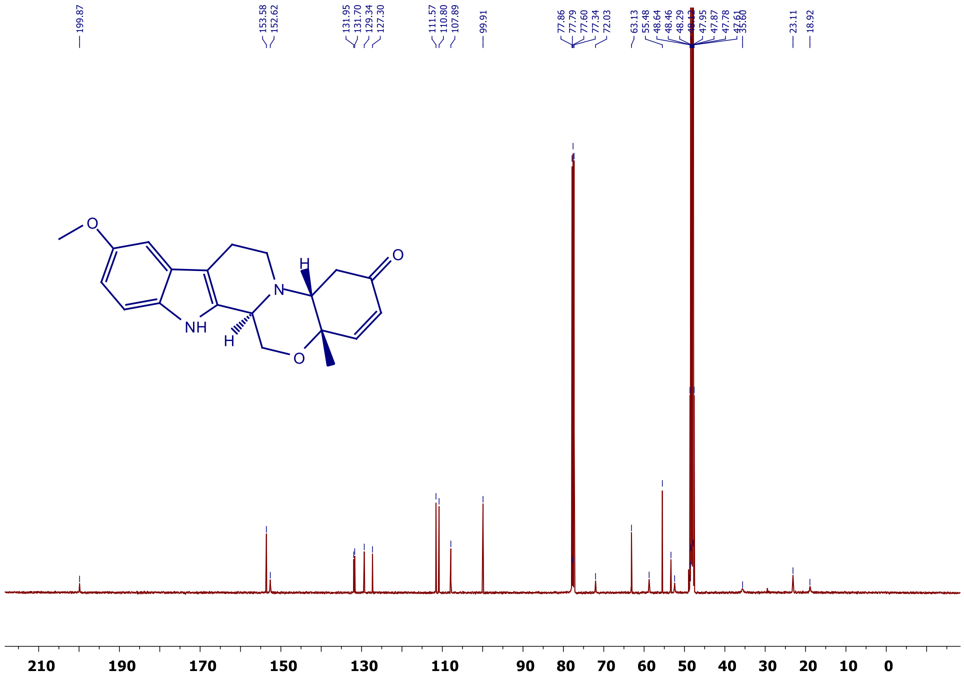

Supplementary Figure 42 ^{13}C NMR (125 MHz, $CDCl_3$) spectrum of (2aS,6aR,14bR)-11-methoxy-2a-methyl-6,6a,8,9,14,14b-hexahydro-1H-benzo[5',6'][1,4]oxazino[4',3':1,2]pyrido[3,4-b]indol-5(2aH)-one (**5d**)

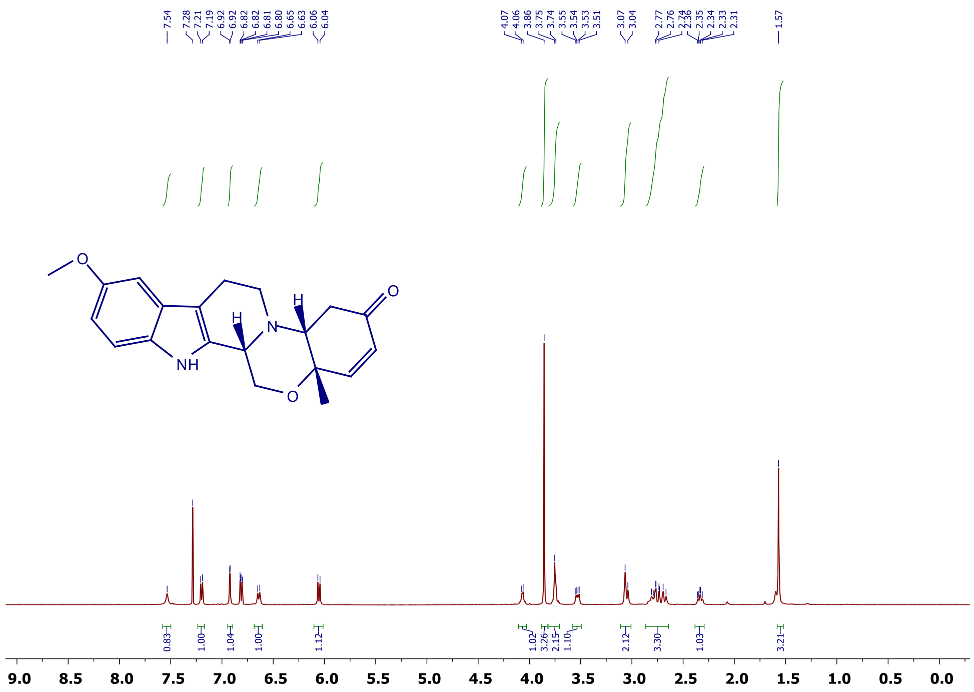

Supplementary Figure 43 ^{1}H NMR (500 MHz, $CDCl_3$) spectrum of (2aS,6aR,14bS)-11-methoxy-2a-methyl-6,6a,8,9,14,14b-hexahydro-1H-benzo[5',6'][1,4]oxazino[4',3':1,2]pyrido[3,4-b]indol-5(2aH)-one (**6d**)

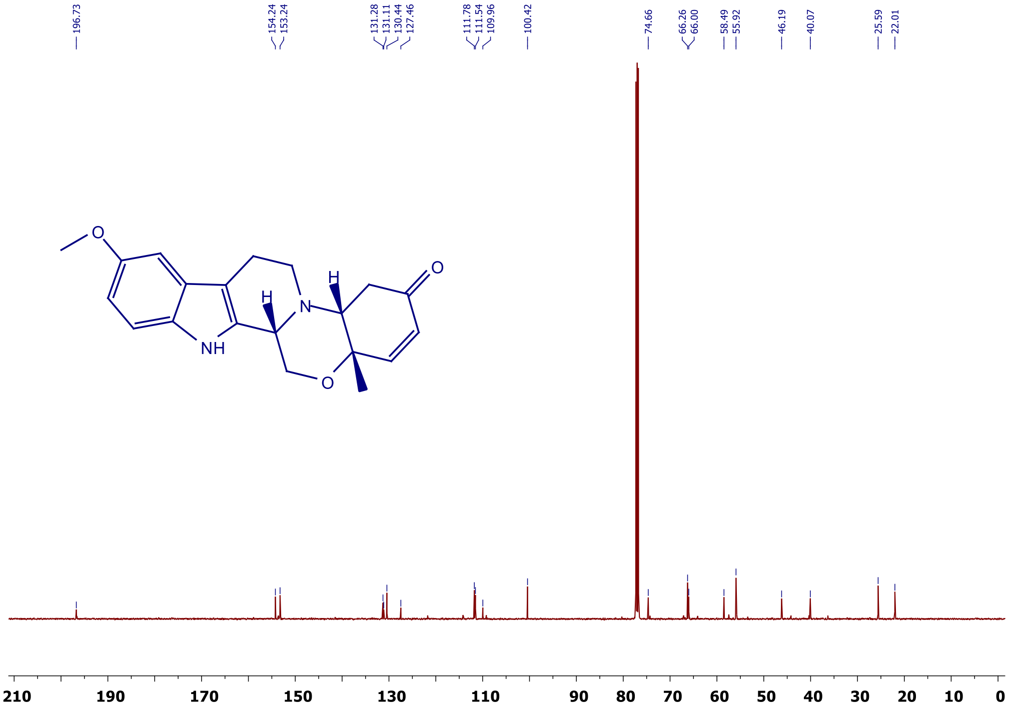

Supplementary Figure 44 ^{13}C NMR (125 MHz, $CDCl_3$) spectrum of (2aS,6aR,14bS)-11-methoxy-2a-methyl-6,6a,8,9,14,14b-hexahydro-1H-benzo[5',6'][1,4]oxazino[4',3':1,2]pyrido[3,4-b]indol-5(2aH)-one (**6d**)

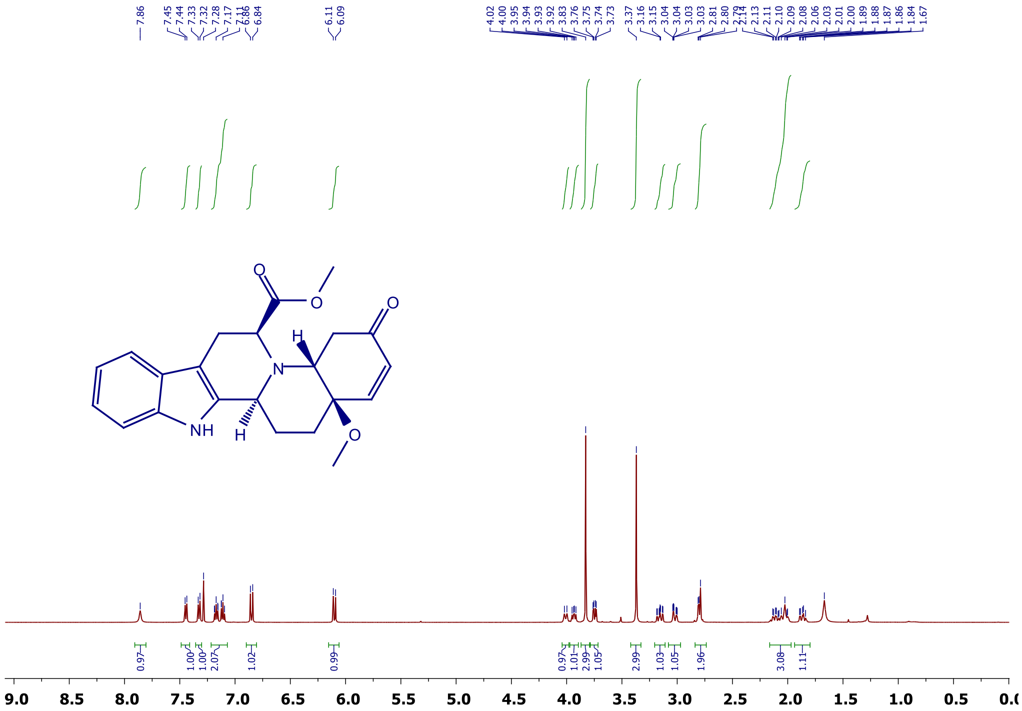

Supplementary Figure 45 ^{1}H NMR (500 MHz, $CDCl_3$) spectrum of methyl (2aR,6aR,8S,14bS)-2a-methoxy-5-oxo-1,2,2a,5,6,6a,8,9,14,14b-decahydroindolo[2',3':3,4]pyrido[1,2-a]quinoline-8-carboxylate (**8a**)

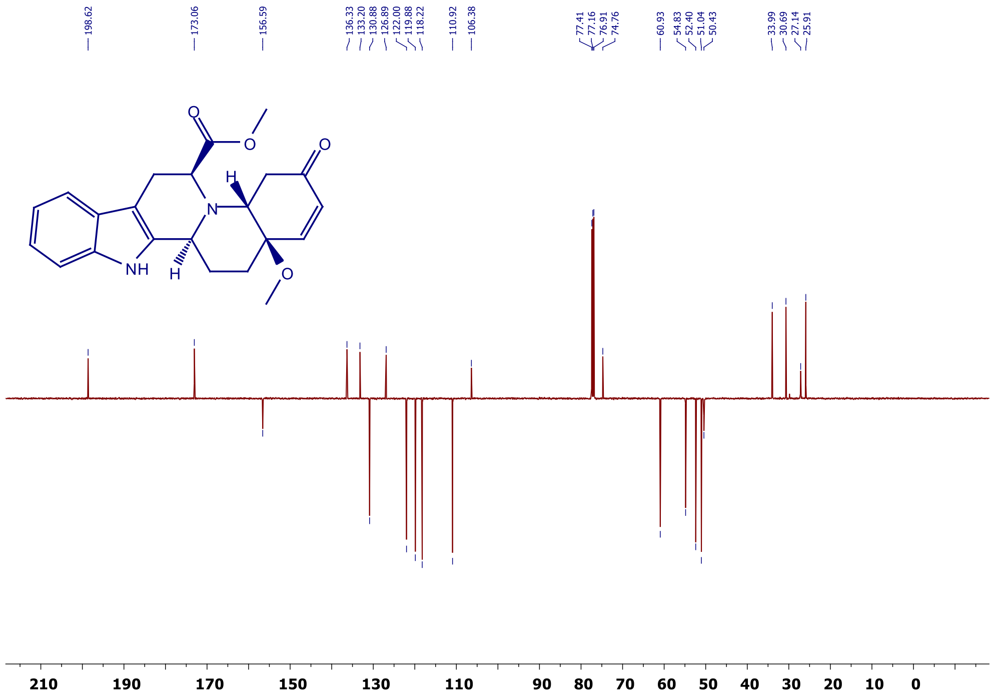

Supplementary Figure 46 APT NMR (125 MHz, $CDCl_3$) spectrum of methyl (2aR,6aR,8S,14bS)-2a-methoxy-5-oxo-1,2,2a,5,6,6a,8,9,14,14b-decahydroindolo[2',3':3,4]pyrido[1,2-a]quinoline-8-carboxylate (**8a**)

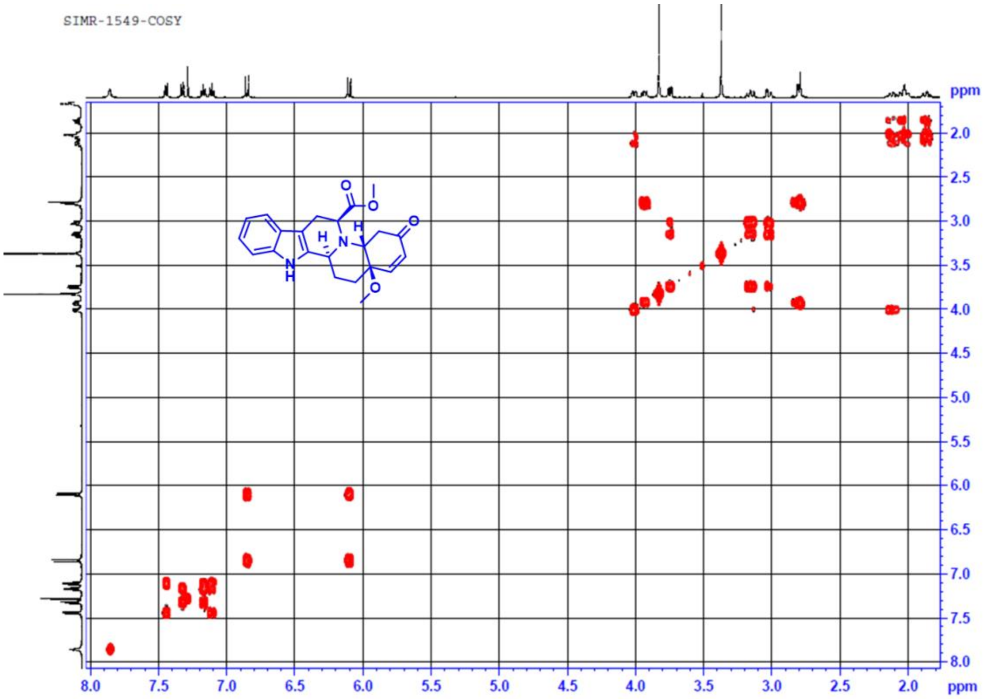

Supplementary Figure 47 COSY (500 MHz, $CDCl_3$) spectrum of methyl (2aR,6aR,8S,14bS)-2a-methoxy-5-oxo-1,2,2a,5,6,6a,8,9,14,14b-decahydroindolo[2',3':3,4]pyrido[1,2-a]quinoline-8-carboxylate (**8a**)

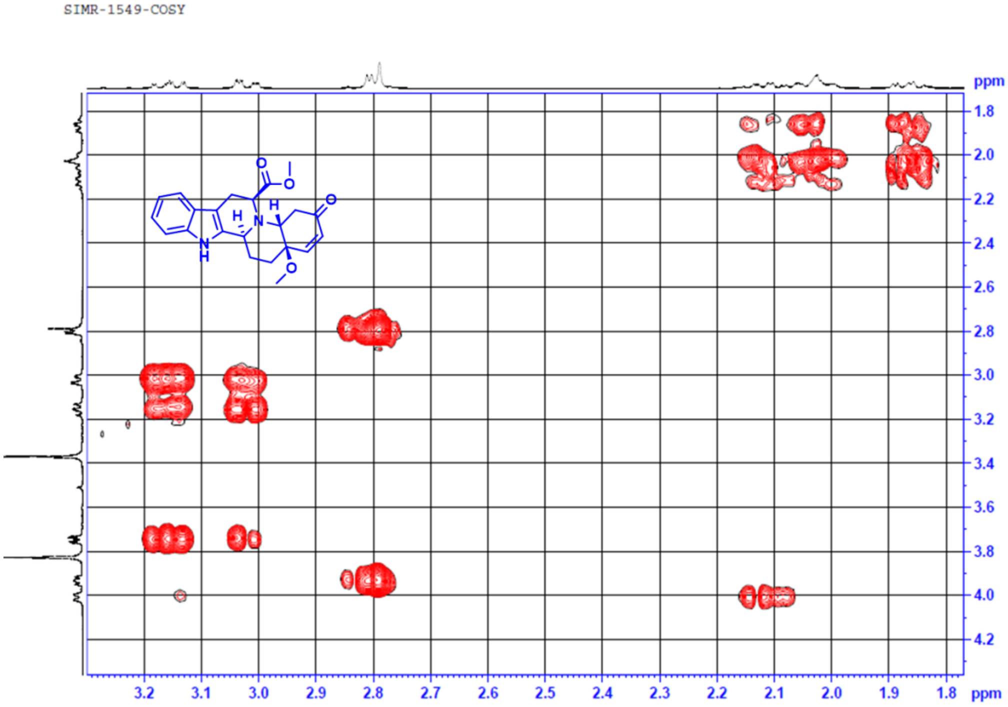

Supplementary Figure 48 COSY (Expansion) spectrum of methyl (2aR,6aR,8S,14bS)-2a-methoxy-5-oxo-1,2,2a,5,6,6a,8,9,14,14b-decahydroindolo[2',3':3,4]pyrido[1,2-a]quinoline-8-carboxylate (**8a**)

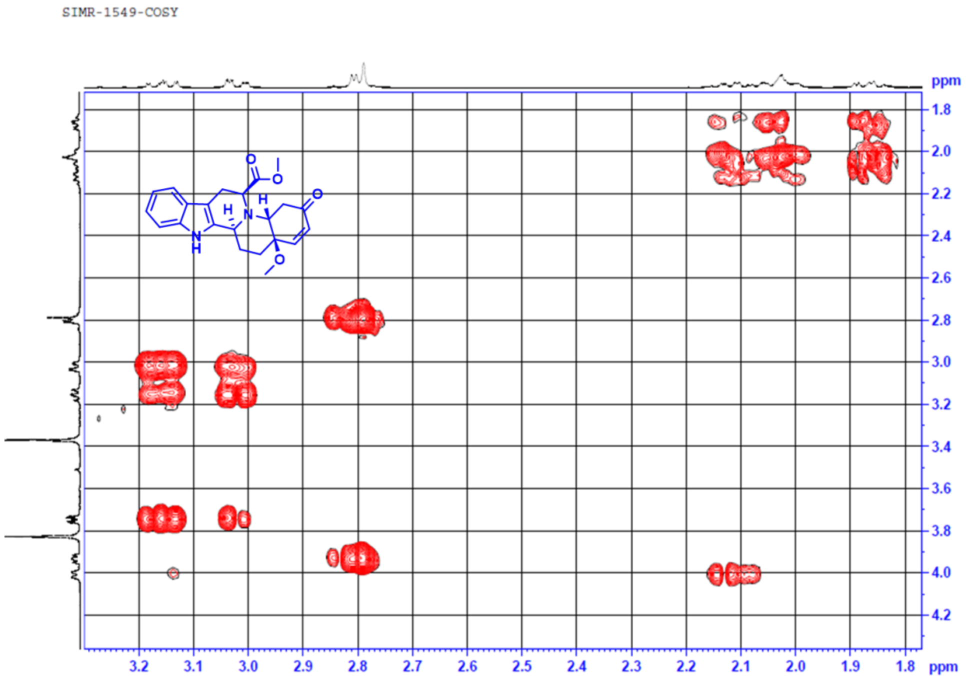

Supplementary Figure 49 COSY (Expansion) spectrum of methyl (2aR,6aR,8S,14bS)-2a-methoxy-5-oxo-1,2,2a,5,6,6a,8,9,14,14b-decahydroindolo[2',3':3,4]pyrido[1,2-a]quinoline-8-carboxylate (**8a**)

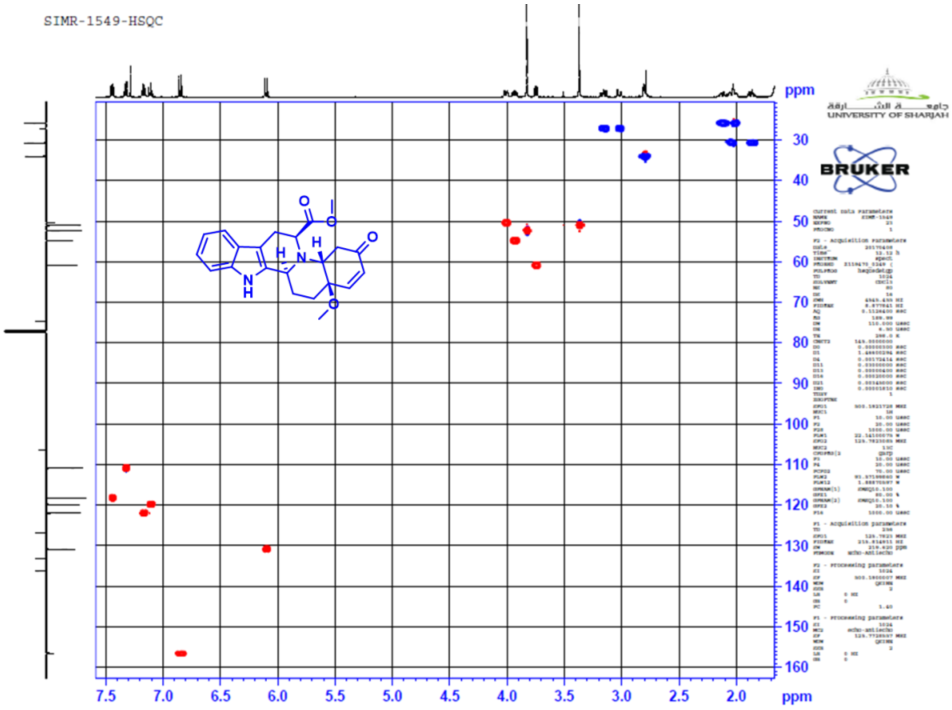

Supplementary Figure 50 HSQC (500 MHz, $CDCl_3$) spectrum of methyl (2aR,6aR,8S,14bS)-2a-methoxy-5-oxo-1,2,2a,5,6,6a,8,9,14,14b-decahydroindolo[2',3':3,4]pyrido[1,2-a]quinoline-8-carboxylate (**8a**)

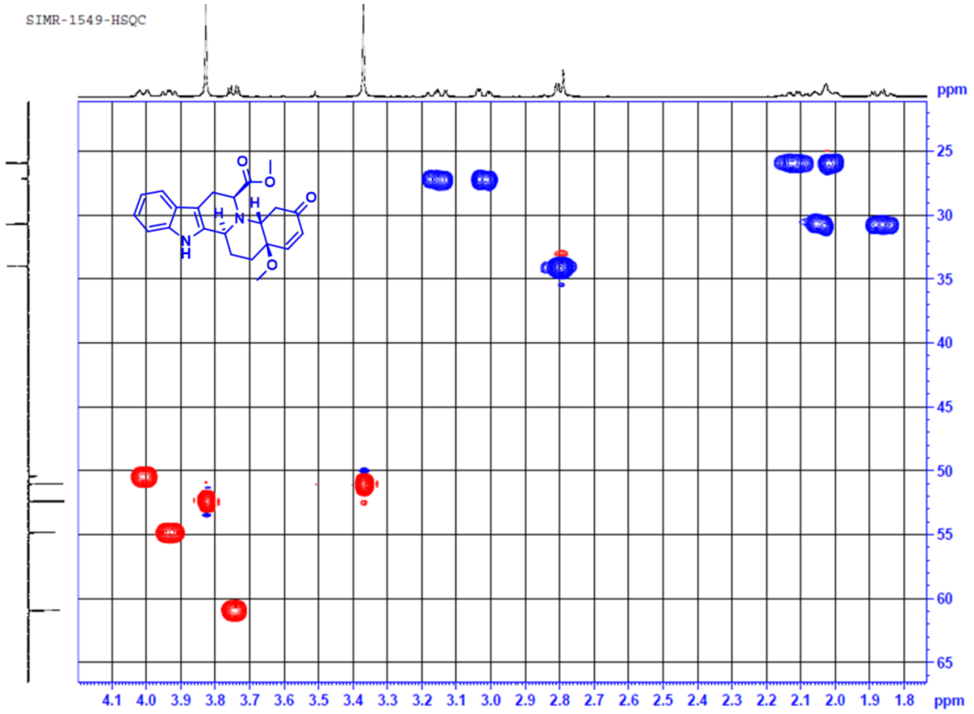

Supplementary Figure 51 HSQC (Expansion) spectrum of methyl (2aR,6aR,8S,14bS)-2a-methoxy-5-oxo-1,2,2a,5,6,6a,8,9,14,14b-decahydroindolo[2',3':3,4]pyrido[1,2-a]quinoline-8-carboxylate (**8a**)

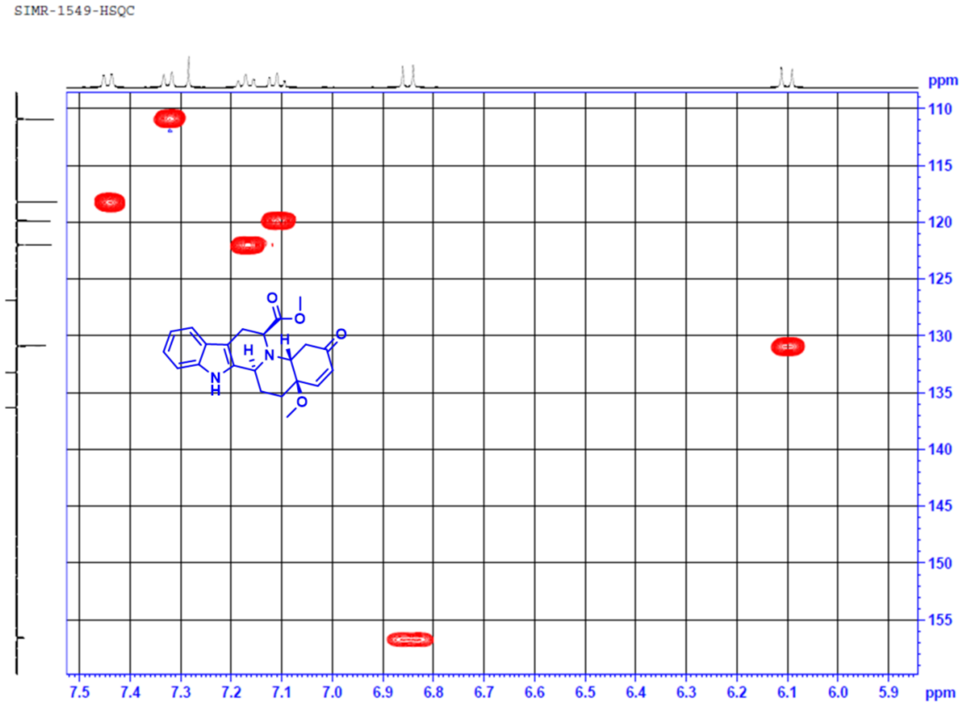

Supplementary Figure 52 HSQC (Expansion) spectrum of methyl (2aR,6aR,8S,14bS)-2a-methoxy-5-oxo-1,2,2a,5,6,6a,8,9,14,14b-decahydroindolo[2',3':3,4]pyrido[1,2-a]quinoline-8-carboxylate (**8a**)

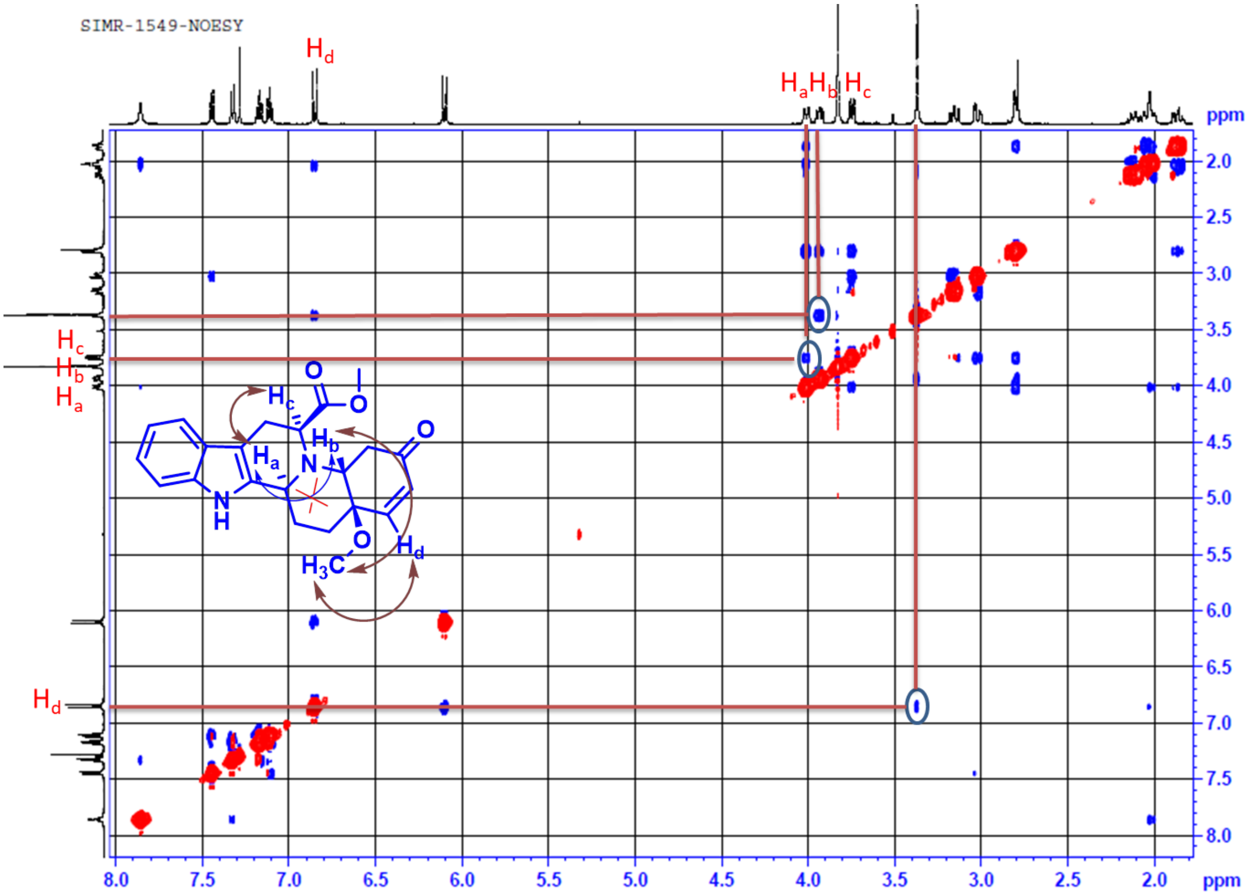

Supplementary Figure 53 NOESY (500 MHz, $CDCl_3$) spectrum of methyl (2aR,6aR,8S,14bS)-2a-methoxy-5-oxo-1,2,2a,5,6,6a,8,9,14,14b-decahydroindolo[2',3':3,4]pyrido[1,2-a]quinoline-8-carboxylate (**8a**)

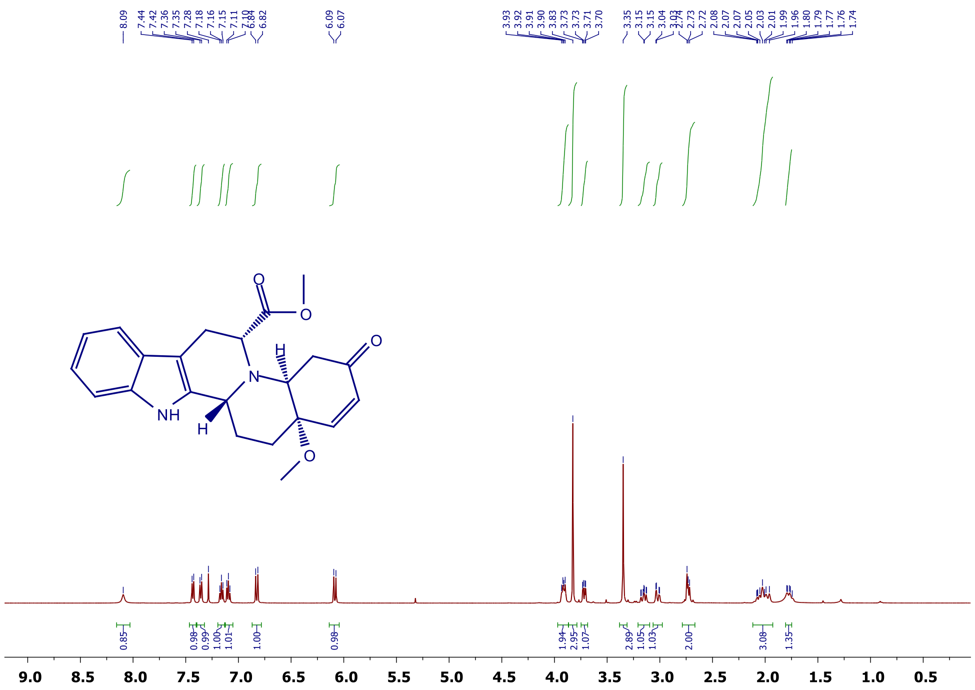

Supplementary Figure 54 ^{1}H NMR (500 MHz, $CDCl_3$) spectrum of methyl (2aS,6aS,8R,14bR)-2a-methoxy-5-oxo-1,2,2a,5,6,6a,8,9,14,14b-decahydroindolo[2',3':3,4]pyrido[1,2-a]quinoline-8-carboxylate (**8b**)

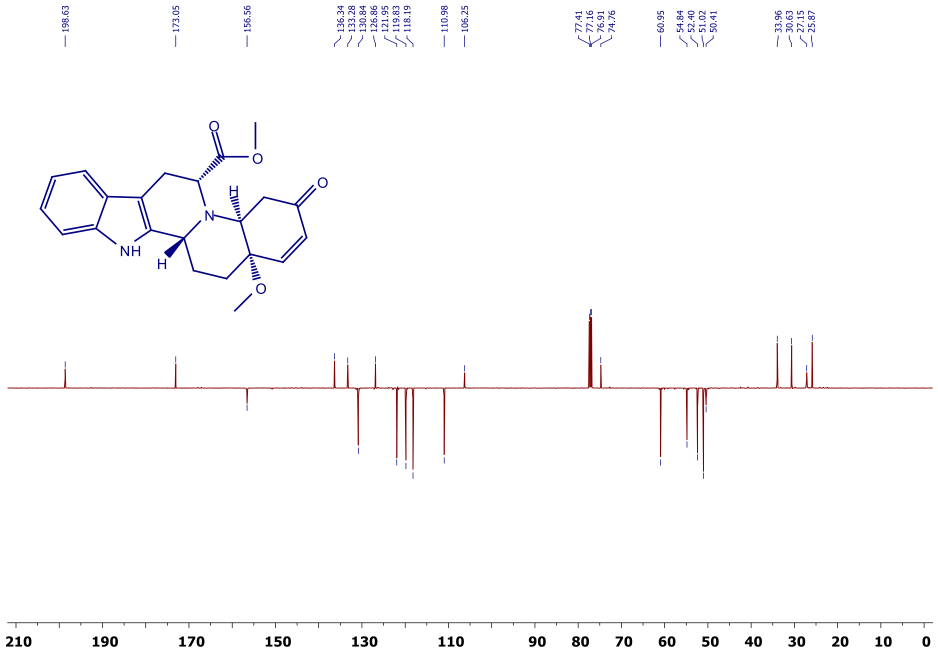

Supplementary Figure 55 APT NMR (125 MHz, $CDCl_3$) spectrum of methyl (2aS,6aS,8R,14bR)-2a-methoxy-5-oxo-1,2,2a,5,6,6a,8,9,14,14b-decahydroindolo[2',3':3,4]pyrido[1,2-a]quinoline-8-carboxylate (**8b**)

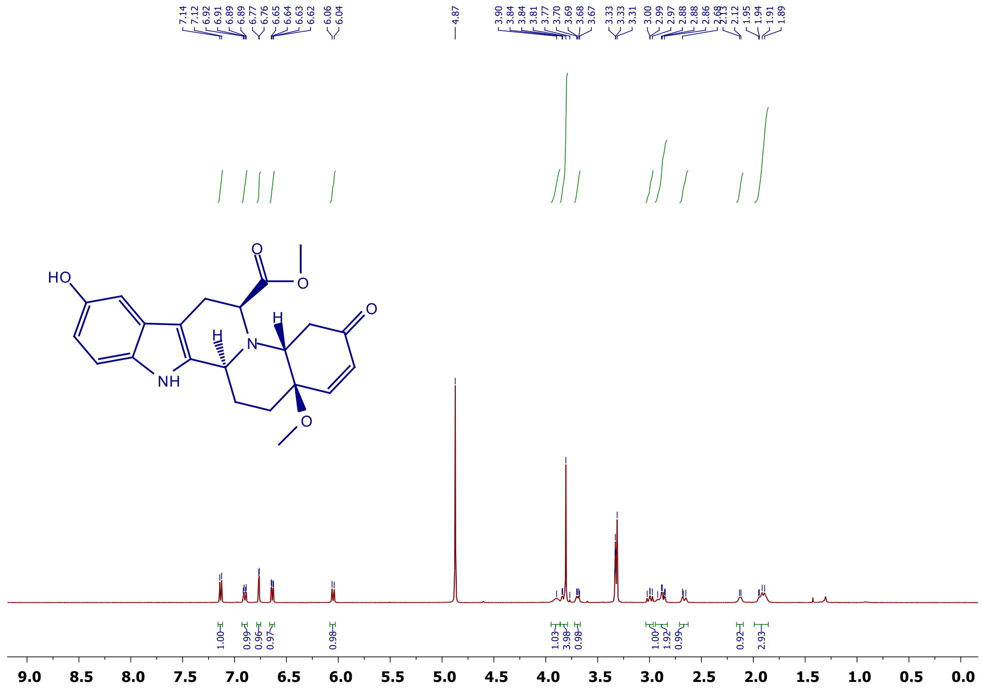

Supplementary Figure 56 ^{1}H NMR (500 MHz, CD_3OD) spectrum of Methyl (2aR,6aR,8S,14bS)-11-hydroxy-2a-methoxy-5-oxo-1,2,2a,5,6,6a,8,9,14,14b-decahydroindolo[2',3':3,4]pyrido[1,2-a]quinoline-8-carboxylate (**8c**)

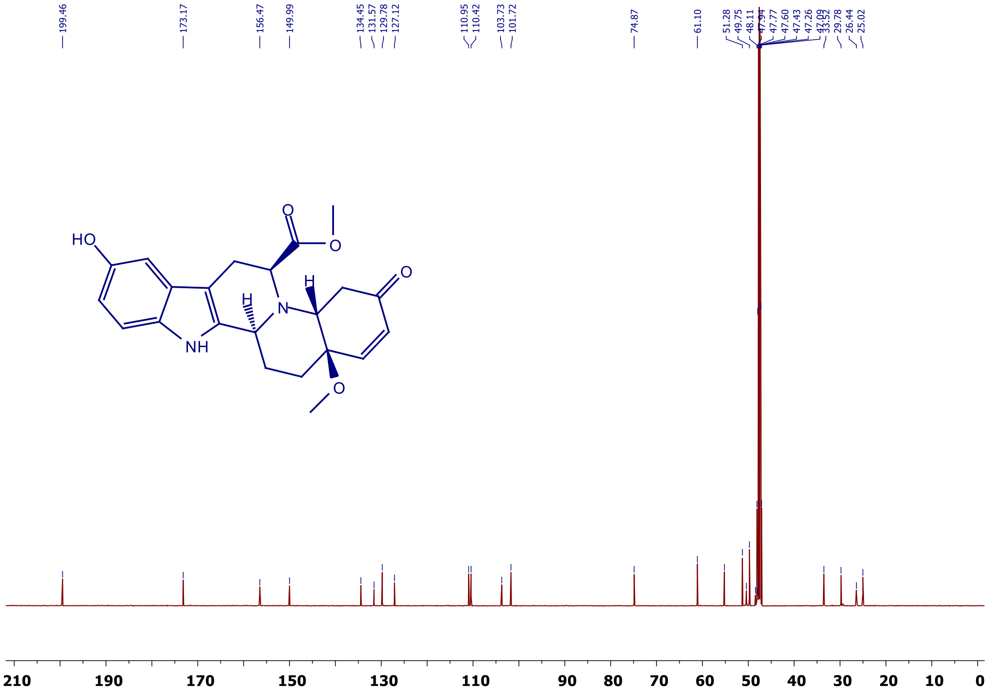

Supplementary Figure 57 ^{13}C NMR (125 MHz, CD_3OD) spectrum of Methyl (2aR,6aR,8S,14bS)-11-hydroxy-2a-methoxy-5-oxo-1,2,2a,5,6,6a,8,9,14,14b-decahydroindolo[2',3':3,4]pyrido[1,2-a]quinoline-8-carboxylate (**8c**)

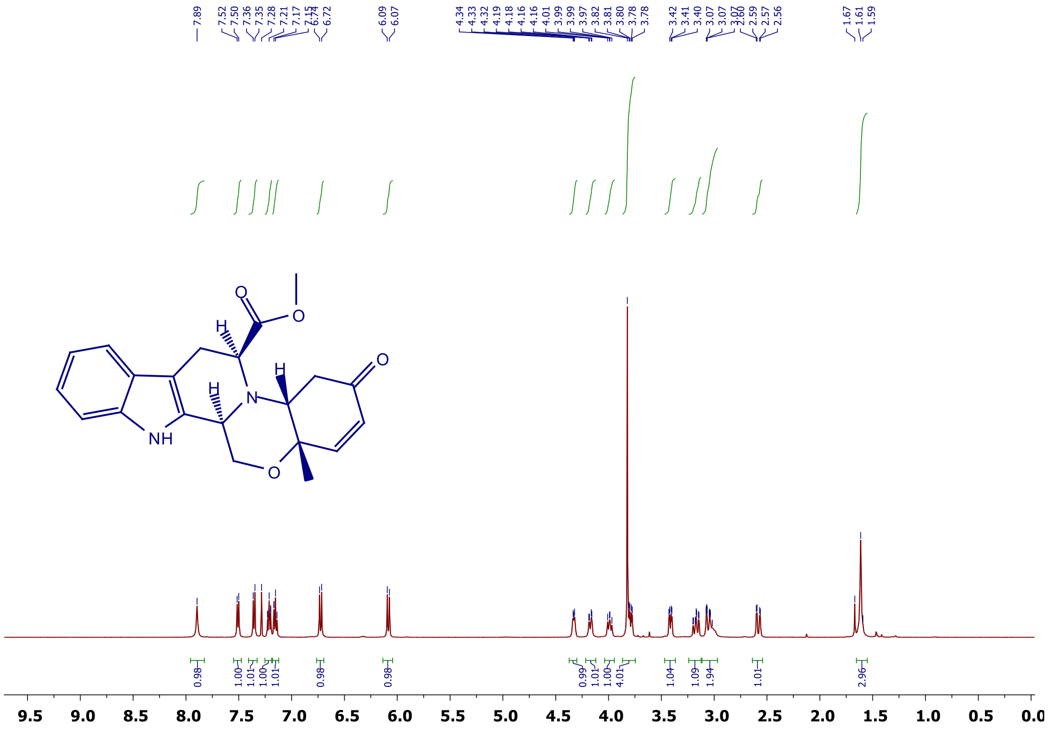

Supplementary Figure 58 ^{1}H NMR (500 MHz, $CDCl_3$) spectrum of methyl (2aS,6aR,8S,14bR)-2a-methyl-5-oxo-2a,5,6,6a,8,9,14,14b-octahydro-1H-benzo[5',6'][1,4]oxazino[4',3':1,2]pyrido[3,4-b]indole-8-carboxylate (**8d**)

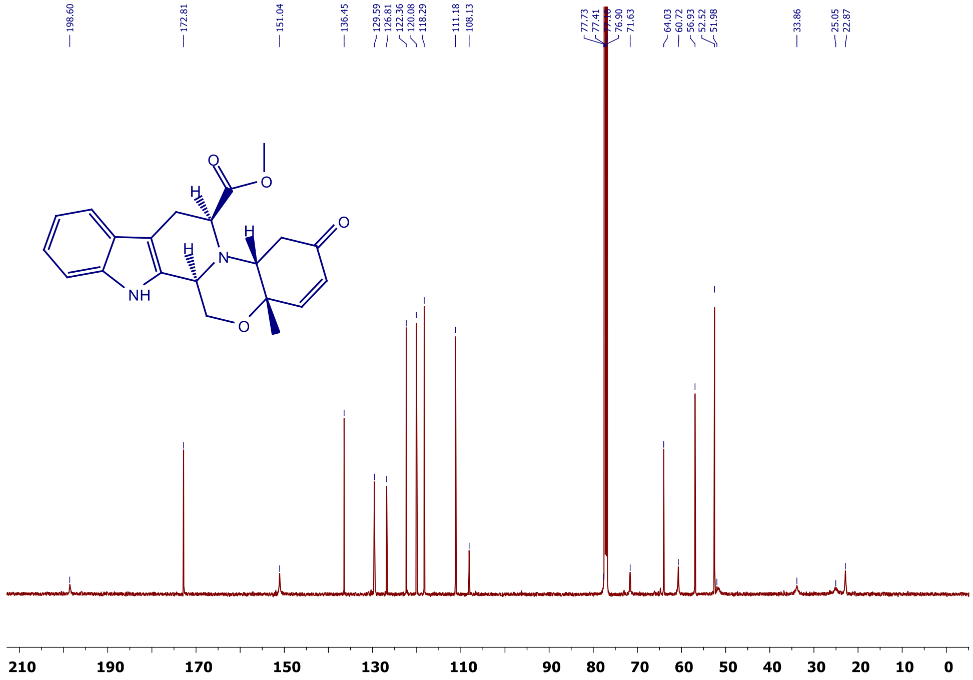

Supplementary Figure 59 ^{13}C NMR (125 MHz, $CDCl_3$) spectrum of methyl (2aS,6aR,8S,14bR)-2a-methyl-5-oxo-2a,5,6,6a,8,9,14,14b-octahydro-1H-benzo[5',6'][1,4]oxazino[4',3':1,2]pyrido[3,4-b]indole-8-carboxylate (**8d**)

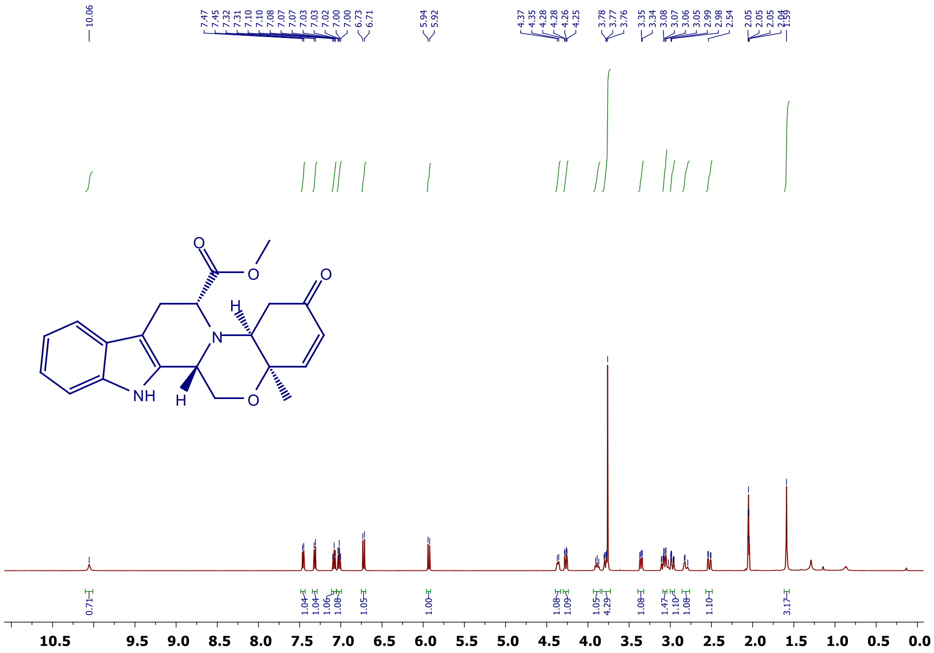

Supplementary Figure 60 ^{1}H NMR (500 MHz, Acetone-d$_6$) spectrum of methyl (2aR,6aS,8R,14bS)-2a-methyl-5-oxo-2a,5,6,6a,8,9,14,14b-octahydro-1H-benzo[5',6'][1,4]oxazino[4',3':1,2]pyrido[3,4-b]indole-8-carboxylate (**8e**)

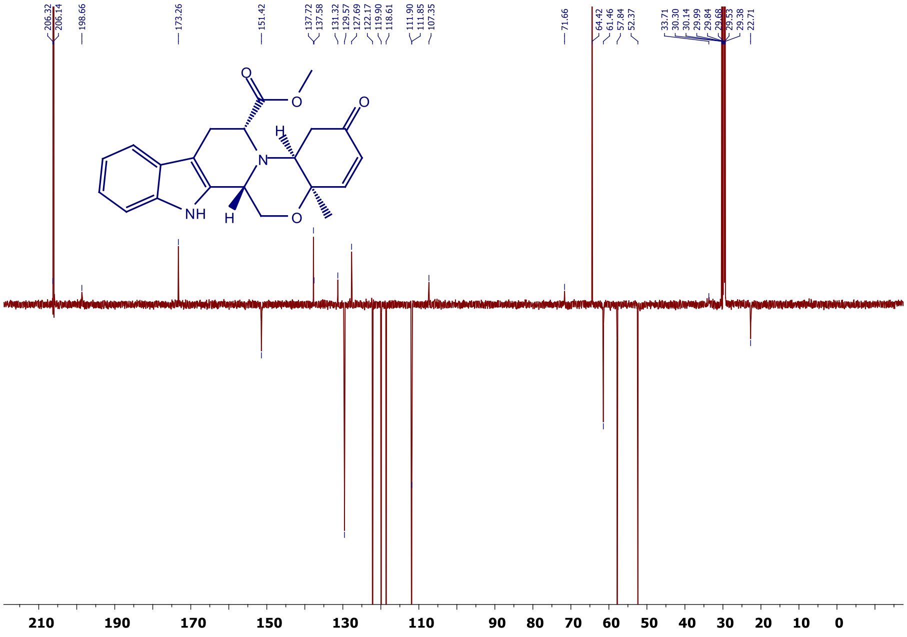

Supplementary Figure 61 APT NMR (125 MHz, Acetone-d$_6$) spectrum of methyl (2aR,6aS,8R,14bS)-2a-methyl-5-oxo-2a,5,6,6a,8,9,14,14b-octahydro-1H-benzo[5',6'][1,4]oxazino[4',3':1,2]pyrido[3,4-b]indole-8-carboxylate (**8e**)

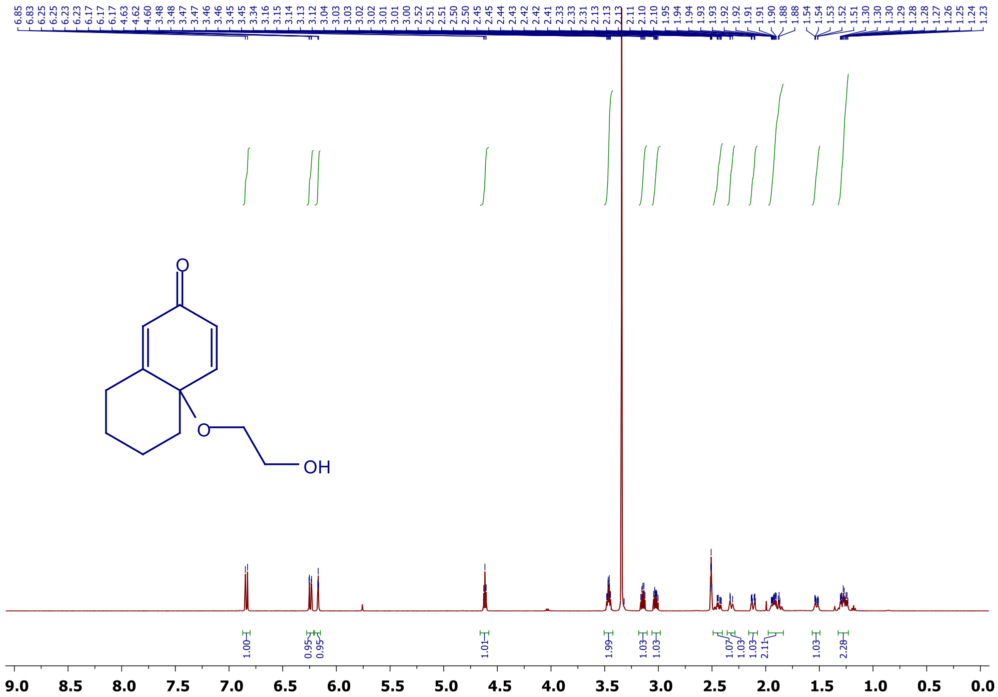

Supplementary Figure 62 ^{1}H NMR (500 MHz, DMSO-d_6) spectrum of 4a-(2-hydroxyethoxy)-5,6,7,8-tetrahydronaphthalen-2(4aH)-one (**9b**)

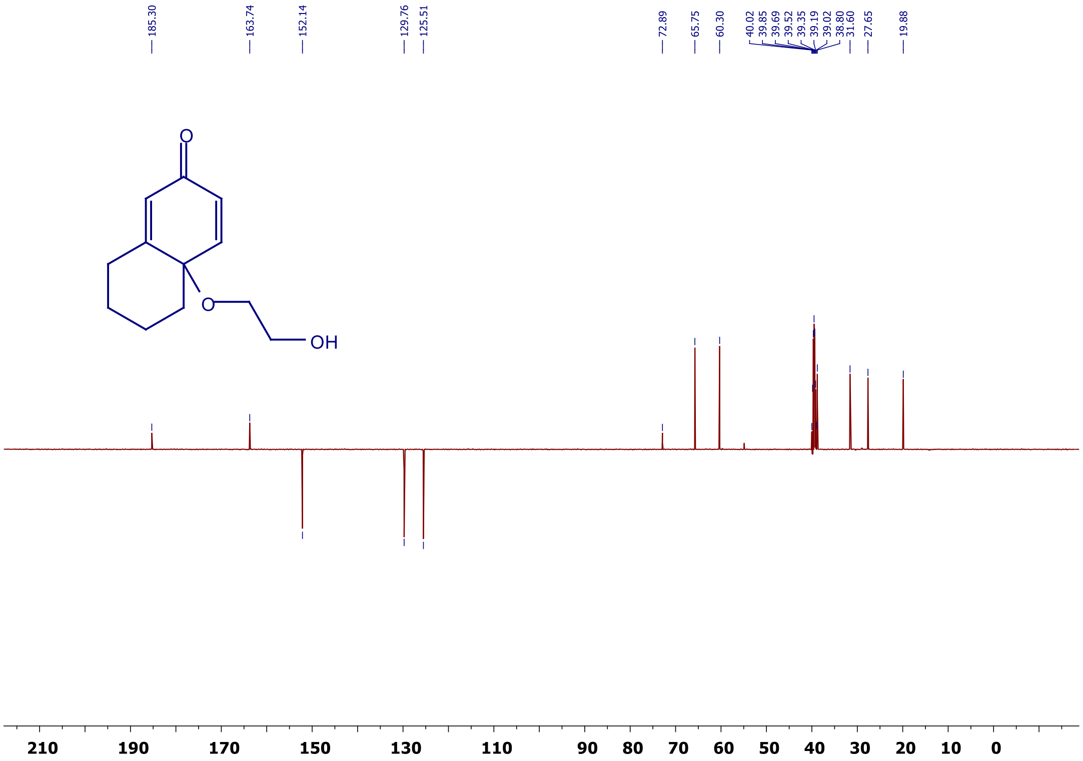

Supplementary Figure 63 APT NMR (125 MHz, DMSO-d_6) spectrum of 4a-(2-hydroxyethoxy)-5,6,7,8-tetrahydronaphthalen-2(4aH)-one (**9b**)

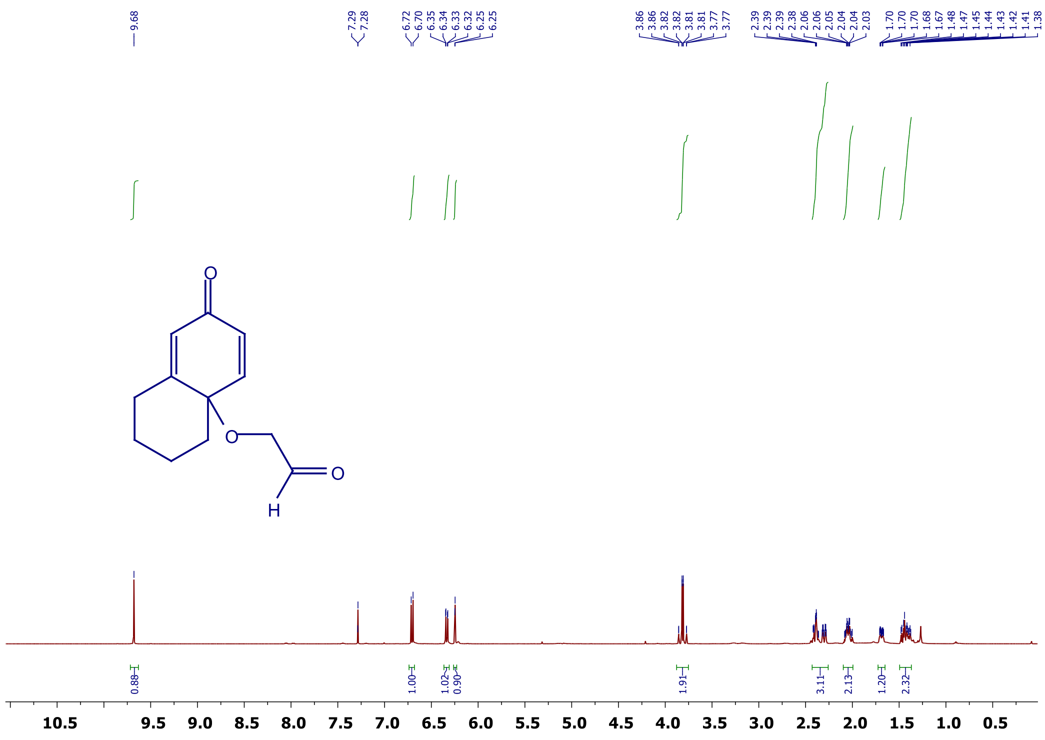

Supplementary Figure 64 ^{1}H NMR (500 MHz, $CDCl_3$) spectrum of 2-((7-oxo-1,3,4,7-tetrahydronaphthalen-4a(2H)-yl)oxy)acetaldehyde (**9**)

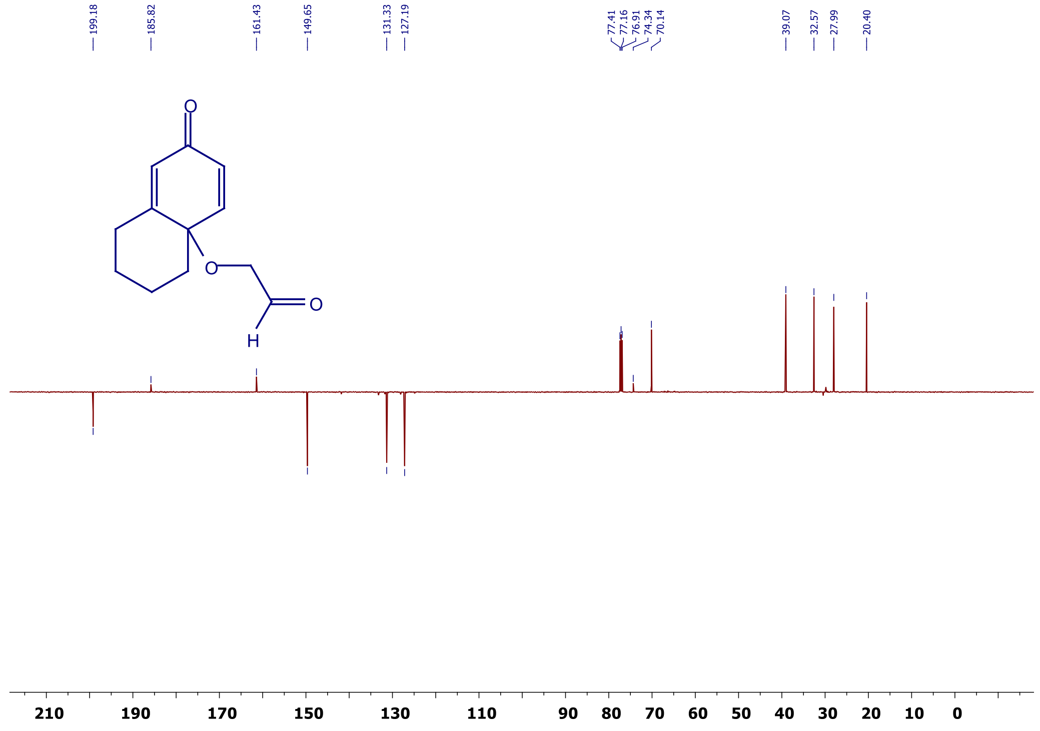

Supplementary Figure 65 APT NMR (125 MHz, $CDCl_3$) spectrum of 4a-(2-hydroxyethoxy)-5,6,7,8-tetrahydronaphthalen-2(4aH)-one (**9**)

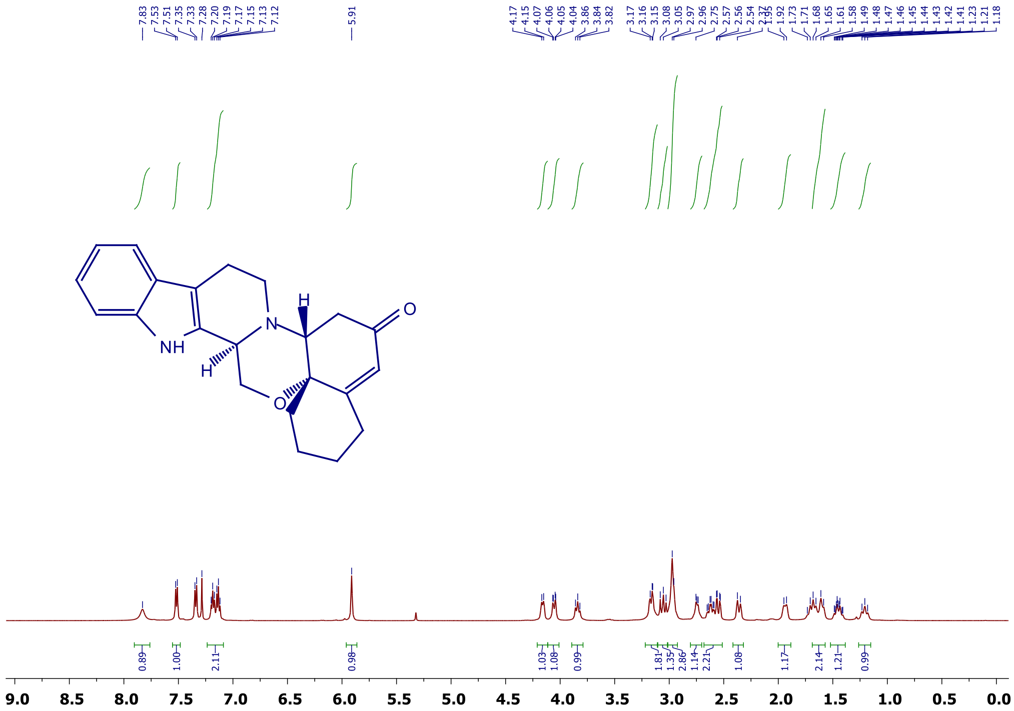

Supplementary Figure 66 ^{1}H NMR (500 MHz, $CDCl_3$) spectrum of (2aS,9aR,17bR)-3,4,5,6,9,9a,11,12,17,17b-decahydro-1H,8H-naphtho[1",8a":5',6'][1,4]oxazino[4',3':1,2]pyrido[3,4-b]indol-8-one (**10**)

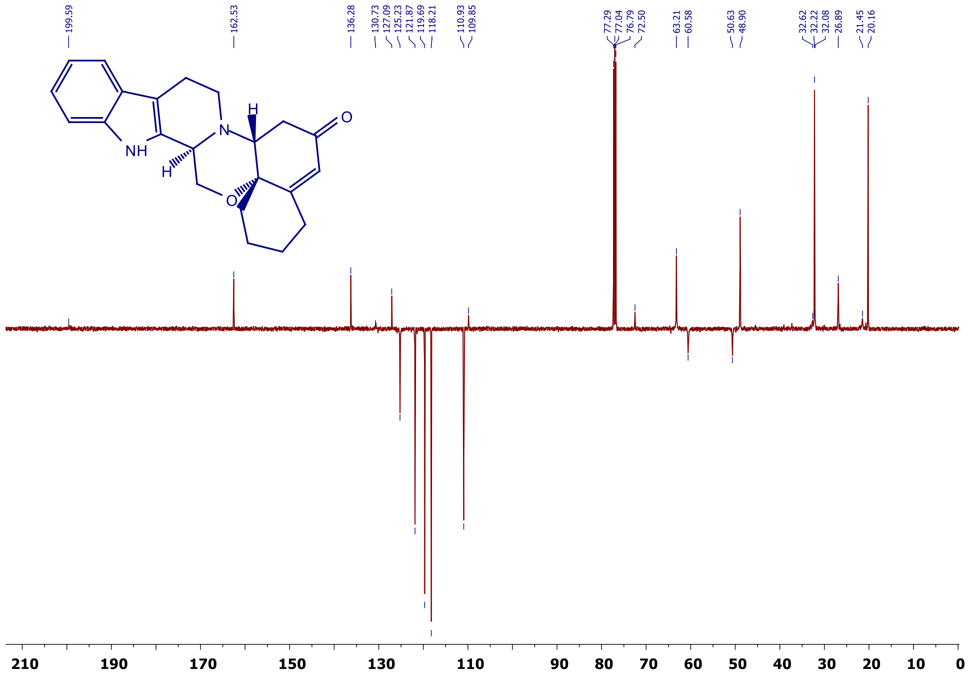

Supplementary Figure 67 APT NMR (125 MHz, $CDCl_3$) spectrum of (2aS,9aR,17bR)-3,4,5,6,9,9a,11,12,17,17b-decahydro-1H,8H-naphtho[1",8a":5',6'][1,4]oxazino[4',3':1,2]pyrido[3,4-b]indol-8-one (**10**)

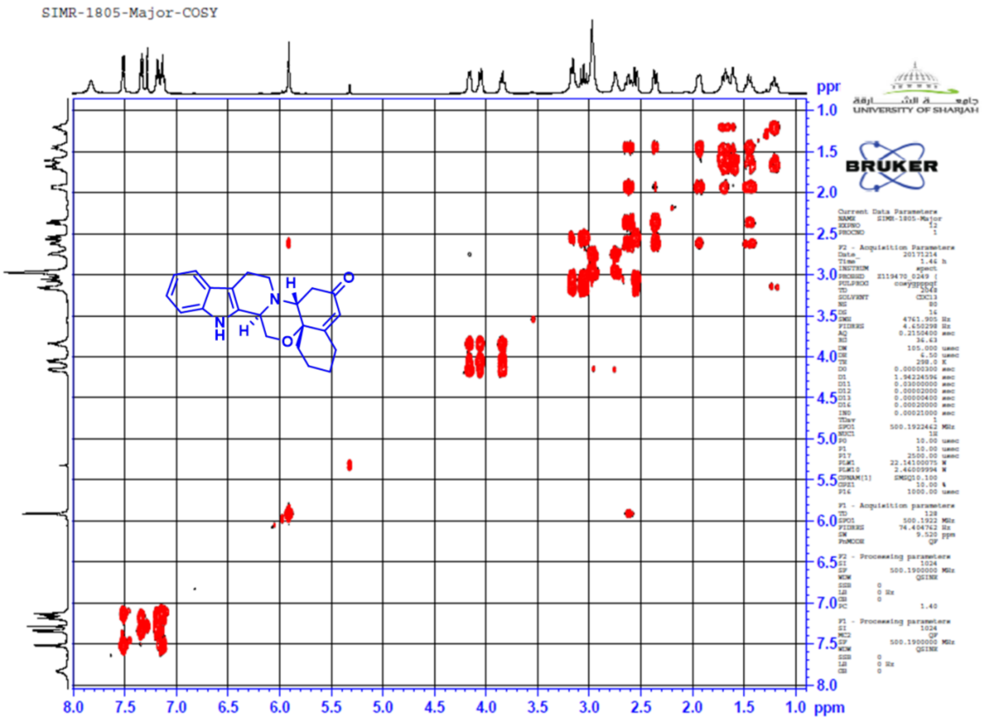

Supplementary Figure 68 COSY (500 MHz, $CDCl_3$) spectrum of (2aS,9aR,17bR)-3,4,5,6,9,9a,11,12,17,17b-decahydro-1H,8H-naphtho[1",8a":5',6'][1,4]oxazino[4',3':1,2]pyrido[3,4-b]indol-8-one (**10**)

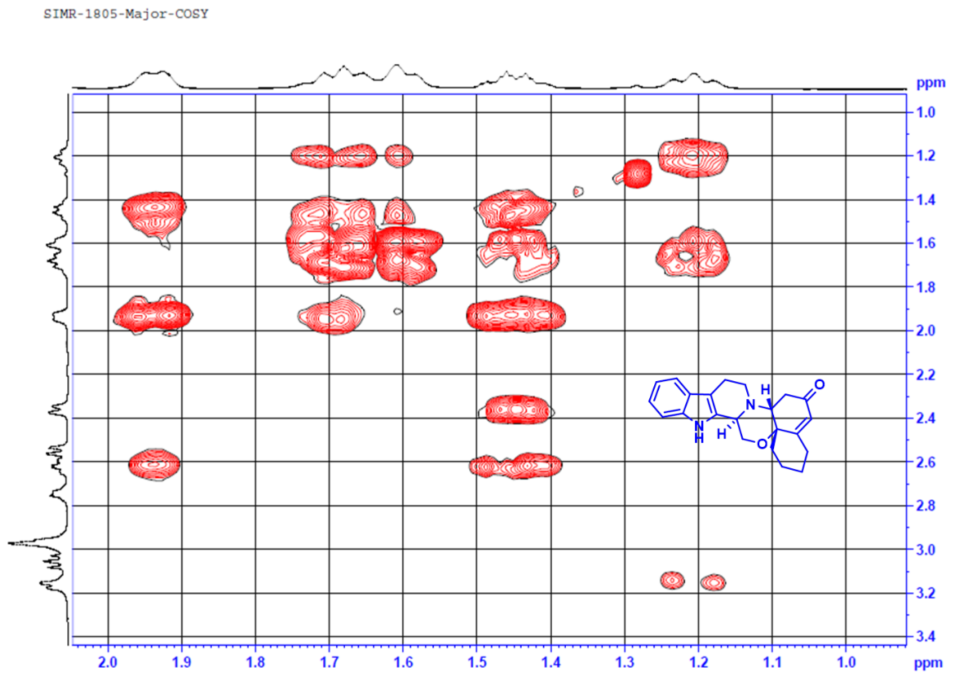

Supplementary Figure 69 COSY (Expansion) spectrum of (2aS,9aR,17bR)-3,4,5,6,9,9a,11,12,17,17b-decahydro-1H,8H-naphtho[1",8a":5',6'][1,4]oxazino[4',3':1,2]pyrido[3,4-b]indol-8-one (**10**)

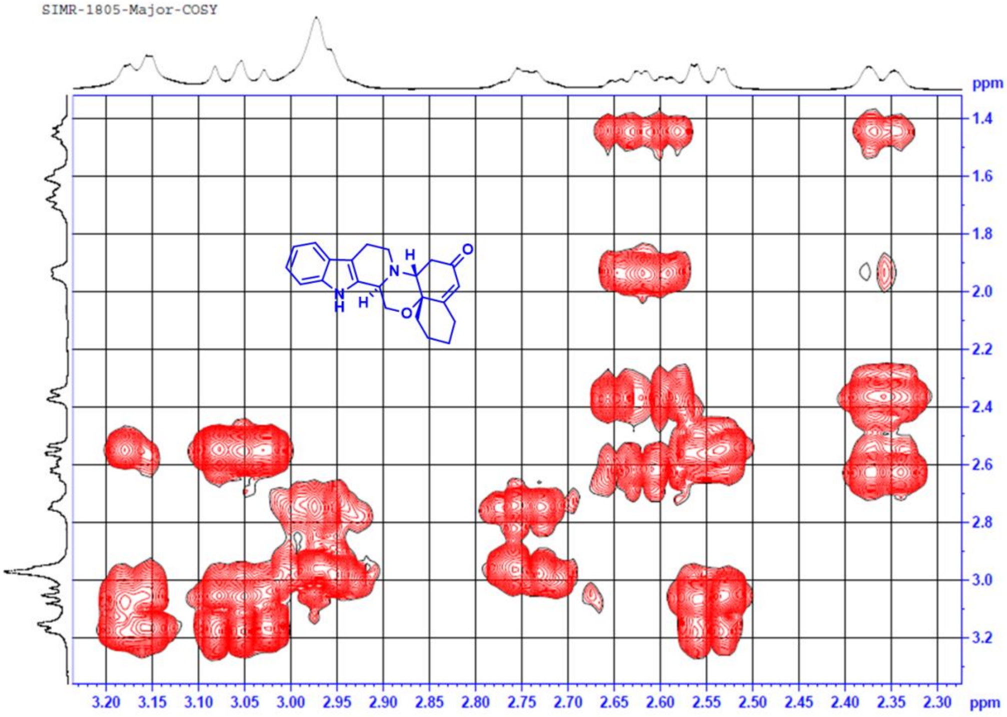

Supplementary Figure 70 COSY (Expansion) spectrum of (2aS,9aR,17bR)-3,4,5,6,9,9a,11,12,17,17b-decahydro-1H,8H-naphtho[1",8a":5',6'][1,4]oxazino[4',3':1,2]pyrido[3,4-b]indol-8-one (**10**)

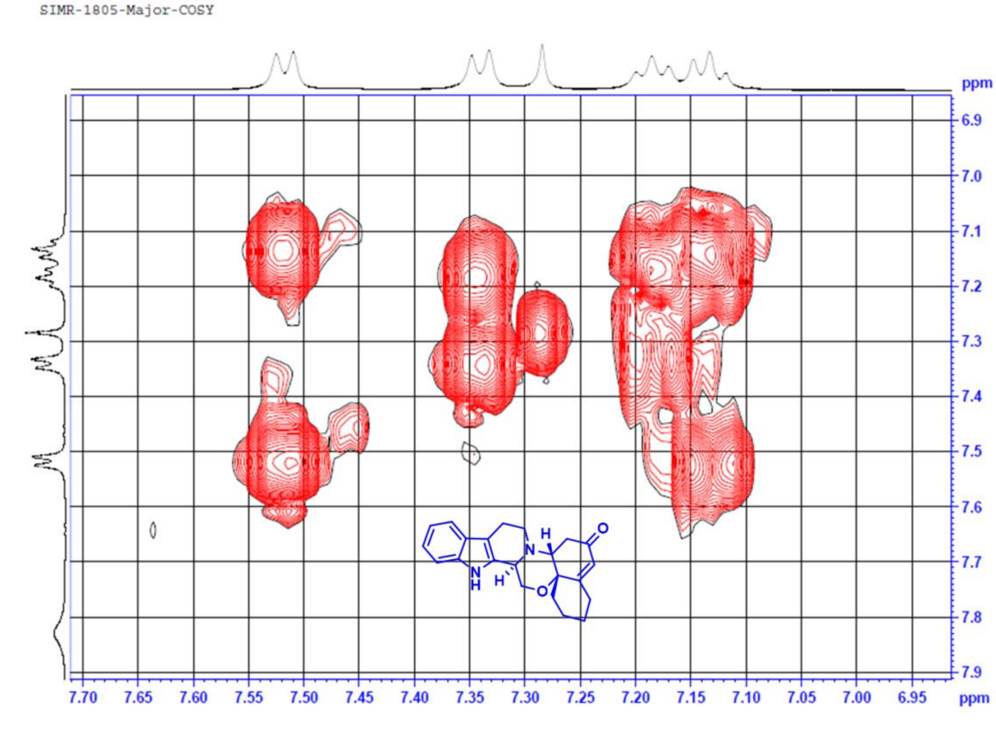

Supplementary Figure 71 COSY (Expansion) spectrum of (2aS,9aR,17bR)-3,4,5,6,9,9a,11,12,17,17b-decahydro-1H,8H-naphtho[1",8a":5',6'][1,4]oxazino[4',3':1,2]pyrido[3,4-b]indol-8-one (**10**)

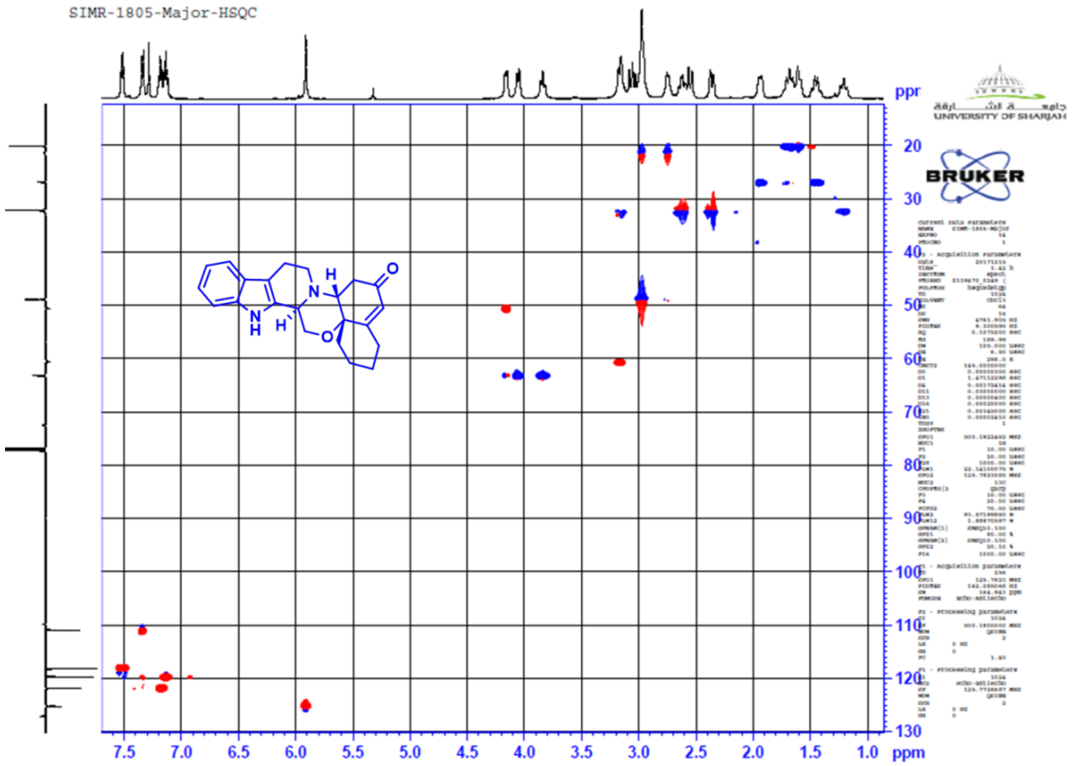

Supplementary Figure 72 HSQC (500 MHz, $CDCl_3$) spectrum of (2aS,9aR,17bR)-3,4,5,6,9,9a,11,12,17,17b-decahydro-1H,8H-naphtho[1",8a":5',6'][1,4]oxazino[4',3':1,2]pyrido[3,4-b]indol-8-one (**10**)

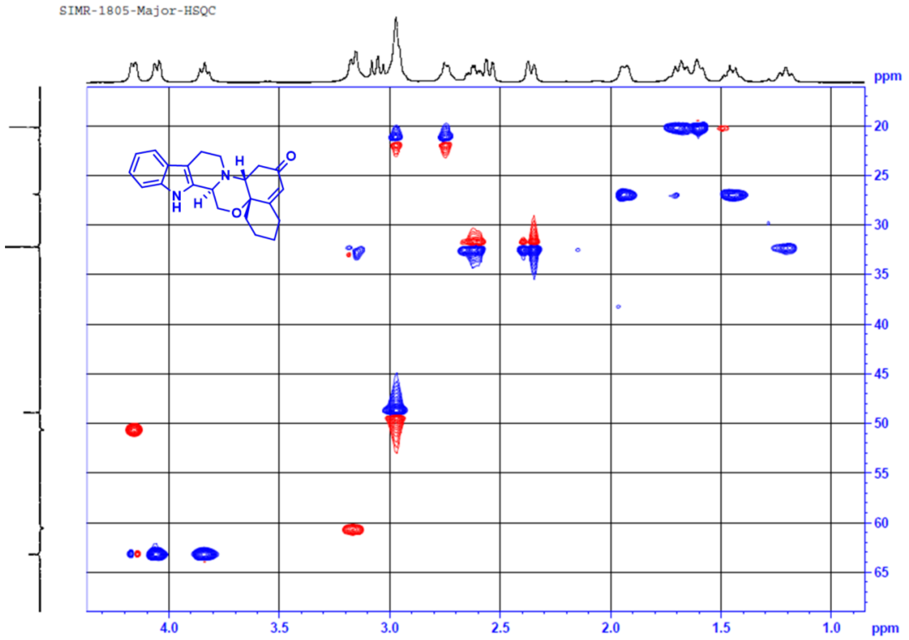

Supplementary Figure 73 HSQC (Expansion) spectrum of (2aS,9aR,17bR)-3,4,5,6,9,9a,11,12,17,17b-decahydro-1H,8H-naphtho[1",8a":5',6'][1,4]oxazino[4',3':1,2]pyrido[3,4-b]indol-8-one (**10**)

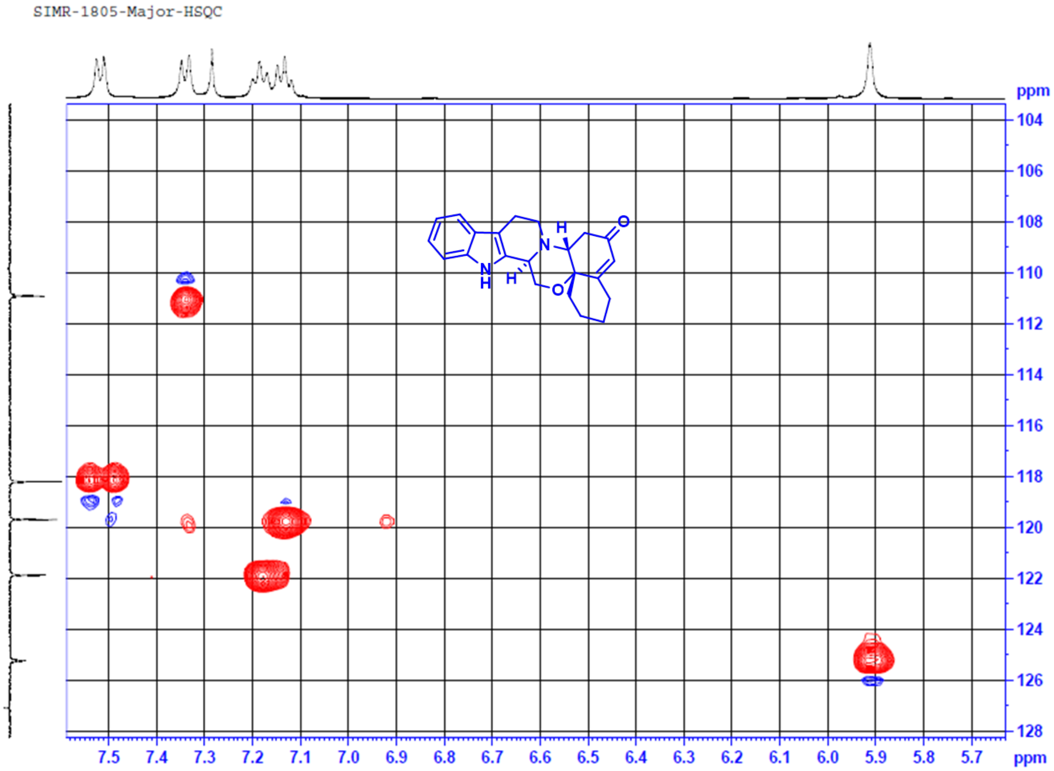

Supplementary Figure 74 HSQC (Expansion) spectrum of (2aS,9aR,17bR)-3,4,5,6,9,9a,11,12,17,17b-decahydro-1H,8H-naphtho[1",8a":5',6'][1,4]oxazino[4',3':1,2]pyrido[3,4-b]indol-8-one (**10**)

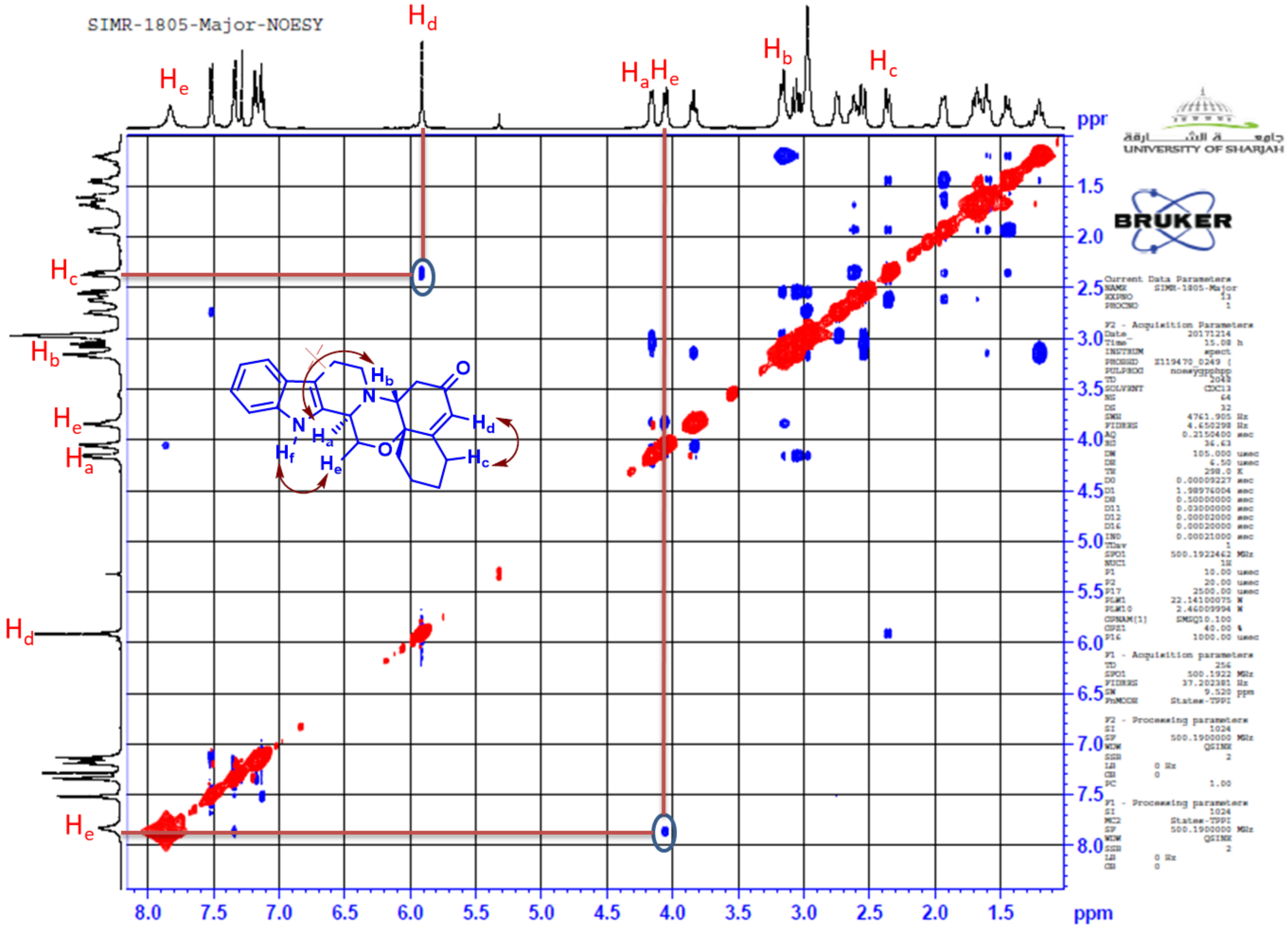

Supplementary Figure 75 NOESY (500 MHz, $CDCl_3$) spectrum of (2aS,9aR,17bR)-3,4,5,6,9,9a,11,12,17,17b-decahydro-1H,8H-naphtho[1",8a":5',6'][1,4]oxazino[4',3':1,2]pyrido[3,4-b]indol-8-one (**10**)

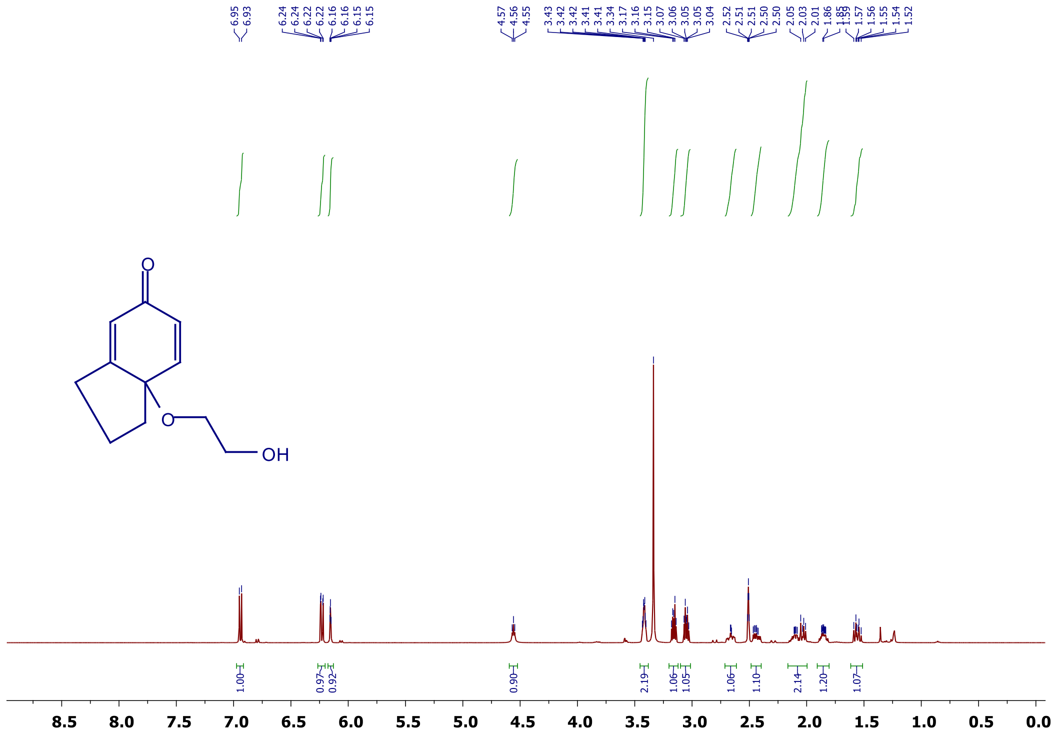

Supplementary Figure 76 ^{1}H NMR (500 MHz, DMSO-d$_6$) spectrum of 7a-(2-hydroxyethoxy)-1,2,3,7a-tetrahydro-5H-inden-5-one (**11b**)

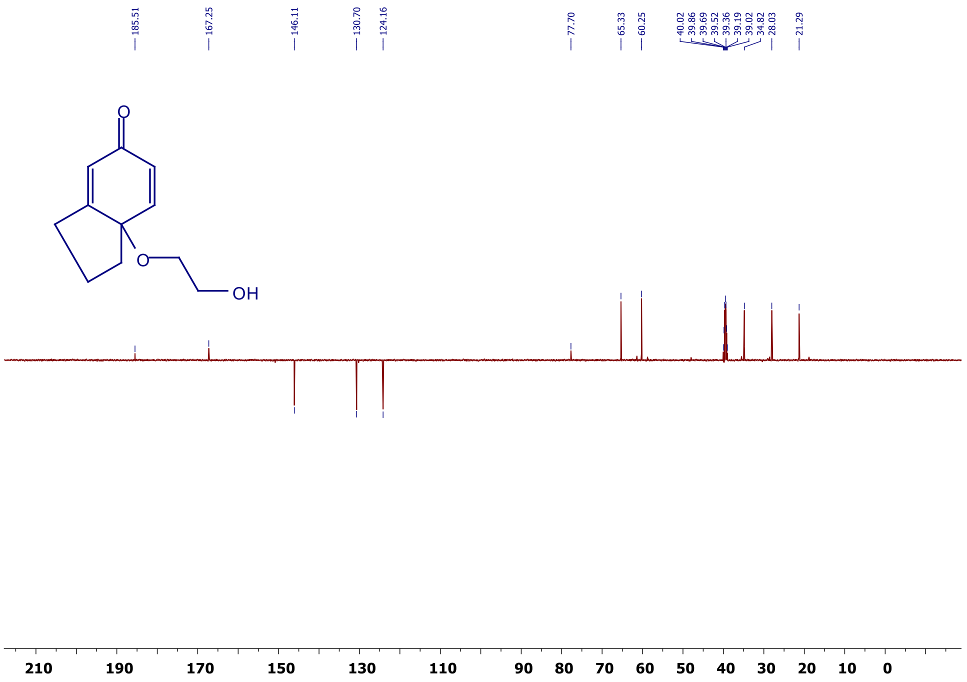

Supplementary Figure 77 APT NMR (125 MHz, DMSO-d$_6$) spectrum of 7a-(2-hydroxyethoxy)-1,2,3,7a-tetrahydro-5H-inden-5-one (**11b**)

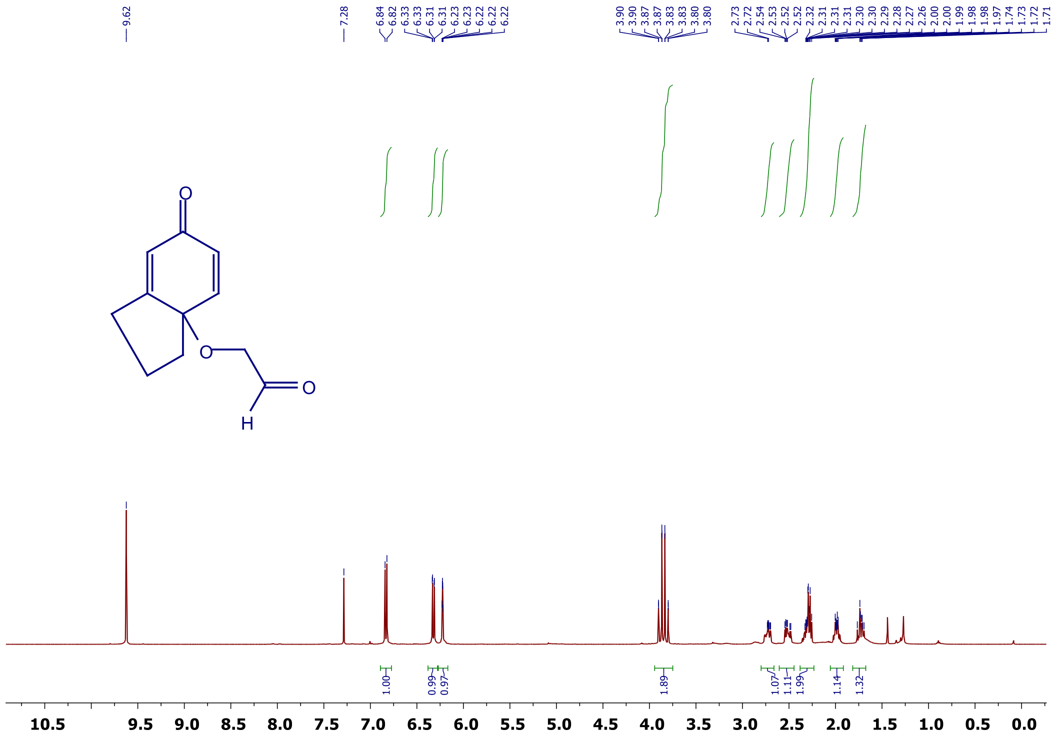

Supplementary Figure 78 ^{1}H NMR (500 MHz, $CDCl_3$) spectrum of 2-((6-oxo-1,2,3,6-tetrahydro-3aH-inden-3a-yl)oxy)acetaldehyde (**11**)

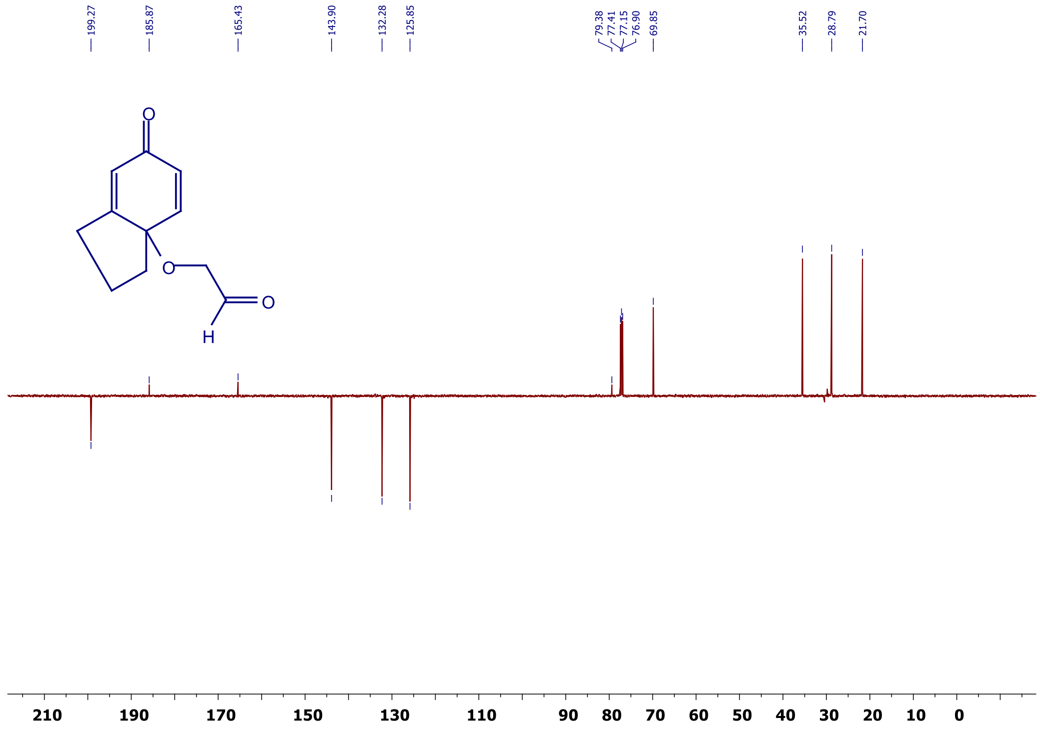

Supplementary Figure 79 APT NMR (125 MHz, $CDCl_3$) spectrum of 2-((6-oxo-1,2,3,6-tetrahydro-3aH-inden-3a-yl)oxy)acetaldehyde (**11**)

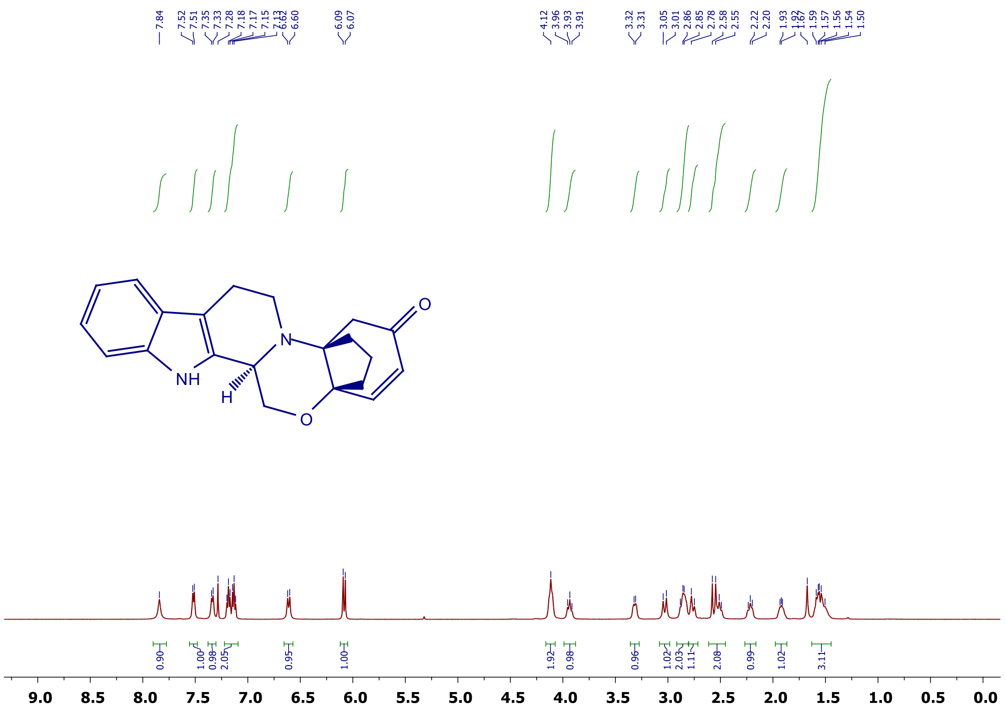

Supplementary Figure 80 ^{1}H NMR (500 MHz, $CDCl_3$) spectrum of (2aS,6aR,14bR)-8,9,14,14b-tetrahydro-1H-2a,6a-propanobenzo[5',6'][1,4]oxazino[4',3':1,2]pyrido[3,4-b]indol-5(6H)-one (**12**)

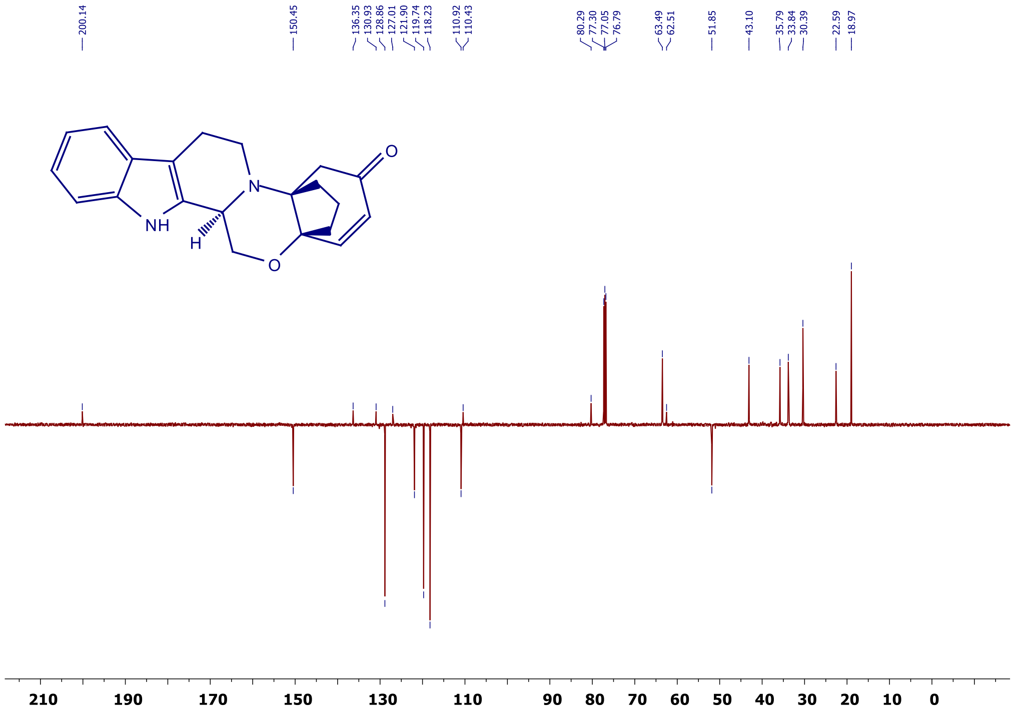

Supplementary Figure 81 APT NMR (125 MHz, $CDCl_3$) spectrum of (2aS,6aR,14bR)-8,9,14,14b-tetrahydro-1H-2a,6a-propanobenzo[5',6'][1,4]oxazino[4',3':1,2]pyrido[3,4-b]indol-5(6H)-one (**12**)

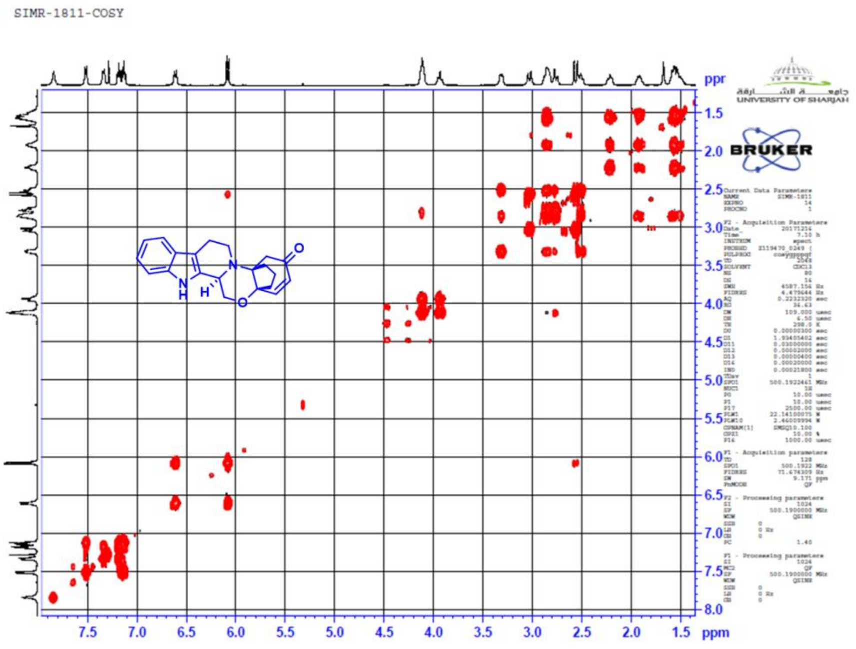

Supplementary Figure 82 COSY (500 MHz, $CDCl_3$) spectrum of (2aS,6aR,14bR)-8,9,14,14b-tetrahydro-1H-2a,6a-propanobenzo[5',6'][1,4]oxazino[4',3':1,2]pyrido[3,4-b]indol-5(6H)-one (**12**)

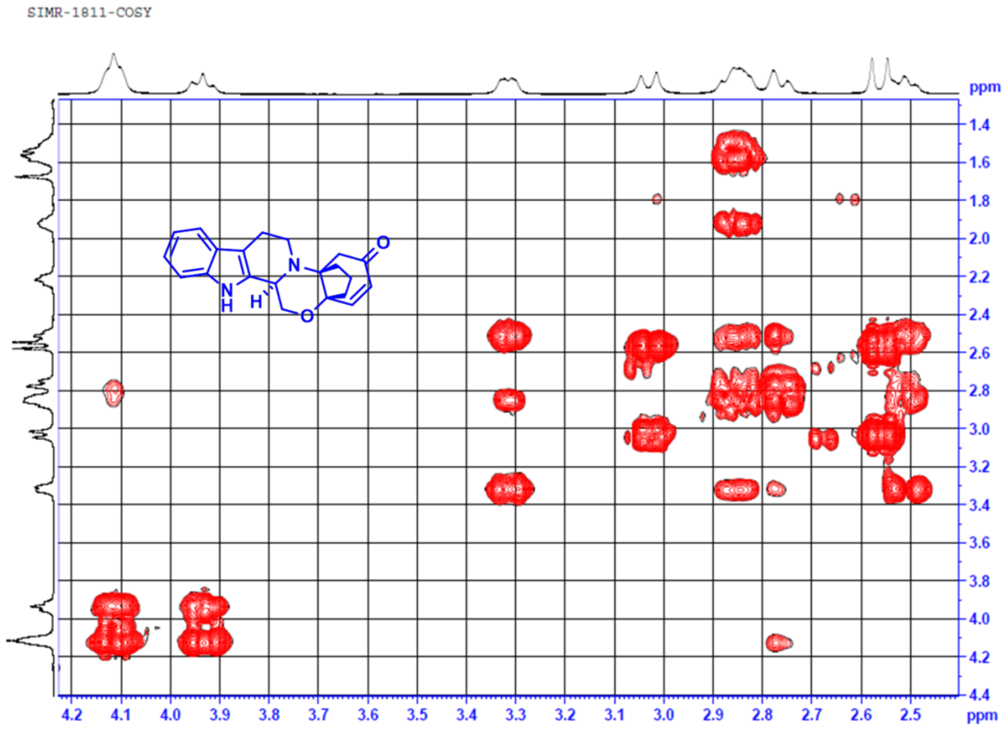

Supplementary Figure 83 COSY (Expansion) spectrum of (2aS,6aR,14bR)-8,9,14,14b-tetrahydro-1H-2a,6a-propanobenzo[5',6'][1,4]oxazino[4',3':1,2]pyrido[3,4-b]indol-5(6H)-one (**12**)

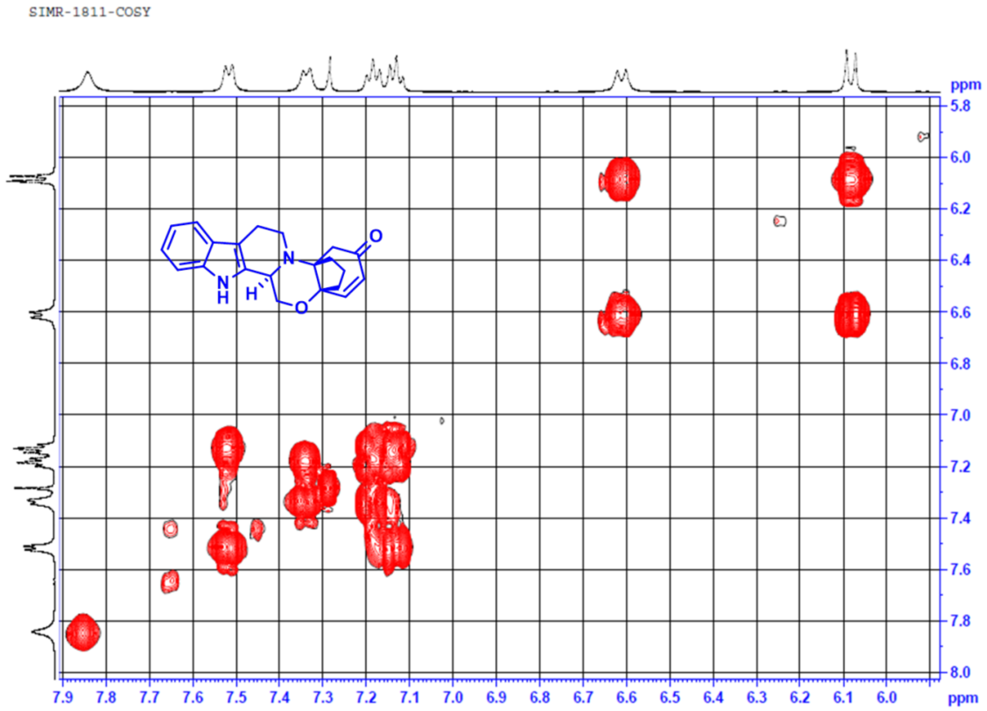

Supplementary Figure 84 COSY (Expansion) spectrum of (2aS,6aR,14bR)-8,9,14,14b-tetrahydro-1H-2a,6a-propanobenzo[5',6'][1,4]oxazino[4',3':1,2]pyrido[3,4-b]indol-5(6H)-one (**12**)

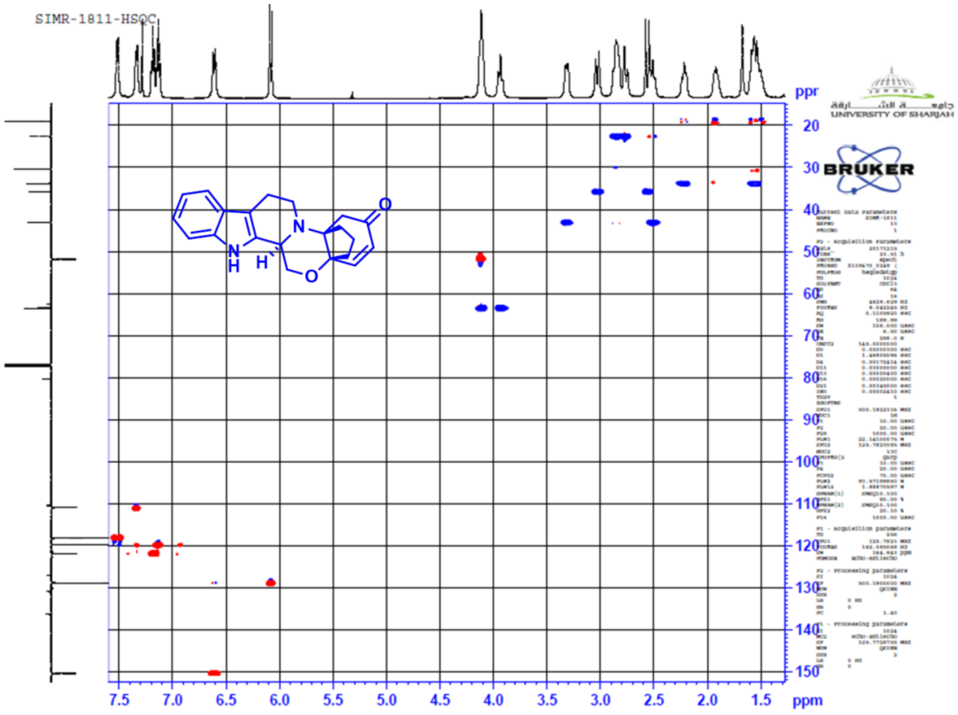

Supplementary Figure 85 HSQC (500 MHz, $CDCl_3$) spectrum of (2aS,6aR,14bR)-8,9,14,14b-tetrahydro-1H-2a,6a-propanobenzo[5',6'][1,4]oxazino[4',3':1,2]pyrido[3,4-b]indol-5(6H)-one (**12**)

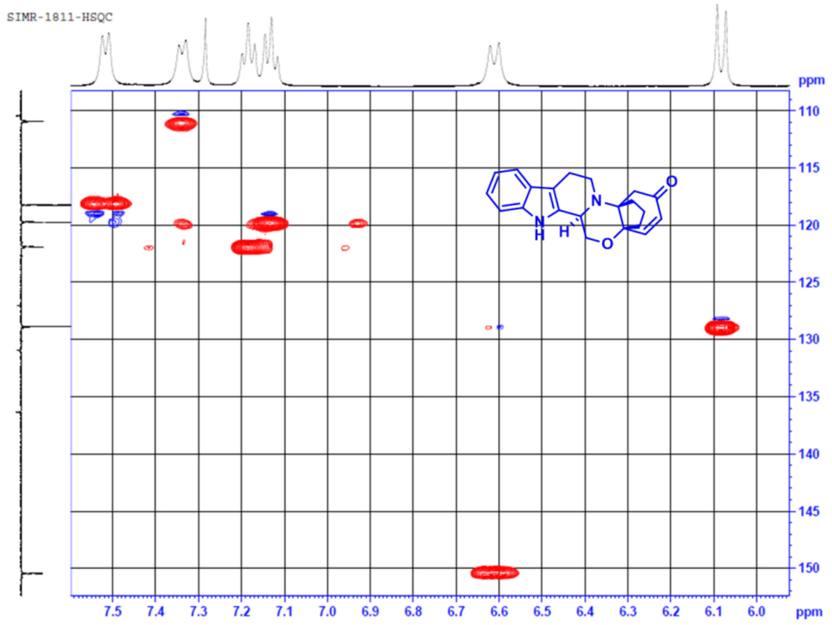

Supplementary Figure 86 HSQC (Expansion) spectrum of (2aS,6aR,14bR)-8,9,14,14b-tetrahydro-1H-2a,6a-propanobenzo[5',6'][1,4]oxazino[4',3':1,2]pyrido[3,4-b]indol-5(6H)-one (**12**)

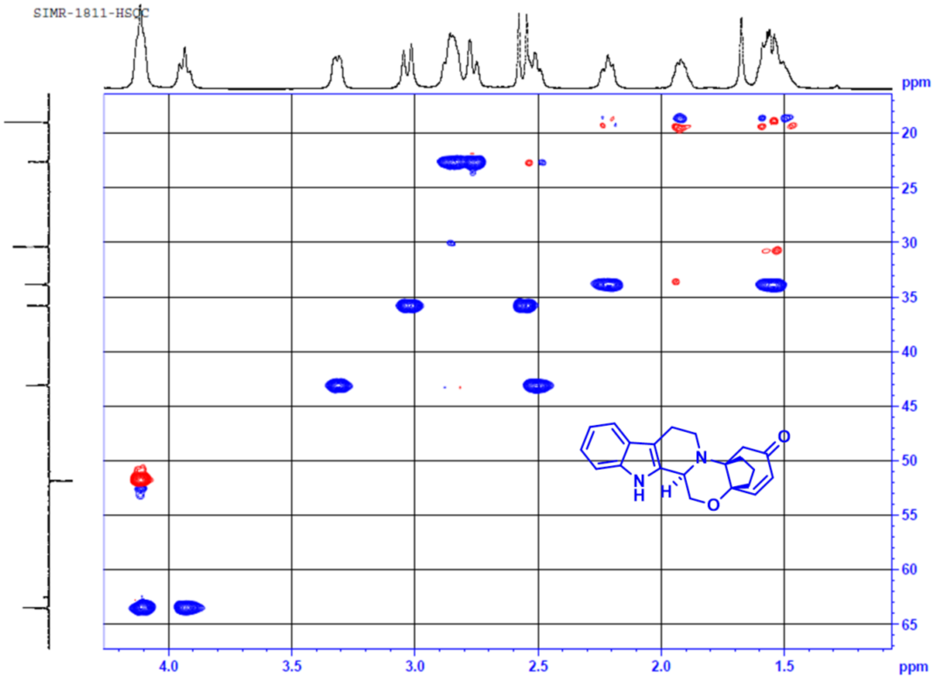

Supplementary Figure 87 HSQC (Expansion) spectrum of (2aS,6aR,14bR)-8,9,14,14b-tetrahydro-1H-2a,6a-propanobenzo[5',6'][1,4]oxazino[4',3':1,2]pyrido[3,4-b]indol-5(6H)-one (**12**)

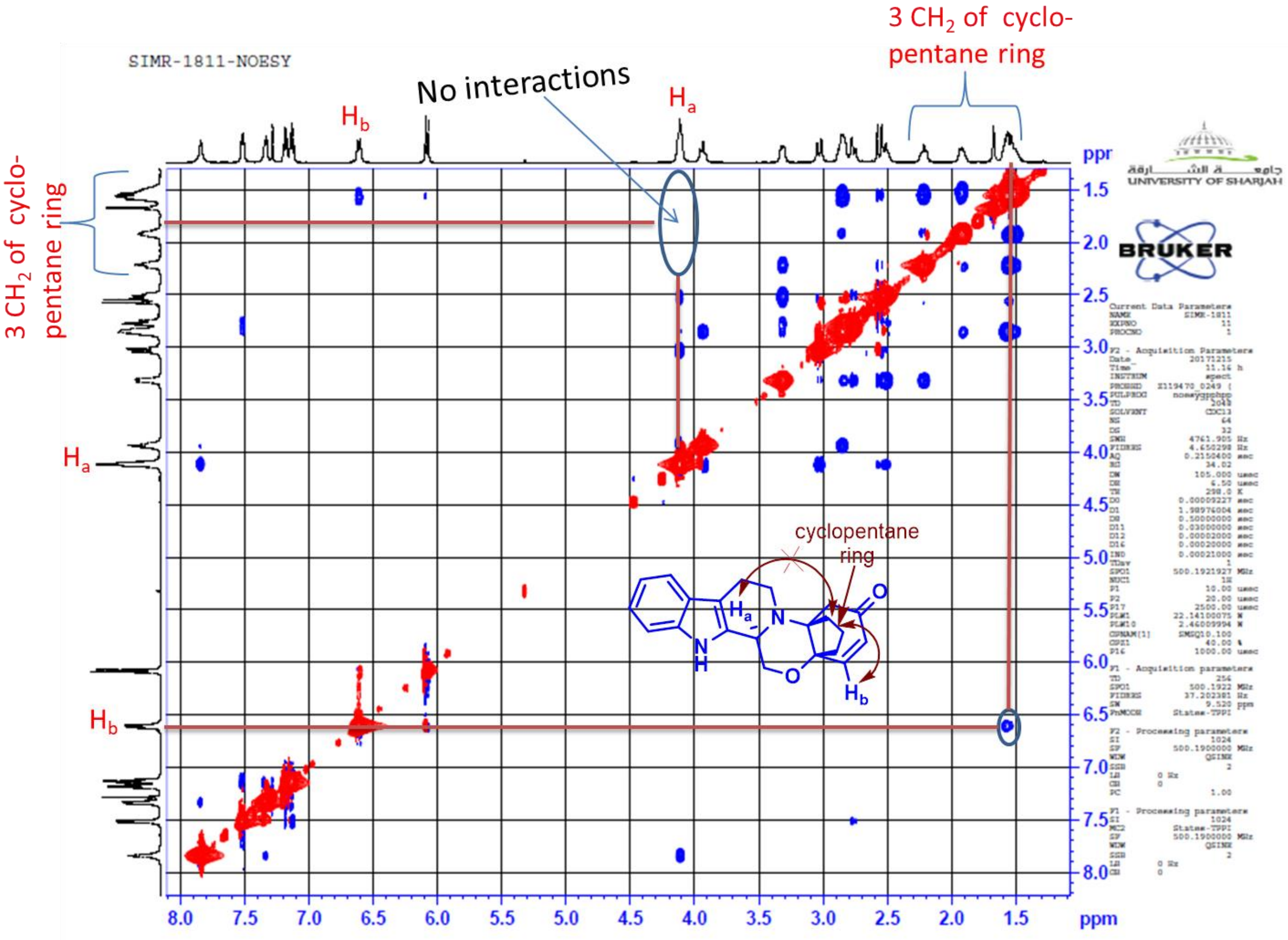

Supplementary Figure 88 NOESY (500 MHz, $CDCl_3$) spectrum of (2aS,6aR,14bR)-8,9,14,14b-tetrahydro-1H-2a,6a-propanobenzo[5',6'][1,4]oxazino[4',3':1,2]pyrido[3,4-b]indol-5(6H)-one (**12**)

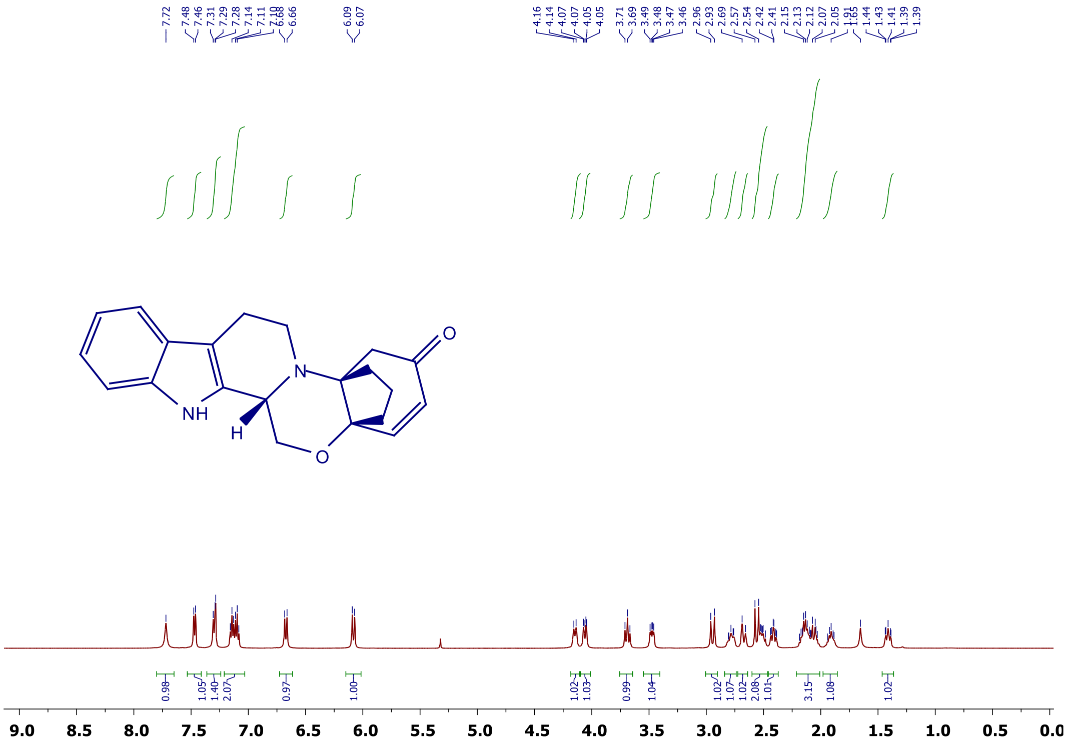

Supplementary Figure 89 ^{1}H NMR (500 MHz, $CDCl_3$) spectrum of (2aS,6aR,14bS)-8,9,14,14b-tetrahydro-1H-2a,6a-propanobenzo[5',6'][1,4]oxazino[4',3':1,2]pyrido[3,4-b]indol-5(6H)-one (**13**)

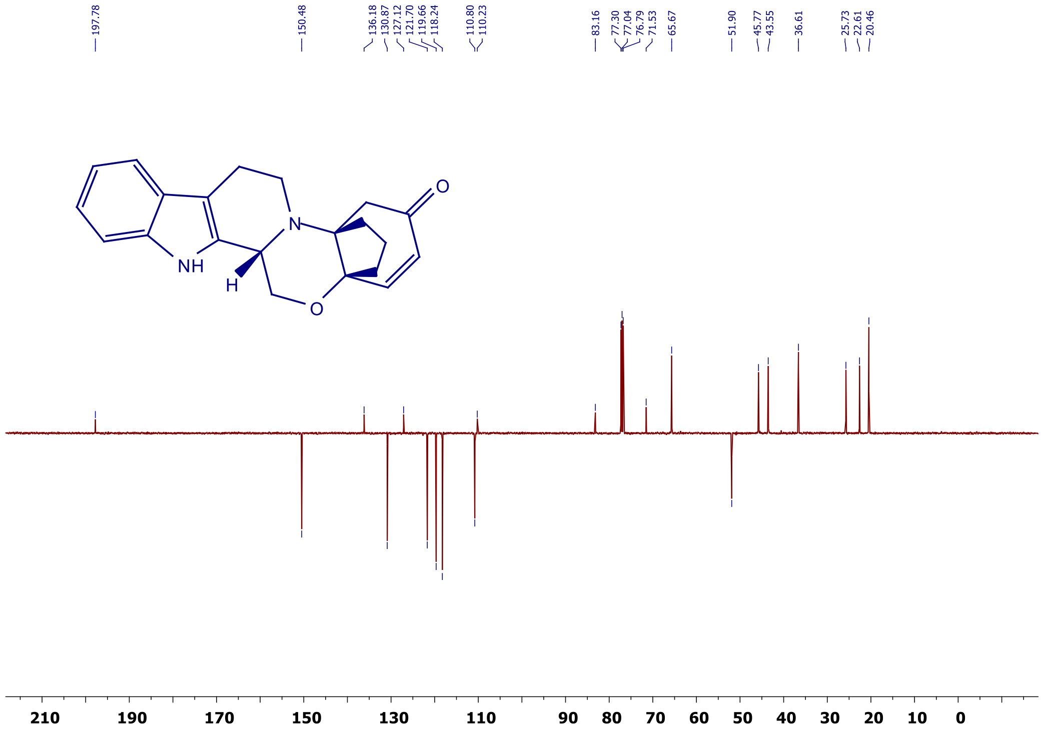

Supplementary Figure 90 APT NMR (125 MHz, $CDCl_3$) spectrum of (2aS,6aR,14bS)-8,9,14,14b-tetrahydro-1H-2a,6a-propanobenzo[5',6'][1,4]oxazino[4',3':1,2]pyrido[3,4-b]indol-5(6H)-one (**13**)

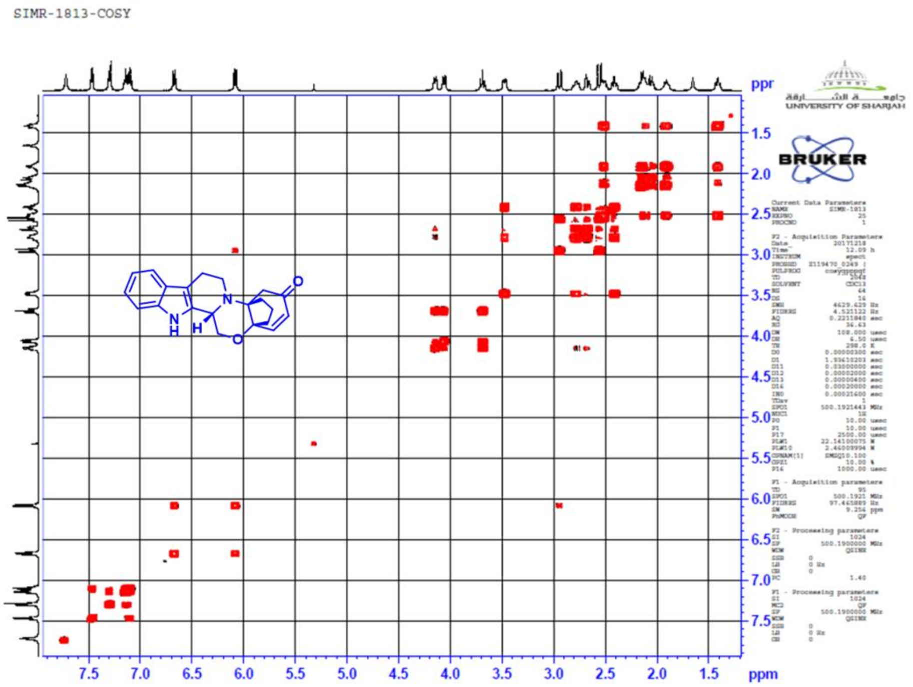

Supplementary Figure 91 COSY (500 MHz, $CDCl_3$) spectrum of (2aS,6aR,14bS)-8,9,14,14b-tetrahydro-1H-2a,6a-propanobenzo[5',6'][1,4]oxazino[4',3':1,2]pyrido[3,4-b]indol-5(6H)-one (**13**)

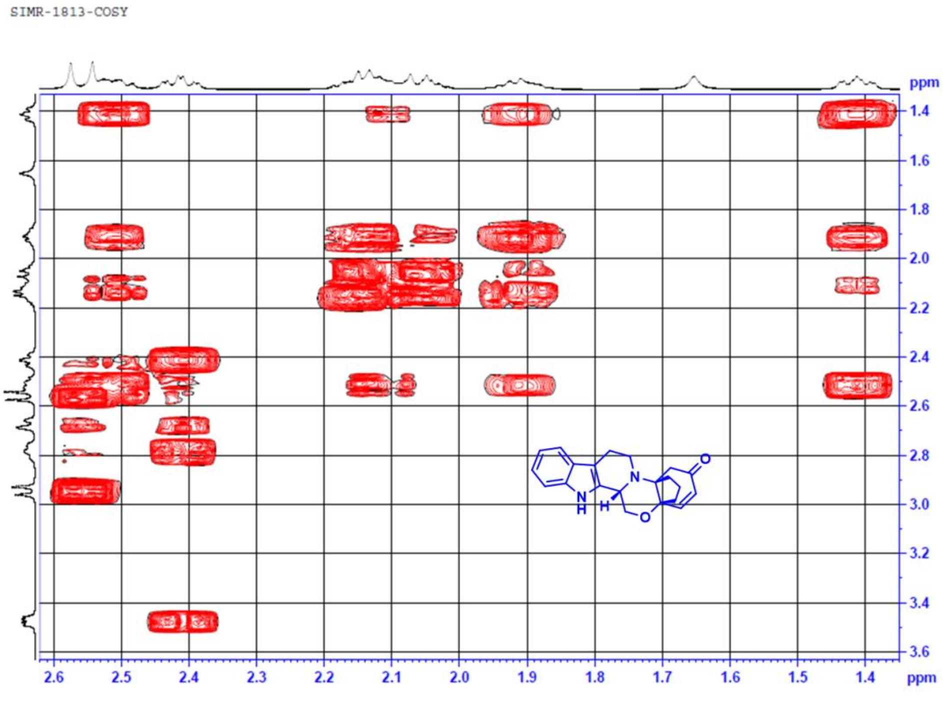

Supplementary Figure 92 COSY (Expansion) spectrum of (2aS,6aR,14bS)-8,9,14,14b-tetrahydro-1H-2a,6a-propanobenzo[5',6'][1,4]oxazino[4',3':1,2]pyrido[3,4-b]indol-5(6H)-one (**13**)

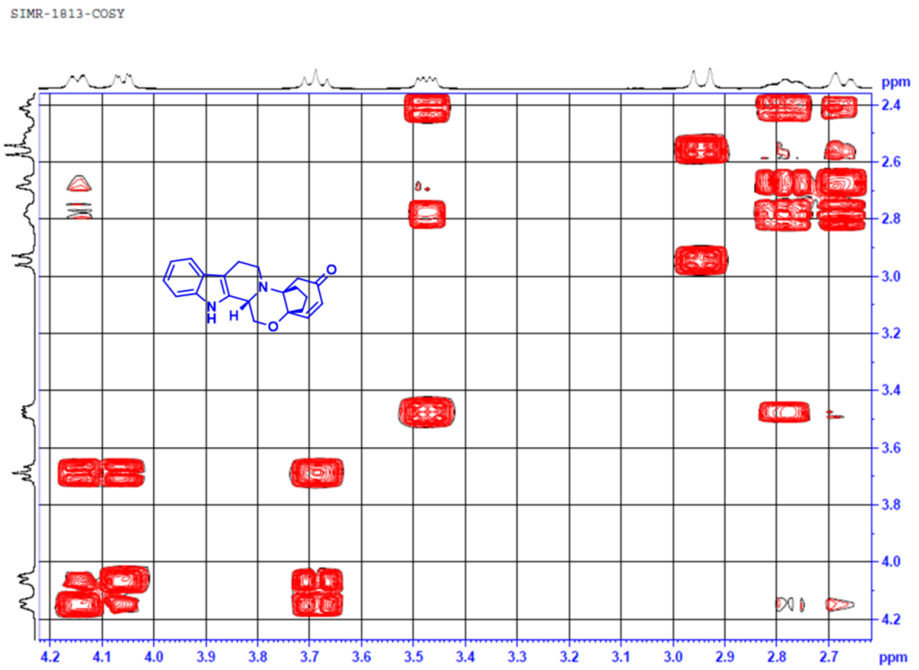

Supplementary Figure 93 COSY (Expansion) spectrum of (2aS,6aR,14bS)-8,9,14,14b-tetrahydro-1H-2a,6a-propanobenzo[5',6'][1,4]oxazino[4',3':1,2]pyrido[3,4-b]indol-5(6H)-one (**13**)

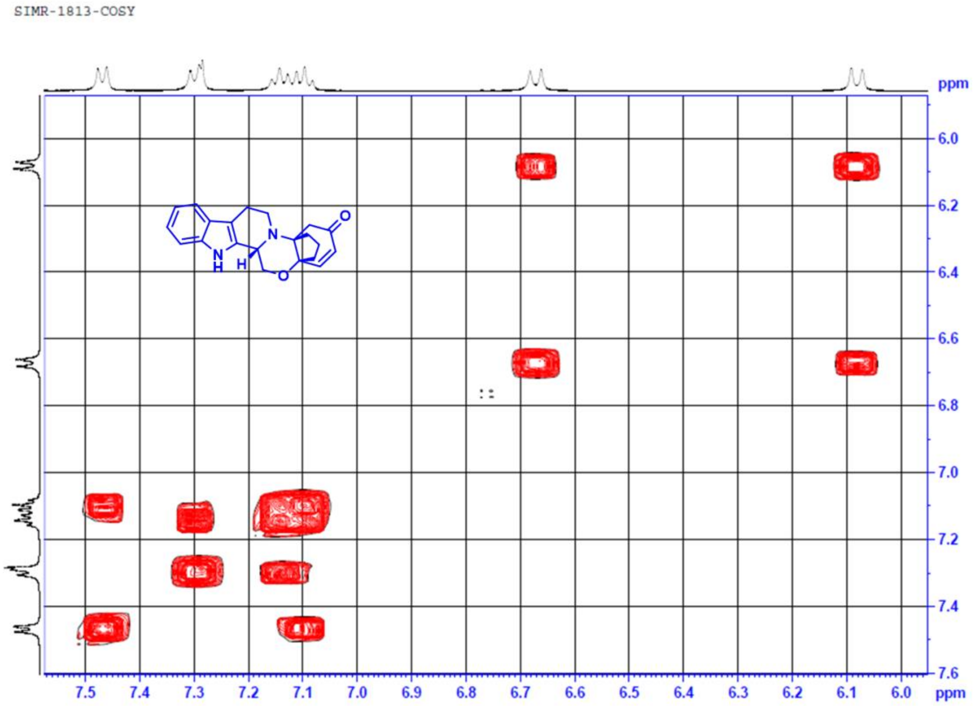

Supplementary Figure 94 COSY (Expansion) spectrum of (2aS,6aR,14bS)-8,9,14,14b-tetrahydro-1H-2a,6a-propanobenzo[5',6'][1,4]oxazino[4',3':1,2]pyrido[3,4-b]indol-5(6H)-one (**13**)

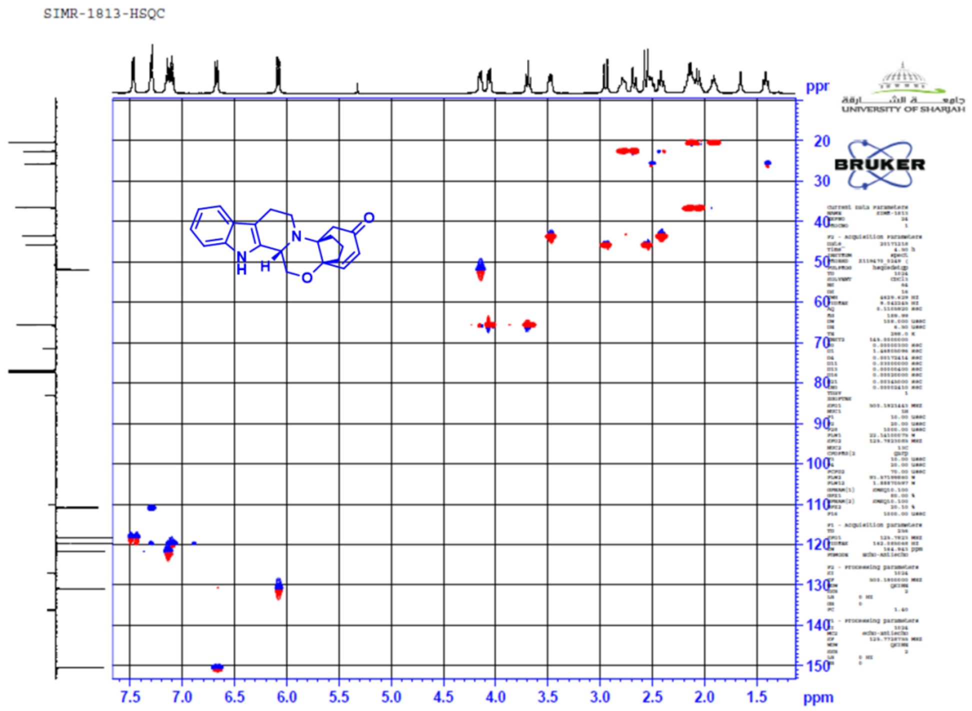

Supplementary Figure 95 HSQC (500 MHz, $CDCl_3$) spectrum of (2aS,6aR,14bS)-8,9,14,14b-tetrahydro-1H-2a,6a-propanobenzo[5',6'][1,4]oxazino[4',3':1,2]pyrido[3,4-b]indol-5(6H)-one (**13**)

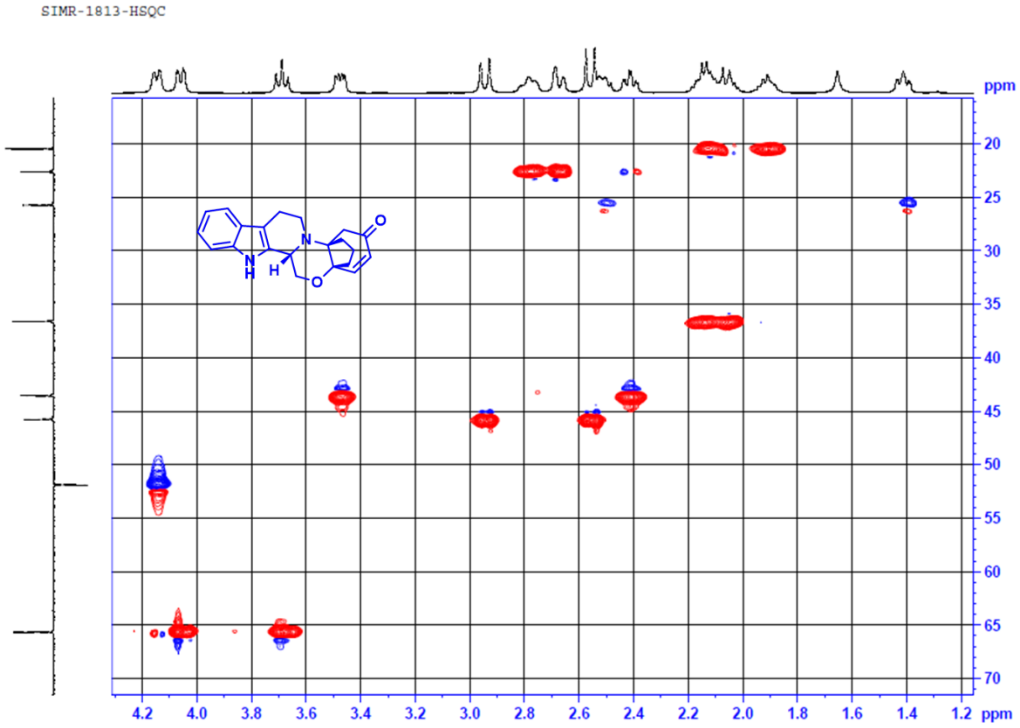

Supplementary Figure 96 HSQC (Expansion) spectrum of (2aS,6aR,14bS)-8,9,14,14b-tetrahydro-1H-2a,6a-propanobenzo[5',6'][1,4]oxazino[4',3':1,2]pyrido[3,4-b]indol-5(6H)-one (**13**)

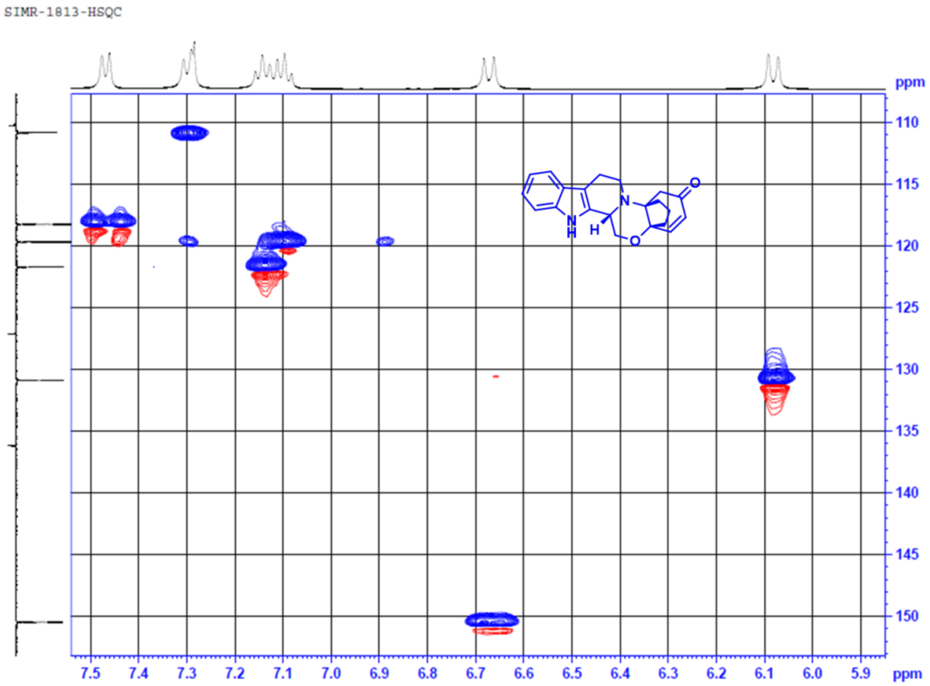

Supplementary Figure 97 HSQC (Expansion) spectrum of (2aS,6aR,14bS)-8,9,14,14b-tetrahydro-1H-2a,6a-propanobenzo[5',6'][1,4]oxazino[4',3':1,2]pyrido[3,4-b]indol-5(6H)-one (**13**)

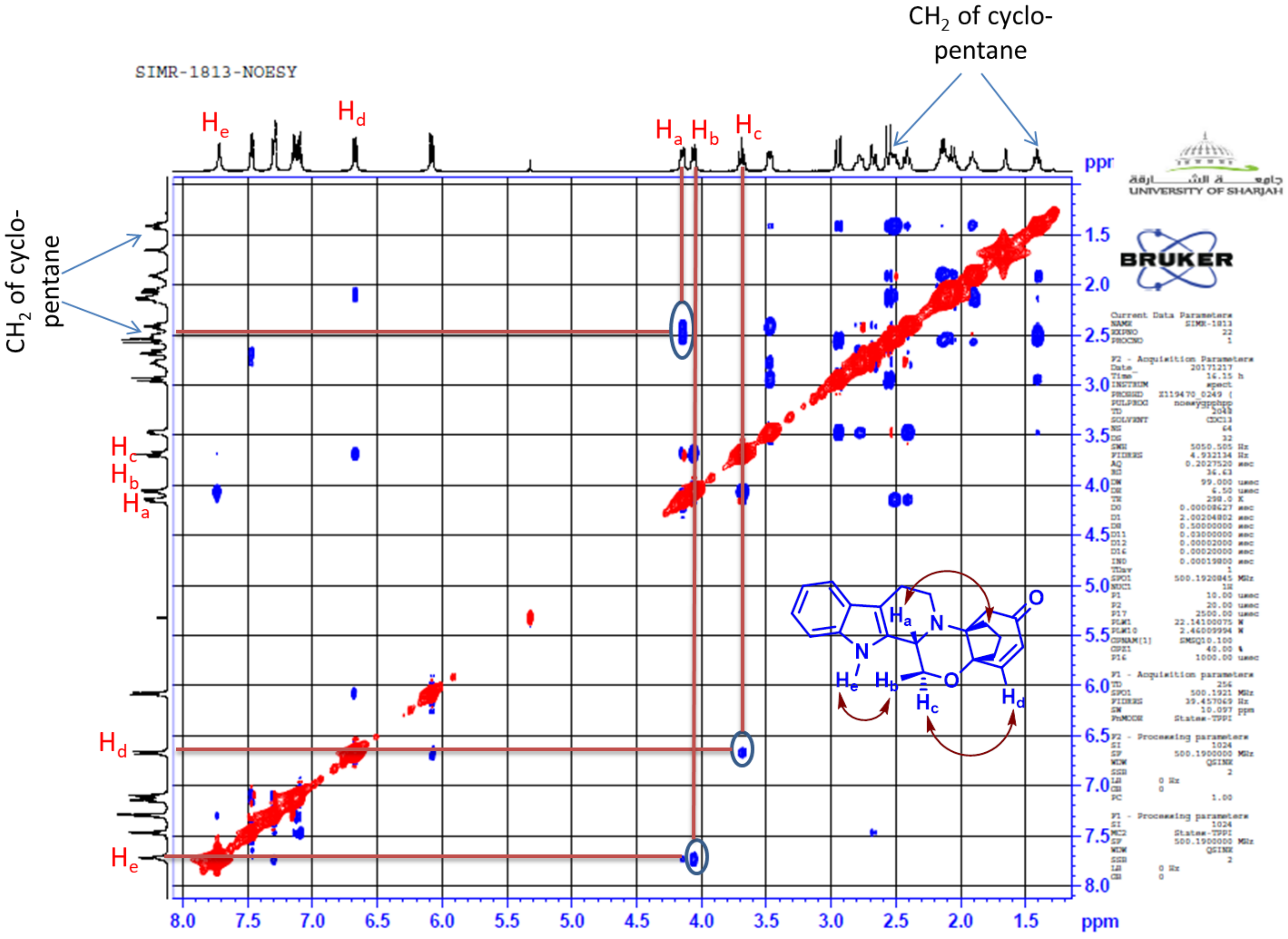

Supplementary Figure 98 NOESY (500 MHz, $CDCl_3$) spectrum of (2aS,6aR,14bS)-8,9,14,14b-tetrahydro-1H-2a,6a-propanobenzo[5',6'][1,4]oxazino[4',3':1,2]pyrido[3,4-b]indol-5(6H)-one (**13**)

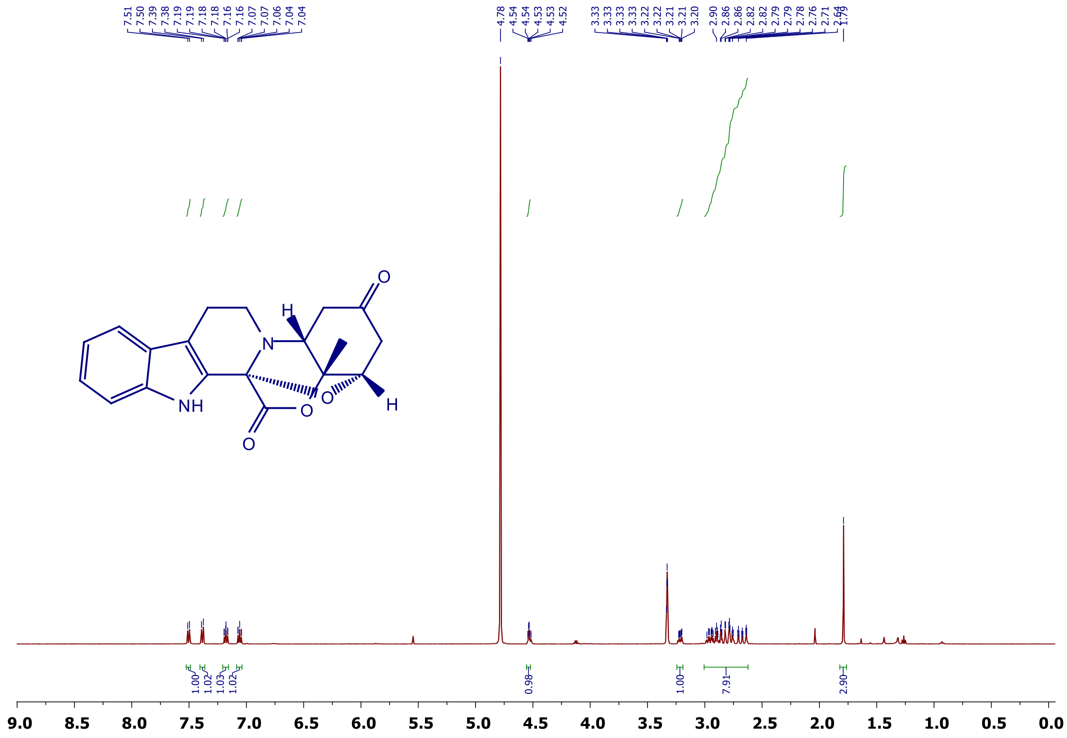

Supplementary Figure 99 ^{1}H NMR (500 MHz, CD_3OD) spectrum of (2aR,3S,6aR,14bS)-2a-methyl-3,4,6,6a,9,14-hexahydro-1H,8H-3,14b-epoxybenzo[5',6'][1,4]oxazino[4',3':1,2]pyrido[3,4-b]indole-1,5(2aH)-dione (**14**)

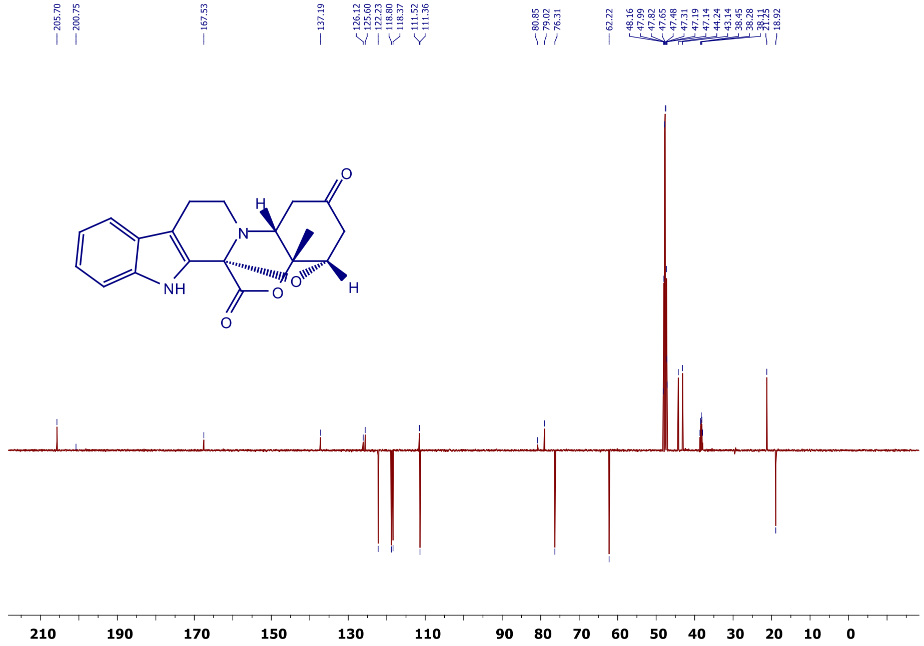

Supplementary Figure 100 APT NMR (125 MHz, CD_3OD) spectrum of (2aR,3S,6aR,14bS)-2a-methyl-3,4,6,6a,9,14-hexahydro-1H,8H-3,14b-epoxybenzo[5',6'][1,4]oxazino[4',3':1,2]pyrido[3,4-b]indole-1,5(2aH)-dione (**14**)

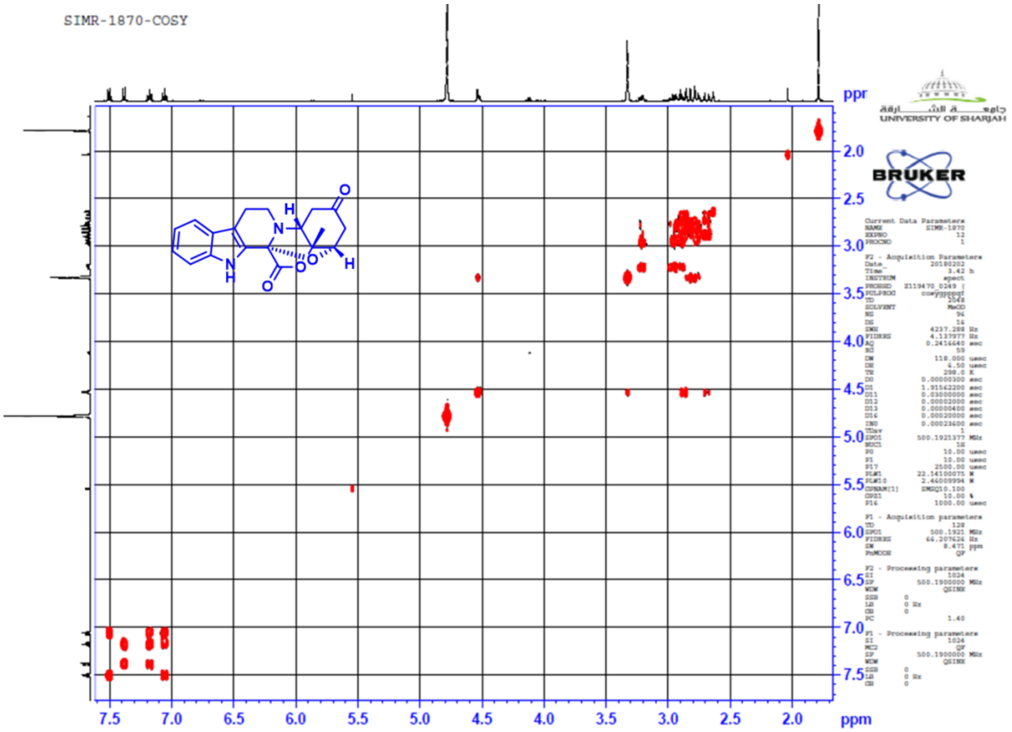

Supplementary Figure 101 COSY (500 MHz, CD_3OD) spectrum of (2aR,3S,6aR,14bS)-2a-methyl-3,4,6,6a,9,14-hexahydro-1H,8H-3,14b-epoxybenzo[5',6'][1,4]oxazino[4',3':1,2]pyrido[3,4-b]indole-1,5(2aH)-dione (**14**)

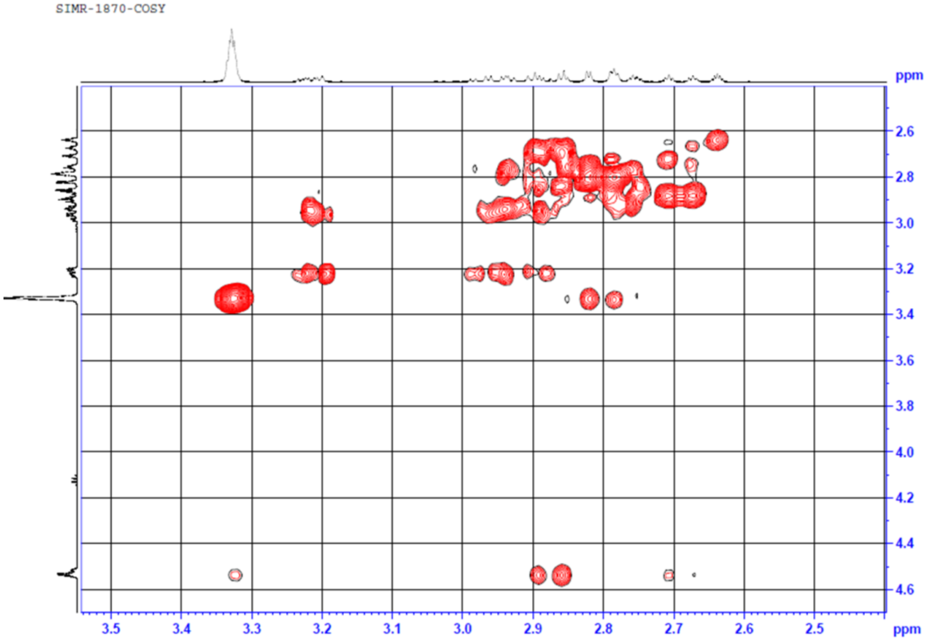

Supplementary Figure 102 COSY (Expansion) spectrum of (2aR,3S,6aR,14bS)-2a-methyl-3,4,6,6a,9,14-hexahydro-1H,8H-3,14b-epoxybenzo[5',6'][1,4]oxazino[4',3':1,2]pyrido[3,4-b]indole-1,5(2aH)-dione (**14**)

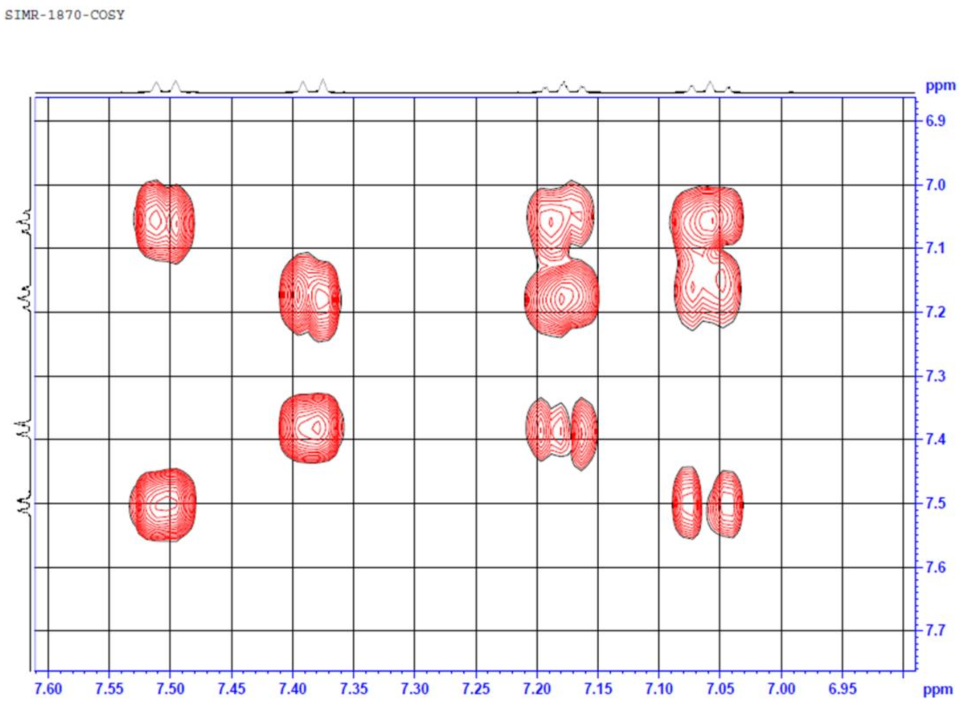

Supplementary Figure 103 COSY (Expansion) spectrum of (2aR,3S,6aR,14bS)-2a-methyl-3,4,6,6a,9,14-hexahydro-1H,8H-3,14b-epoxybenzo[5',6'][1,4]oxazino[4',3':1,2]pyrido[3,4-b]indole-1,5(2aH)-dione (**14**)

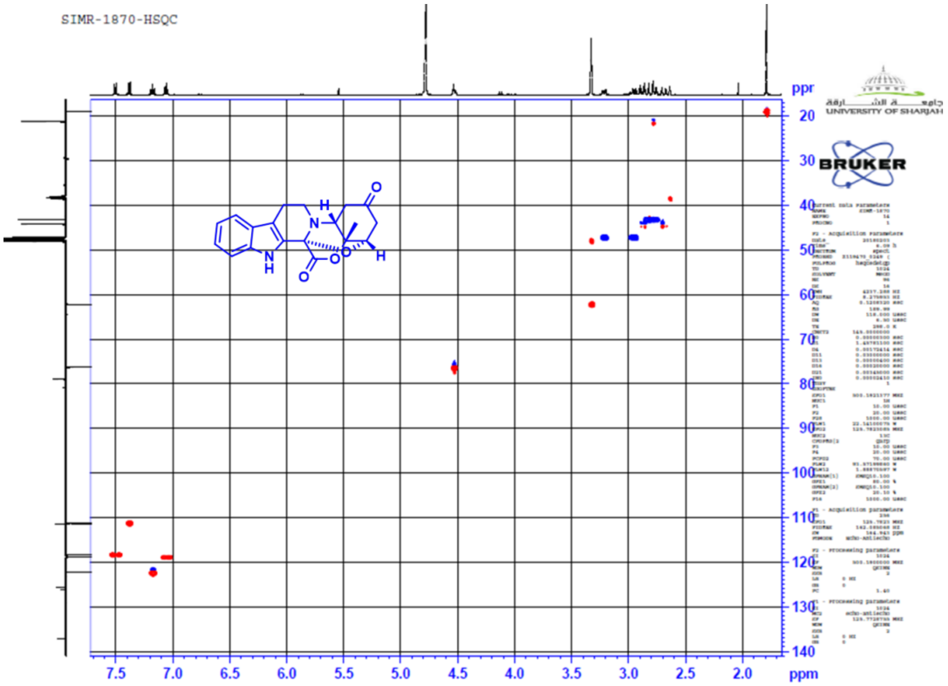

Supplementary Figure 104 HSQC (500 MHz, CD_3OD) spectrum of (2aR,3S,6aR,14bS)-2a-methyl-3,4,6,6a,9,14-hexahydro-1H,8H-3,14b-epoxybenzo[5',6'][1,4]oxazino[4',3':1,2]pyrido[3,4-b]indole-1,5(2aH)-dione (**14**)

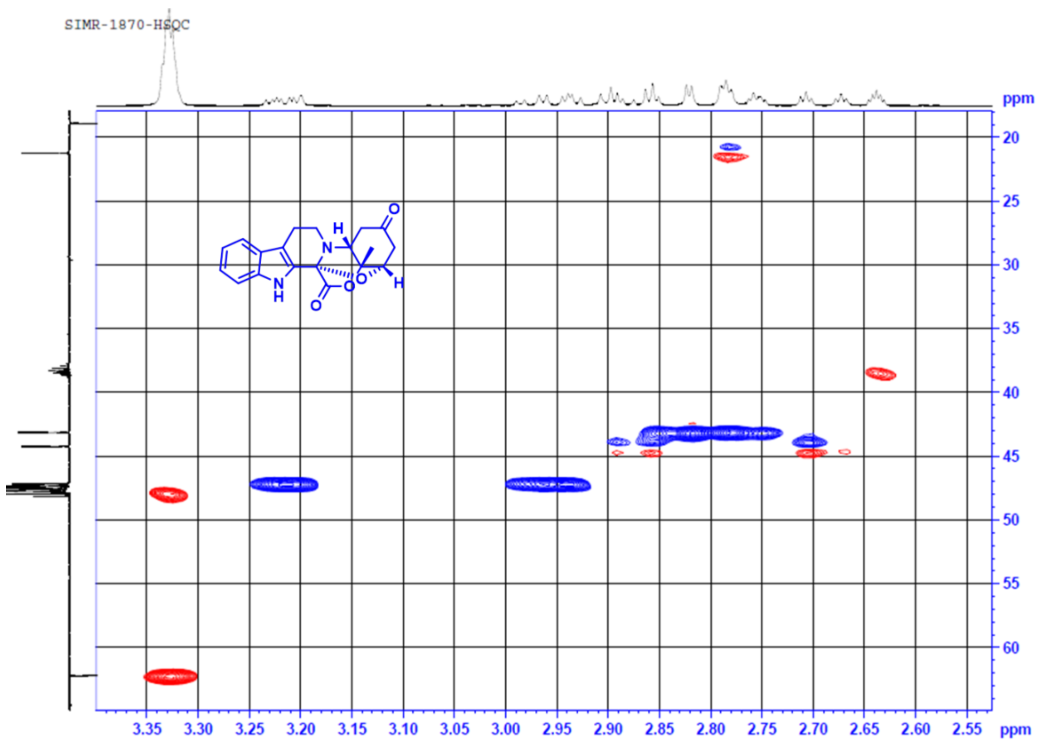

Supplementary Figure 105 HSQC (Expansion) spectrum of (2aR,3S,6aR,14bS)-2a-methyl-3,4,6,6a,9,14-hexahydro-1H,8H-3,14b-epoxybenzo[5',6'][1,4]oxazino[4',3':1,2]pyrido[3,4-b]indole-1,5(2aH)-dione (**14**)

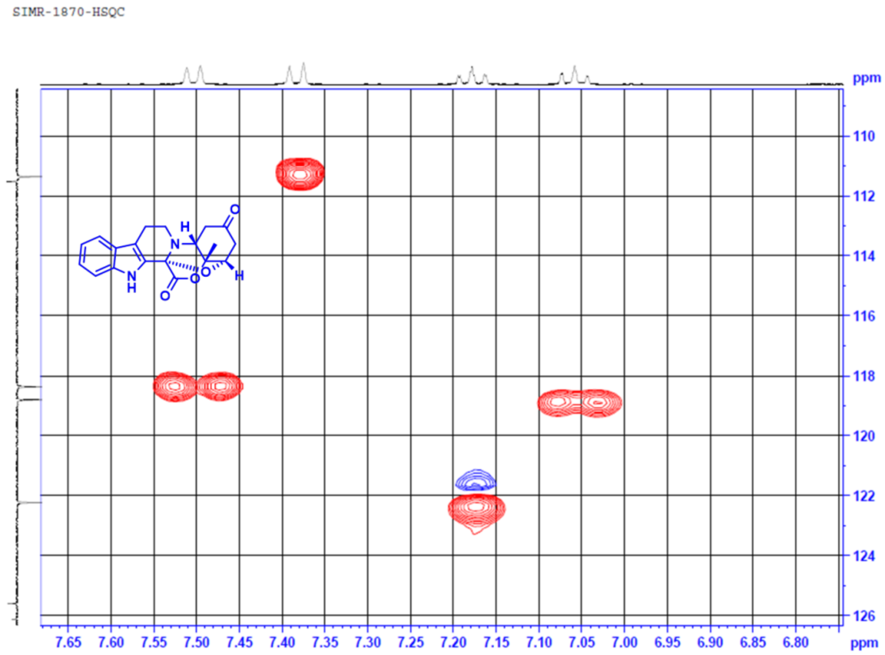

Supplementary Figure 106 HSQC (Expansion) spectrum of (2aR,3S,6aR,14bS)-2a-methyl-3,4,6,6a,9,14-hexahydro-1H,8H-3,14b-epoxybenzo[5',6'][1,4]oxazino[4',3':1,2]pyrido[3,4-b]indole-1,5(2aH)-dione (**14**)

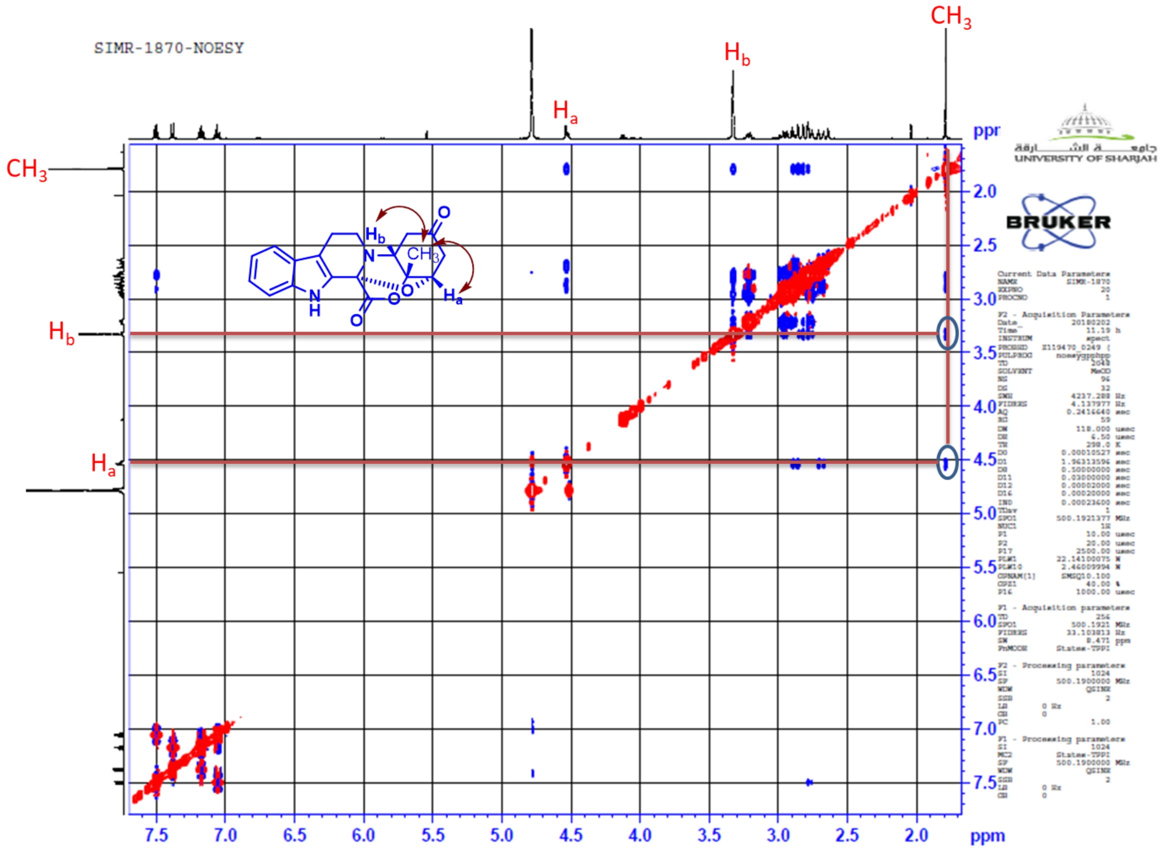

Supplementary Figure 107 NOESY (500 MHz, CD_3OD) spectrum of (2aR,3S,6aR,14bS)-2a-methyl-3,4,6,6a,9,14-hexahydro-1H,8H-3,14b-epoxybenzo[5',6'][1,4]oxazino[4',3':1,2]pyrido[3,4-b]indole-1,5(2aH)-dione (**14**)

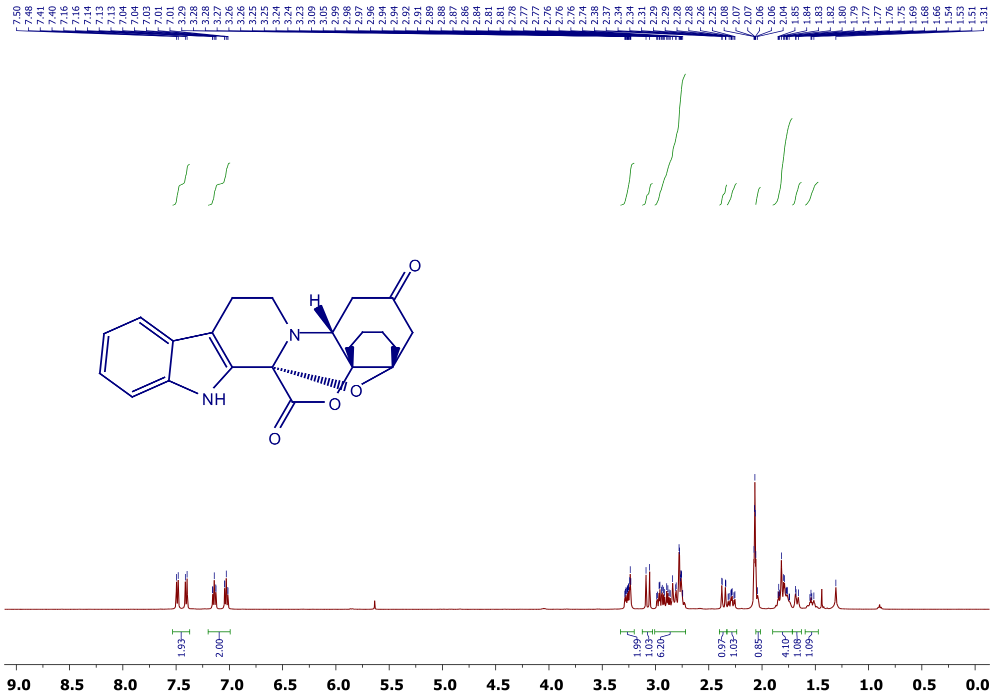

Supplementary Figure 108 ^{1}H NMR (500 MHz, Acetone-d_6) spectrum of (2aR,6aS,9aR,17bS)-3,4,5,6,9,9a,12,17-octahydro-1H,11H-6a,17b-epoxynaphtho[1'',8a'':5',6'][1,4]oxazino[4',3':1,2]pyrido[3,4-b]indole-1,8(7H)-dione (**15**)

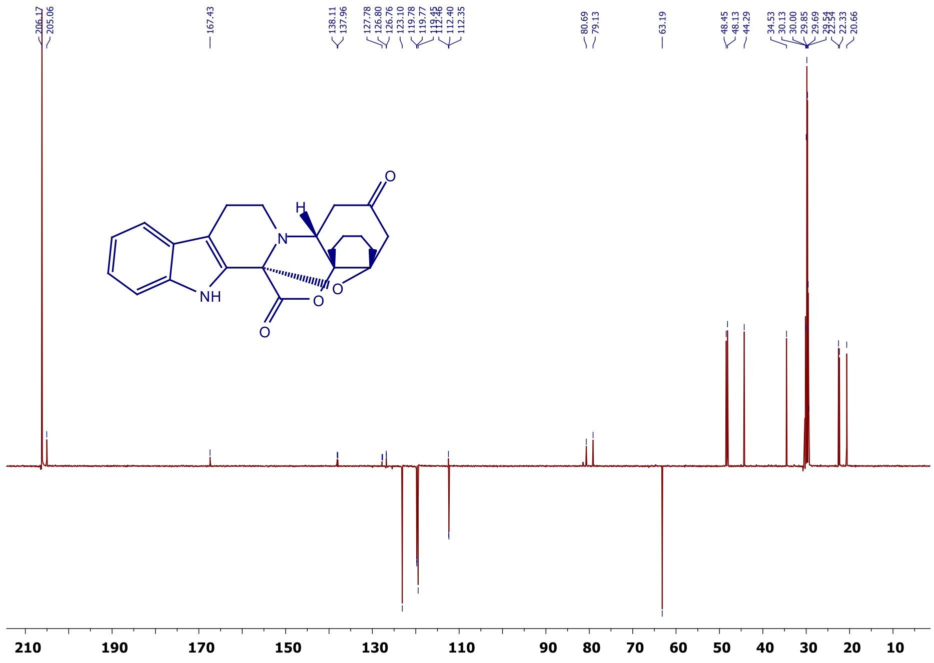

Supplementary Figure 109 APT NMR (125 MHz, Acetone-d_6) spectrum of (2aR,6aS,9aR,17bS)-3,4,5,6,9,9a,12,17-octahydro-1H,11H-6a,17b-epoxynaphtho[1'',8a'':5',6'][1,4]oxazino[4',3':1,2]pyrido[3,4-b]indole-1,8(7H)-dione (**15**)

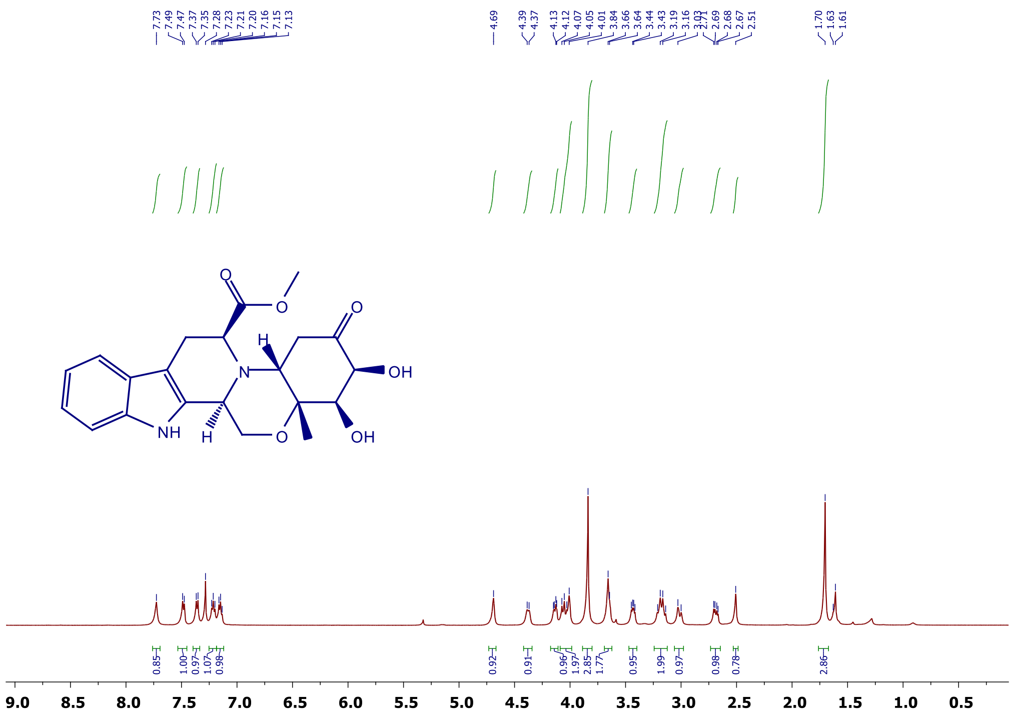

Supplementary Figure 110 ^{1}H (500 MHz, $CDCl_3$) spectrum of methyl (2aR,3R,4S,6aR,8S,14bR)-3,4-dihydroxy-2a-methyl-5-oxo-2a,3,4,5,6,6a,8,9,14,14b-decahydro-1H-benzo[5',6'][1,4]oxazino[4',3':1,2]pyrido[3,4-b]indole-8-carboxylate (**16**)

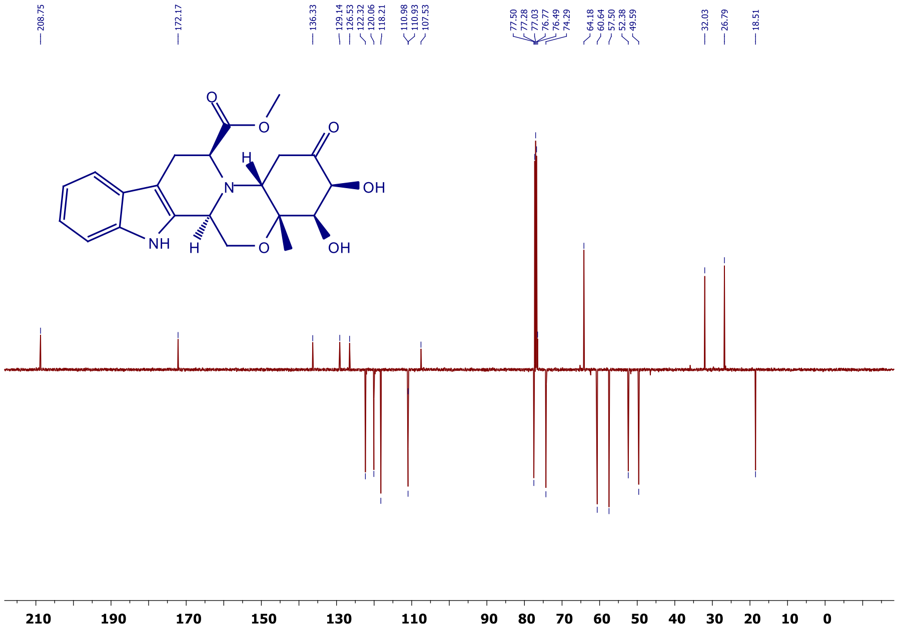

Supplementary Figure 111 APT (125 MHz, $CDCl_3$) spectrum of methyl (2aR,3R,4S,6aR,8S,14bR)-3,4-dihydroxy-2a-methyl-5-oxo-2a,3,4,5,6,6a,8,9,14,14b-decahydro-1H-benzo[5',6'][1,4]oxazino[4',3':1,2]pyrido[3,4-b]indole-8-carboxylate (**16**)

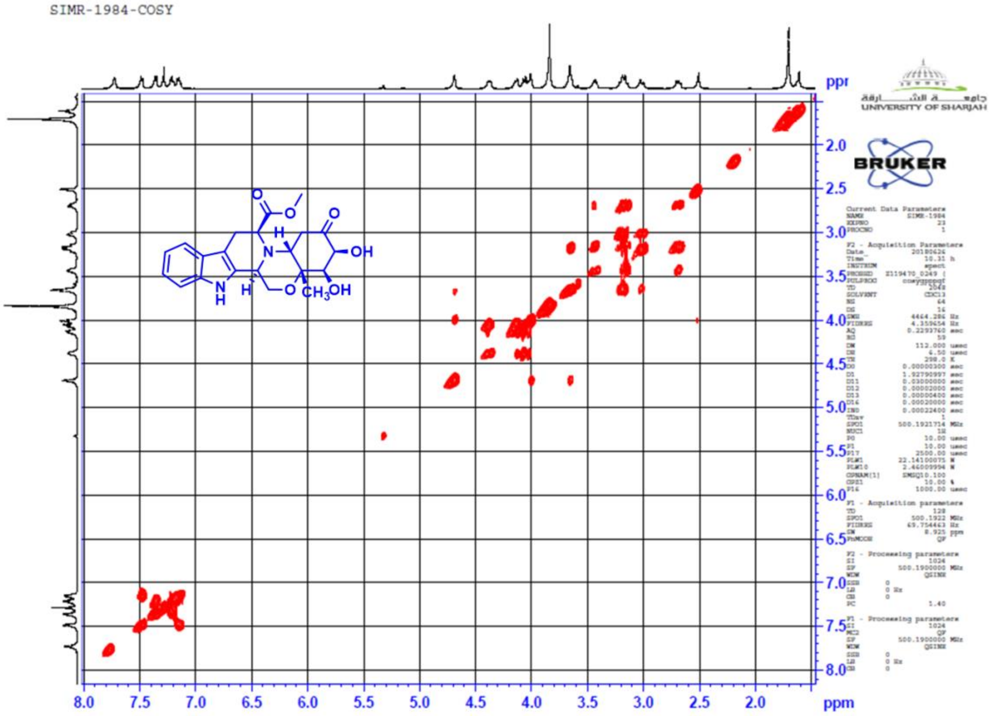

Supplementary Figure 112 COSY (500 MHz, $CDCl_3$) spectrum of methyl (2aR,3R,4S,6aR,8S,14bR)-3,4-dihydroxy-2a-methyl-5-oxo-2a,3,4,5,6,6a,8,9,14,14b-decahydro-1H-benzo[5',6'][1,4]oxazino[4',3':1,2]pyrido[3,4-b]indole-8-carboxylate (**16**)

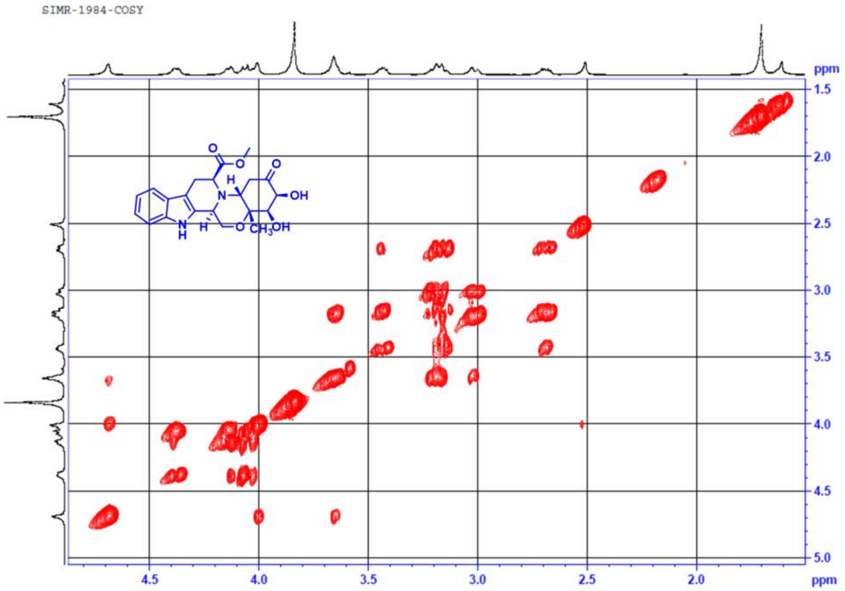

Supplementary Figure 113 COSY (Expansion) spectrum of methyl (2aR,3R,4S,6aR,8S,14bR)-3,4-dihydroxy-2a-methyl-5-oxo-2a,3,4,5,6,6a,8,9,14,14b-decahydro-1H-benzo[5',6'][1,4]oxazino[4',3':1,2]pyrido[3,4-b]indole-8-carboxylate (**16**)

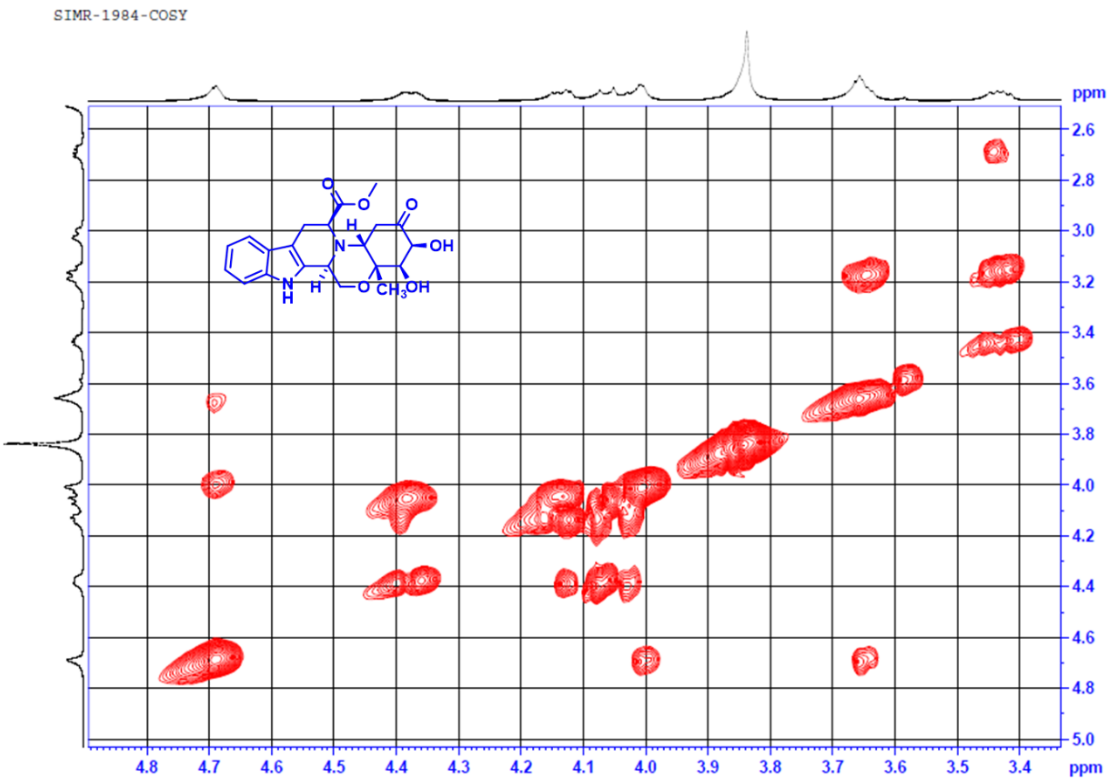

Supplementary Figure 114 COSY (Expansion) spectrum of methyl (2aR,3R,4S,6aR,8S,14bR)-3,4-dihydroxy-2a-methyl-5-oxo-2a,3,4,5,6,6a,8,9,14,14b-decahydro-1H-benzo[5',6'][1,4]oxazino[4',3':1,2]pyrido[3,4-b]indole-8-carboxylate (**16**)

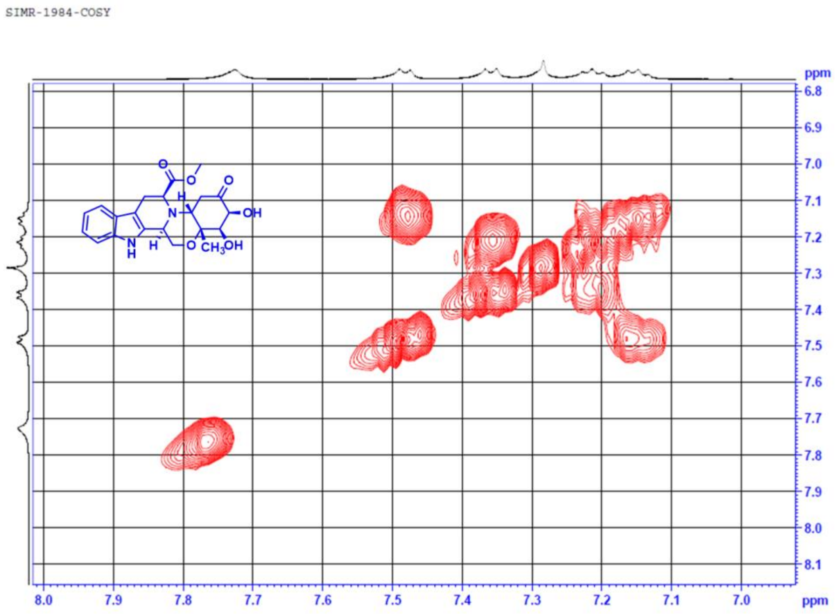

Supplementary Figure 115 COSY (Expansion) spectrum of methyl (2aR,3R,4S,6aR,8S,14bR)-3,4-dihydroxy-2a-methyl-5-oxo-2a,3,4,5,6,6a,8,9,14,14b-decahydro-1H-benzo[5',6'][1,4]oxazino[4',3':1,2]pyrido[3,4-b]indole-8-carboxylate (**16**)

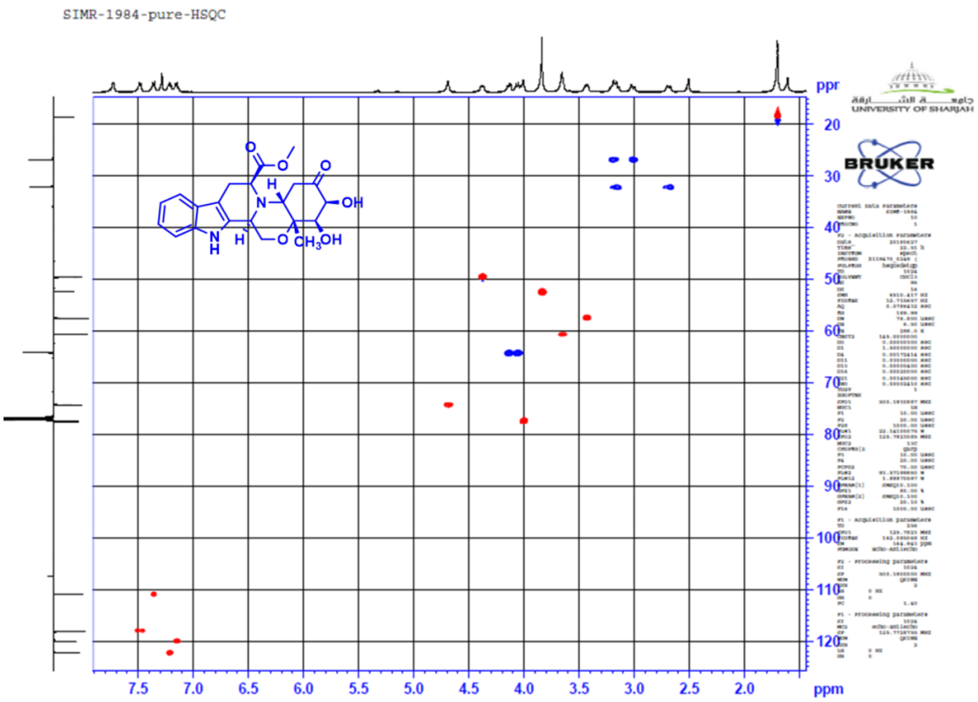

Supplementary Figure 116 HSQC (500 MHz, $CDCl_3$) spectrum of methyl (2aR,3R,4S,6aR,8S,14bR)-3,4-dihydroxy-2a-methyl-5-oxo-2a,3,4,5,6,6a,8,9,14,14b-decahydro-1H-benzo[5',6'][1,4]oxazino[4',3':1,2]pyrido[3,4-b]indole-8-carboxylate (**16**)

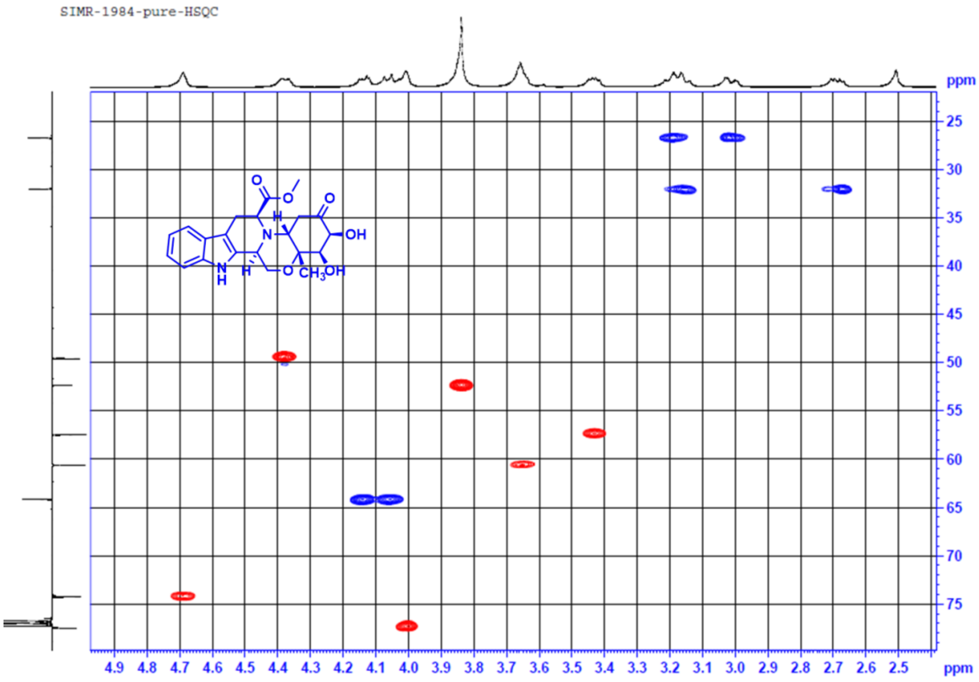

Supplementary Figure 117 HSQC (Expansion) spectrum of methyl (2aR,3R,4S,6aR,8S,14bR)-3,4-dihydroxy-2a-methyl-5-oxo-2a,3,4,5,6,6a,8,9,14,14b-decahydro-1H-benzo[5',6'][1,4]oxazino[4',3':1,2]pyrido[3,4-b]indole-8-carboxylate (**16**)

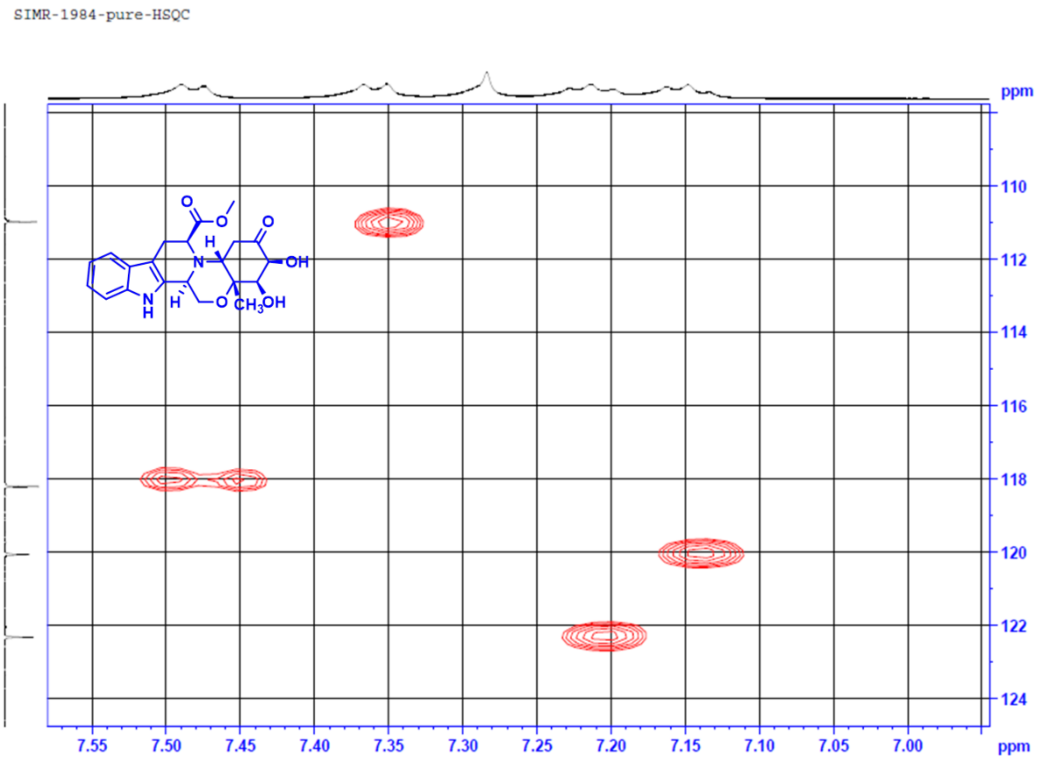

Supplementary Figure 118 HSQC (Expansion) spectrum of methyl (2aR,3R,4S,6aR,8S,14bR)-3,4-dihydroxy-2a-methyl-5-oxo-2a,3,4,5,6,6a,8,9,14,14b-decahydro-1H-benzo[5',6'][1,4]oxazino[4',3':1,2]pyrido[3,4-b]indole-8-carboxylate (**16**)

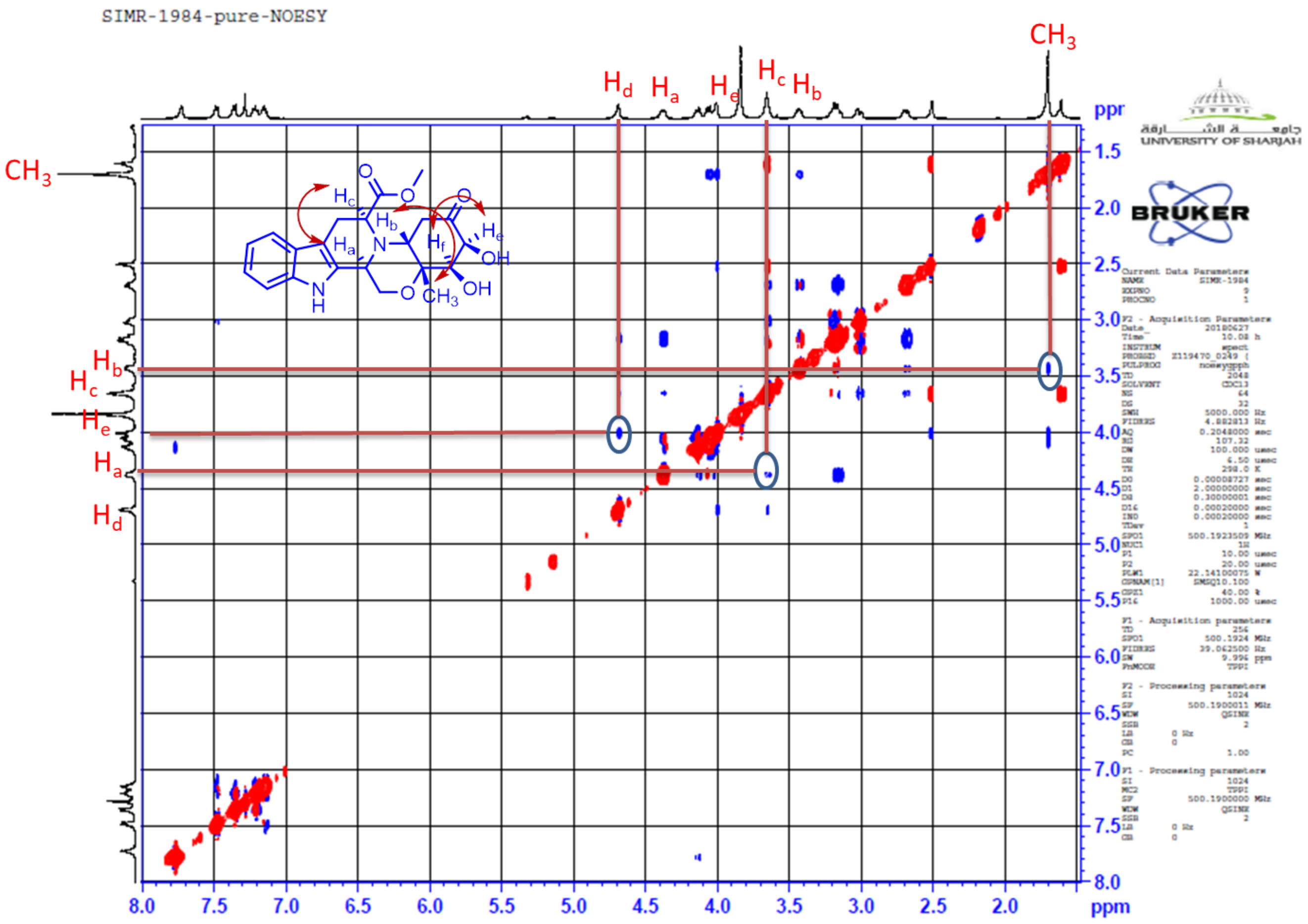

Supplementary Figure 119 NOESY (500 MHz, $CDCl_3$) spectrum of methyl (2aR,3R,4S,6aR,8S,14bR)-3,4-dihydroxy-2a-methyl-5-oxo-2a,3,4,5,6,6a,8,9,14,14b-decahydro-1H-benzo[5',6'][1,4]oxazino[4',3':1,2]pyrido[3,4-b]indole-8-carboxylate (**16**)

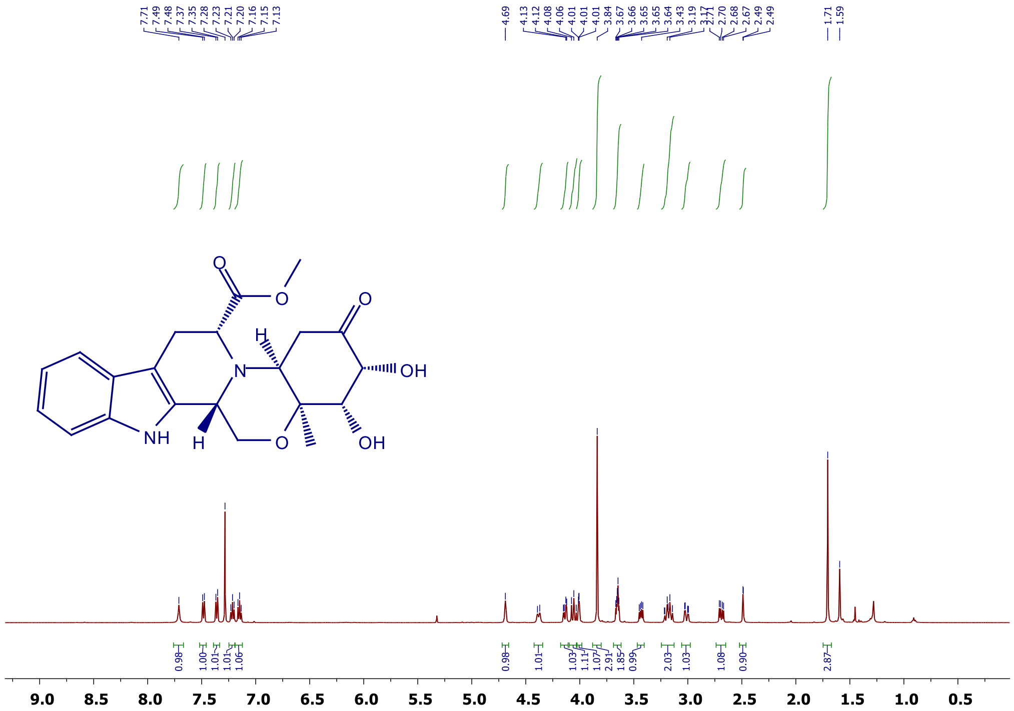

Supplementary Figure 120 ^{1}H (500 MHz, $CDCl_3$) spectrum of methyl (2aS,3S,4R,6aS,8R,14bS)-3,4-dihydroxy-2a-methyl-5-oxo-2a,3,4,5,6,6a,8,9,14,14b-decahydro-1H-benzo[5',6'][1,4]oxazino[4',3':1,2]pyrido[3,4-b]indole-8-carboxylate (**17**)

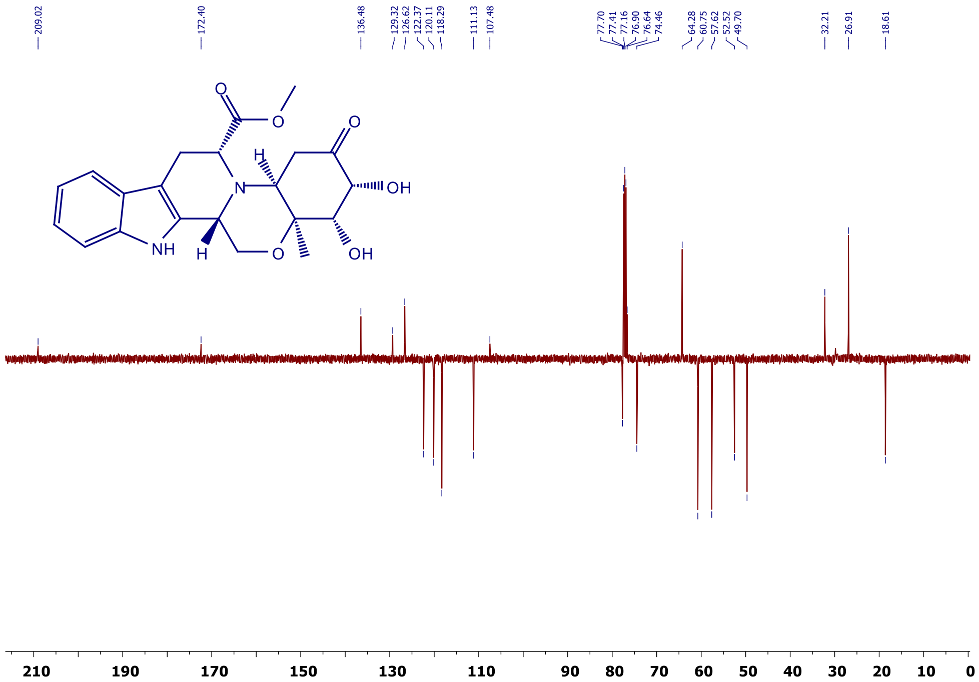

Supplementary Figure 121 APT NMR (125 MHz, $CDCl_3$) spectrum of methyl (2aS,3S,4R,6aS,8R,14bS)-3,4-dihydroxy-2a-methyl-5-oxo-2a,3,4,5,6,6a,8,9,14,14b-decahydro-1H-benzo[5',6'][1,4]oxazino[4',3':1,2]pyrido[3,4-b]indole-8-carboxylate (**17**)

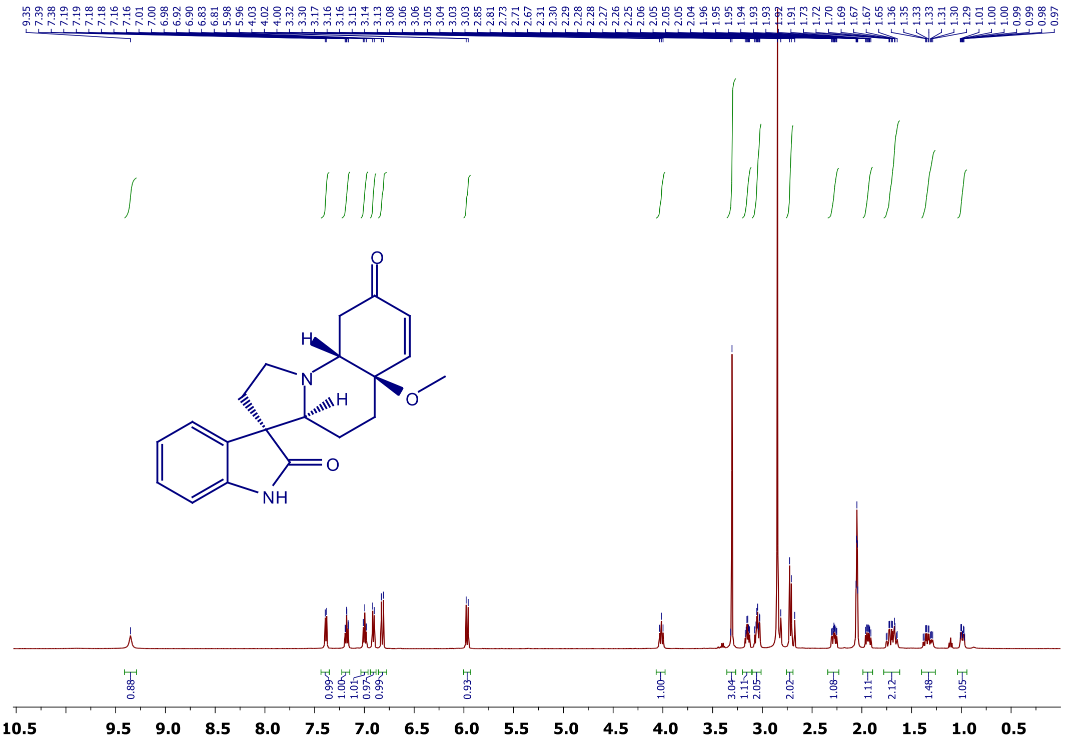

Supplementary Figure 122 ^{1}H (500 MHz, Acetone-d_6) spectrum of (3S,3a'S,5a'R,9a'R)-5a'-methoxy-1',2',3a',4',5',5a',9',9a'-octahydro-8'H-spiro[indoline-3,3'-pyrrolo[1,2-a]quinoline]-2,8'-dione (**18**)

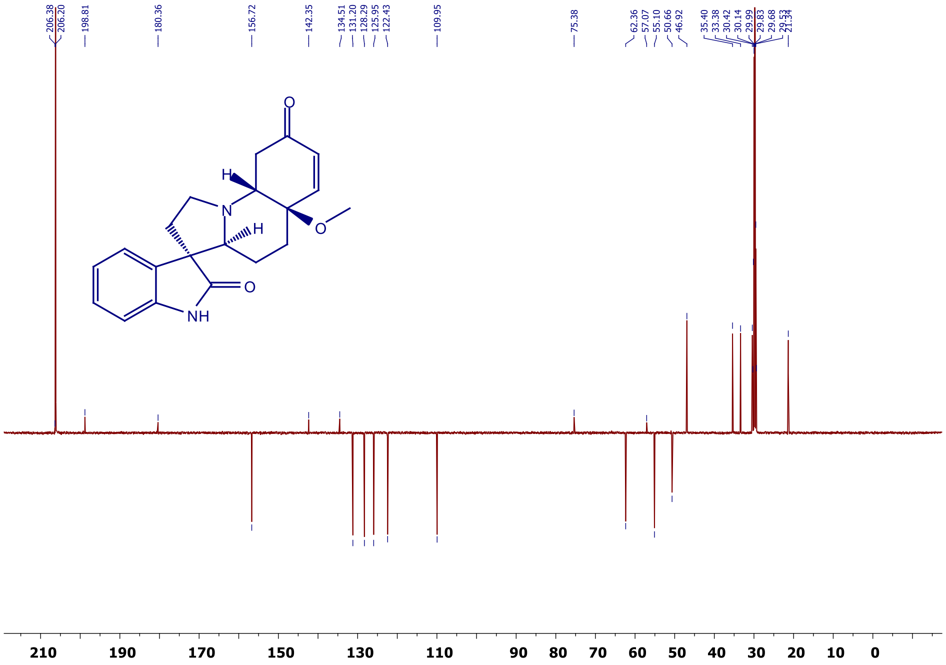

Supplementary Figure 123 APT NMR (500 MHz, Acetone-d_6) spectrum of (3S,3a'S,5a'R,9a'R)-5a'-methoxy-1',2',3a',4',5',5a',9',9a'-octahydro-8'H-spiro[indoline-3,3'-pyrrolo[1,2-a]quinoline]-2,8'-dione (**18**)

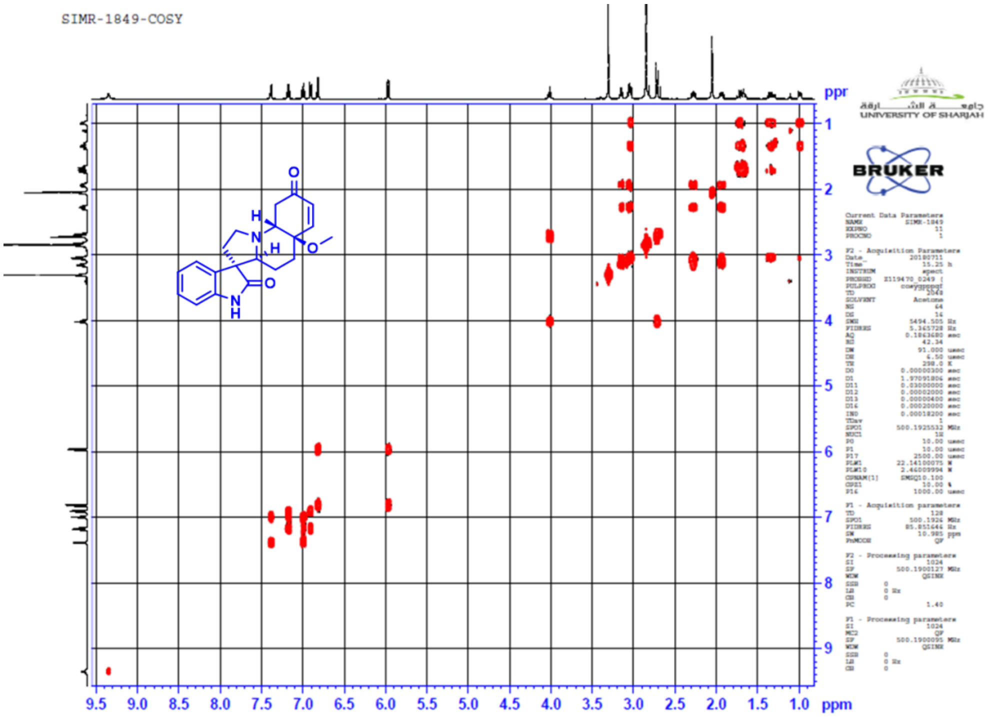

Supplementary Figure 124 COSY (500 MHz, Acetone-d_6) spectrum of (3S,3a'S,5a'R,9a'R)-5a'-methoxy-1',2',3a',4',5',5a',9',9a'-octahydro-8'H-spiro[indoline-3,3'-pyrrolo[1,2-a]quinoline]-2,8'-dione (**18**)

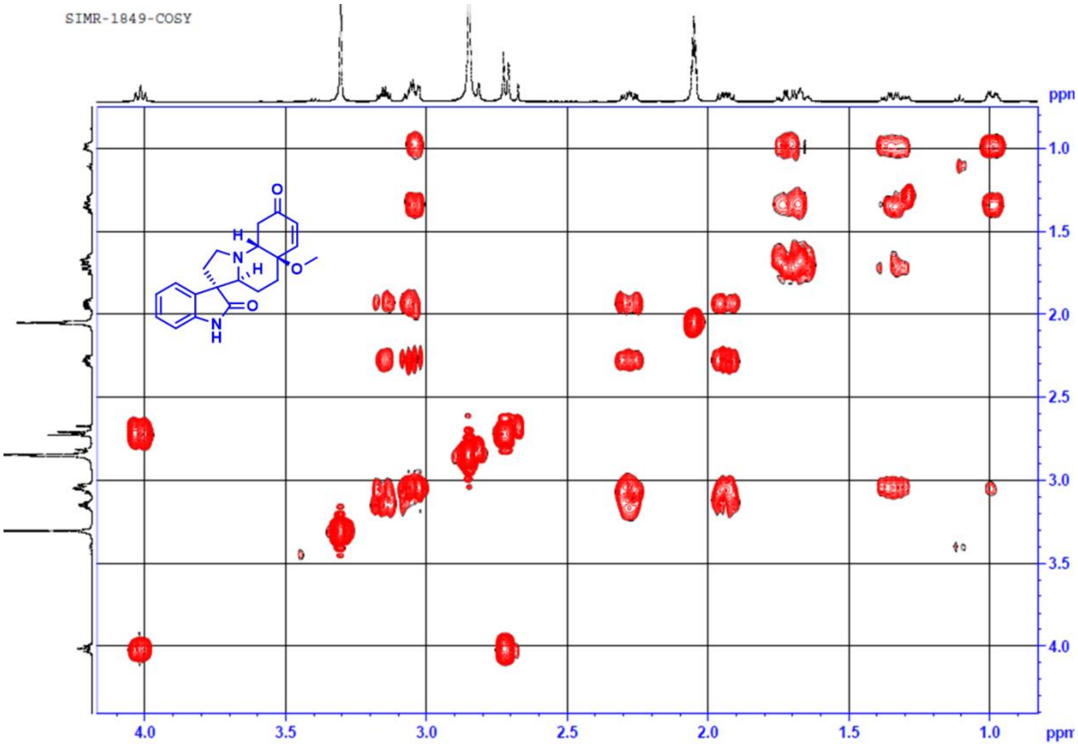

Supplementary Figure 125 COSY (Expansion) spectrum of (3S,3a'S,5a'R,9a'R)-5a'-methoxy-1',2',3a',4',5',5a',9',9a'-octahydro-8'H-spiro[indoline-3,3'-pyrrolo[1,2-a]quinoline]-2,8'-dione (**18**)

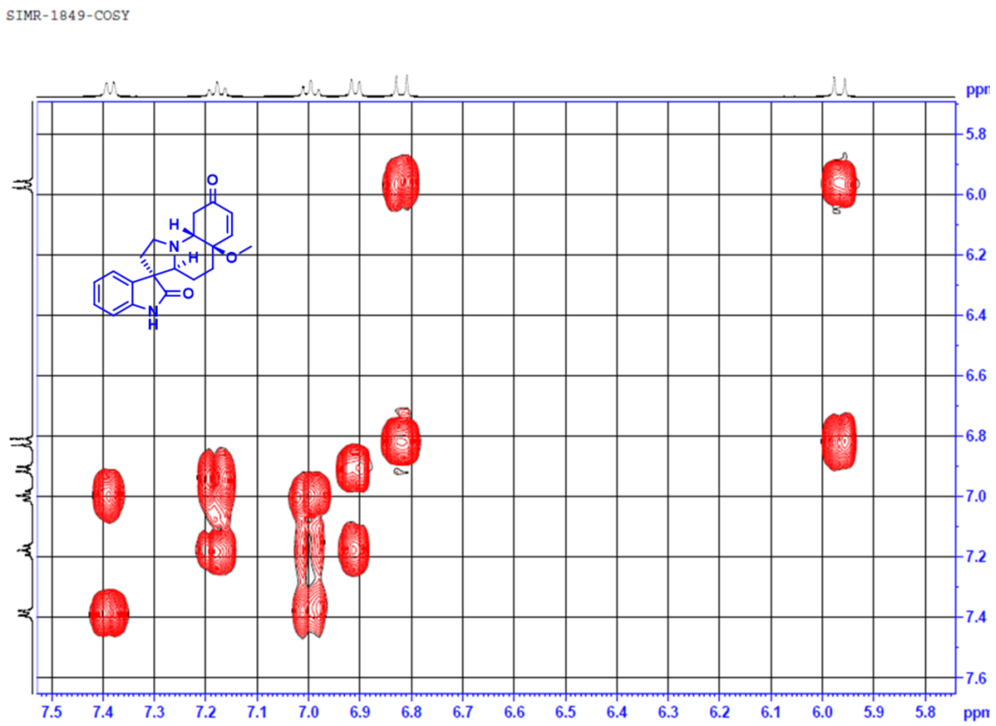

Supplementary Figure 126 COSY (Expansion) spectrum of (3S,3a'S,5a'R,9a'R)-5a'-methoxy-1',2',3a',4',5',5a',9',9a'-octahydro-8'H-spiro[indoline-3,3'-pyrrolo[1,2-a]quinoline]-2,8'-dione (**18**)

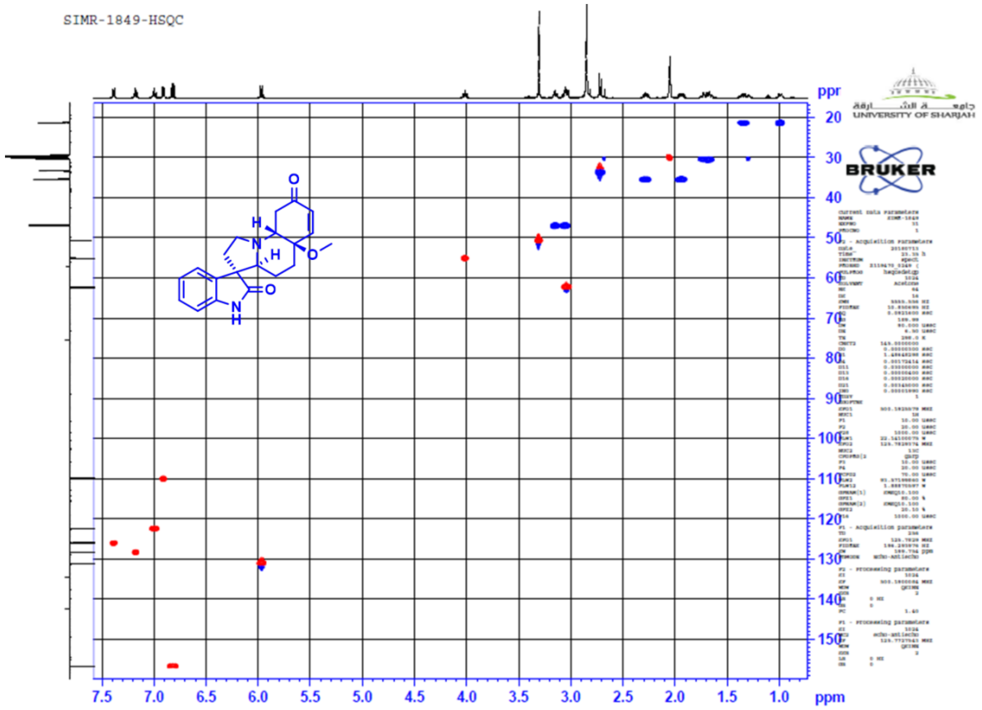

Supplementary Figure 127 HSQC (500 MHz, Acetone-d_6) spectrum of (3S,3a'S,5a'R,9a'R)-5a'-methoxy-1',2',3a',4',5',5a',9',9a'-octahydro-8'H-spiro[indoline-3,3'-pyrrolo[1,2-a]quinoline]-2,8'-dione (**18**)

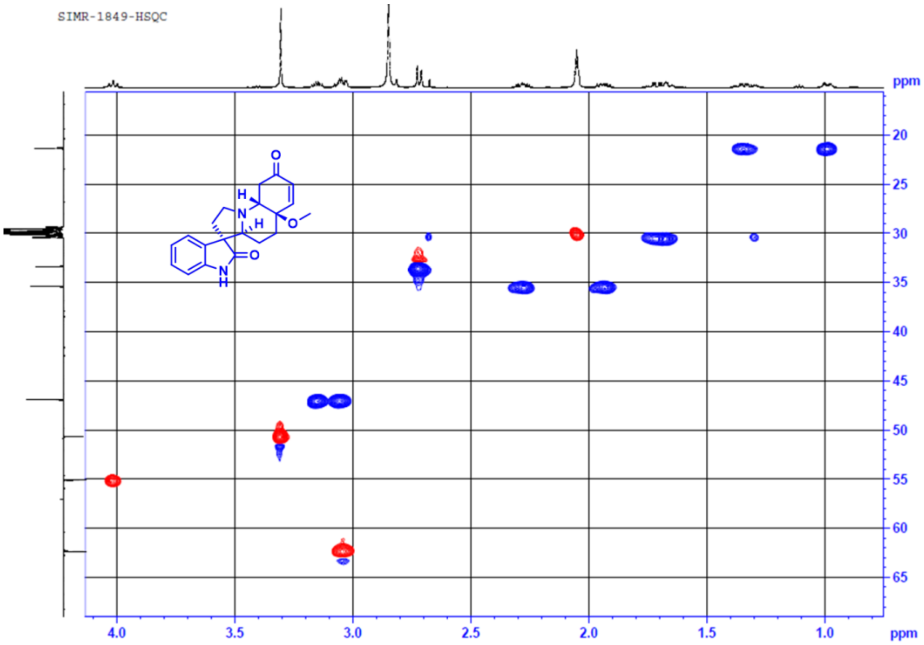

Supplementary Figure 128 HSQC (Expansion) spectrum of (3S,3a'S,5a'R,9a'R)-5a'-methoxy-1',2',3a',4',5',5a',9',9a'-octahydro-8'H-spiro[indoline-3,3'-pyrrolo[1,2-a]quinoline]-2,8'-dione (**18**)

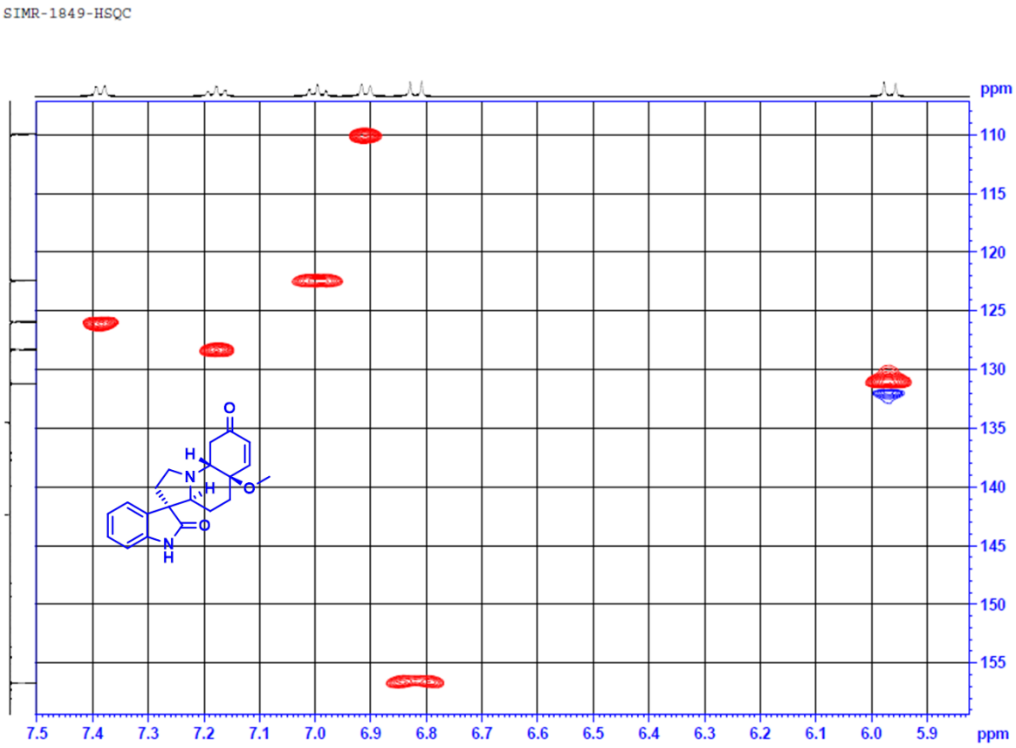

Supplementary Figure 129 HSQC (Expansion) spectrum of (3S,3a'S,5a'R,9a'R)-5a'-methoxy-1',2',3a',4',5',5a',9',9a'-octahydro-8'H-spiro[indoline-3,3'-pyrrolo[1,2-a]quinoline]-2,8'-dione (**18**)

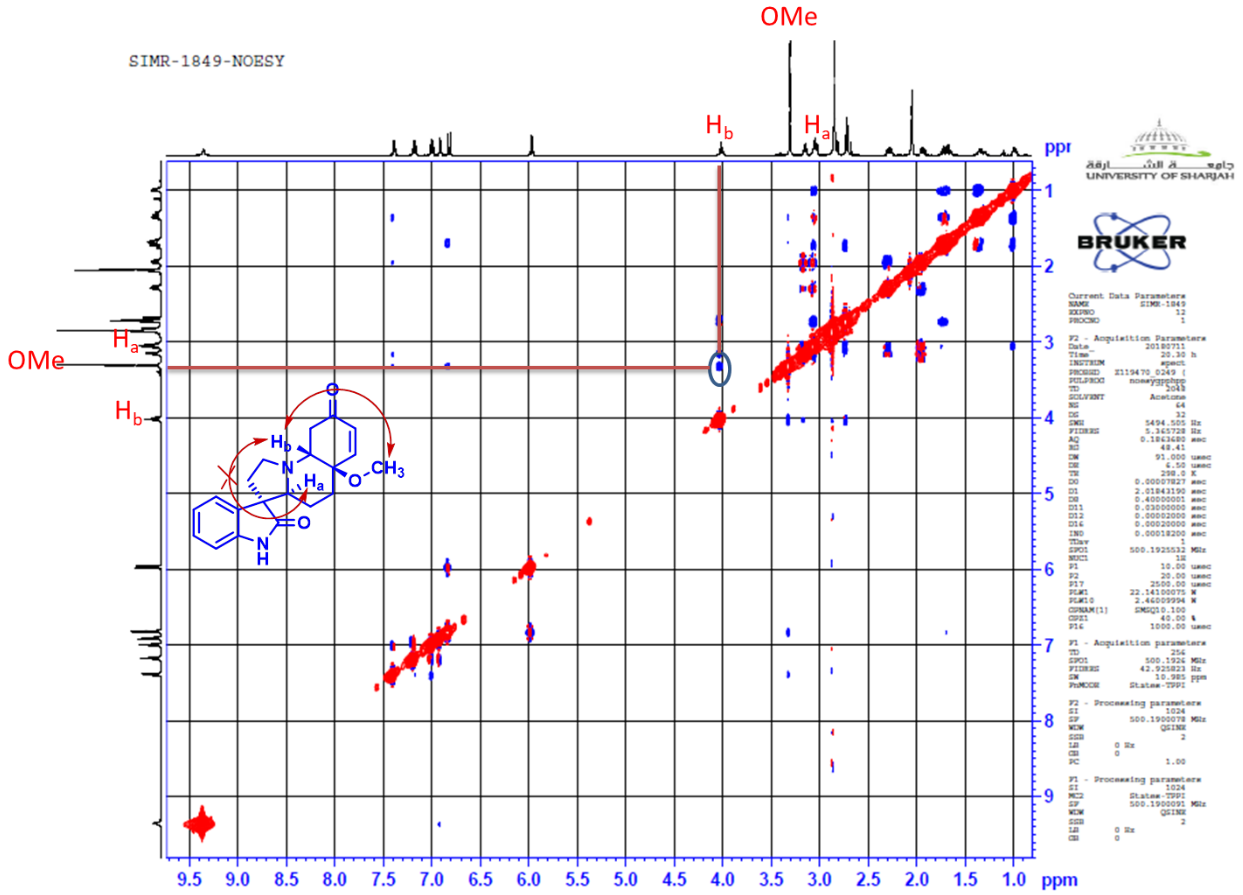

Supplementary Figure 130 NOESY (500 MHz, Acetone-d_6) spectrum of (3S,3a'S,5a'R,9a'R)-5a'-methoxy-1',2',3a',4',5',5a',9',9a'-octahydro-8'H-spiro[indoline-3,3'-pyrrolo[1,2-a]quinoline]-2,8'-dione (**18**)

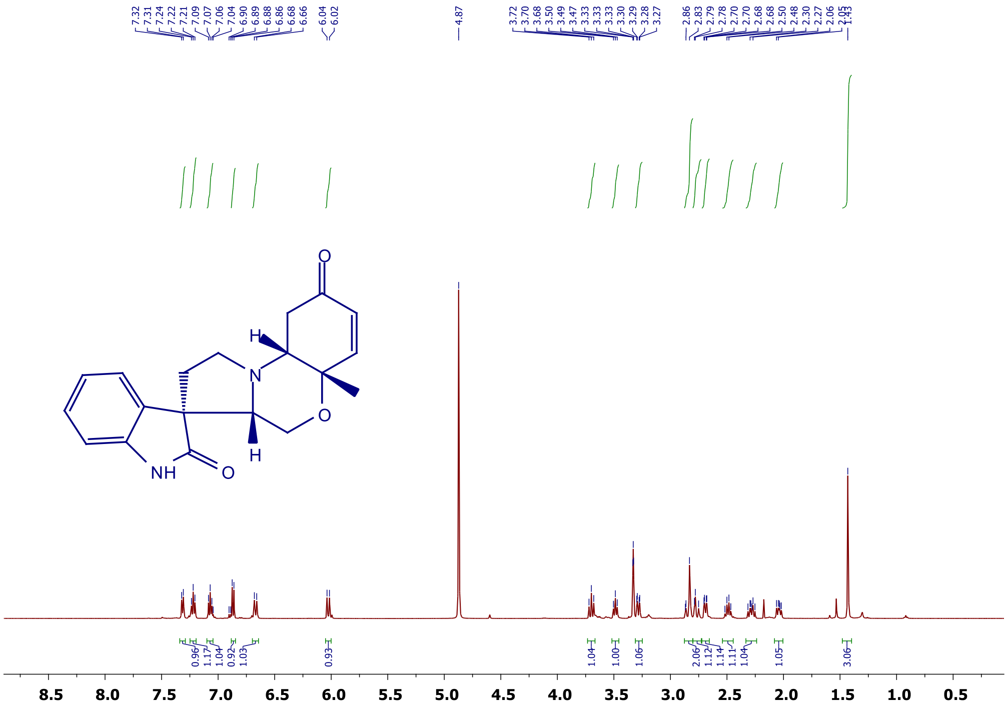

Supplementary Figure 131 ^{1}H NMR (500 MHz, CD_3OD) spectrum of (3S,3aS,5aS,9aR)-5a-methyl-1,2,3a,4,9,9a-hexahydrospiro[benzo[b]pyrrolo[1,2-d][1,4]oxazine-3,3'-indoline]-2',8(5aH)-dione (**19**)

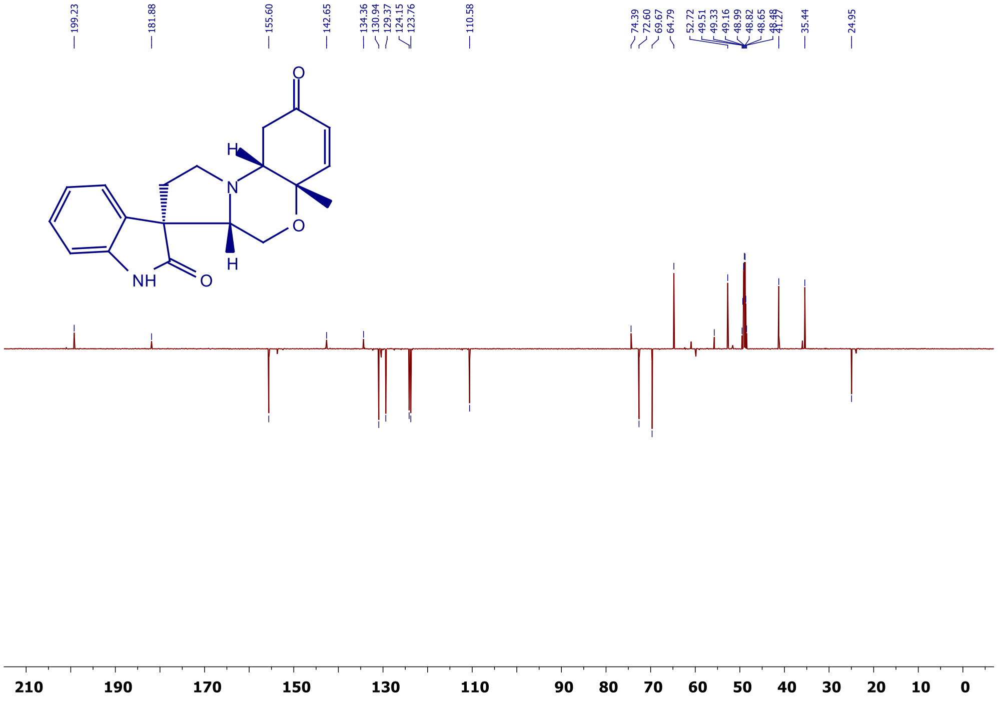

Supplementary Figure 132 APT NMR (125 MHz, CD_3OD) spectrum of (3S,3aS,5aS,9aR)-5a-methyl-1,2,3a,4,9,9a-hexahydrospiro[benzo[b]pyrrolo[1,2-d][1,4]oxazine-3,3'-indoline]-2',8(5aH)-dione (**19**)

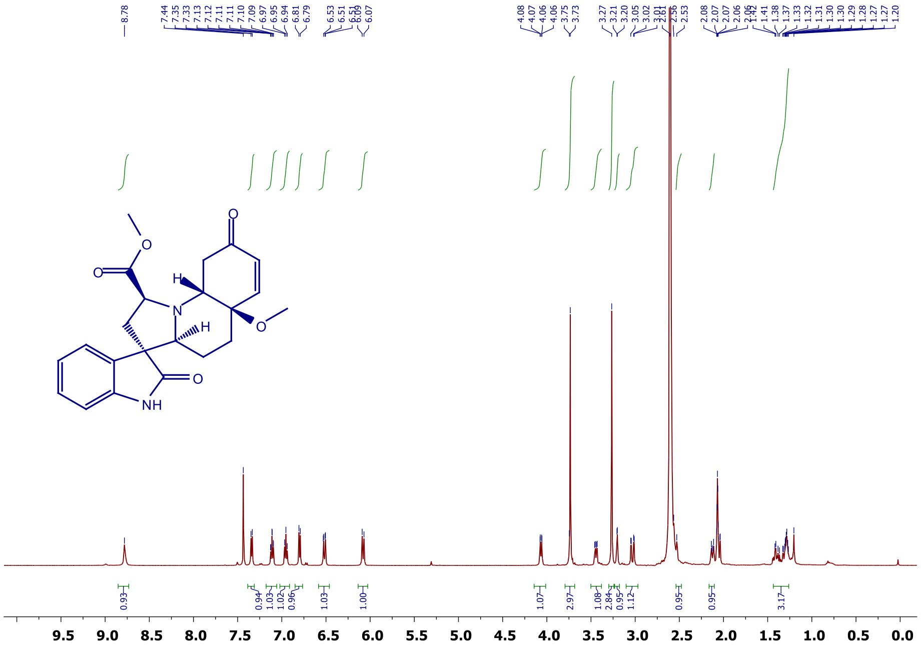

Supplementary Figure 133 ^{1}H NMR (500 MHz, $CDCl_3$+Acetone-d_6) spectrum of methyl (1'S,3S,3a'S,5a'R,9a'R)-5a'-methoxy-2,8'-dioxo-1',2',3a',4',5a',8',9',9a'-octahydro-5'H-spiro[indoline-3,3'-pyrrolo[1,2-a]quinoline]-1'-carboxylate (**20**)

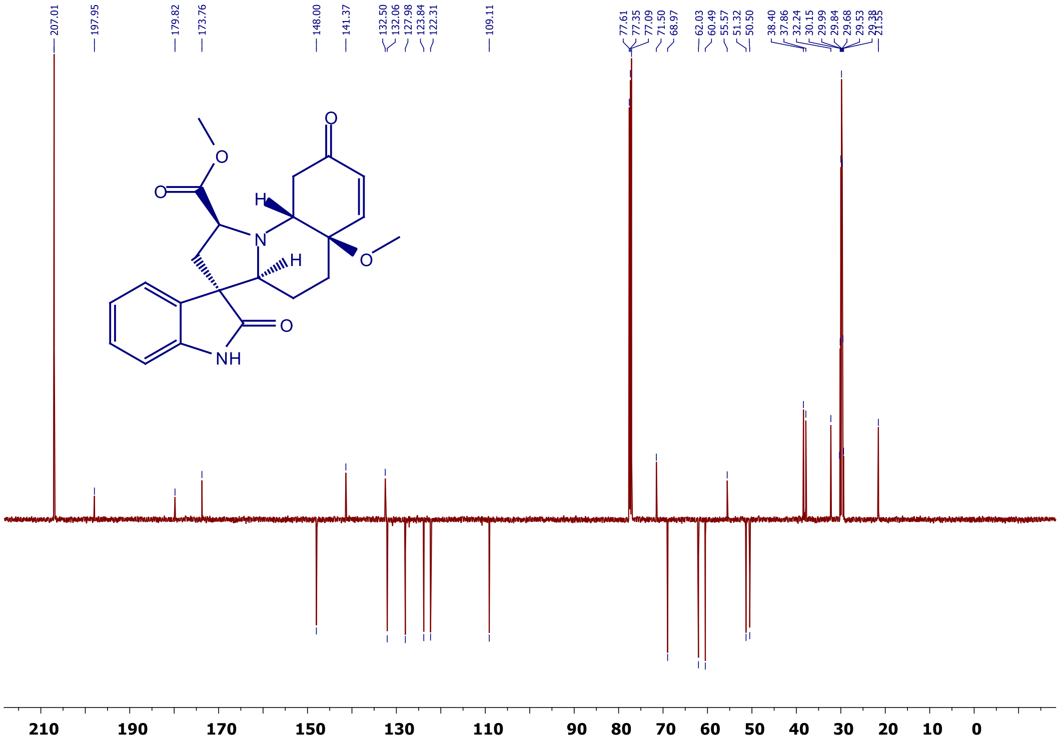

Supplementary Figure 134 APT NMR (125 MHz, $CDCl_3$+Acetone-d_6) spectrum of methyl (1'S,3S,3a'S,5a'R,9a'R)-5a'-methoxy-2,8'-dioxo-1',2',3a',4',5a',8',9',9a'-octahydro-5'H-spiro[indoline-3,3'-pyrrolo[1,2-a]quinoline]-1'-carboxylate (**20**)

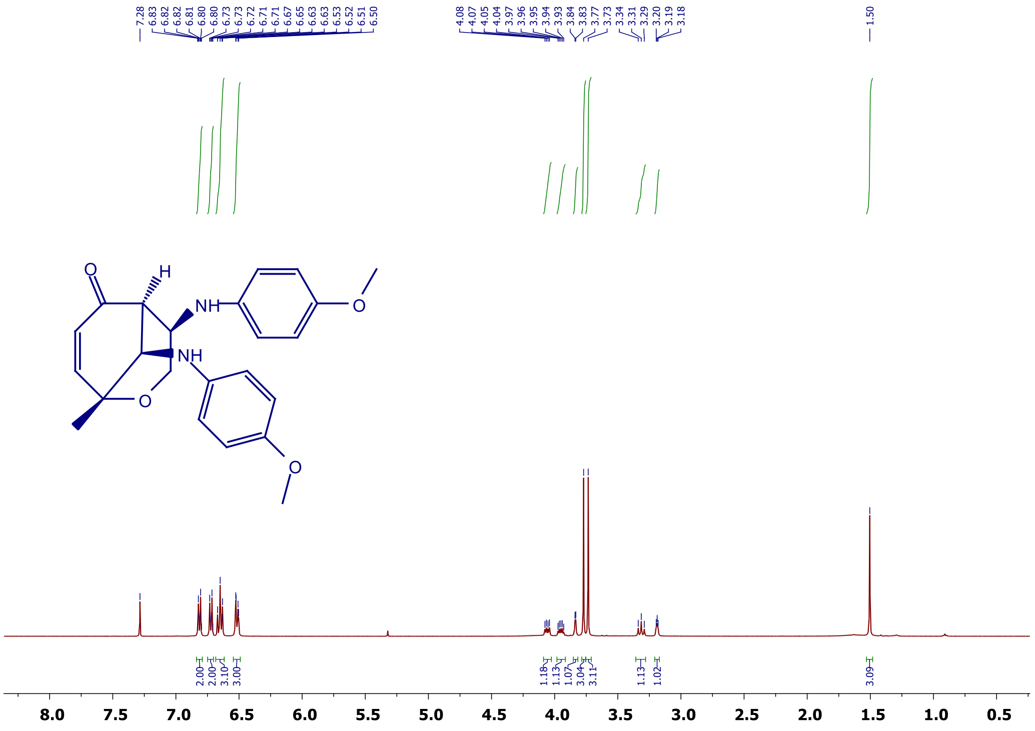

Supplementary Figure 135 ^{1}H NMR (500 MHz, $CDCl_3$) spectrum of (1R,4R,5S,9R)-4,9-bis((4-methoxyphenyl)amino)-1-methyl-2-oxabicyclo[3.3.1]non-7-en-6-one (**22a**)

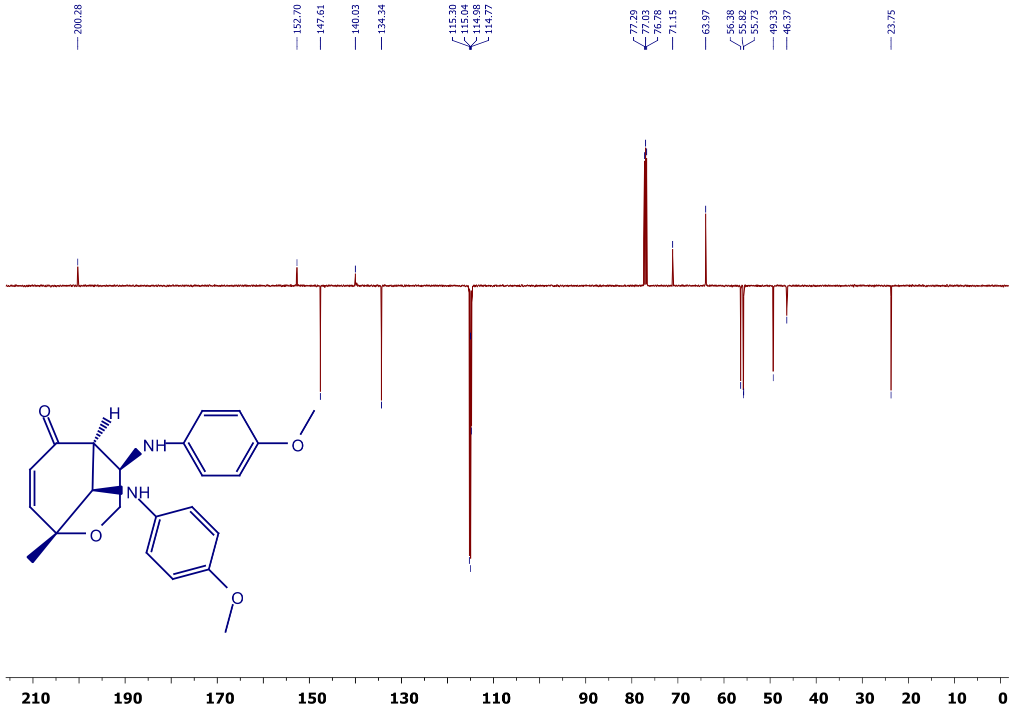

Supplementary Figure 136 APT NMR (125 MHz, $CDCl_3$) spectrum of (1R,4R,5S,9R)-4,9-bis((4-methoxyphenyl)amino)-1-methyl-2-oxabicyclo[3.3.1]non-7-en-6-one (**22a**)

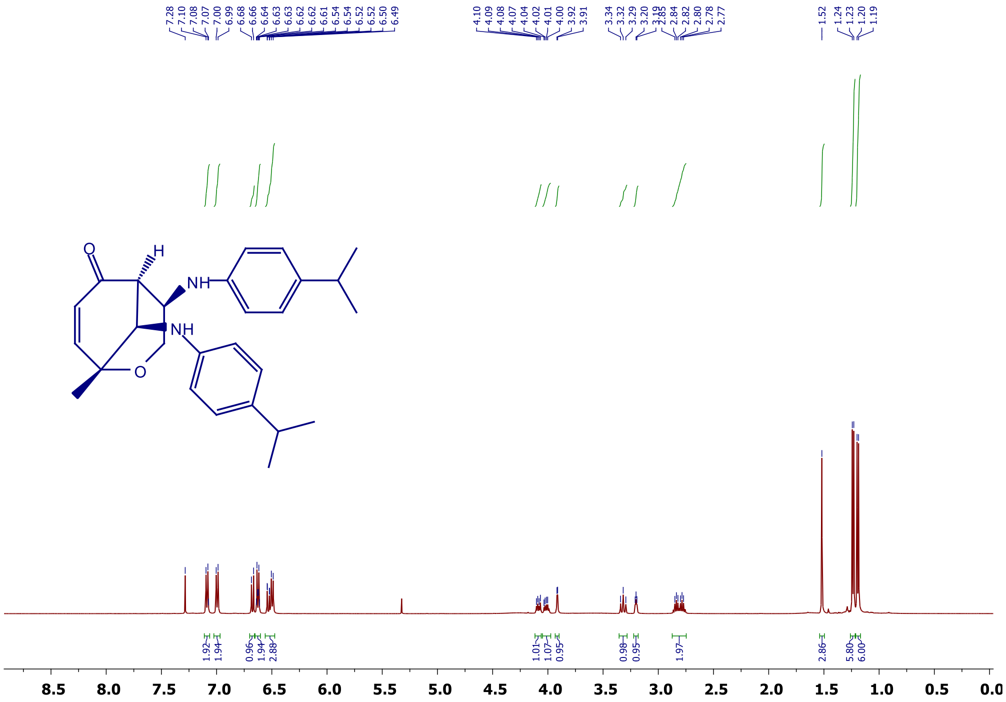

Supplementary Figure 137 ^{1}H NMR (500 MHz, $CDCl_3$) spectrum of (1R,4R,5S,9R)-4,9-bis((4-isopropylphenyl)amino)-1-methyl-2-oxabicyclo[3.3.1]non-7-en-6-one (**22b**)

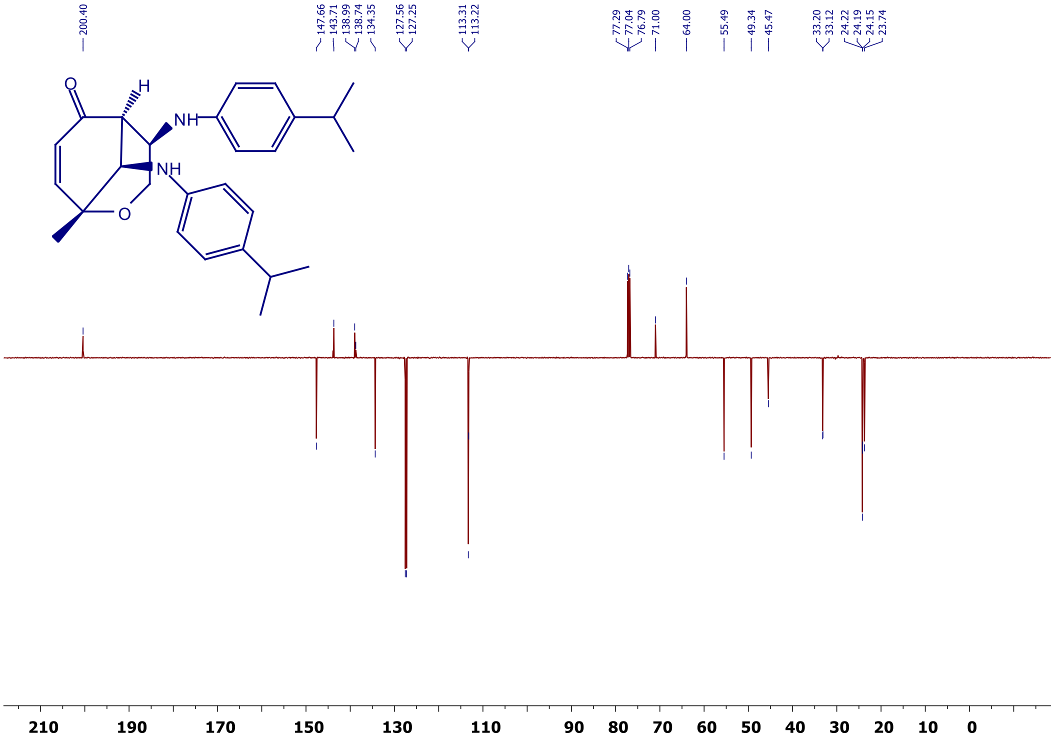

Supplementary Figure 138 APT NMR (125 MHz, $CDCl_3$) spectrum of (1R,4R,5S,9R)-4,9-bis((4-isopropylphenyl)amino)-1-methyl-2-oxabicyclo[3.3.1]non-7-en-6-one (**22b**)

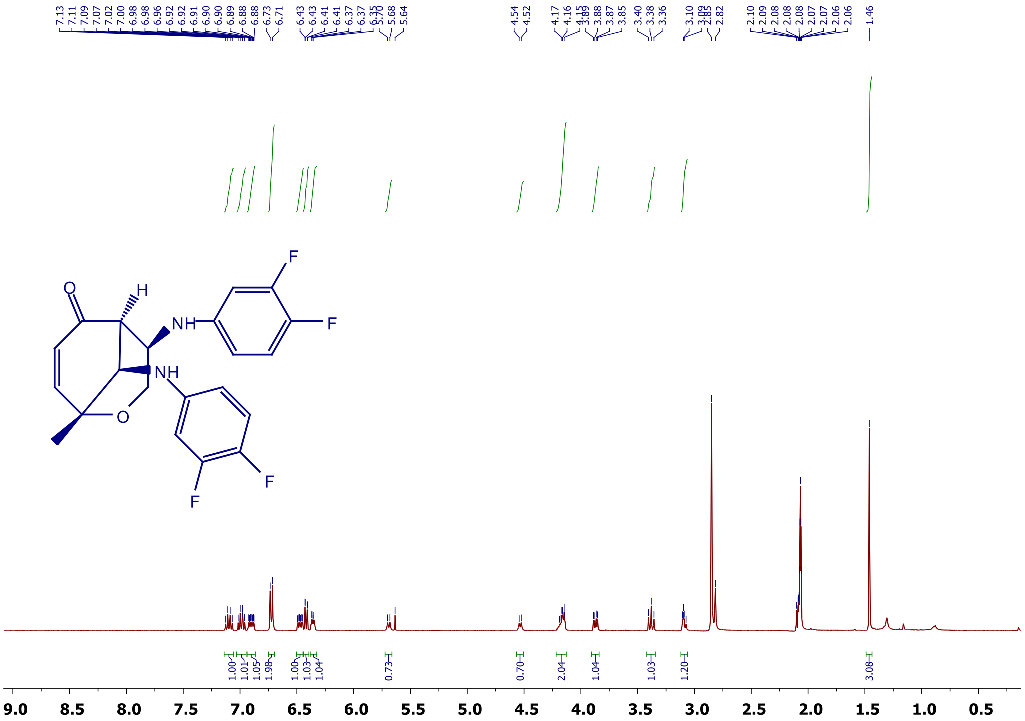

Supplementary Figure 139 ^{1}H NMR (500 MHz, Acetone-d_6) spectrum of (1R,4R,5S,9R)-4,9-bis((3,4-difluorophenyl)amino)-1-methyl-2-oxabicyclo[3.3.1]non-7-en-6-one (**22c**)

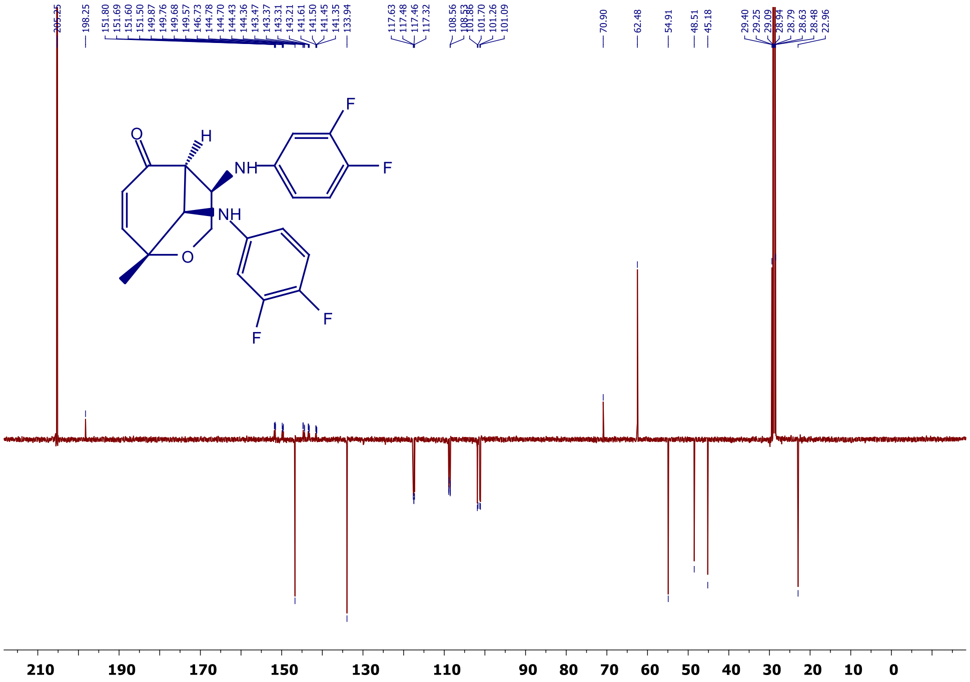

Supplementary Figure 140 APT NMR (125 MHz, Acetone-d_6) spectrum of (1R,4R,5S,9R)-4,9-bis((3,4-difluorophenyl)amino)-1-methyl-2-oxabicyclo[3.3.1]non-7-en-6-one (**22c**)

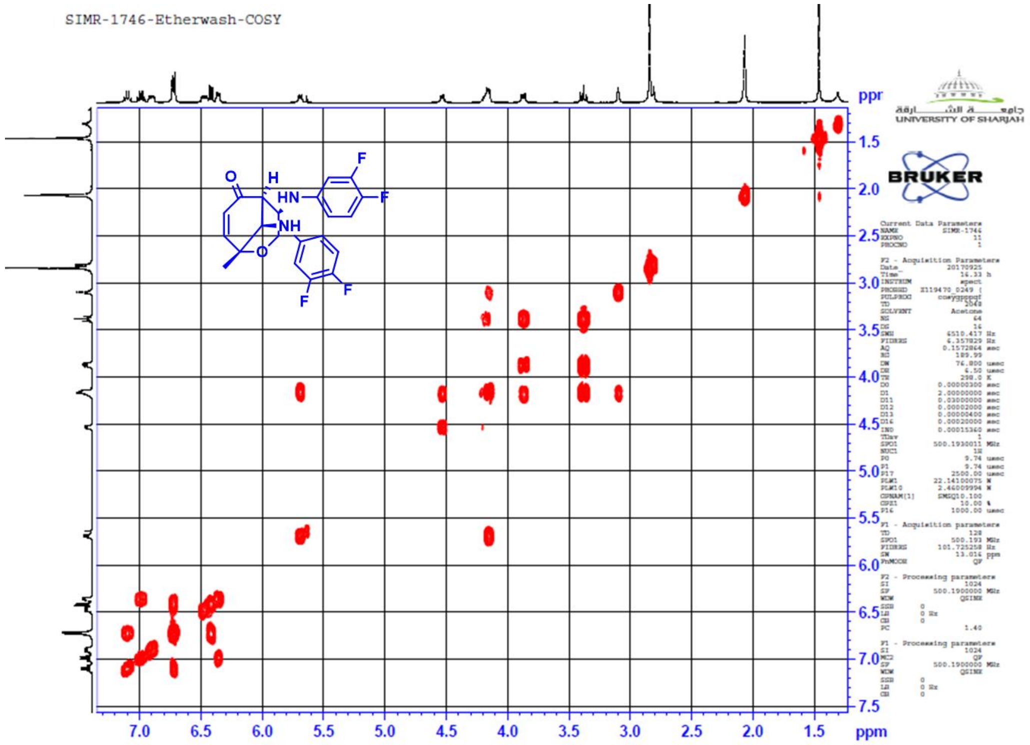

Supplementary Figure 141 COSY (500 MHz, Acetone-d_6) spectrum of (1R,4R,5S,9R)-4,9-bis((3,4-difluorophenyl)amino)-1-methyl-2-oxabicyclo[3.3.1]non-7-en-6-one (**22c**)

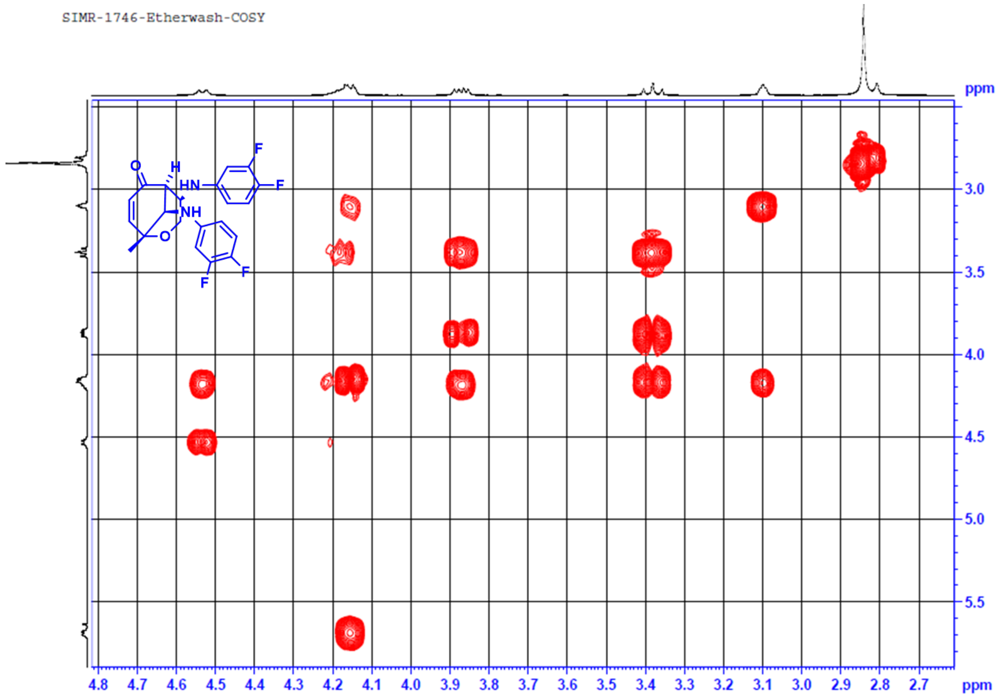

Supplementary Figure 142 COSY (Expansion) spectrum of (1R,4R,5S,9R)-4,9-bis((3,4-difluorophenyl)amino)-1-methyl-2-oxabicyclo[3.3.1]non-7-en-6-one (**22c**)

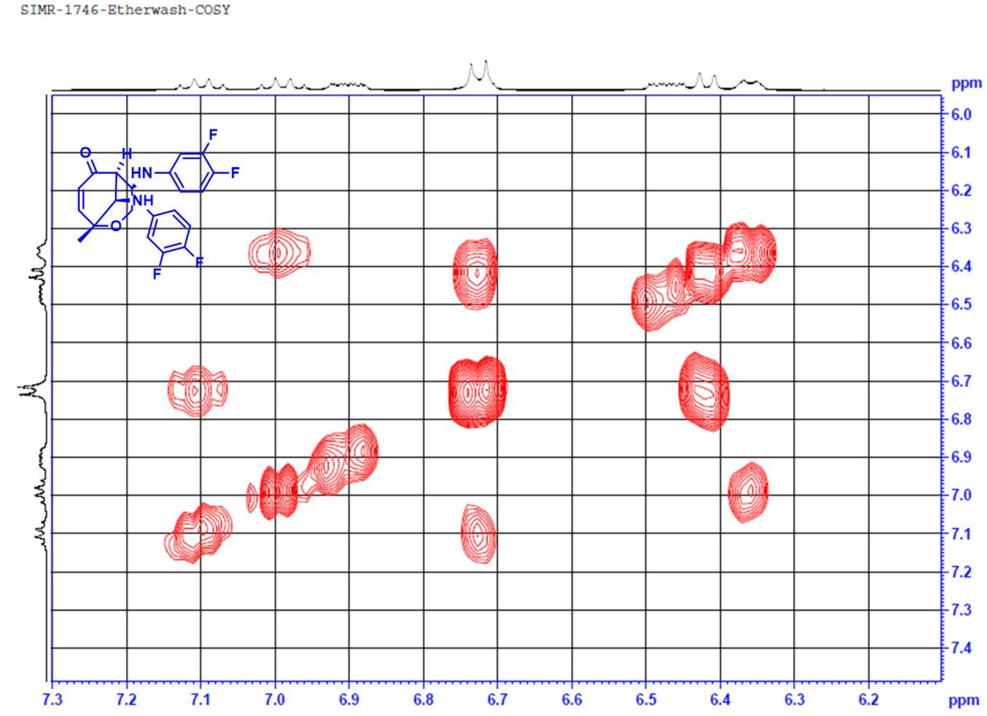

Supplementary Figure 143 COSY (Expansion) spectrum of (1R,4R,5S,9R)-4,9-bis((3,4-difluorophenyl)amino)-1-methyl-2-oxabicyclo[3.3.1]non-7-en-6-one (**22c**)

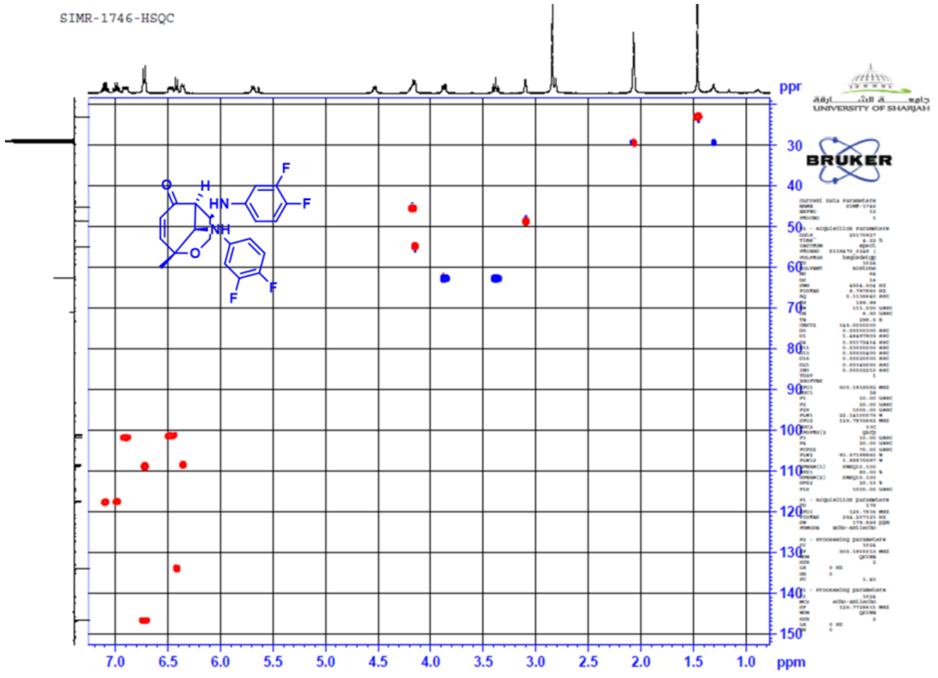

Supplementary Figure 144 HSQC (500 MHz, Acetone) spectrum of (1R,4R,5S,9R)-4,9-bis((3,4-difluorophenyl)amino)-1-methyl-2-oxabicyclo[3.3.1]non-7-en-6-one (**22c**)

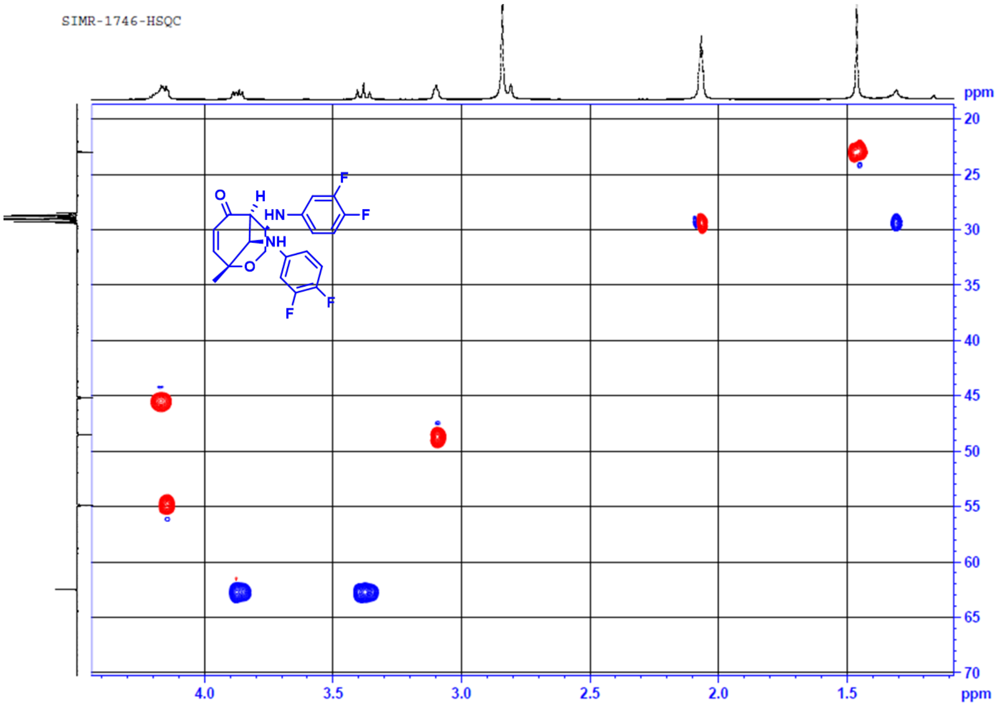

Supplementary Figure 145 HSQC (Expansion) spectrum of (1R,4R,5S,9R)-4,9-bis((3,4-difluorophenyl)amino)-1-methyl-2-oxabicyclo[3.3.1]non-7-en-6-one (**22c**)

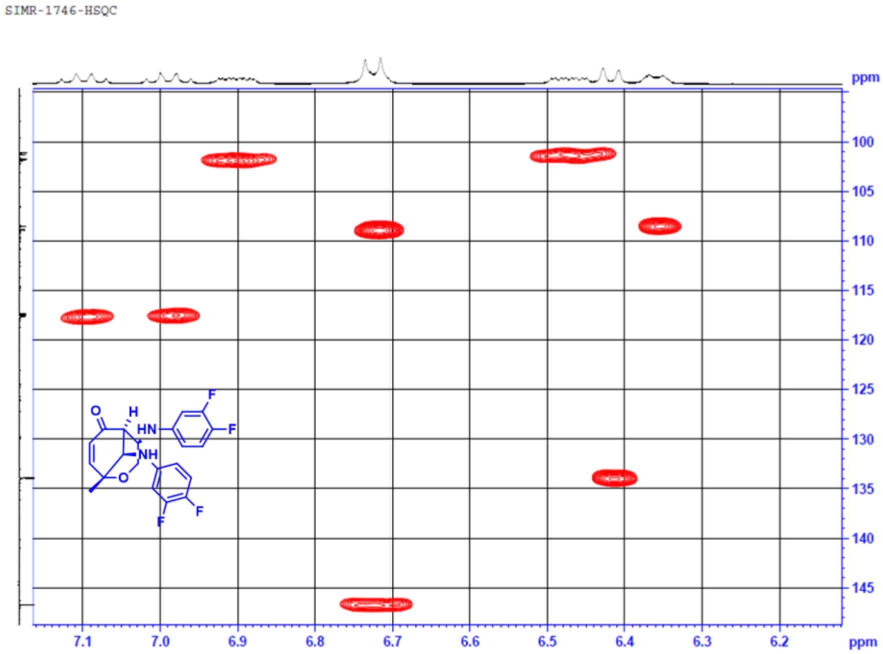

Supplementary Figure 146 HSQC (Expansion) spectrum of (1R,4R,5S,9R)-4,9-bis((3,4-difluorophenyl)amino)-1-methyl-2-oxabicyclo[3.3.1]non-7-en-6-one (**22c**)

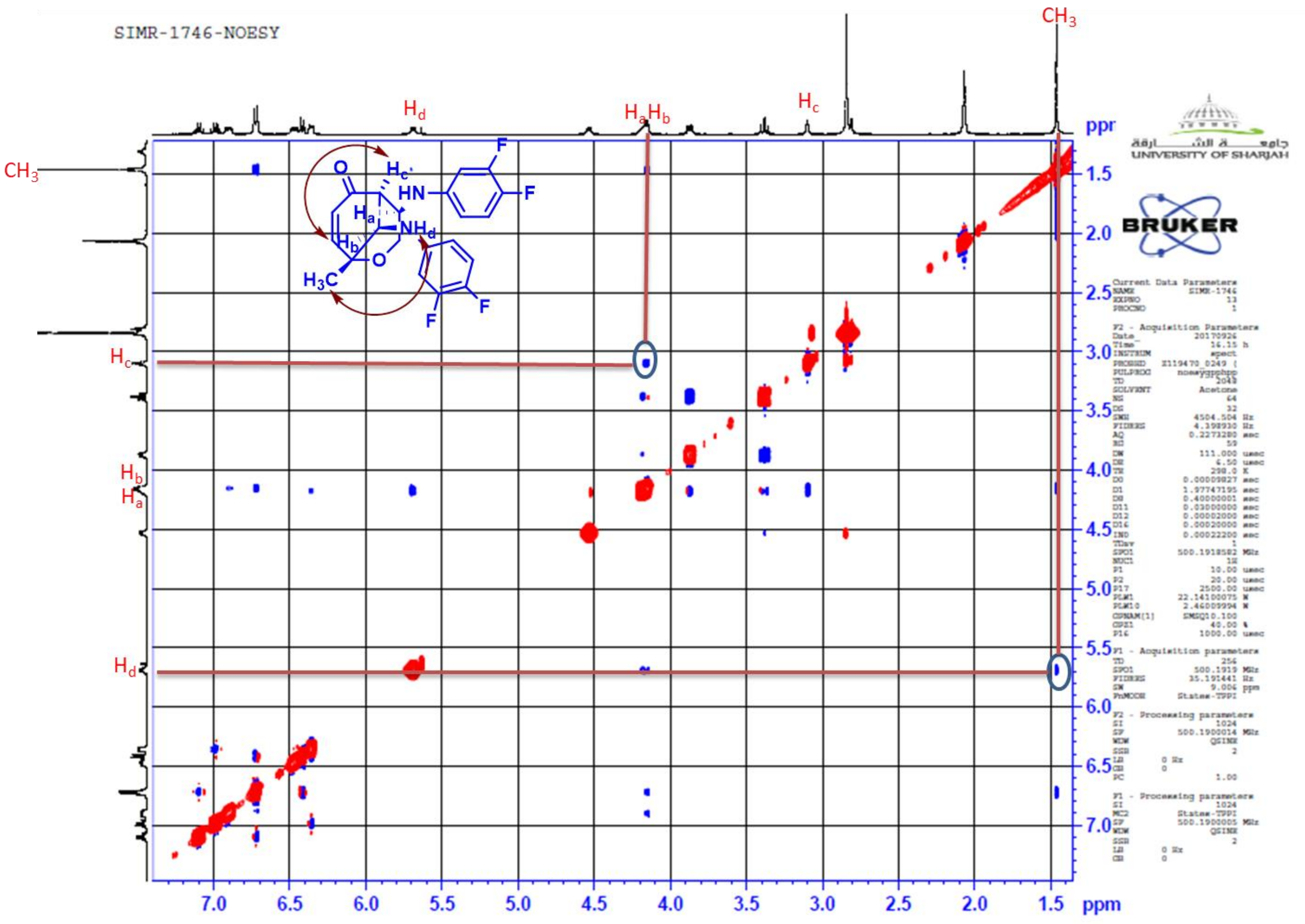

Supplementary Figure 147 NOESY (500 MHz, Acetone-d$_6$) spectrum of (1R,4R,5S,9R)-4,9-bis((3,4-difluorophenyl)amino)-1-methyl-2-oxabicyclo[3.3.1]non-7-en-6-one (**22c**)

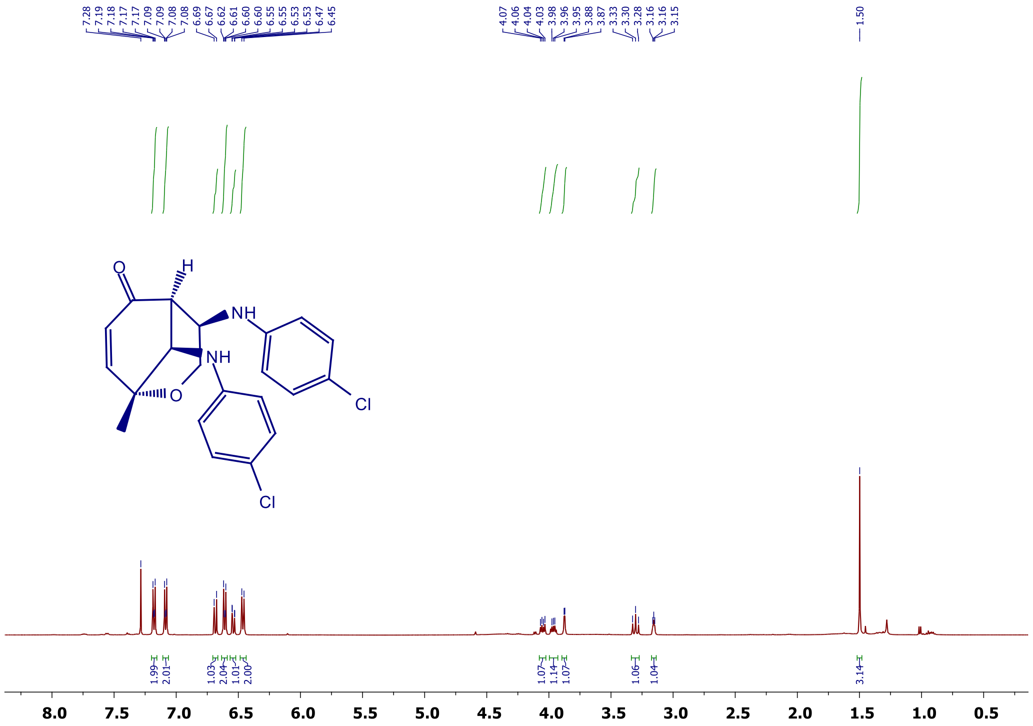

Supplementary Figure 148 ^{1}H (500 MHz, $CDCl_3$) spectrum of (1R,4R,5S,9R)-4,9-bis((4-chlorophenyl)amino)-1-methyl-2-oxabicyclo[3.3.1]non-7-en-6-one (**22d**)

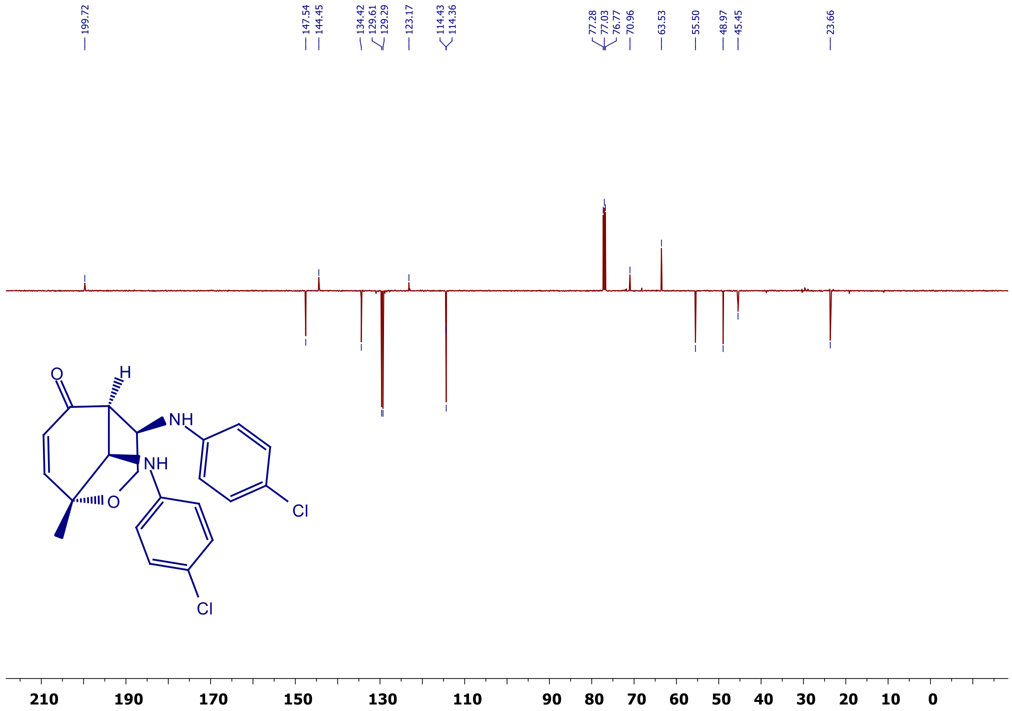

Supplementary Figure 149 APT NMR (125 MHz, $CDCl_3$) spectrum of (1R,4R,5S,9R)-4,9-bis((4-chlorophenyl)amino)-1-methyl-2-oxabicyclo[3.3.1]non-7-en-6-one (**22d**)

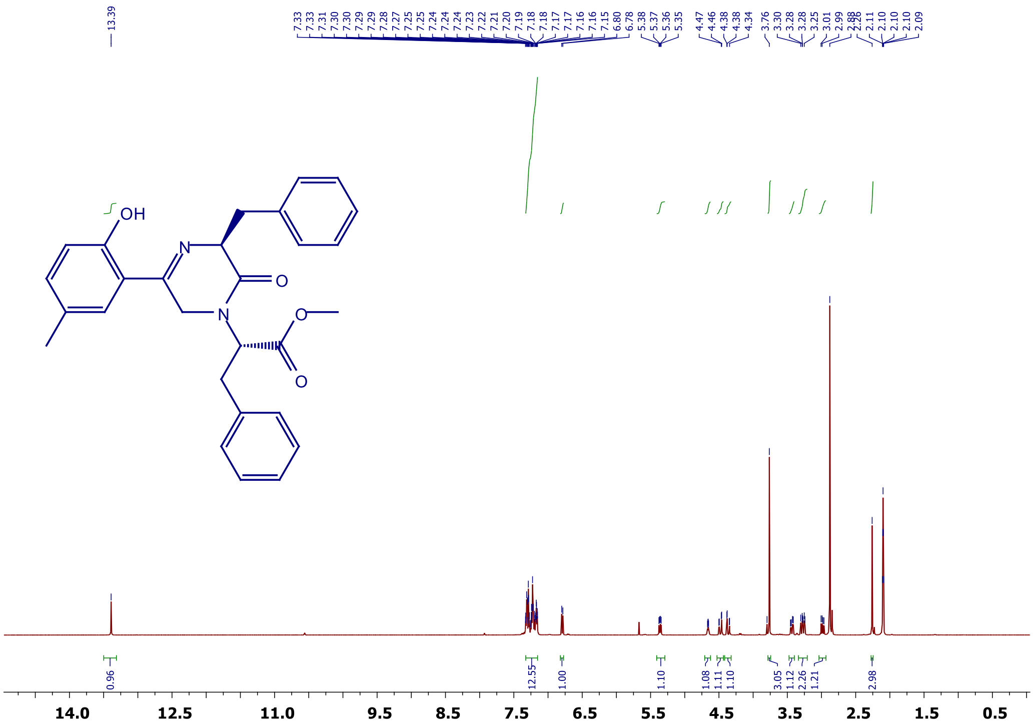

Supplementary Figure 150 ^{1}H (500 MHz, Acetone-d_6) spectrum of methyl (S)-2-((S)-3-benzyl-5-(2-hydroxy-5-methylphenyl)-2-oxo-3,6-dihydropyrazin-1(2H)-yl)-3-phenylpropanoate (**24a**)

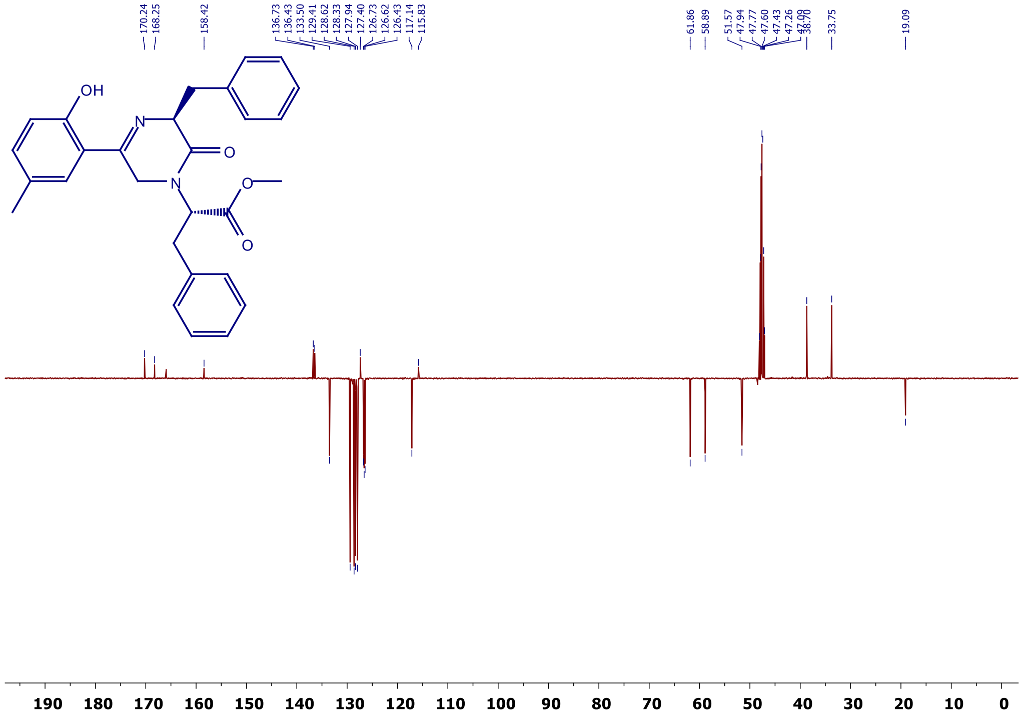

Supplementary Figure 151 APT NMR (125 MHz, CD_3OD) spectrum of methyl (S)-2-((S)-3-benzyl-5-(2-hydroxy-5-methylphenyl)-2-oxo-3,6-dihydropyrazin-1(2H)-yl)-3-phenylpropanoate (**24a**)

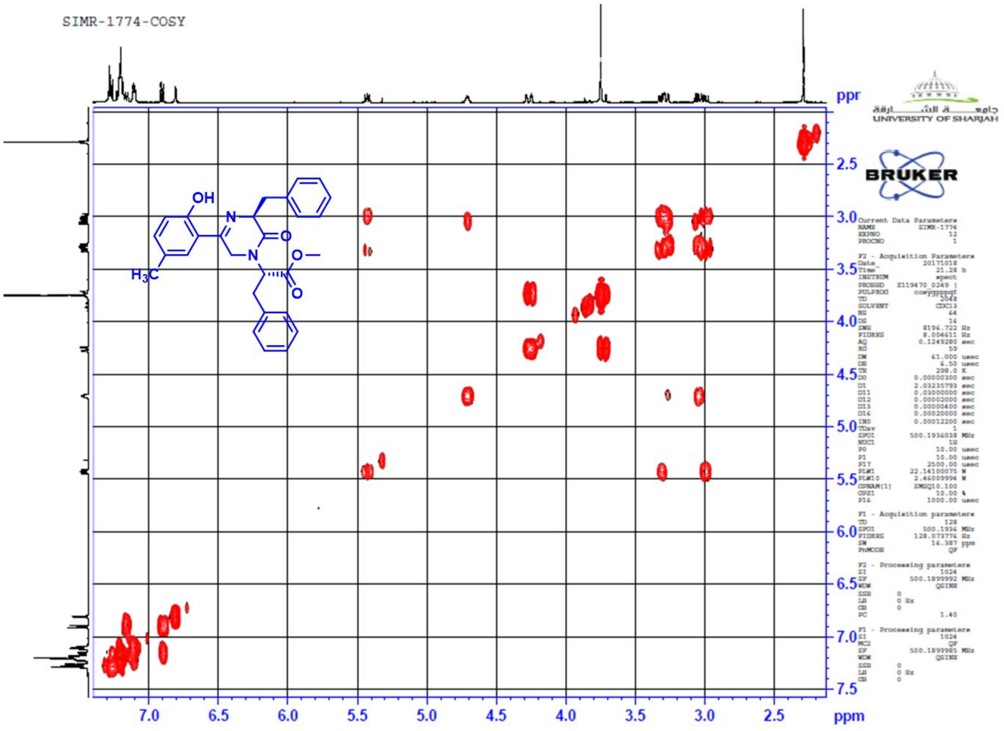

Supplementary Figure 152 COSY (500 MHz, $CDCl_3$) spectrum of methyl (S)-2-((S)-3-benzyl-5-(2-hydroxy-5-methylphenyl)-2-oxo-3,6-dihydropyrazin-1(2H)-yl)-3-phenylpropanoate (**24a**)

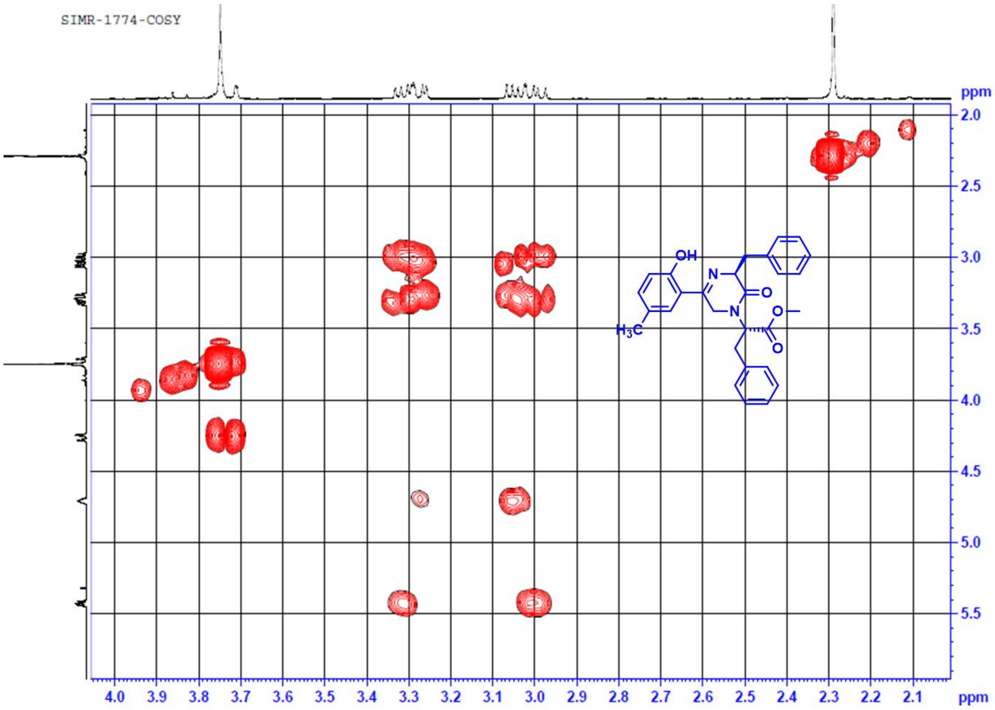

Supplementary Figure 153 COSY (Expansion) spectrum of methyl (S)-2-((S)-3-benzyl-5-(2-hydroxy-5-methylphenyl)-2-oxo-3,6-dihydropyrazin-1(2H)-yl)-3-phenylpropanoate (**24a**)

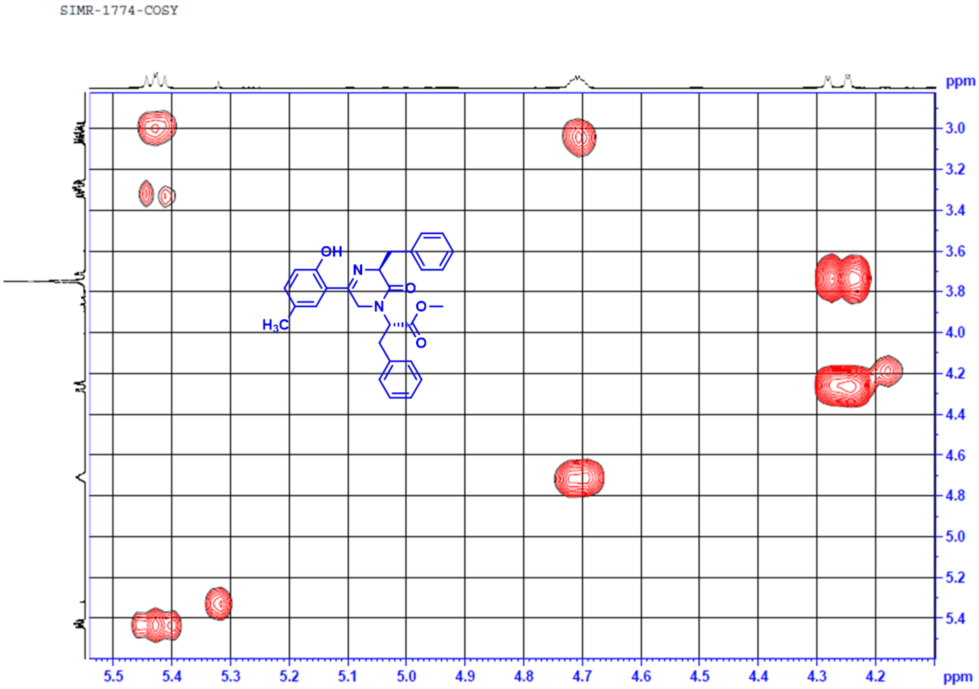

Supplementary Figure 154 COSY (Expansion) spectrum of methyl (S)-2-((S)-3-benzyl-5-(2-hydroxy-5-methylphenyl)-2-oxo-3,6-dihydropyrazin-1(2H)-yl)-3-phenylpropanoate (**24a**)

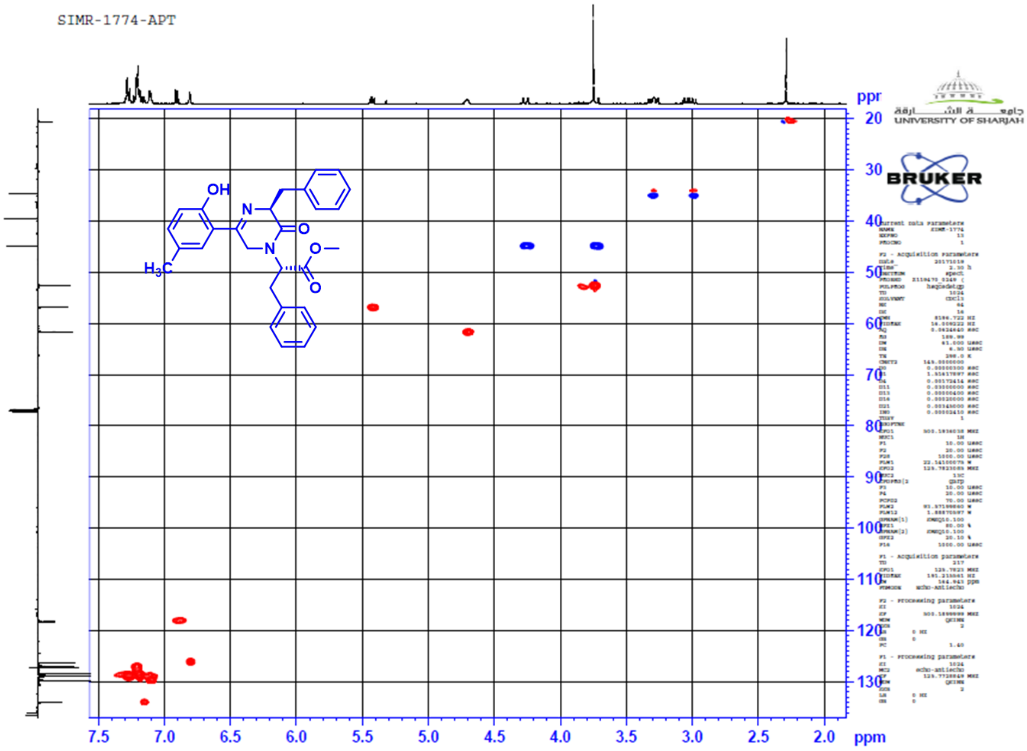

Supplementary Figure 155 HSQC (500 MHz, $CDCl_3$) spectrum of methyl (S)-2-((S)-3-benzyl-5-(2-hydroxy-5-methylphenyl)-2-oxo-3,6-dihydropyrazin-1(2H)-yl)-3-phenylpropanoate (**24a**)

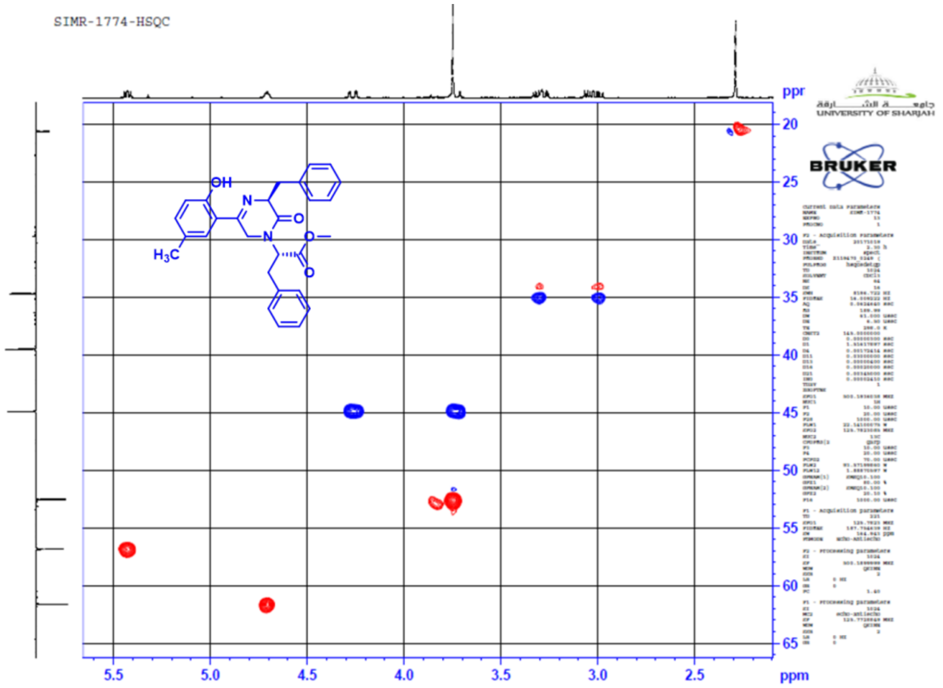

Supplementary Figure 156 HSQC (Expansion) spectrum of methyl (S)-2-((S)-3-benzyl-5-(2-hydroxy-5-methylphenyl)-2-oxo-3,6-dihydropyrazin-1(2H)-yl)-3-phenylpropanoate (**24a**)

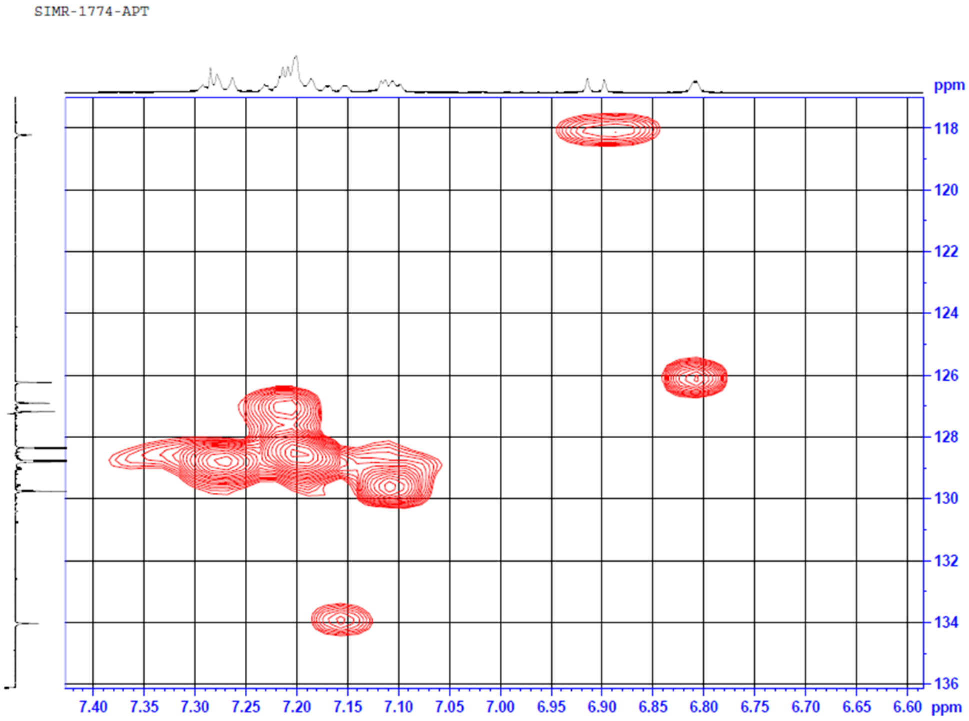

Supplementary Figure 157 HSQC (Expansion) spectrum of methyl (S)-2-((S)-3-benzyl-5-(2-hydroxy-5-methylphenyl)-2-oxo-3,6-dihydropyrazin-1(2H)-yl)-3-phenylpropanoate (**24a**)

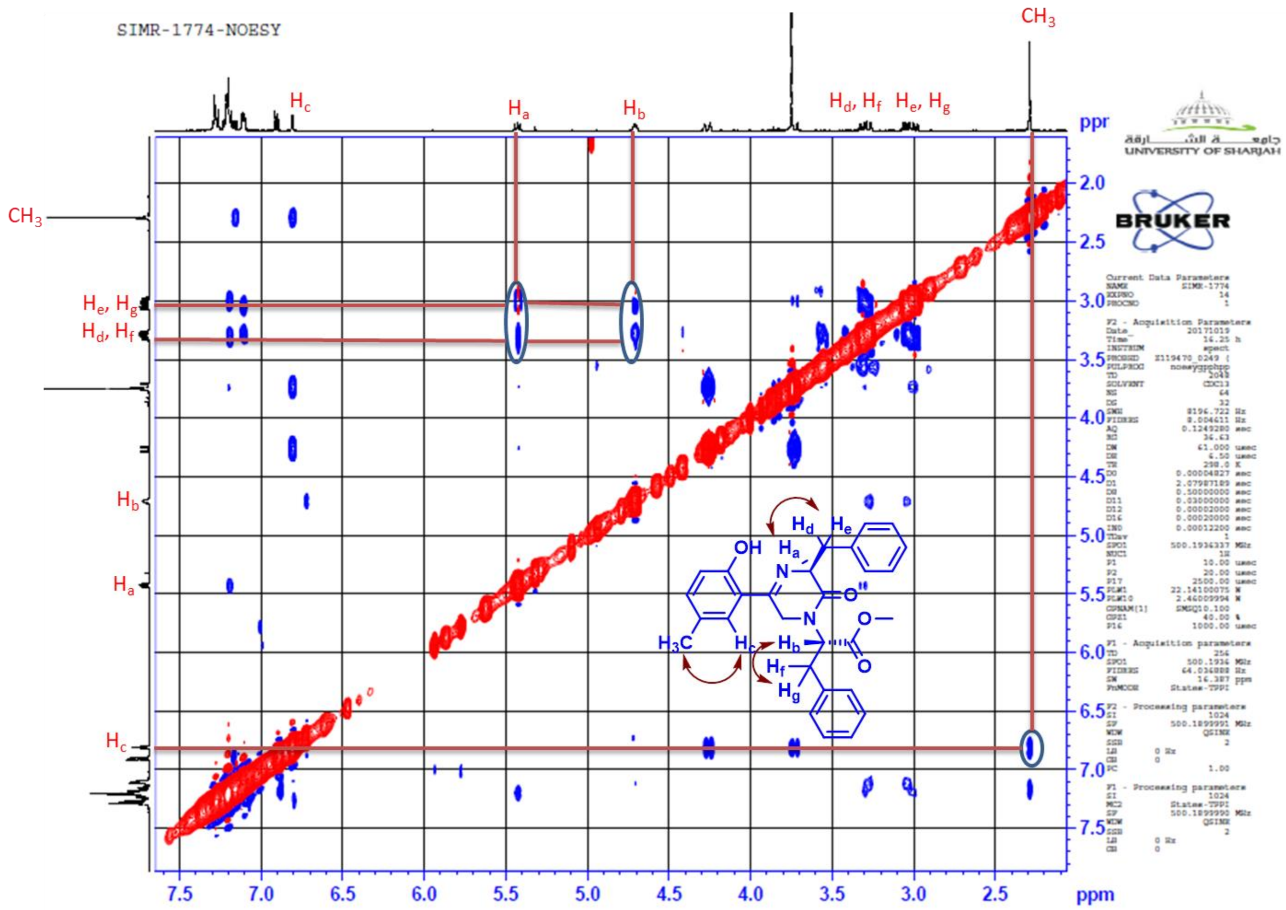

Supplementary Figure 158 NOESY (500 MHz, $CDCl_3$) spectrum of methyl (S)-2-((S)-3-benzyl-5-(2-hydroxy-5-methylphenyl)-2-oxo-3,6-dihydropyrazin-1(2H)-yl)-3-phenylpropanoate (**24a**)

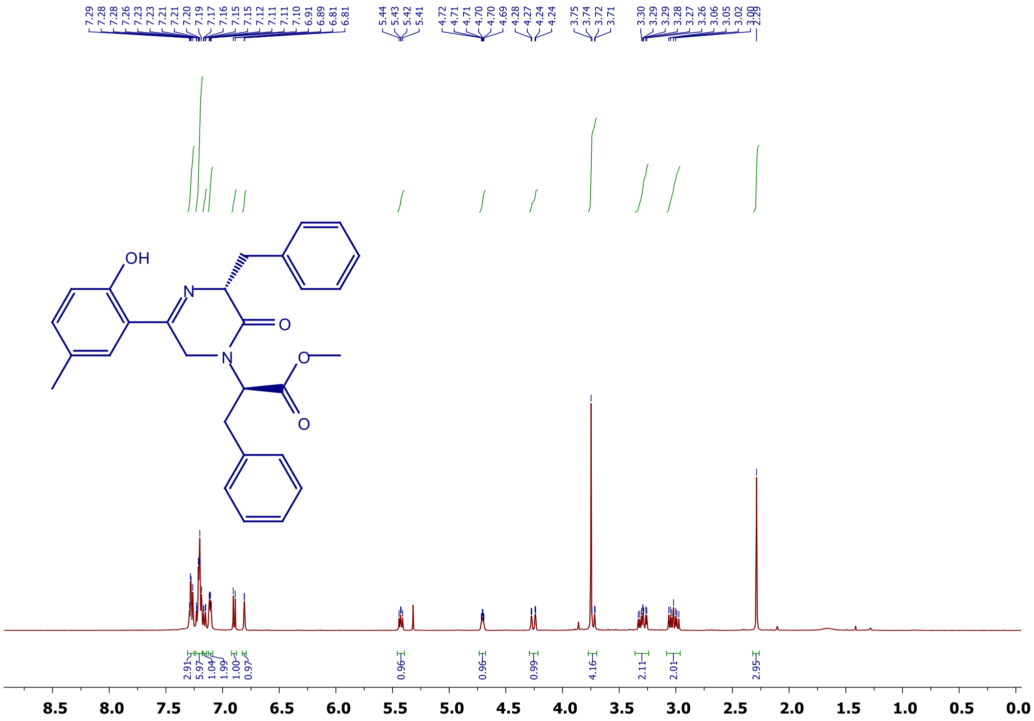

Supplementary Figure 159 ^{1}H (500 MHz, $CDCl_3$) spectrum of methyl (R)-2-((R)-3-benzyl-5-(2-hydroxy-5-methylphenyl)-2-oxo-3,6-dihydropyrazin-1(2H)-yl)-3-phenylpropanoate (**24b**)

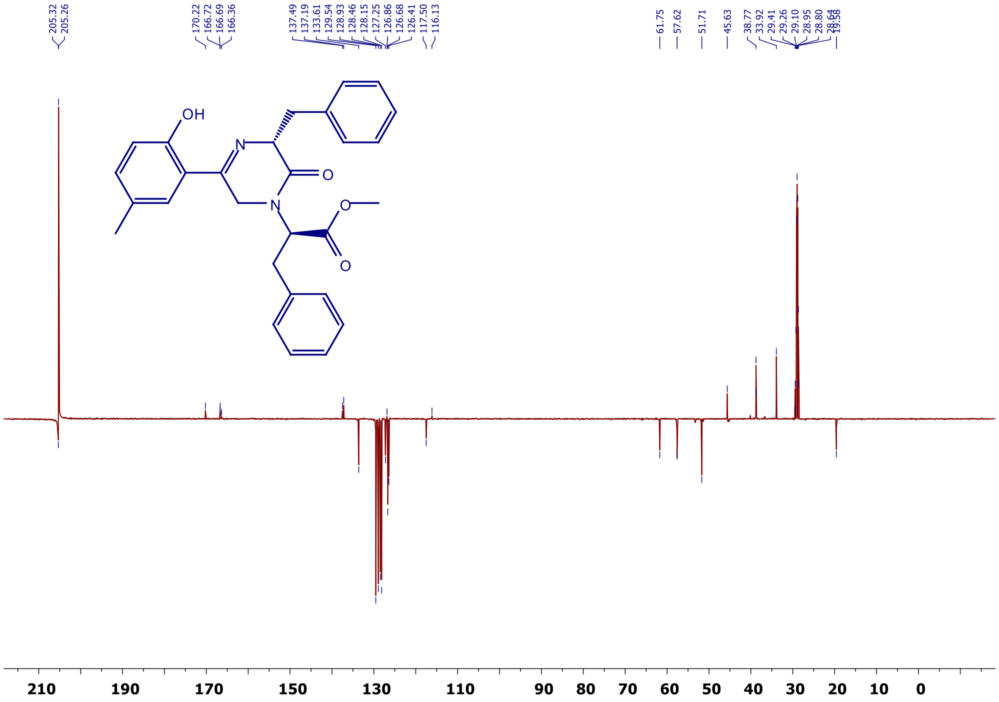

Supplementary Figure 160 APT NMR (125 MHz, Acetone-d_6) spectrum of methyl (R)-2-((R)-3-benzyl-5-(2-hydroxy-5-methylphenyl)-2-oxo-3,6-dihydropyrazin-1(2H)-yl)-3-phenylpropanoate (**24b**)

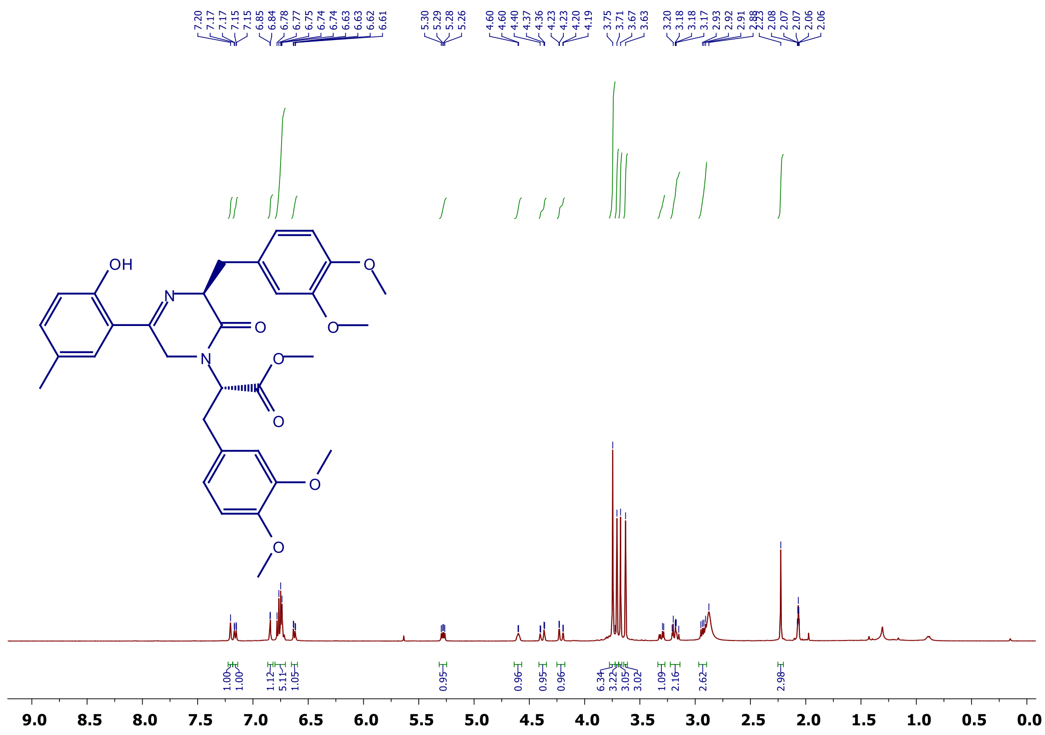

Supplementary Figure 161 ^{1}H (500 MHz, Acetone-d_6) spectrum methyl (S)-2-((S)-3-(3,4-dimethoxybenzyl)-5-(2-hydroxy-5-methylphenyl)-2-oxo-3,6-dihydropyrazin-1(2H)-yl)-3-(3,4-dimethoxyphenyl)propanoate (**24c**)

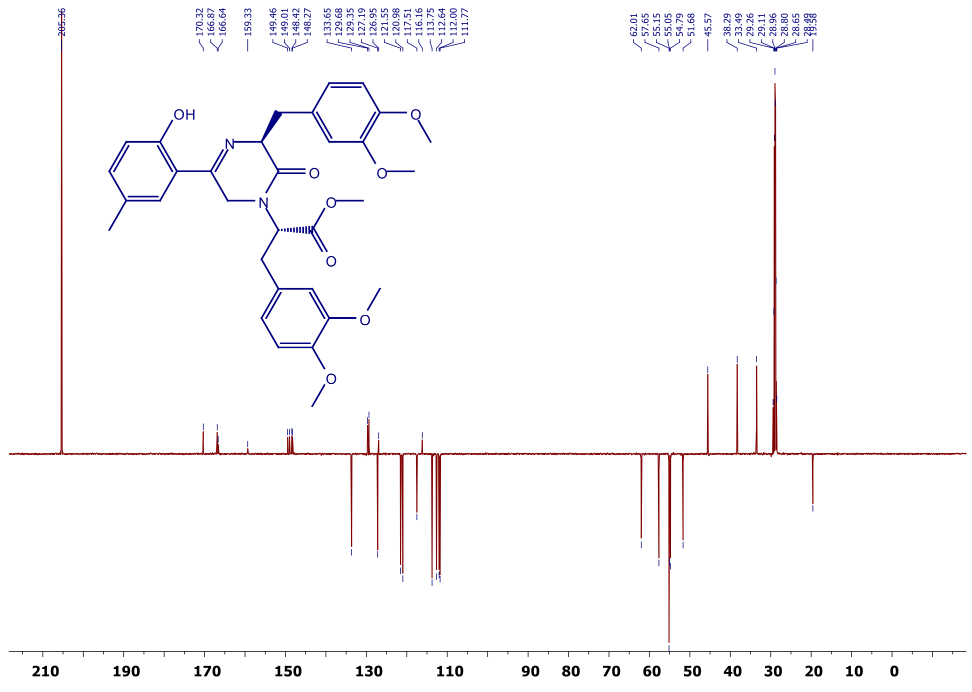

Supplementary Figure 162 APT NMR (125 MHz, Acetone-d_6) spectrum methyl (S)-2-((S)-3-(3,4-dimethoxybenzyl)-5-(2-hydroxy-5-methylphenyl)-2-oxo-3,6-dihydropyrazin-1(2H)-yl)-3-(3,4-dimethoxyphenyl)propanoate (**24c**)

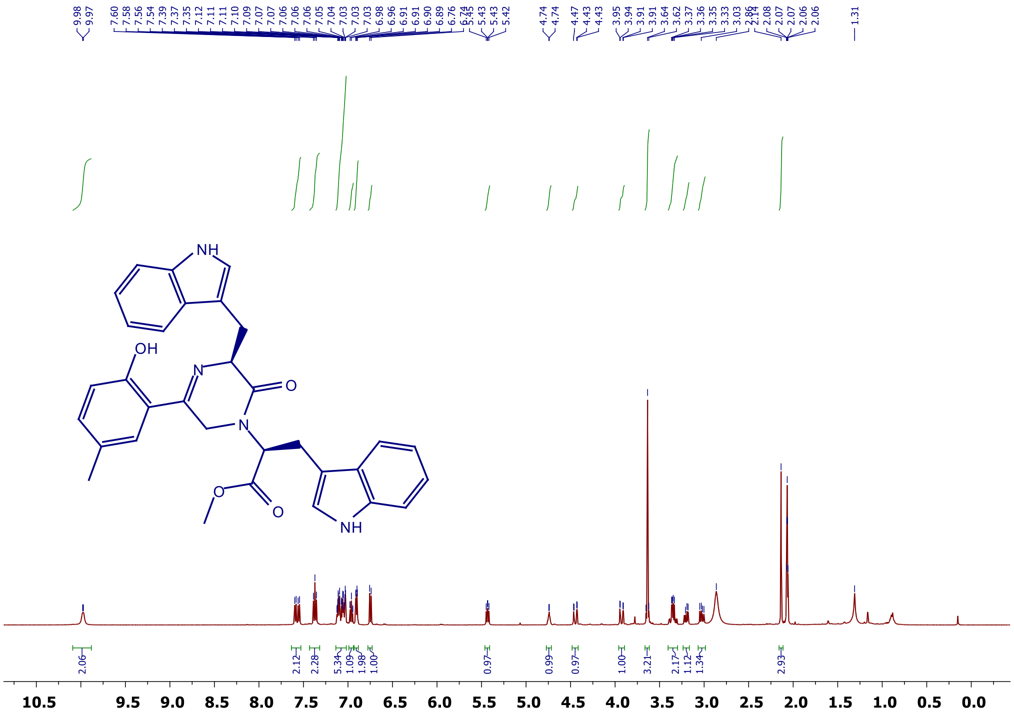

Supplementary Figure 163 ^{1}H (500 MHz, Acetone-d$_6$) spectrum methyl (S)-2-((S)-3-((1H-indol-3-yl)methyl)-5-(2-hydroxy-5-methylphenyl)-2-oxo-3,6-dihydropyrazin-1(2H)-yl)-3-(1H-indol-3-yl)propanoate (**24d**)

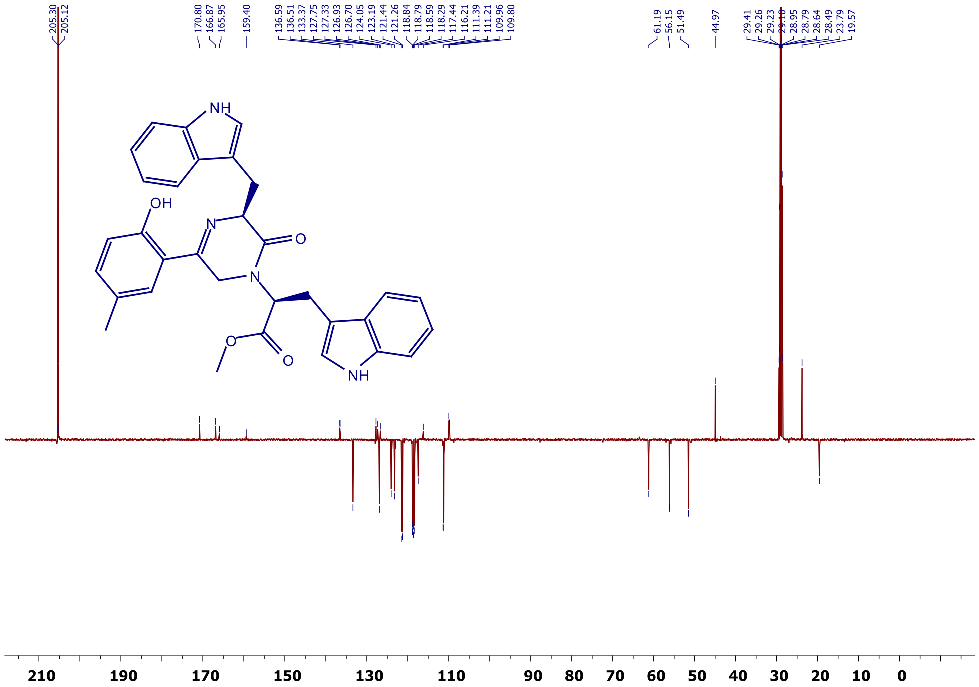

Supplementary Figure 164 APT NMR (125 MHz, Acetone-d$_6$) spectrum methyl (S)-2-((S)-3-((1H-indol-3-yl)methyl)-5-(2-hydroxy-5-methylphenyl)-2-oxo-3,6-dihydropyrazin-1(2H)-yl)-3-(1H-indol-3-yl)propanoate (**24d**)

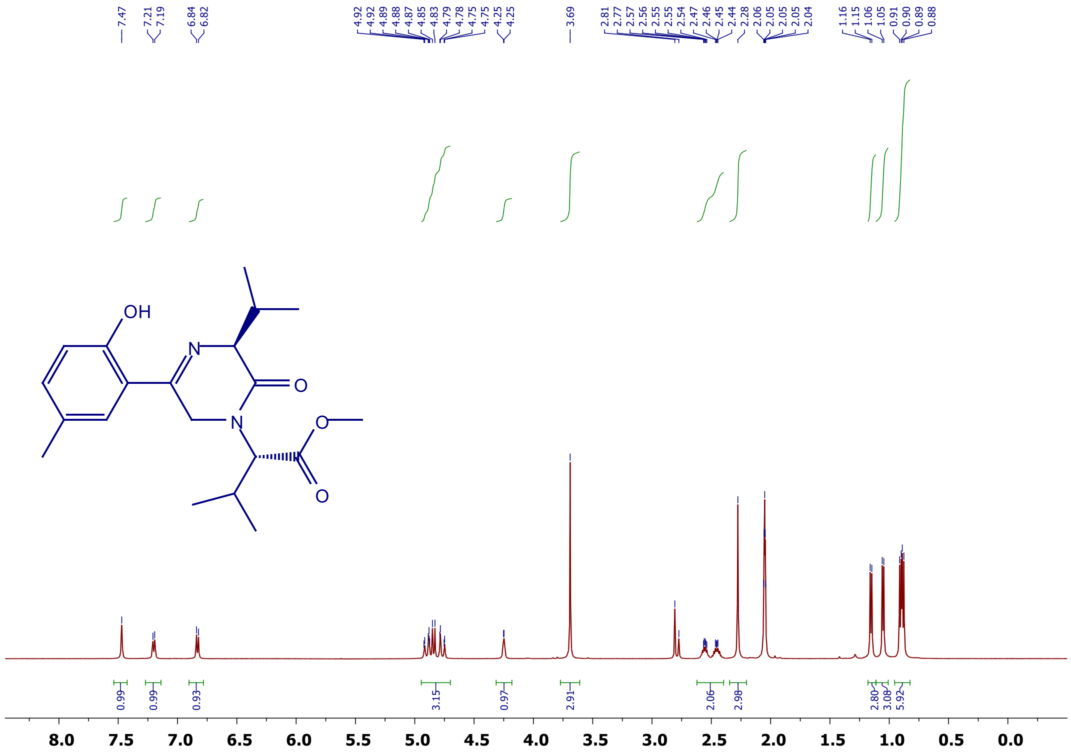

Supplementary Figure 165 ^{1}H (500 MHz, Acetone-d_6) spectrum methyl (S)-2-((S)-5-(2-hydroxy-5-methylphenyl)-3-isopropyl-2-oxo-3,6-dihydropyrazin-1(2H)-yl)-3-methylbutanoate (**24e**)

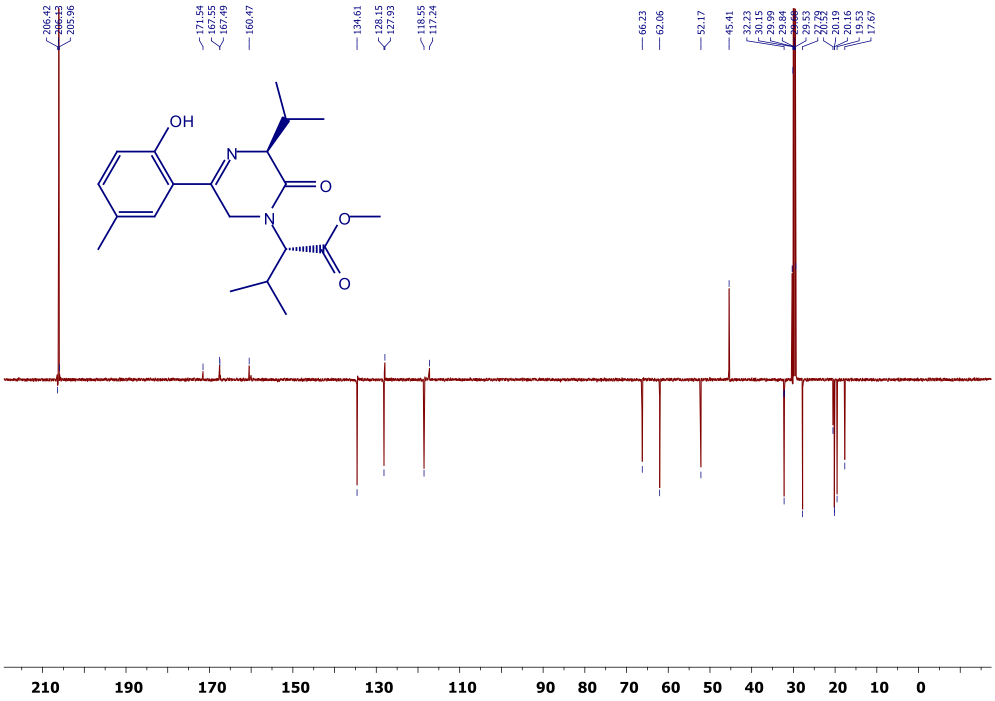

Supplementary Figure 166 APT NMR (125 MHz, Acetone-d_6) spectrum methyl (S)-2-((S)-5-(2-hydroxy-5-methylphenyl)-3-isopropyl-2-oxo-3,6-dihydropyrazin-1(2H)-yl)-3-methylbutanoate (**24e**)

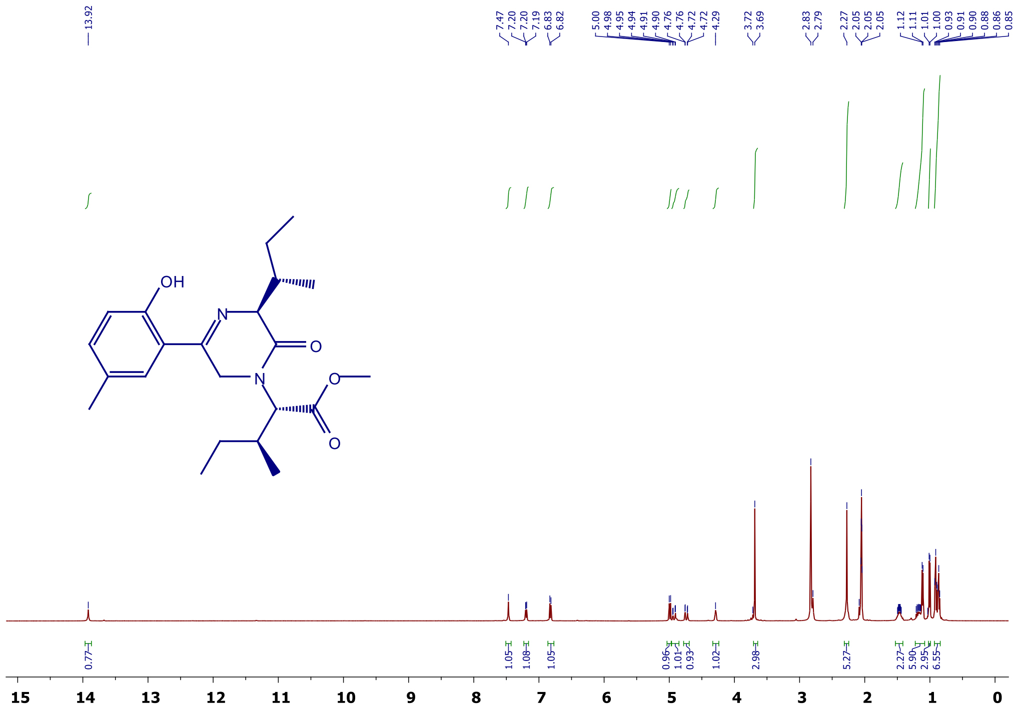

Supplementary Figure 167 ^{1}H (500 MHz, $CDCl_3$) spectrum methyl (2S,3S)-2-((S)-3-((S)-sec-butyl)-5-(2-hydroxy-5-methylphenyl)-2-oxo-3,6-dihydropyrazin-1(2H)-yl)-3-methylpentanoate (**24f**)

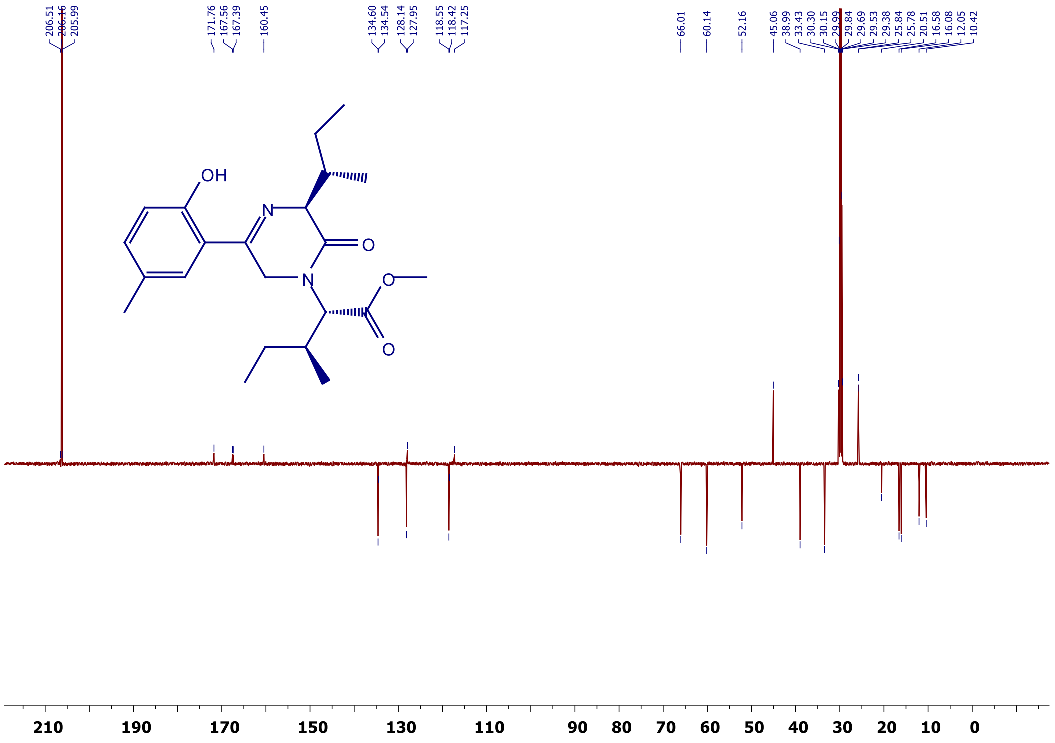

Supplementary Figure 168 APT NMR (125 MHz, $CDCl_3$) spectrum methyl (2S,3S)-2-((S)-3-((S)-sec-butyl)-5-(2-hydroxy-5-methylphenyl)-2-oxo-3,6-dihydropyrazin-1(2H)-yl)-3-methylpentanoate (**24f**)

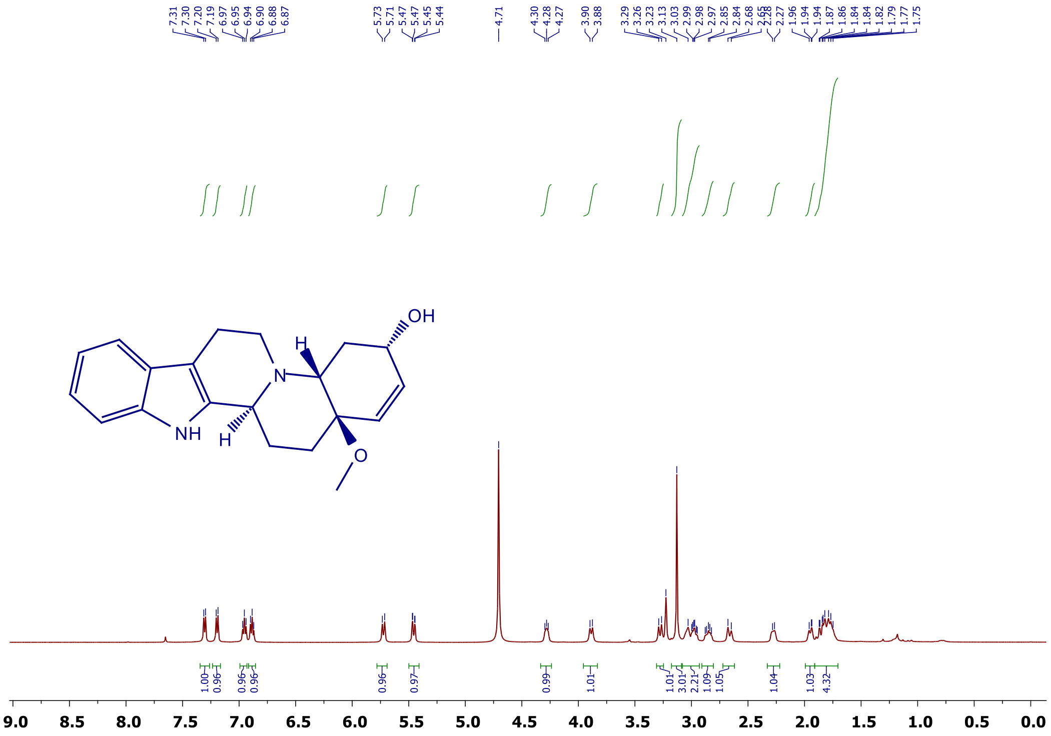

Supplementary Figure 169 ^{1}H (500 MHz, CD_3OD) spectrum of (2aR,5S,6aR,14bS)-2a-methoxy-1,2,2a,5,6,6a,8,9,14,14b-decahydroindolo[2',3':3,4]pyrido[1,2-a]quinolin-5-ol (**25**)

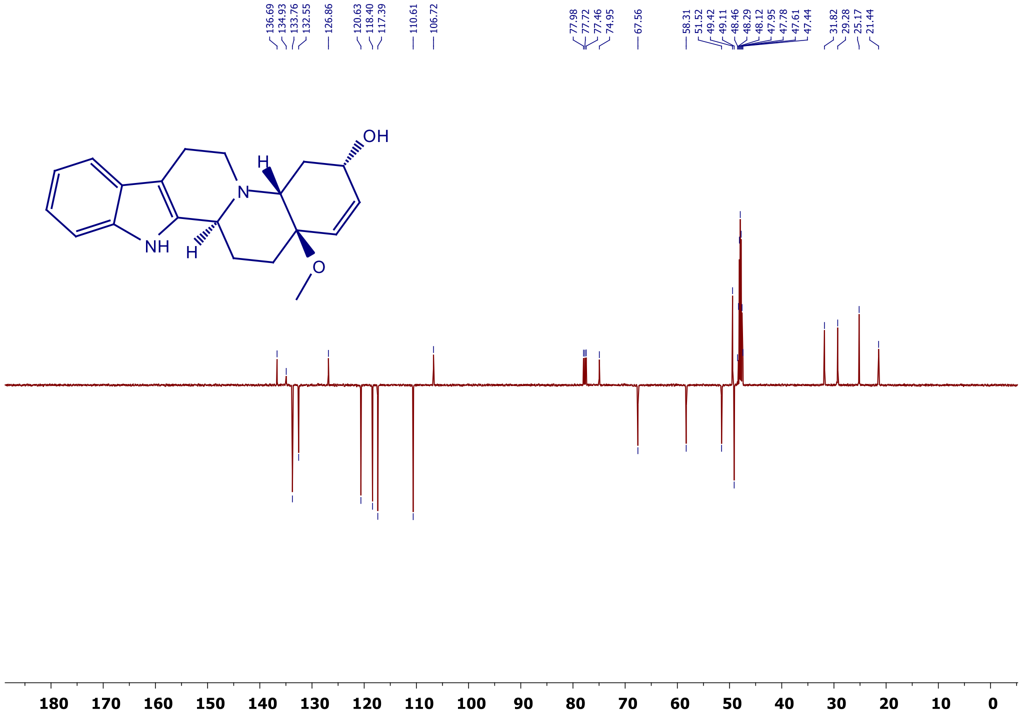

Supplementary Figure 170 APT NMR (125 MHz, CD_3OD) spectrum of (2aR,5S,6aR,14bS)-2a-methoxy-1,2,2a,5,6,6a,8,9,14,14b-decahydroindolo[2',3':3,4]pyrido[1,2-a]quinolin-5-ol (**25**)

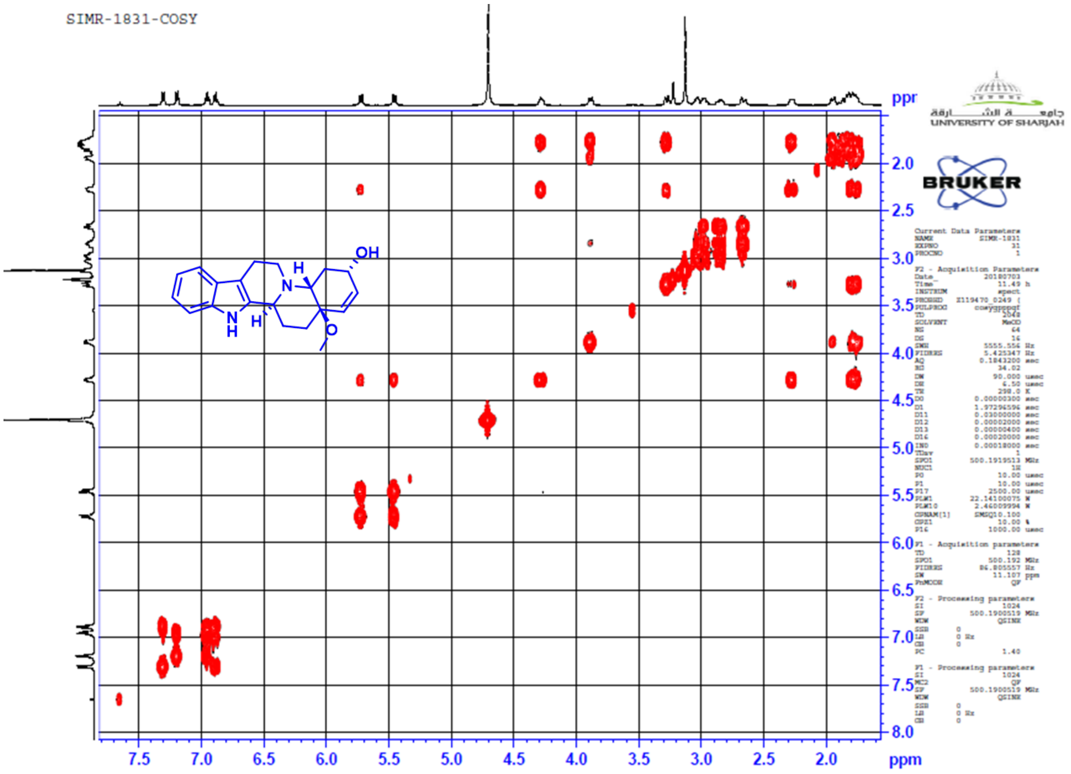

Supplementary Figure 171 COSY (500 MHz, CD$_3$OD) spectrum of (2aR,5S,6aR,14bS)-2a-methoxy-1,2,2a,5,6,6a,8,9,14,14b-decahydroindolo[2',3':3,4]pyrido[1,2-a]quinolin-5-ol (**25**)

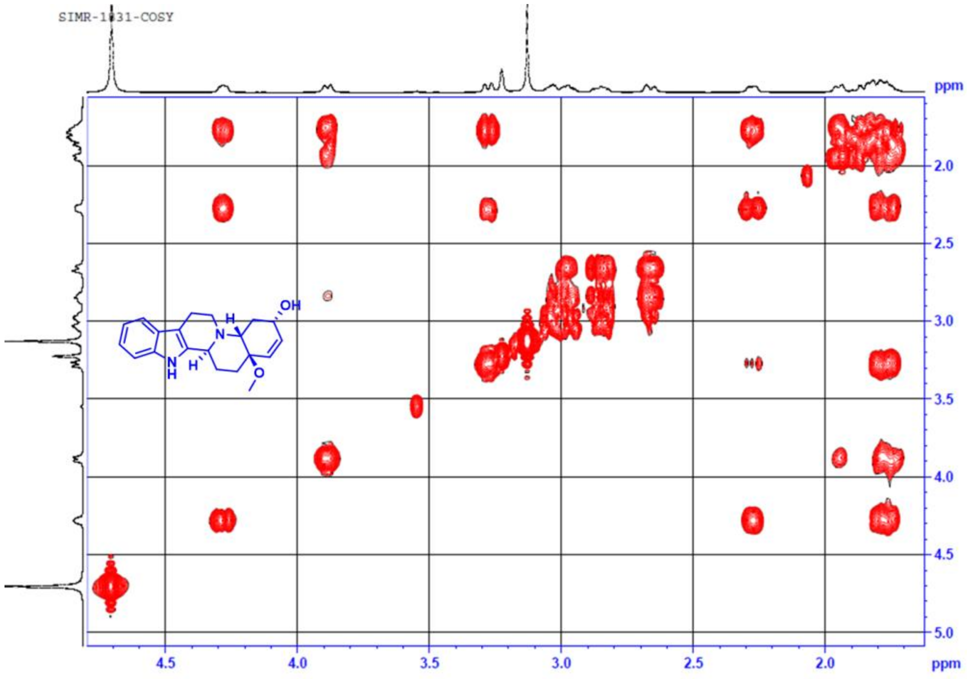

Supplementary Figure 172 COSY (Expansion) spectrum of (2aR,5S,6aR,14bS)-2a-methoxy-1,2,2a,5,6,6a,8,9,14,14b-decahydroindolo[2',3':3,4]pyrido[1,2-a]quinolin-5-ol (**25**)

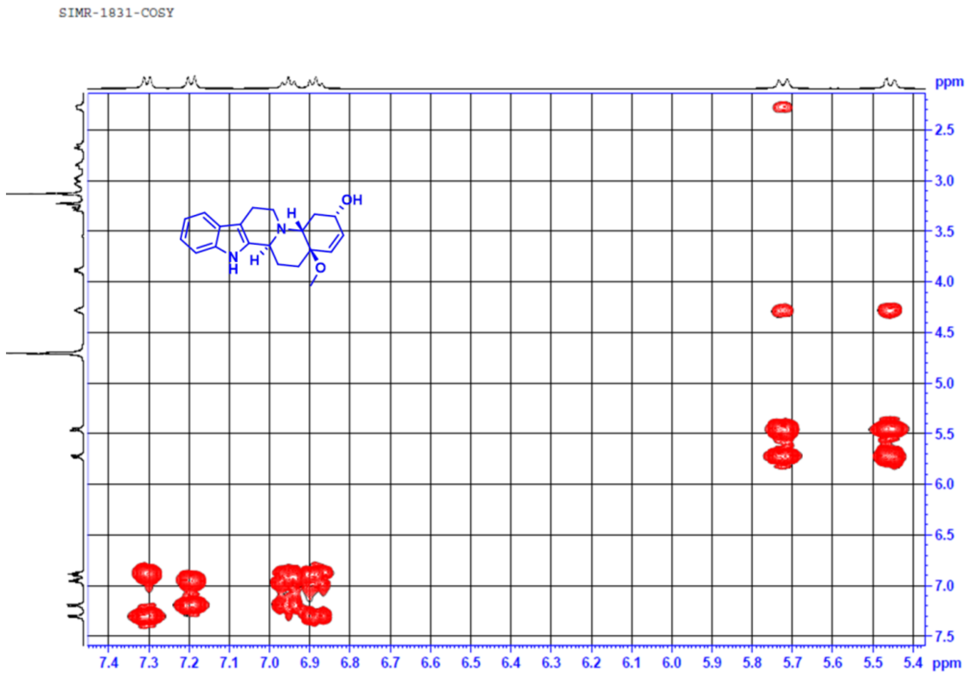

Supplementary Figure 173 COSY (Expansion) spectrum of (2aR,5S,6aR,14bS)-2a-methoxy-1,2,2a,5,6,6a,8,9,14,14b-decahydroindolo[2',3':3,4]pyrido[1,2-a]quinolin-5-ol (**25**)

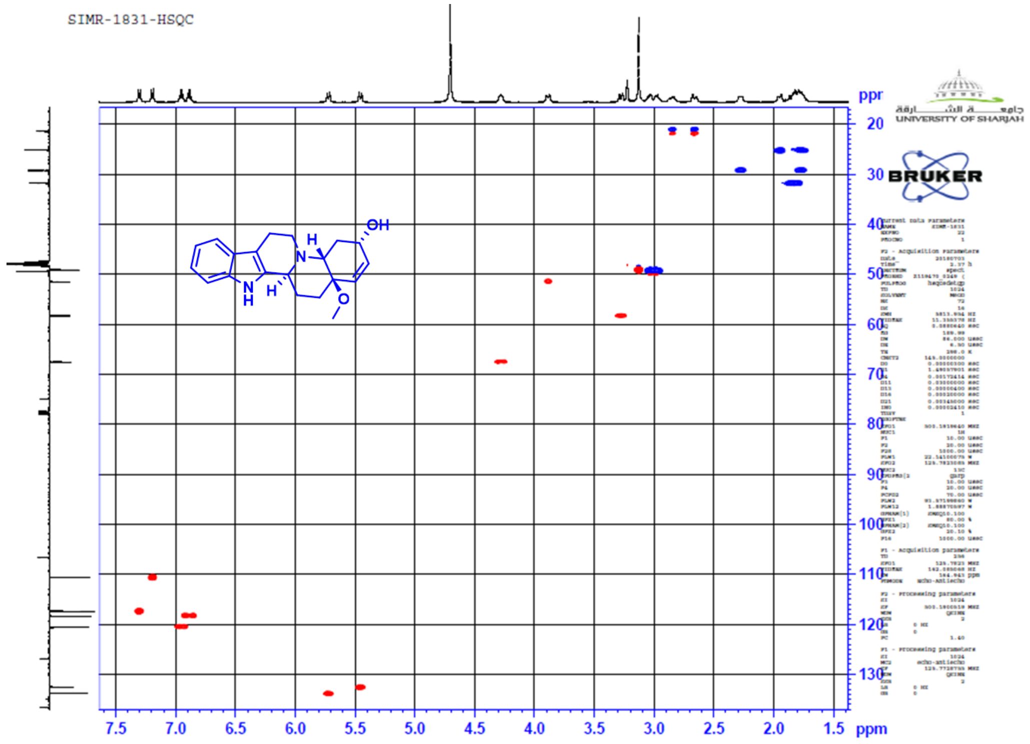

Supplementary Figure 174 HSQC (500 MHz, CD_3OD) spectrum of (2aR,5S,6aR,14bS)-2a-methoxy-1,2,2a,5,6,6a,8,9,14,14b-decahydroindolo[2',3':3,4]pyrido[1,2-a]quinolin-5-ol (**25**)

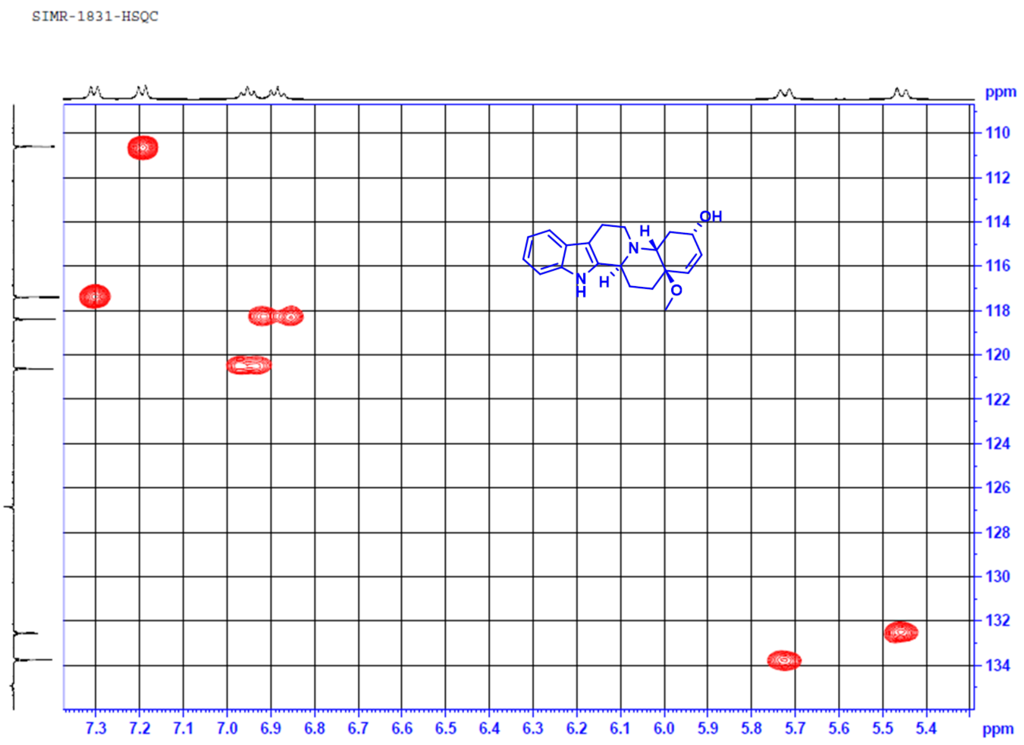

Supplementary Figure 175 HSQC (Expansion) spectrum of (2aR,5S,6aR,14bS)-2a-methoxy-1,2,2a,5,6,6a,8,9,14,14b-decahydroindolo[2',3':3,4]pyrido[1,2-a]quinolin-5-ol (**25**)

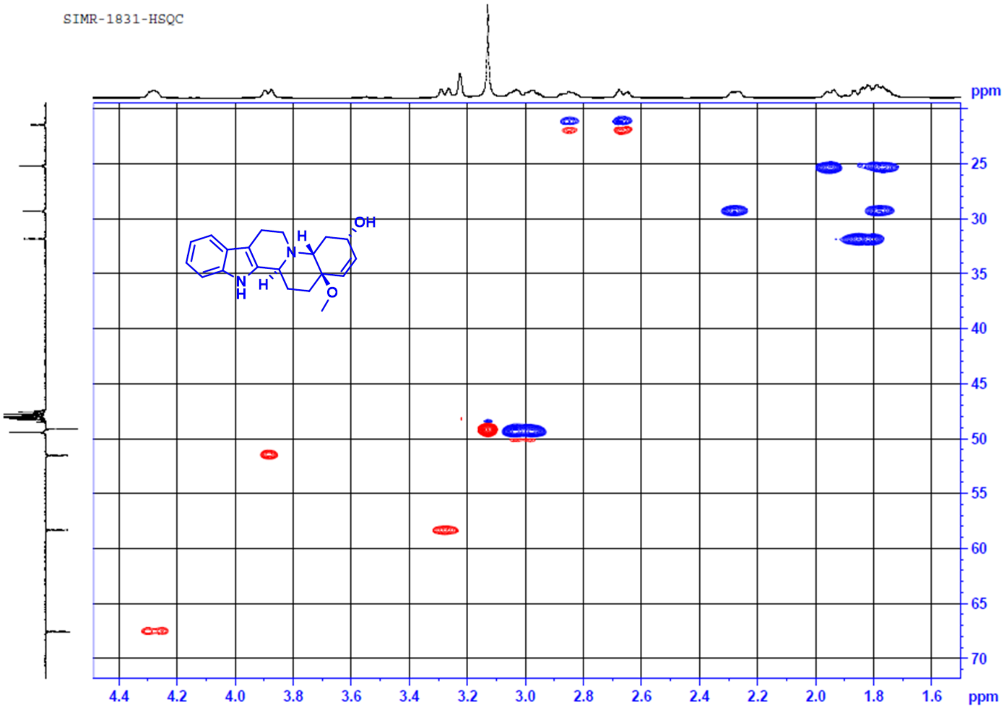

Supplementary Figure 176 HSQC (Expansion) spectrum of (2aR,5S,6aR,14bS)-2a-methoxy-1,2,2a,5,6,6a,8,9,14,14b-decahydroindolo[2',3':3,4]pyrido[1,2-a]quinolin-5-ol (**25**)

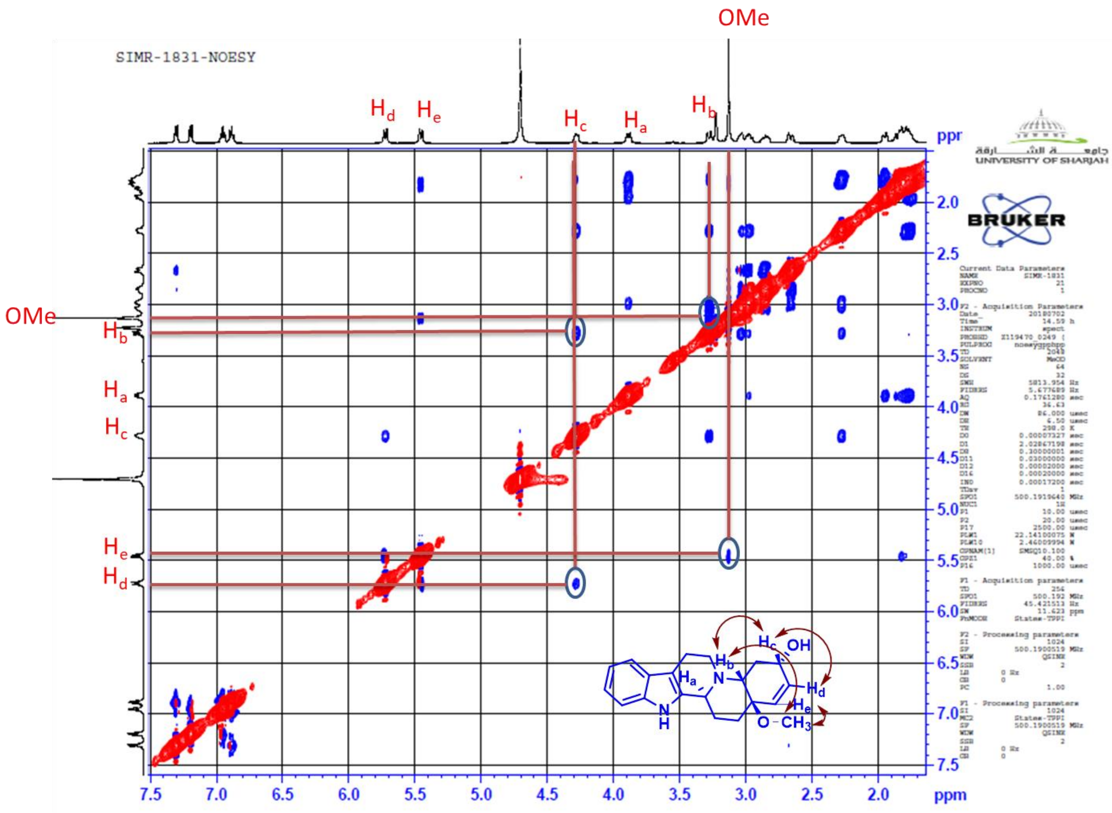

Supplementary Figure 177 NOESY (500 MHz, CD_3OD) spectrum of (2aR,5S,6aR,14bS)-2a-methoxy-1,2,2a,5,6,6a,8,9,14,14b-decahydroindolo[2',3':3,4]pyrido[1,2-a]quinolin-5-ol (**25**)

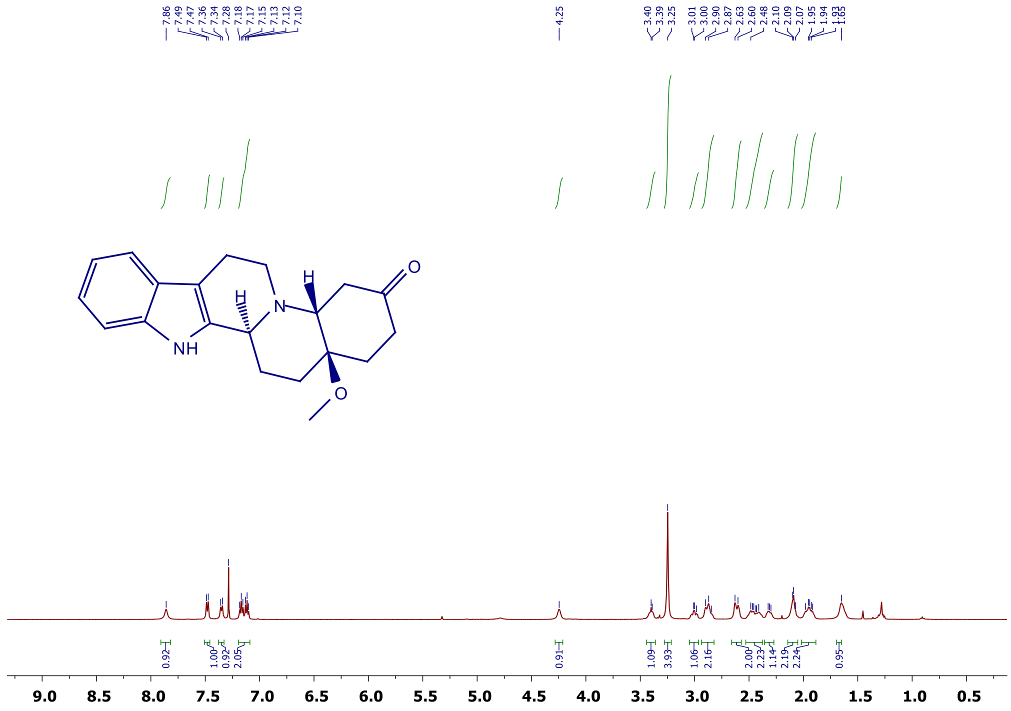

Supplementary Figure 178 ^{1}H NMR (500 MHz, $CDCl_3$) spectrum of (2aR,6aR,14bS)-2a-methoxy-2,2a,3,4,6,6a,8,9,14,14b-decahydroindolo[2',3':3,4]pyrido[1,2-a]quinolin-5(1H)-one (**26a**)

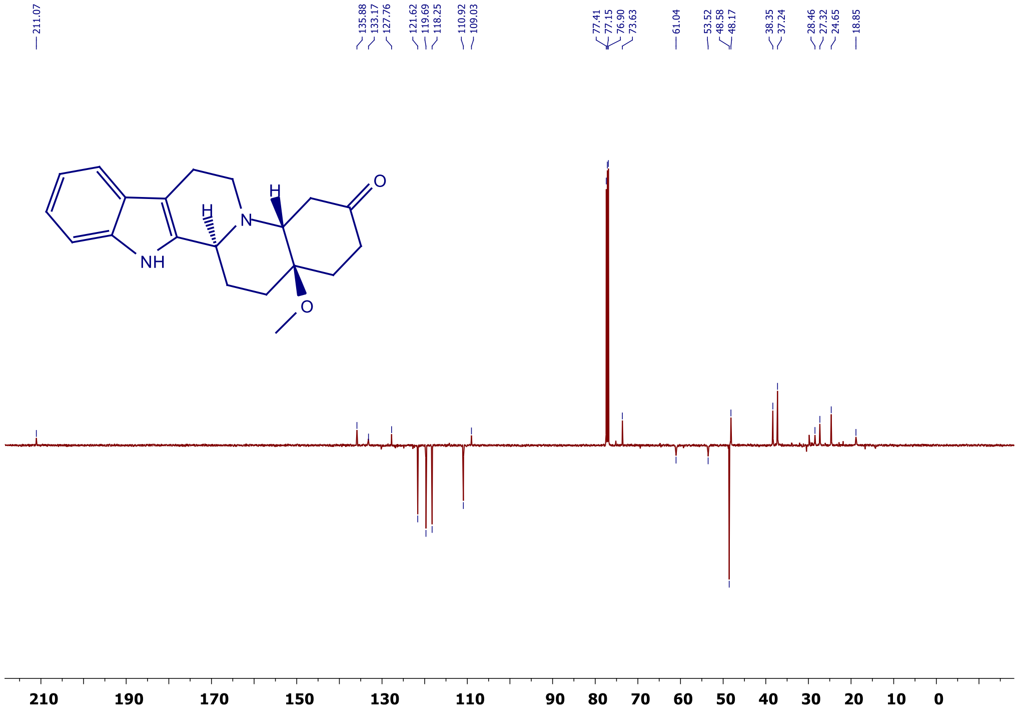

Supplementary Figure 179 APT NMR (125 MHz, $CDCl_3$) spectrum of (2aR,6aR,14bS)-2a-methoxy-2,2a,3,4,6,6a,8,9,14,14b-decahydroindolo[2',3':3,4]pyrido[1,2-a]quinolin-5(1H)-one (**26a**)

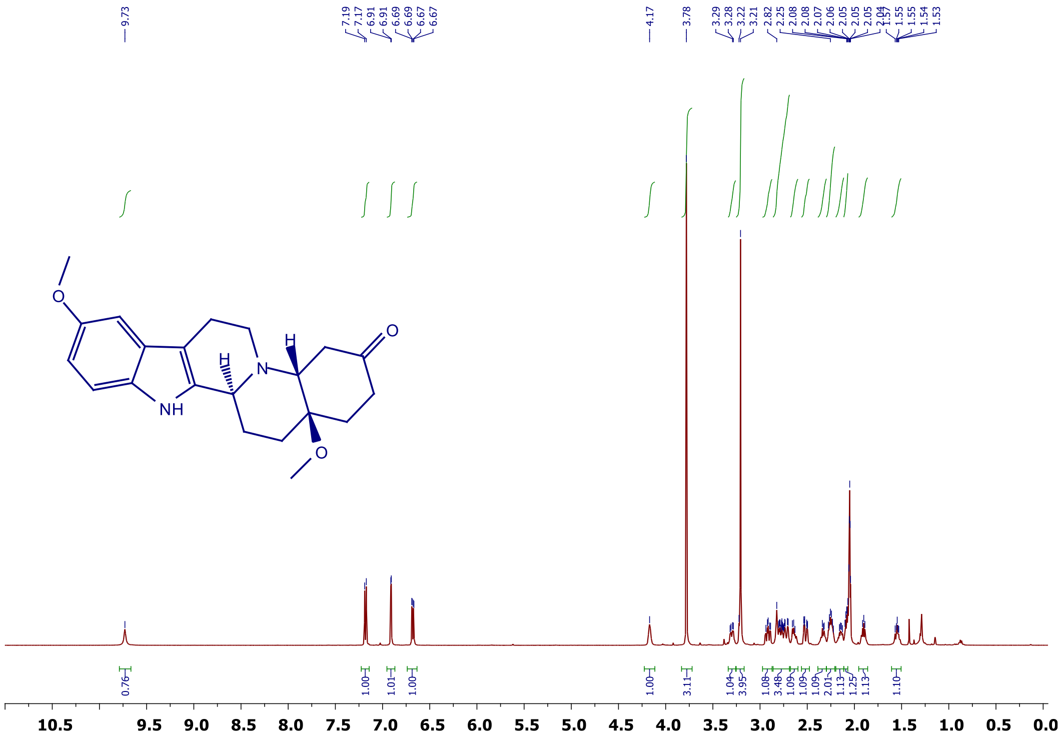

Supplementary Figure 180 ^{1}H NMR (500 MHz, Acetone-d_6) spectrum of (2aR,6aR,14bS)-2a,11-dimethoxy-2,2a,3,4,6,6a,8,9,14,14b-decahydroindolo[2',3':3,4]pyrido[1,2-a]quinolin-5(1H)-one (**26b**)

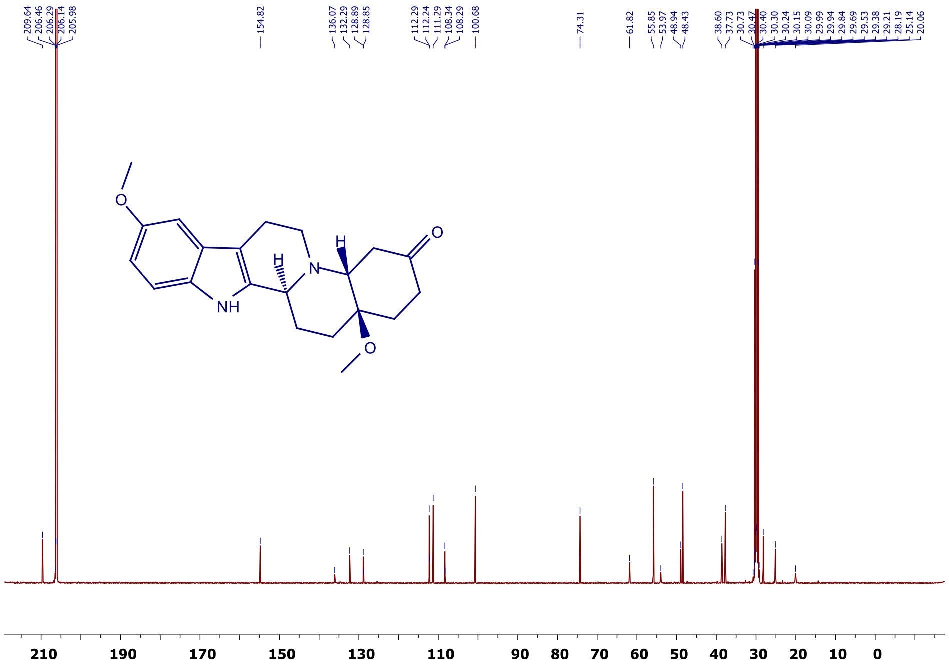

Supplementary Figure 181 ^{13}C NMR (125 MHz, Acetone-d_6) spectrum of (2aR,6aR,14bS)-2a,11-dimethoxy-2,2a,3,4,6,6a,8,9,14,14b-decahydroindolo[2',3':3,4]pyrido[1,2-a]quinolin-5(1H)-one (**26b**)

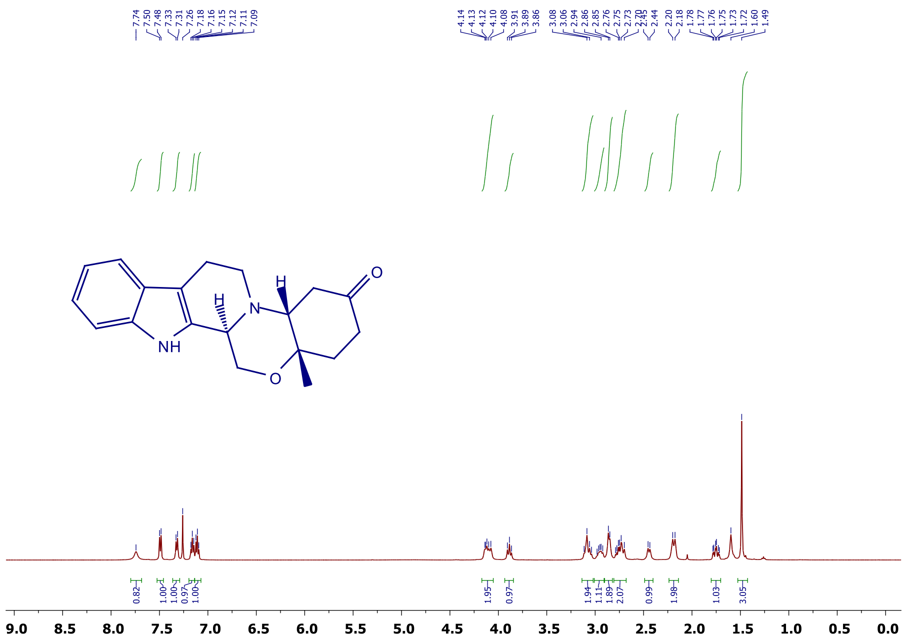

Supplementary Figure 182 ^{1}H NMR (500 MHz, $CDCl_3$) spectrum of (2aS,6aR,14bR)-2a-methyl-3,4,6,6a,8,9,14,14b-octahydro-1H-benzo[5',6'][1,4]oxazino[4',3':1,2]pyrido[3,4-b]indol-5(2aH)-one (**26c**)

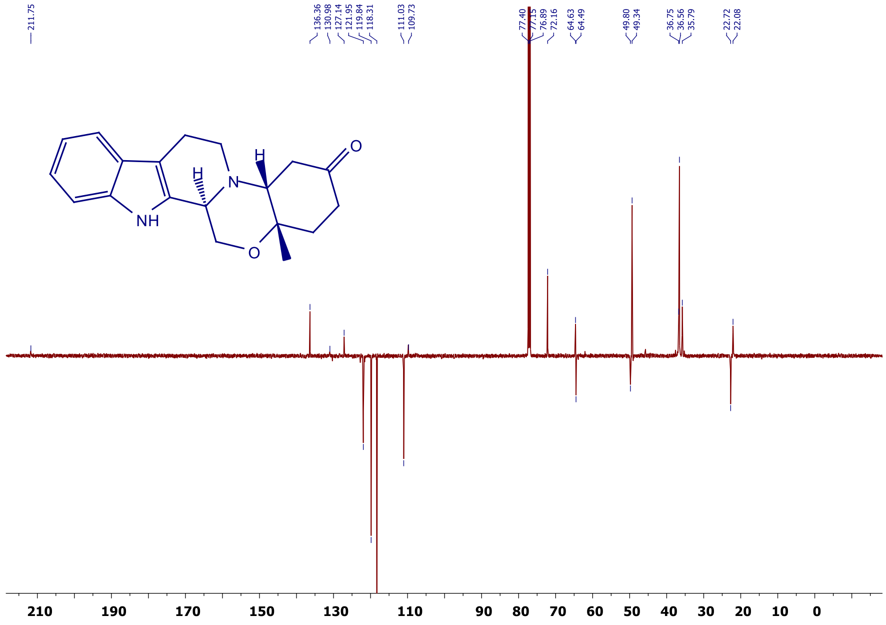

Supplementary Figure 183 APT NMR (125 MHz, $CDCl_3$) spectrum of (2aS,6aR,14bR)-2a-methyl-3,4,6,6a,8,9,14,14b-octahydro-1H-benzo[5',6'][1,4]oxazino[4',3':1,2]pyrido[3,4-b]indol-5(2aH)-one (**26c**)

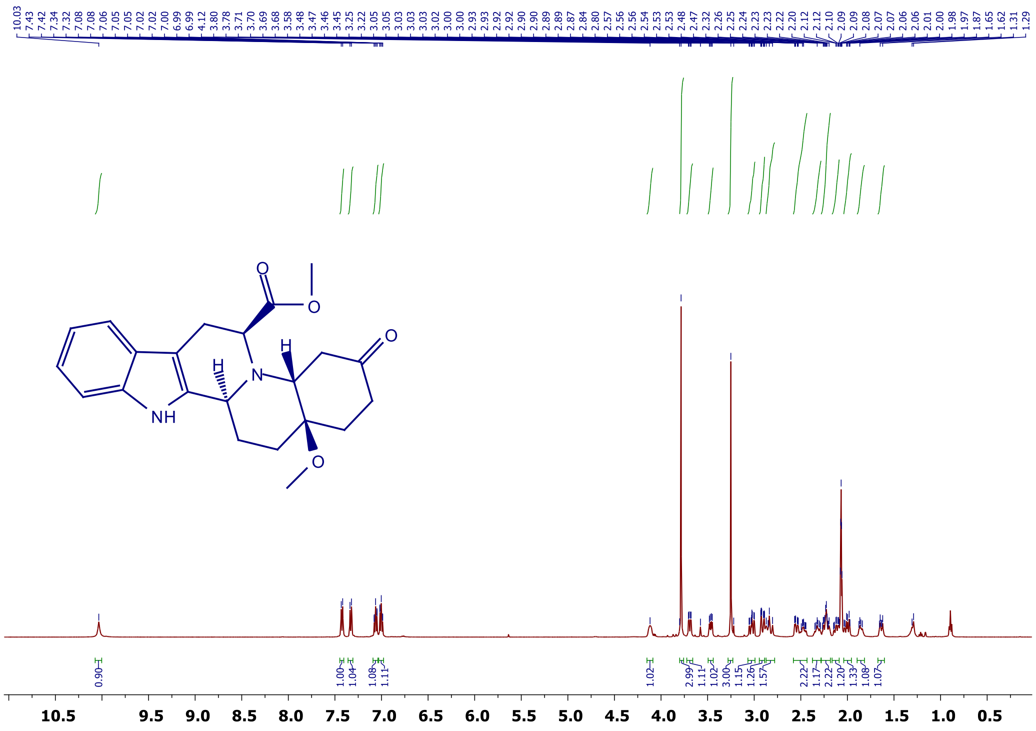

Supplementary Figure 184 ^{1}H NMR (500 MHz, Acetone-d_6) spectrum of methyl (2aR,6aR,8S,14bS)-2a-methoxy-5-oxo-1,2,2a,3,4,5,6,6a,8,9,14,14b-dodecahydroindolo[2',3':3,4]pyrido[1,2-a]quinoline-8-carboxylate (**26d**)

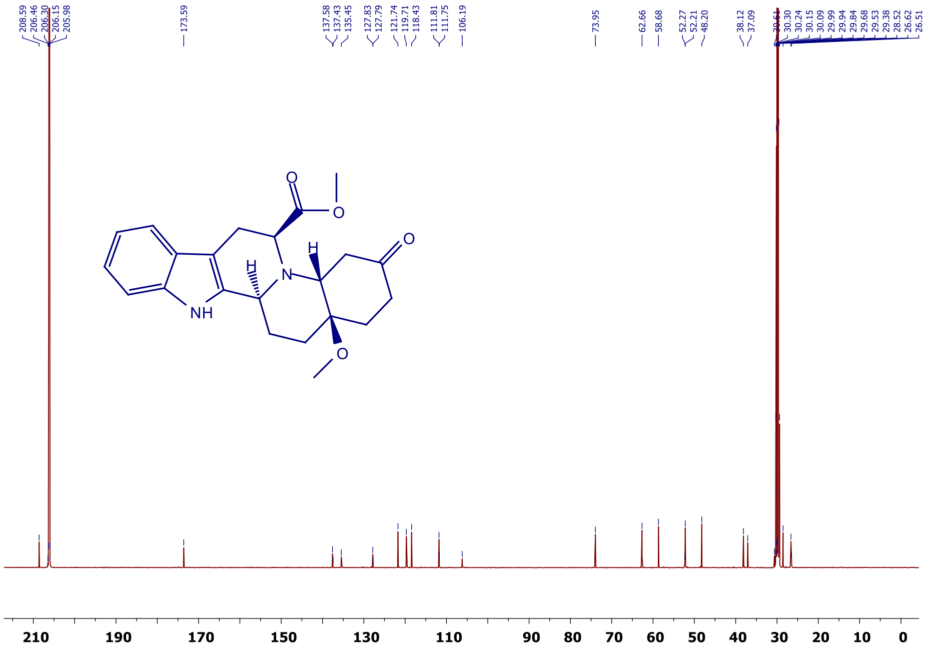

Supplementary Figure 185 ^{13}C NMR (125 MHz, Acetone-d_6) spectrum of methyl (2aR,6aR,8S,14bS)-2a-methoxy-5-oxo-1,2,2a,3,4,5,6,6a,8,9,14,14b-dodecahydroindolo[2',3':3,4]pyrido[1,2-a]quinoline-8-carboxylate (**26d**)

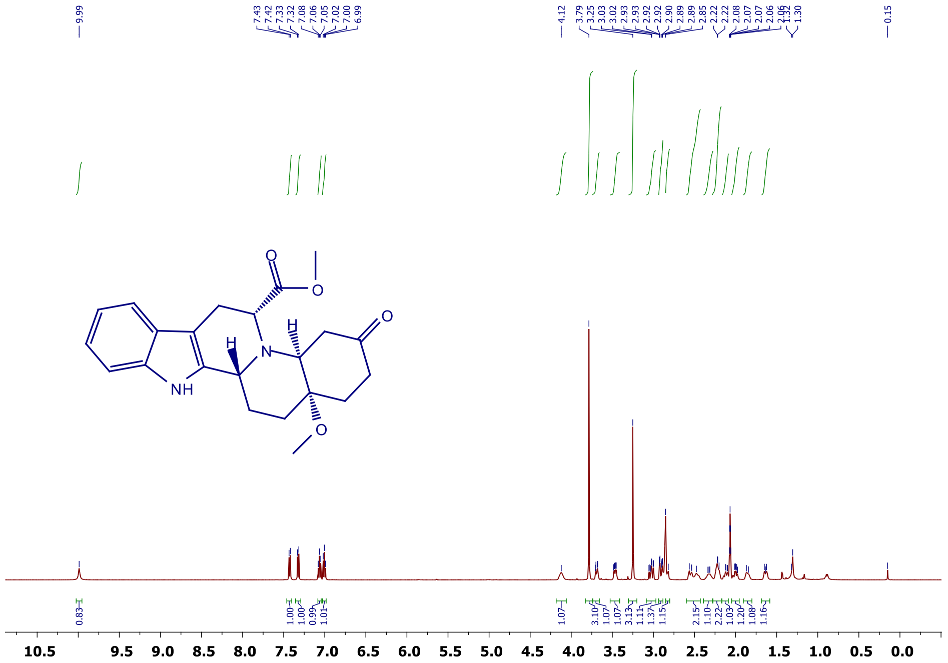

Supplementary Figure 186 ^{1}H NMR (500 MHz, Acetone-d_6) methyl (2aS,6aS,8R,14bR)-2a-methoxy-5-oxo-1,2,2a,3,4,5,6,6a,8,9,14,14b-dodecahydroindolo[2',3':3,4]pyrido[1,2-a]quinoline-8-carboxylate (**26e**)

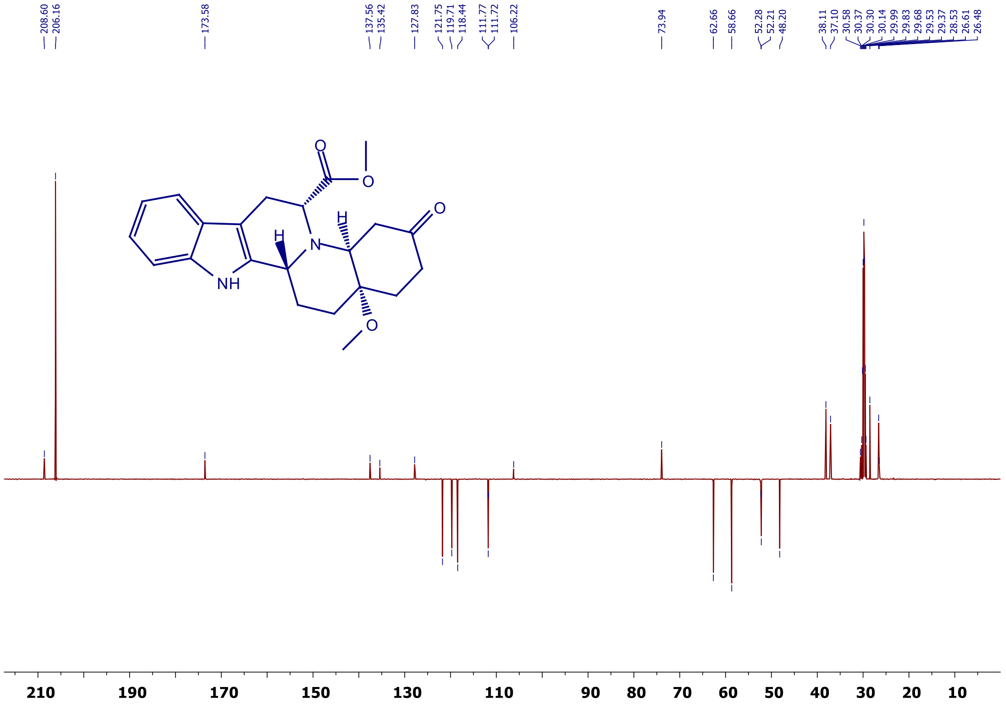

Supplementary Figure 187 APT NMR (125 MHz, Acetone-d_6) methyl (2aS,6aS,8R,14bR)-2a-methoxy-5-oxo-1,2,2a,3,4,5,6,6a,8,9,14,14b-dodecahydroindolo[2',3':3,4]pyrido[1,2-a]quinoline-8-carboxylate (**26e**)

Supplementary References

1. CrysAlis Software System (Version 1.171) (Oxford Diffraction Ltd. England: Oxford, 2002).

2. Dolomanov, O. V., Bourhis, L. J., Gildea, R. J., Howard, J. A. K. & Puschmann, H. *OLEX2*: a complete structure solution, refinement and analysis program. J. *Appl. Cryst.* **42**, 339-341 (2009).

3. Sheldrick, G. M. SHELXL-97, Program for X-ray Crystal Structure Refinement. Gottingen, Germany: University of Gottingen, 1997.

4. Sheldrick, G. M. Crystal structure refinement with *SHELXL*. *Acta. Crystallogr. C. Struct. Chem.* **A71**, 3-8 (2015)

5. Bourhis, L. J., Dolomanov, O. V., Gildea, R. J., Howard, J. A. K. & Puschmann, H. The anatomy of a comprehensive constrained, restrained refinement program for the modern computing environment – *Olex2* dissected. *Acta. Crystallogr. A* **A71**, 59-75 (2015).
